# Mapping structural aging across human tissues reveals tissue-specific trajectories and coordinated deterioration

**DOI:** 10.1038/s43587-026-01200-4

**Published:** 2026-08-31

**Authors:** Anamika Yadav, Kyle Alvarez, Anna Chechenina, Kevin Y. Yip, Eytan Ruppin, Alexandre Colas, Jacqueline C. Yano Maher, Veronica Gomez-Lobo, Caroline Kumsta, Sanju Sinha

**Affiliations:** 1 Center for Data Science and Artificial Intelligence, Sanford Burnham Prebys Medical Discovery Institute, La Jolla, CA USA; 2 Cancer Data Science Lab, National Cancer Institute, National Institutes of Health, Bethesda, MD USA; 3 Center for Cardiovascular and Muscular Diseases, Sanford Burnham Prebys Medical Discovery Institute, La Jolla, CA USA; 4 Pediatric and Adolescent Gynecology, Eunice Kennedy Shriver National Institute of Child Health and Human Development, Bethesda, MD USA; 5 Present Address: Translational Research Institute and Jim and Eleanor Randall Department of Surgery, Cedars-Sinai Medical Center, Los Angeles, CA USA

**Keywords:** Machine learning, Ageing, Computational biology and bioinformatics

## Abstract

Organ structure, including the organization of cells, vasculature and extracellular matrix, underpins its function, yet how structure changes with age remains mostly unknown. Here we developed PathStAR, a framework that quantifies tissue structural aging from routine histopathology images, without being trained to predict chronological age. Applying PathStAR to 25,306 post-mortem biopsies from 40 tissues in 970 donors aged 21–70 years revealed that organ structural aging progresses via distinct, nonlinear temporal trajectories: vascular tissue structural aging accelerates early, uterus and vagina structural aging accelerates late (around menopause) and certain tissues including digestive and male reproductive organs show biphasic accelerations. We show that accelerations of structural aging are characterized across organs by increased inflammation alongside reduced energy production, repair and quality control. Cross-organ analysis reveals coordinated deterioration within individuals, including digestive and male reproductive tissues, linked by sex hormones. Together, our analysis provides a systematic map of structural aging across the human body.

## Abstract (web summary)

Yadav et al. develop a framework that quantifies tissue structural aging without training on chronological age, using a large dataset of human histology images. They reveal distinct yet coordinated organ aging trajectories, and link accelerated aging to pathways, genetic variants and pathology.

## Main

Aging is the biggest risk factor for most chronic diseases^1^. Current aging research has advanced our molecular understanding of aging, including both cell-intrinsic processes such as telomere shortening, senescence and autophagy, as well as systemic pathways such as the insulin/IGF-1 axis^1–4^. Molecular aging clocks have improved prediction of risk of mortality and diseases^5,6^. Recent studies further show that molecular aging unfolds nonlinearly, pointing to the possibility of time-period-specific targeting^7,8^.

While we have advanced our knowledge of molecular, cellular and systemic changes during aging, a critical missing gap remains: how the physical structure of tissues, the spatial organization of cells, vasculature and extracellular matrix, and the basis of their function, deteriorate with aging (structural aging). Our current understanding of structural aging comes from manual inspection of histology images revealing both global deterioration patterns such as fiber fragmentation^9,10^, atrophy^11^ and fibrosis^12^ as well as tissue-specific patterns such as ovarian follicle loss^13^, bone porosity^14^, brain cortical thinning and demyelination, muscle atrophy^15^, arterial stiffening^16^, heart fibrosis^17,18^, thymic involution^19^ and lens clouding^20^. These structural changes cause multiple known organ function loss during age, such as ovarian follicle loss reducing fertility and cardiac fibrosis increasing heart failure risk. While scan-based histology clocks are trained on chronological age^21,22^, there is no systematic study asking how organ structure changes during aging. This gap has persisted due to lack of large-scale high-resolution tissue images across the body and computational tools to quantify these tissue substructures.

These limitations have given rise to several fundamental open questions about aging: When and how do different tissue physical structures deteriorate during aging? What are their molecular drivers? Are there tissue-specific periods of accelerated structural aging (ASA), and what molecular dysregulation drives this acceleration? Which organs are early-agers versus late-agers? Do organs age independently or in coordination? How do clinical factors accelerate or protect against structural aging? And, finally, how do germline variants affect organ-specific structural aging?

We present PathStAR (Pathology-based Structural Aging Rate), a framework that quantifies tissue structural aging from histopathology images without training on chronological age. We applied it to ~25k post-mortem biopsies across 40 tissues from 970 Genotype-Tissue Expression (GTEx) donors aged 21–70 years. Unlike age-prediction clocks, PathStAR resolves when structure changes: it recovers the ovary’s nonlinear decline, fertility loss in the 30s and menopause in the 50s. It then maps three temporal aging programs, coordinated cross-organ deterioration, molecular drivers of each accelerated period, and germline variants tied to organ-specific trajectories, establishing structural aging as a quantifiable dimension of aging that complements molecular clocks.

## Result

### Overview of the PathStAR pipeline

**Fig. 1:**
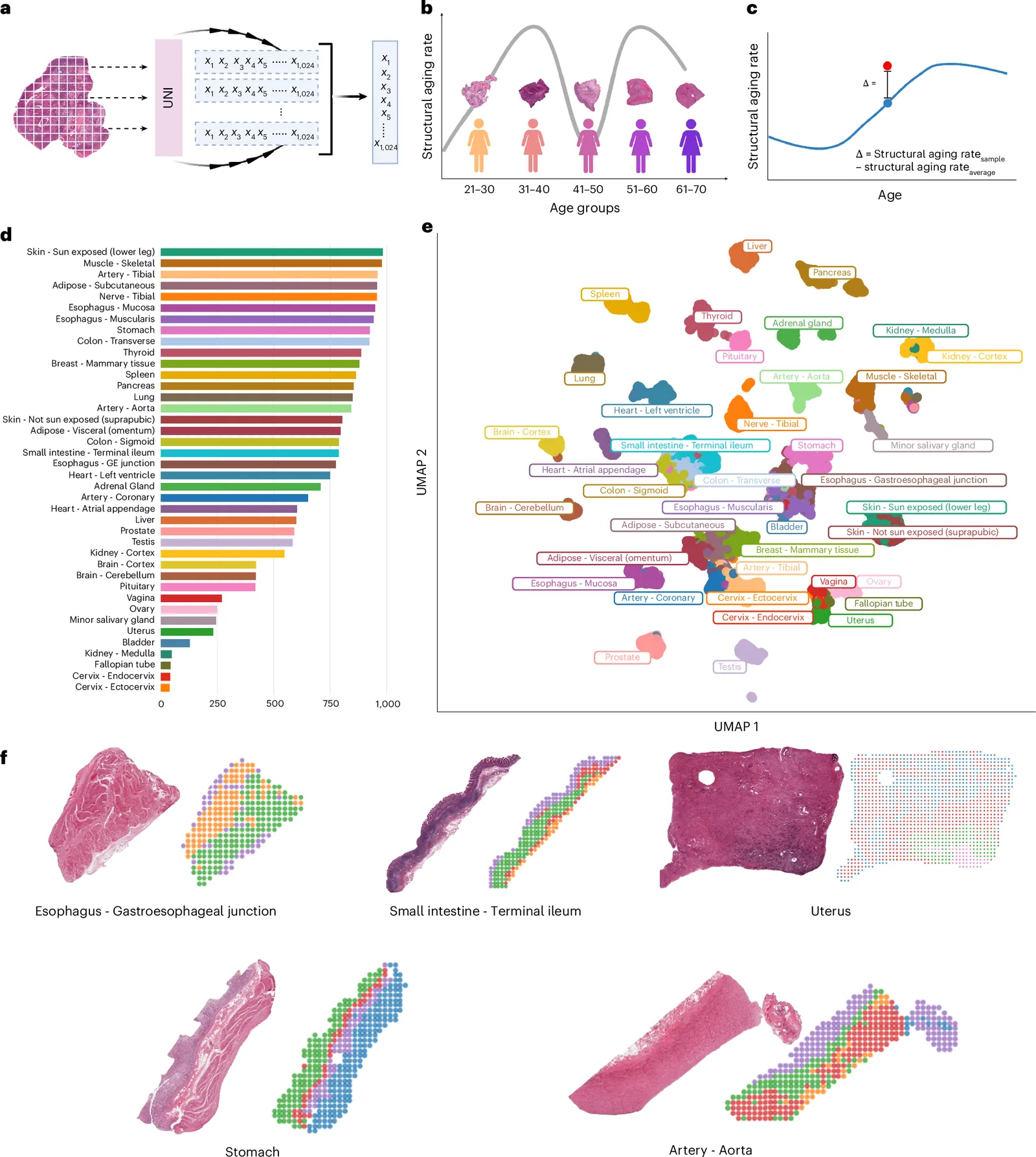
Overview of PathStAR pipeline steps and the GTEx cohort used here. **a**–**c**, PathStAR computational pipeline: PathStAR starts by extracting morphology feature of 256 × 256 pixels patch level using a pretrained pathology foundation model (UNI), which are aggregated (mean pooling) to generate slide-level representation (**a**). It then computes structural aging rate by quantifying the rate of morphological change per year using sliding 10-year windows, generating age-specific trajectories that reveal periods of ASA (**b**). Finally, it computes individual delta-structural aging scores (red) measuring each sample’s deviation from the population aging trajectory (blue) (**c**). **d**, Number of samples available for this study per tissue type. **e**, Uniform Manifold Approximation and Projection (UMAP) based on whole-slide level representation cluster different tissue types, demonstrating its ability to capture tissue type-specific morphological properties. **f**, Morphological patch level features from UNI clustered using spatial information and *K*-means, where different colors represent different clusters. Figure created in BioRender; Alvarez, K. https://biorender.com/5o5bsgk (2026).

Based on the notion that histology images capture architecture features critical for tissue function, we developed a computational framework called PathStAR (Fig. 1a–c), that quantify how and when this tissue structure changes during aging from high-resolution hematoxylin and eosin (H&E) images of large-scale post-mortem biopsies.

**Extended Data Fig. 1:**
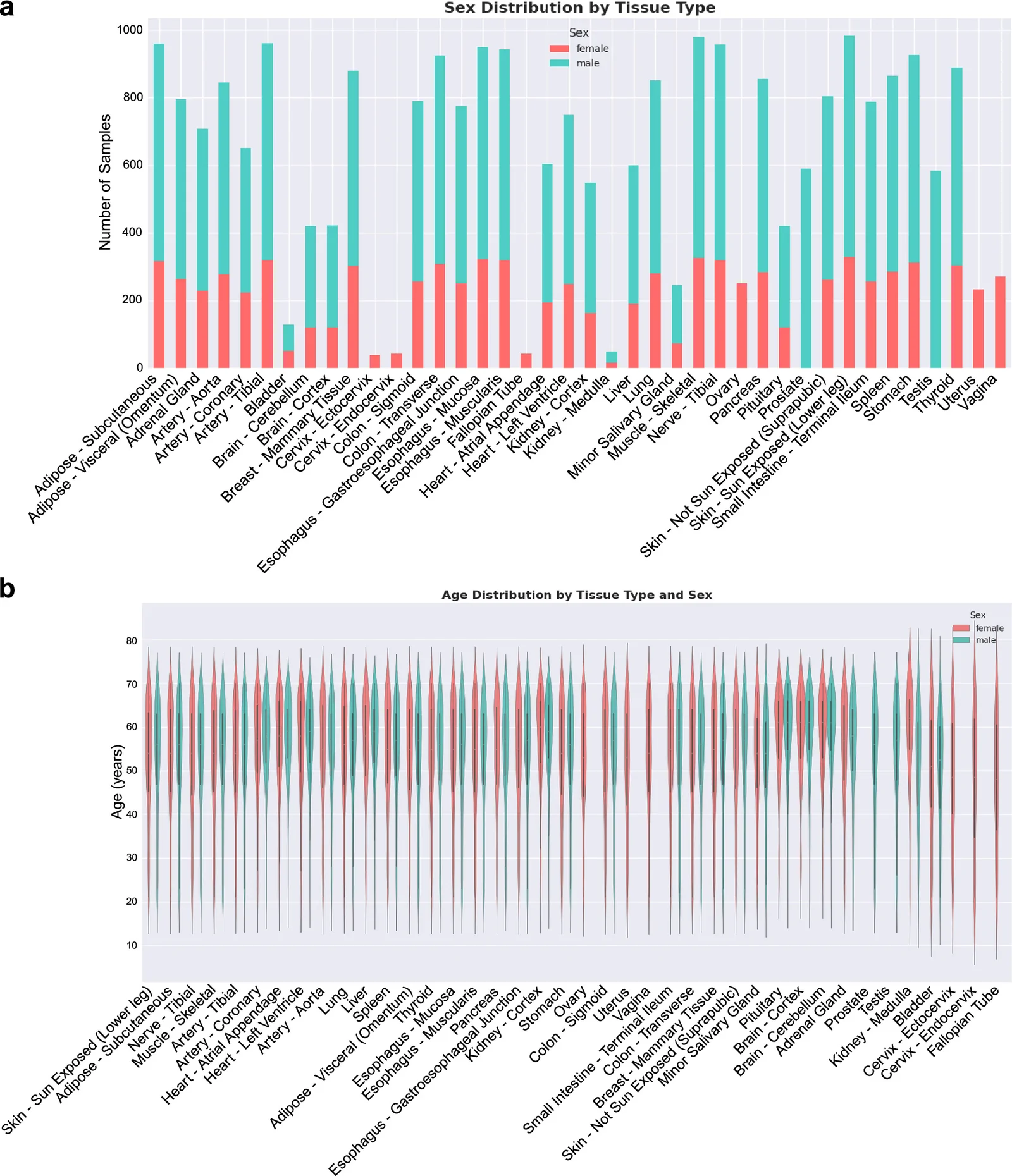
Sex and Age Visualization of GTEx cohort. **a**, The distribution of data for male donor samples vs female donor samples. **b**, Age distribution for both male and female donor samples for each tissue. The male donor samples comprised of of 66.4% (16808 samples) and female donor samples comprised of 34.6% (8498). Most data come from older donors with ages above 45 years. Figure created in BioRender; Alvarez, K. https://biorender.com/cczqv5r (2026).

PathStAR was applied on the GTEx cohort^23^, post-mortem biopsies from 970 nondiseased individuals (age range 21–70 years, 66.4% male donors; Extended Data Fig. 1), spanning 40 tissue types (Fig. 1d). GTEx provided a total of 25,306 organ biopsy whole-slide scans (×20 magnification), from which we extracted 30.3 million patch images (512 × 512 pixels). Pathology annotations from GTEx for these images confirmed canonical aging-related increase in atrophy, fibrosis, cysts and atherosclerosis and decrease in ovum, spermatogenesis and protective layers including mucosa, endometrium and myometrium (Supplementary Figs. 1 and 2), demonstrating quality to develop a systematic method. The tissue-specific quality control analysis is presented in Supplementary Note 1.

PathStAR comprises three steps (see details in the Methods):

1. Step 1: feature extraction from whole-slide images. After background removal, each slide is segmented into patches, each encoded into a 1,024-dimensional embedding via UNI^24^, a vision transformer (ViT) pretrained on more than 100,000 whole-slide images. Mean pooling yields one slide-level representation per sample (Fig. 1a). These capture tissue-specific morphology, separating distinct tissues and substructures in low-dimensional space (Fig. 1e,f). We tested multiple encoders to ensure results are not encoder-specific artifacts (Supplementary Notes 2).
2. Step 2: construction of structural aging trajectories. We next asked: when does tissue structure change most rapidly during aging? For each tissue, we quantify the per-year rate of structural change, the structural aging rate, by comparing morphological representations between consecutive 10-year age windows using an effect-size measure. Sliding the window in 1-year increments yields a continuous, population-level trajectory (ages ~30–60) that distinguishes rapid from stable periods; intervals of pronounced acceleration define ASA periods.
3. Step 3: individual-level deviation from population trajectories. With population-level trajectories established, we next ask: which individuals are aging faster or slower than expected for their tissue and age? For each sample, we compute an individual structural aging score and measure how much a given tissue in a given individual deviates from its expected aging path (Methods and Fig. 1c, delta-structural aging score). These scores enable identification of individuals with accelerated or protected structural aging, and downstream analysis of the clinical, lifestyle and genetic factors that drive these deviations.

### PathStAR captures ovary’s functional decline and its molecular correlates without chronological age training

#### Morphology captures ovary’s nonlinear functional decline without supervision

**Extended Data Fig. 2:**
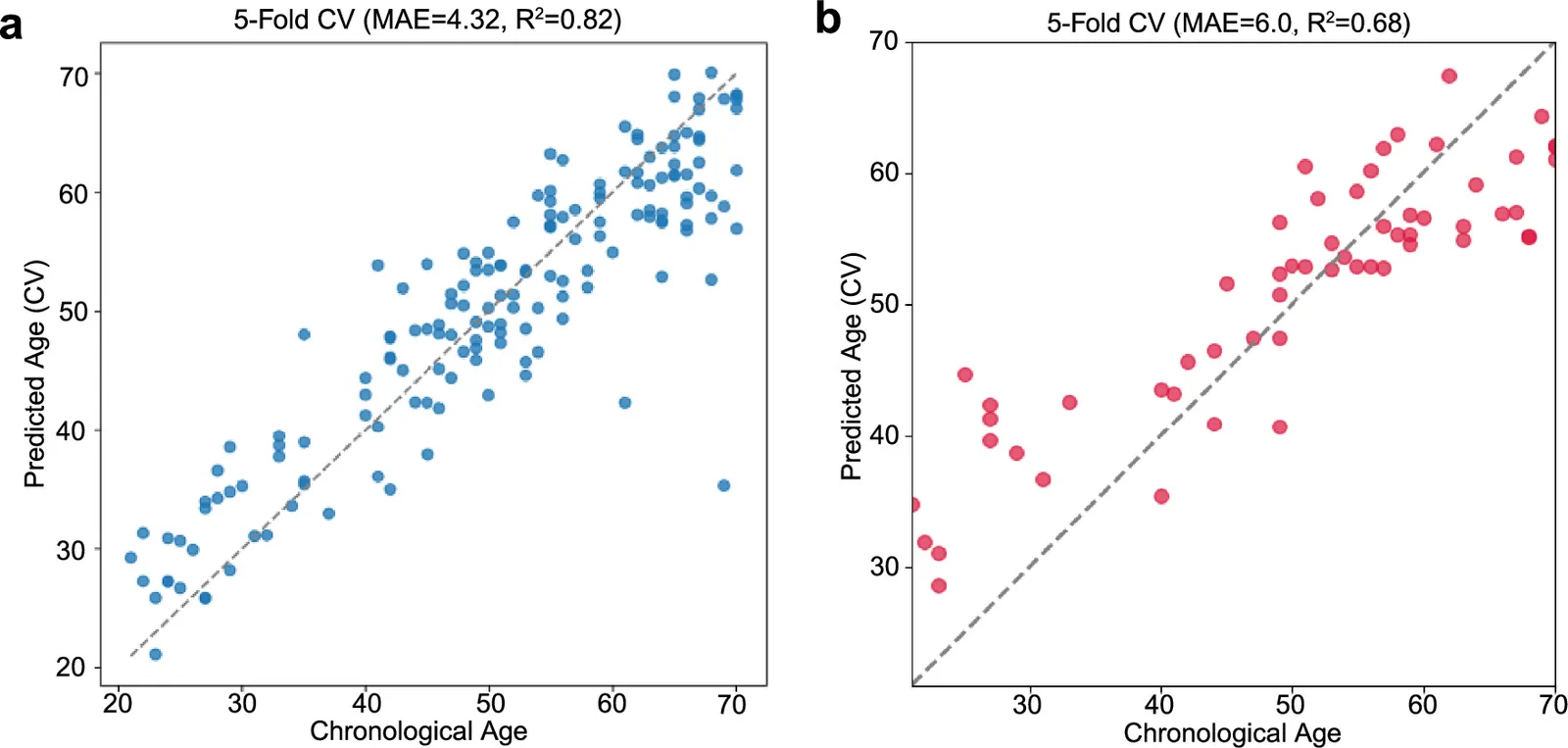
Methylation and Gene Expression-Based Clocks Ovary. Performance comparison of age prediction models using 5-fold cross-validation, where x-axis represents chronological age and y-axis shows predicted age. **a**, methylation-based elastic net model shows high accuracy (MAE = 4.32 years, R^2^ = 0.82). **b**, Elastic net gene expression model demonstrates moderate accuracy (MAE = 6.0 years, R^2^ = 0.68). Dashed diagonal lines indicate perfect prediction. Figure created in BioRender; Alvarez, K. https://biorender.com/cczqv5r (2026).

The ovary exhibits a unique functional decline: fertility decreases gradually from the early 30s, marked by rapid follicular depletion accelerating through the late 30s, culminating in menopause at ~50–55 years^25,26^. Molecular clocks trained on chronological age assume linear aging and thus cannot capture this nonlinear remodeling (Extended Data Fig. 2). We therefore tested whether PathStAR can capture this established nonlinear functional decline.

**Fig. 2:**
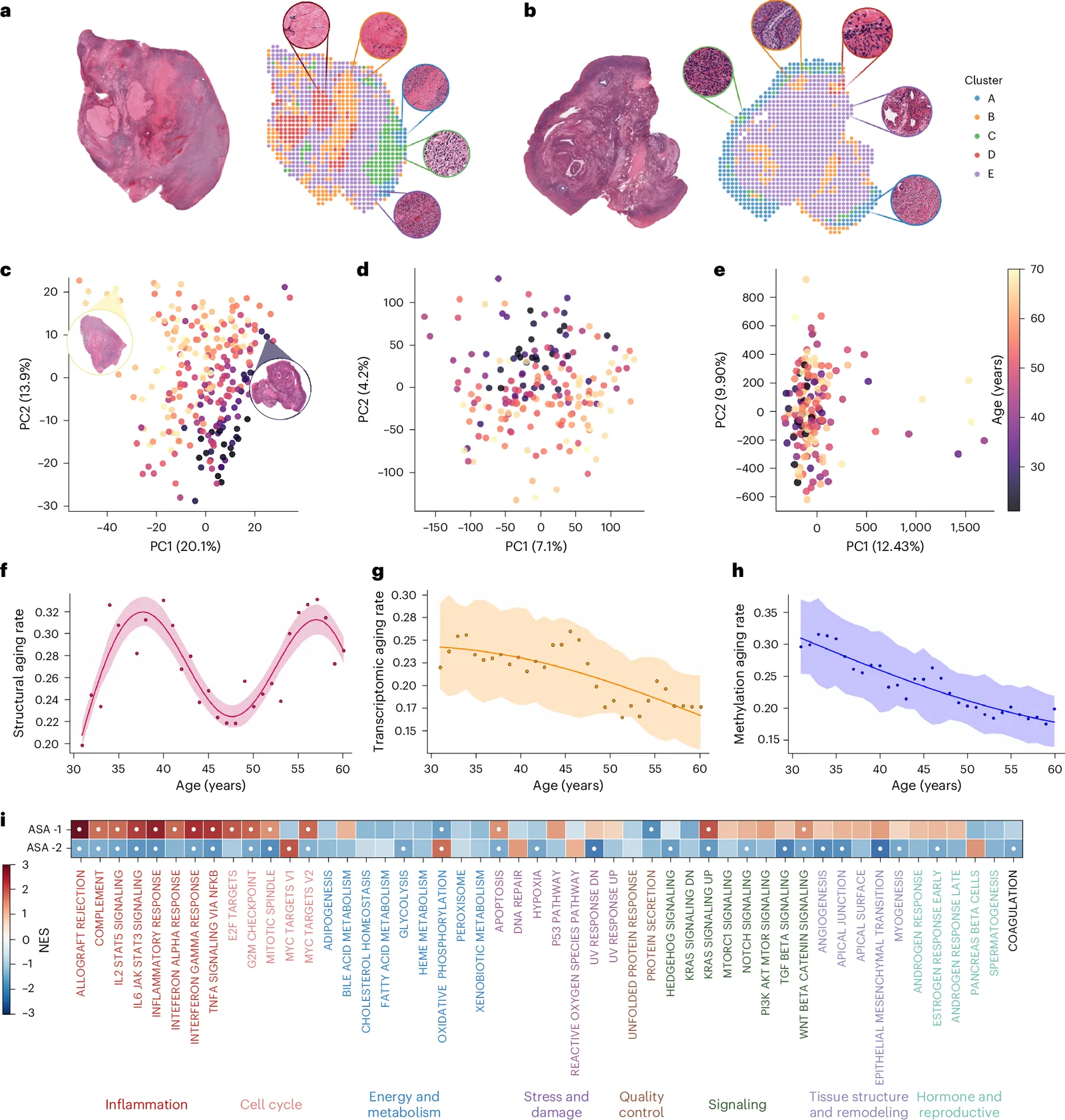
Morphology features capture nonlinear ovary structural aging that bulk-molecular profiles cannot. **a**,**b**, The morphological patch level features from two ovary samples from both older (**a**, left, age 60–69) and younger (**b**, right, age 20–29). The cluster shows that UNI can very well capture the structural property of the tissue. **c**,**e**, PC analysis of all morphology features extracted by UNI in 250 ovary samples from whole-slide images separates young versus old samples, without prior training on chronological age: expression (**c**) and methylation (**e**) profiles from the matched samples. **f**, PathStAR applied to UNI features from 250 ovarian samples, age 21–70 years, captures nonlinear structural aging. Structural aging rate was estimated using sliding 10-year age windows (for example, 21–30 versus 31–40), advanced in 1-year increments across the age span. Each data point represents the structural difference between two adjacent windows and is positioned at the lower bound of the older window. Windows containing insufficient donor representation were excluded, yielding 28 retained trajectory points derived from the full cohort. Trajectory points were retained for smoothing when more than 5% of UNI features were significant. Trajectory points were considered significant when ≥5% of UNI features had nominal *P* < 0.0. *P* values were not adjusted for multiple comparisons, yielding 27 retained points. Lines show mean values, and shaded bands represent 95% confidence intervals. **g**, Expression profiles can segregate young versus old samples but fail to capture the bimodal pattern in the structural aging trajectory of ovary. **h**, Methylation profiles fail to capture young versus old samples and do not reflect the bimodal pattern captured by the structural aging trajectory. **i**, Heatmap showing hallmark pathway enrichment in ASA-1 and ASA-2 compared with their corresponding control age windows. Gene-level differential expression was assessed using two-sided Welch’s *t*-tests, and genes were ranked by sign (log_2_ fold change) × −log_10_(*P*). Pathway enrichment was assessed using preranked GSEA with 10,000 permutations. White dots denote pathways with GSEA FDR *q* value ≤0.10. Figure created in BioRender; Alvarez, K. https://biorender.com/5o5bsgk (2026).

Patch-level UNI features spatially segregated distinct ovary substructures (Fig. 2a–c and Supplementary Fig. 3) and readily distinguished young, middle-aged and old samples, with the first two principal components (PCs) correlating with age (*r*^2^ = 0.43), indicating substantial age-related structural change (Fig. 2c). Computing the structural aging rate trajectory across 250 ovary biopsies (ages 21–70; Fig. 2f) yielded a striking bimodal pattern, with two accelerated-structural-aging peaks aligning with reproductive milestones (Fig. 2f and Methods): the first ASA period peaks at ages 35–40, coinciding with fertility decline, and the second at ages 55–60, corresponding to menopause. Ischemic time and other post-mortem artifacts showed minimal correlation with this trajectory (Supplementary Note 3 and Supplementary Fig. 4).

In comparison, when this exact unsupervised trajectory analysis is applied to bulk-expression and methylation profiles (Fig. 2d,e) from the same samples, neither expression nor methylation trajectory recapitulated the bimodal pattern of structural trajectory (Fig. 2g,h and Methods). Interpreting the two ASA periods with a pathology vision–language model^27^ (see quality control in Supplementary Note 4 and Supplementary Fig. 5) revealed increased fibrosis and atrophy in both, with a pronounced drop in ovum abundance during ASA-2 (Supplementary Fig. 6), consistent with follicular depletion and post-menopausal involution. Morphological feature groups with distinct age-related patterns (increasing, decreasing or stable) are described in Supplementary Note 5 and Supplementary Figs. 7 and 8.

In summary, this nonlinear trajectory emerged from histology without any training on chronological age, a capability that neither unsupervised molecular analysis nor linear-by-design molecular clocks can provide.

#### Molecular changes during ASA periods of ovary

Having established the two ASA periods from histology, we next asked what molecular changes accompany each structural transition. We compared bulk-expression and methylation profiles from each ASA period to its preceding structurally stable phase (Fig. 2i): ASA-1 (ages 35–40) versus baseline (ages 25–30), and ASA-2 (ages 55–60) versus baseline (ages 45–50). For each contrast, we performed differential expression analysis between age groups, ranked genes by signed effect size and statistical significance, and conducted pathway enrichment analysis on the resulting ranked lists to identify coordinated biological programs associated with each structural transition.

ASA-1 is marked by upregulation of multiple inflammatory pathways (TNF via NFKB, IL2-STAT5 and others; Fig. 2i). Together, these changes suggest a pro-inflammatory microenvironment coinciding with rapid loss of ovarian reserve^28–32^. By contrast, ASA-2 is uniquely characterized by downregulation of growth factor signaling (TGF-β), epithelial-mesenchymal transition (EMT) and cell cycle pathways, consistent with loss of follicular activity and reduced steroidogenesis in the postmenopausal period^33–35^ (Fig. 2i). These period-specific molecular programs were identifiable only because PathStAR defined the structural windows from histology, establishing the foundation for whole-body analysis described next.

### Structural aging dynamics in different tissues across the whole human body

#### Morphological features segregate young versus old tissues for many tissues across the body

**Fig. 3:**
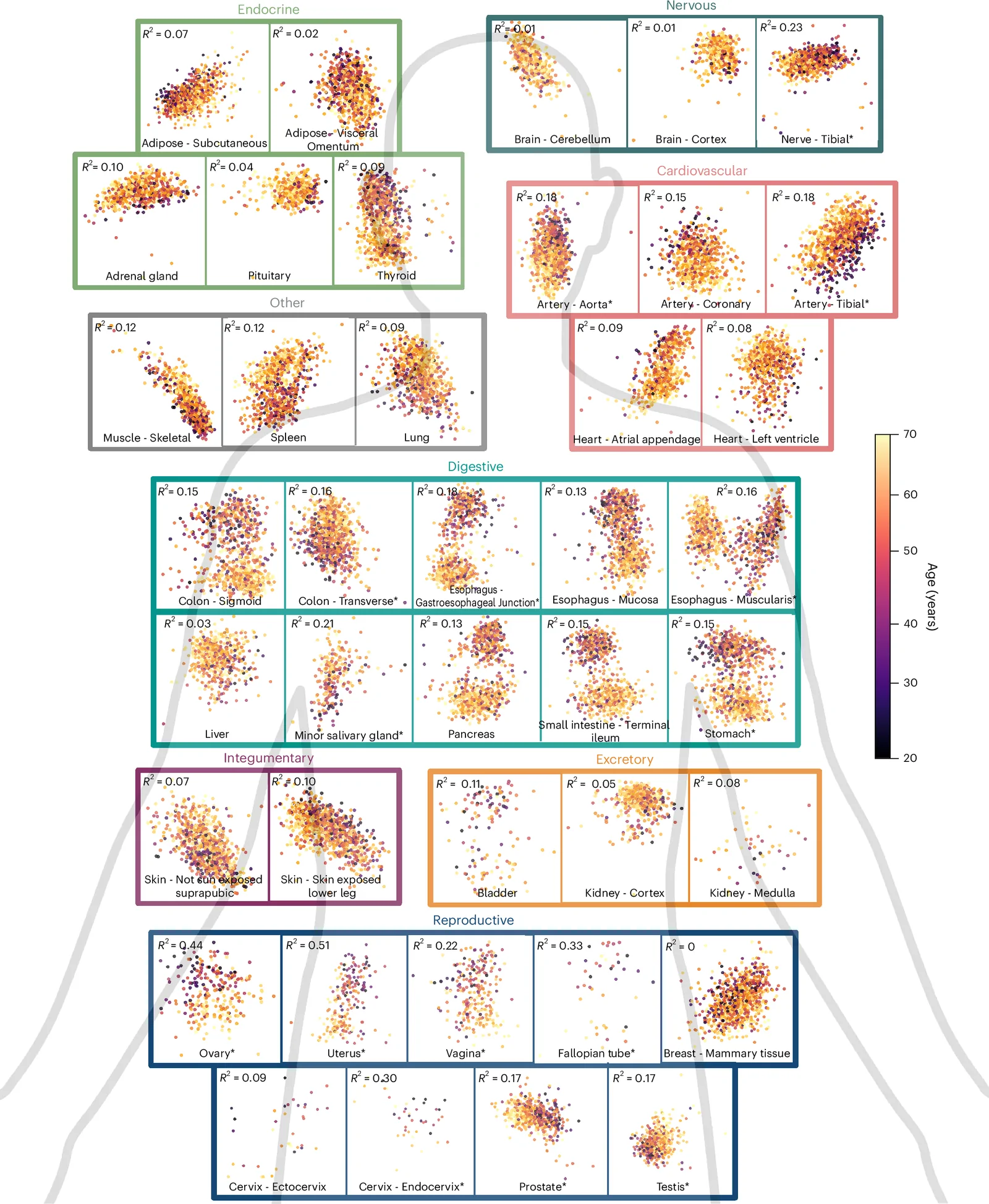
PC analysis of UNI-extracted slide-level morphological features for all 40 tissue types (*n* = 970 individuals, 25,306 biopsies). Each point represents one individual’s tissue sample; color indicates chronological age, with darker shades representing younger donors (ages 21–30) and lighter shades representing older donors (ages 60–70). Tissues are arranged by organ system, with each system outlined in a distinct color. Tissue names marked with an asterisk indicate tissues meeting both criteria for age-driven morphological separation: PC1 and PC2 jointly explain more than 15% of variance in chronological age by ordinary least-squares regression (*r*^2^ > 0.15, *F*-test; Supplementary Table 1). Significance of the *F*-test is indicated by asterisks adjacent to tissue names (*P < 0.05, **P < 0.005, ***P < 0.001). Full *r*^2^ and AUC values for all 40 tissues are reported in Supplementary Tables 1 and 2. No model was trained on chronological age at any stage; separation emerges solely from unsupervised morphological features. Figure created in BioRender; Alvarez, K. https://biorender.com/5o5bsgk (2026).

We next applied PathStAR across 40 human tissues with available H&E images (*N* = 970 individuals; *N* = 25,306 biopsies; Methods). Major PCs of morphology features (PC1–2) readily separated young from old samples in many tissues without any training on age (Fig. 3), indicating that aging, not individual variation or batch effects, explains the majority of the structural change and might be the driver. To quantify this separation, we measured both the age-related variance captured by PC1–2 (cross-validated ordinary least squares; Supplementary Table 1) and the ability to distinguish age groups with 10-year bins (Supplementary Note 6 and Supplementary Table 2). Furthermore, young tissues (20–29) were easiest to separate by age and the 50–59 group were hardest (one-versus-rest AUC up to 0.75; Supplementary Fig. 9 and Supplementary Table 2). We chose 15 tissues where age is the dominant driver of structural change (*r*^2^ > 0.15) as well as at least 200 total samples with samples distributed across the whole age span, for calculation of the structural aging trajectory.

#### Human tissues age through at least three distinct temporal programs

**Fig. 4:**
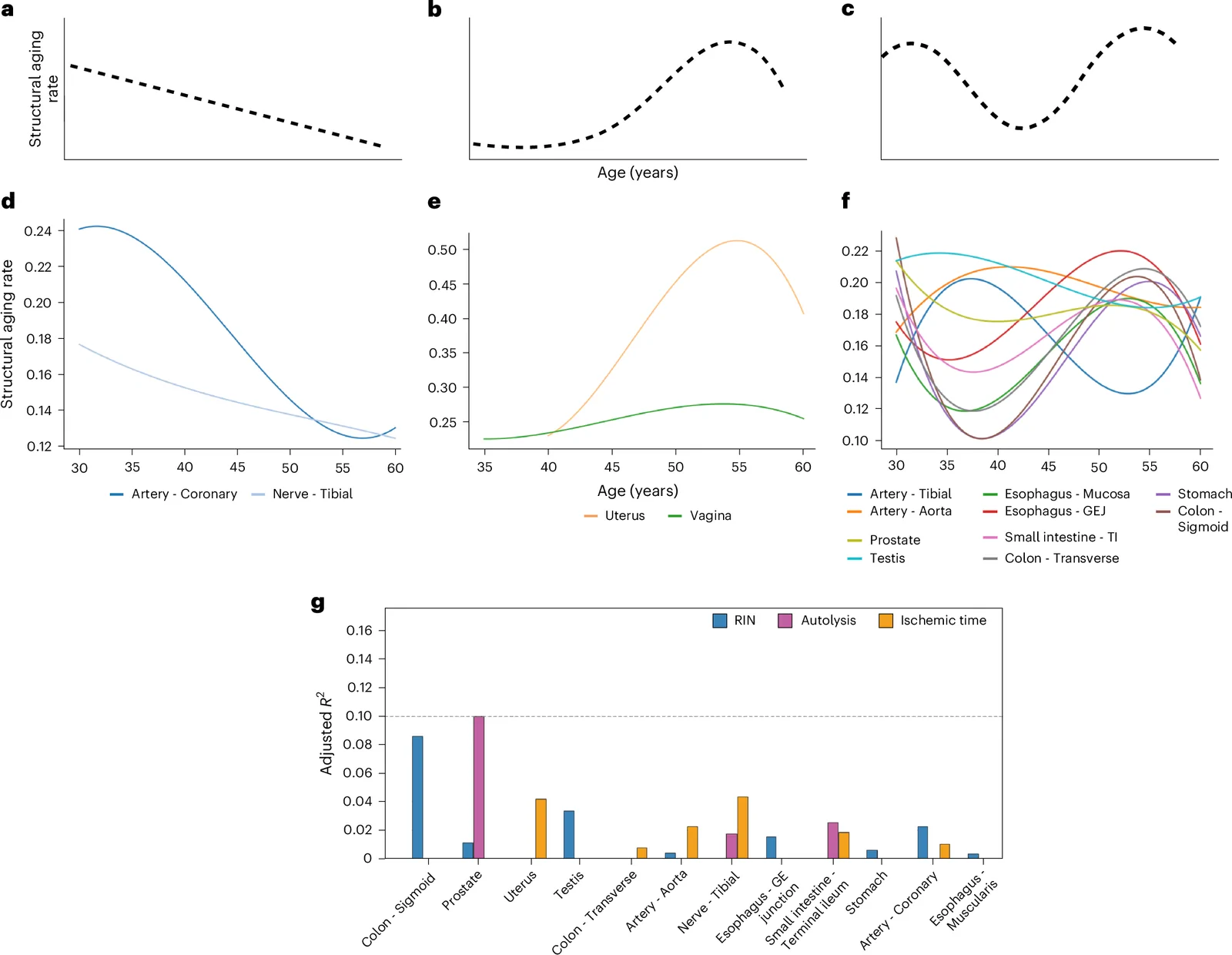
Overview of structural aging trajectories across 14 tissues. **a**–**c**, Three distinct aging patterns identified: early agers peak in the 30s, then decline (**a**); late agers remain stable until major changes in the 50s to 60s (**b**); and biphasic agers (the predominant pattern in 9/14 tissues) exhibit two acceleration periods around the early 30s and 50s (**c**). **d**–**f**, Tissue-specific examples showing vascular and neural tissues as early agers (**d**), female donor reproductive tissues as late agers (**e**) and digestive tract plus male donor reproductive organs as biphasic agers (**f**), revealing coordinated aging within functionally related systems. GEJ, gastroesophageal junction; TI, terminal ileum. **g**, Within-tissue variance in individual structural age (SA) explained by technical artifacts (RNA integrity number (RIN), autolysis and ischemic time); except prostate, other tissues show minimal artifact-driven variance. Figure created in BioRender; Alvarez, K. https://biorender.com/5o5bsgk (2026).

**Extended Data Fig. 3:**
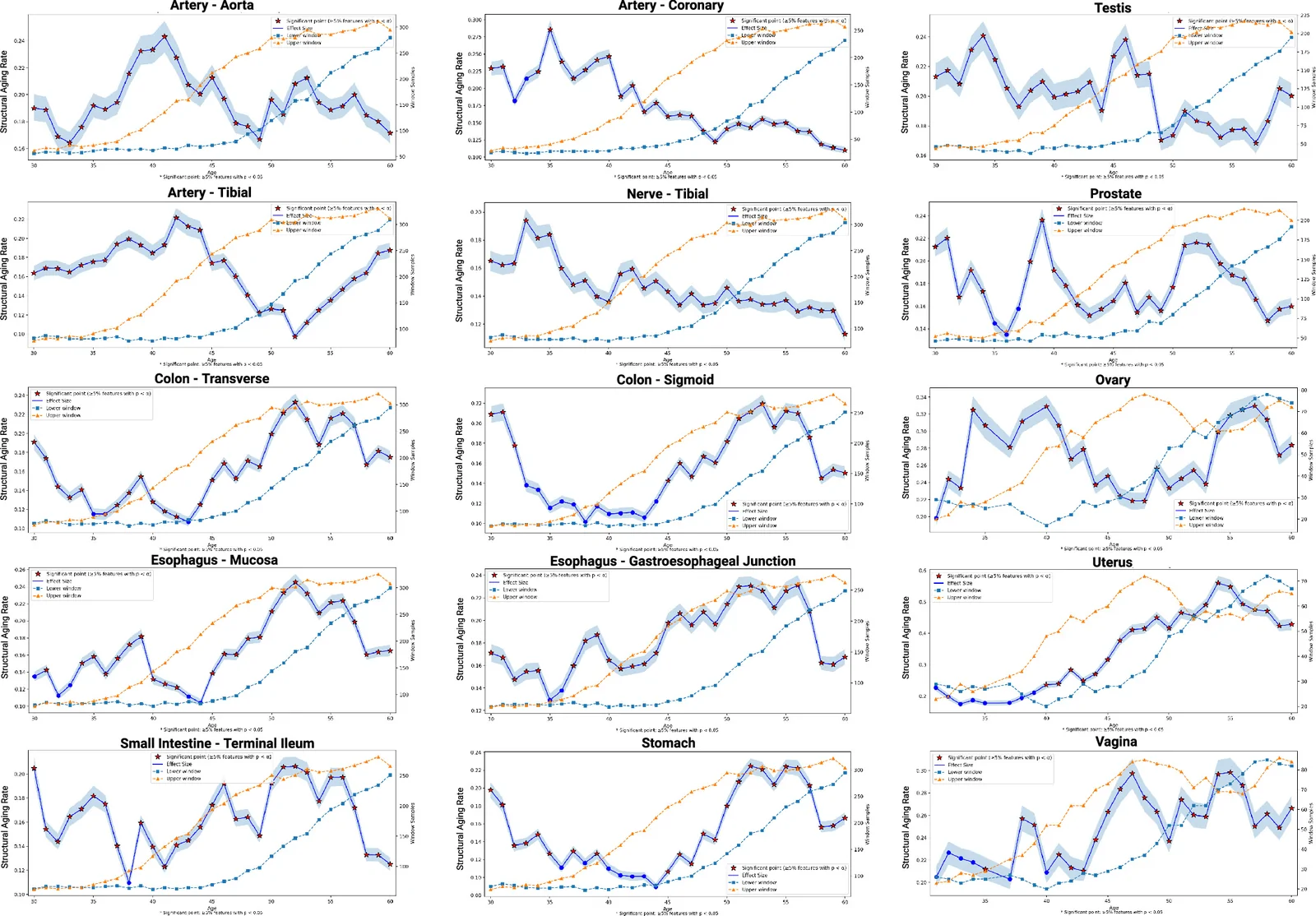
Raw Structural Aging Trajectory. Raw Structural Aging Trajectory (blue lines) are shown for the 15 tissues: Artery (Aorta, Coronary, Tibial), Colon (Sigmoid, Transverse), Esophagus (Gastroesophageal Junction, Muscularis), Nerve (Tibial), Ovary, Prostate, Small Intestine (Terminal Ileum), Stomach, Testis, Uterus, and Vagina. Blue lines show the mean magnitude of morphological change between adjacent age windows, and shaded ribbons indicate 95% bootstrap confidence intervals. Feature-level differences between adjacent age windows were tested using two-sided Welch’s t-tests. Red stars denote age transitions where ≥5% of UNI morphology features showed nominal significance at P < 0.05; P values were not adjusted for multiple comparisons. Dashed curves on the secondary axis show the number of samples contributing to the lower age window (squares) and upper age window (triangles) at each transition. Together, these trajectories highlight tissue-specific timing and magnitude of age-related structural change across male and female donor tissues. Figure created in BioRender; Alvarez, K. https://biorender.com/cczqv5r (2026).

We generated structural aging rate trajectories for each tissue, selecting only tissues that showed clear morphological differences between young and old samples, indicating detectable structural aging above individual variation. We next filtered and focused on tissue types with a high-confidence trajectory (Methods and Supplementary Notes 7). This yielded a total of 15 tissues comprising vascular, digestive and reproductive tissues (including ovaries), where we generated remodeling trajectories (Fig. 4a–c). For transparency, we provide all raw data plots (Extended Data Fig. 3 and Supplementary Fig. 17) showing individual data points, sample distributions and sex-stratified analyses.

Structural aging trajectories across these 15 tissues revealed tissue-specific patterns that could be clustered into three groups (Fig. 4a–c and Methods). Early-aging tissues exhibit high structural aging rates in the 30s, after which this rate declines, experiencing high structural changes during young adulthood (Fig. 4a). Late-aging tissues remain structurally stable through early adulthood, then undergo major structural changes in later decades (Fig. 4b). Biphasic-aging tissues display two distinct periods of ASA separated by stable phases, like the ovary pattern (Fig. 4c). Notably, some tissues exhibited markedly higher structural aging rates than others, indicating greater cumulative remodeling over time.

The vascular system, including tibial and coronary arteries, showed early structural aging, with structural aging highest in the 30s (Fig. 4d,f; see raw points, number of samples per age group and confidence intervals in Extended Data Fig. 3 and a sex-stratified plot in Supplementary Fig. 17). This trajectory is consistent with proteomic studies that also report early cardiovascular aging^36^. The trajectory remains consistent even after filtering out individuals with systemic diseases (Supplementary Fig. 18). Pathology notes support these findings: (1) individuals with higher delta-structural aging scores are more likely to have atherosclerosis (Supplementary Fig. 10); and (2) atherosclerotic changes, such as early plaque formation in arteries, showed their steepest increase during the 30s versus 20s, then plateaued in later decades (Supplementary Fig. 1, top left). Interestingly, tibial nerve also displays early structural aging, an unexpected finding warranting further investigation. Also, overall higher structural aging rates of coronary artery versus tibial nerve indicate higher cumulative remodeling during aging.

In contrast to the above, the uterus and vagina exhibited late structural aging pattern, with their highest structural aging rates occurring in early and middle 50s (Fig. 4e), aligning with menopause and post-menopause. This probably reflects major changes in the uterus including atrophy, endometrial thinning^37^ and myometrial structural changes following estrogen withdrawal^38^ during menopause and postmenopause^39^. The ASA periods of the vagina and uterus align with ASA-2 of the ovary, suggesting coordinated reproductive aging, potentially driven by shared hormonal regulation. We tested this coordination in aging at the individual level in the next section.

The predominant pattern observed in our dataset was biphasic structural aging, as described earlier for the ovary (Fig. 2f), characterized by two distinct peaks of structural aging rates, indicating two periods of ASA (Fig. 4c). While the timing and structural aging rates of two peaks differed among tissue types, this pattern was exhibited by 9 of the 14 tissues examined (aside from the ovary), including the digestive tract (esophagus, stomach, colon, small intestine and salivary gland), male donor reproductive organs (prostate and testis) and tibial artery (Fig. 4f). Overall, the two major periods of ASA for these tissues were in the 30s and around the 50s. Beyond sharing similar population-level trajectory, we explored whether digestive tract and male donor reproductive organs show coordinated structural aging at individual level in the next section. Testing the contribution of post-mortem artifacts toward these patterns, we find the total variance explained of structural aging rates by all post-mortem artifacts to be minimal in almost all tissues (<10%; Fig. 4g and Supplementary Tables 5 and 6).

Finally, the above trajectories do not emerge from molecular age clocks trained on chronological age, which assume to be linear by design, nor when applying the same trajectory analysis to bulk expression or methylation profiles (Supplementary Figs. 11, 20 and 21). Overall, this shows that structural aging of human tissues occurs in nonlinear phases, with distinct patterns across organ systems: vascular tissues age early (30s), female donor reproductive tissues coordinate with the ovary’s late peak (50s), and digestive and male donor reproductive organs follow a biphasic pattern. Trajectories and molecular profiles of 20 additional excluded tissues (≥200 cases each) are shown in Supplementary Figs. 19–21. Repeating the analysis using alternative pathology foundation models (UNI v2, CONCH v1.5^40^ and Virchow2^41^) reproduced overall similar structural patterns across most tissues (Supplementary Figs. 22–24 and Supplementary Note 2), indicating that the observed nonlinear remodeling dynamics are robust to encoder choice.

### Molecular programs during ASA

**Extended Data Fig. 4:**
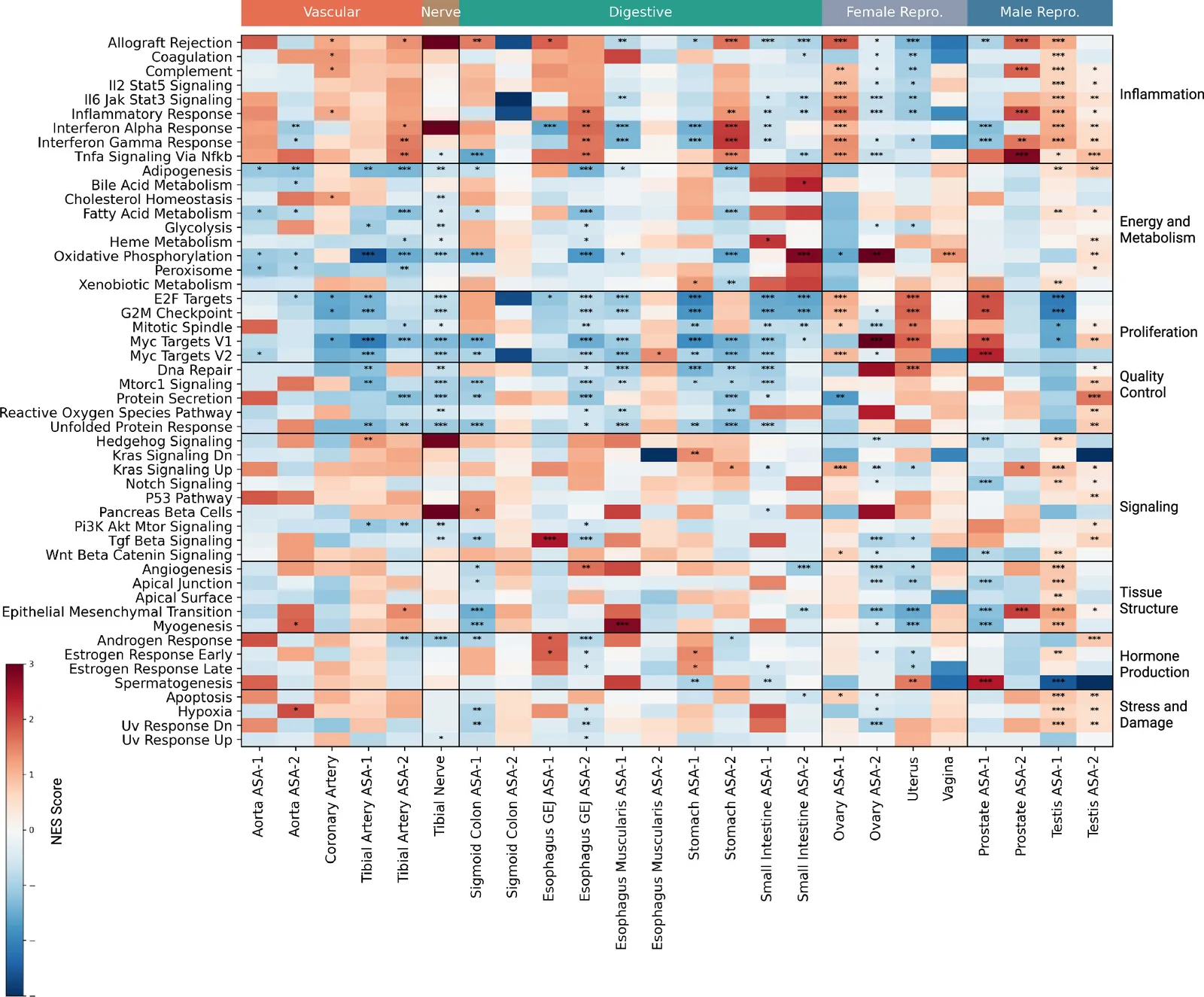
Hallmark Pathway Enrichment Across Accelerated Structural Aging (ASA) Transitions. Z-scored Normalized Enrichment Scores (NES) for Hallmark pathways are shown across tissue-specific Accelerated Structural Aging (ASA) transitions. Columns represent tissue–ASA contrasts (ASA-1 and ASA-2 where applicable), grouped by organ system (vascular, nerve, digestive, female reproductive, male reproductive). Rows are organized into eight biological categories: Inflammation, Energy & Metabolism, Proliferation, Quality Control, Signaling, Tissue Structure, Hormone & Reproductive, and Stress & Damage. NES values were z-scored within each tissue–ASA contrast to emphasize relative pathway shifts. Asterisks denote statistically significant enrichment. NES values were z-scored within each tissue–ASA contrast to emphasize relative pathway shifts. Pathway enrichment was assessed using pre-ranked GSEA with 10,000 permutations. Asterisks denote GSEA FDR q values: *q ≤ 0.10, **q ≤ 0.05 and ***q ≤ 0.01. Distinct and tissue-specific molecular programs accompany structural aging phases. Vascular tissues show coordinated inflammatory and metabolic remodeling, digestive tissues exhibit shifts in stress-response and quality-control pathways, and reproductive tissues demonstrate pronounced hormone and proliferation-related transitions. Figure created in BioRender; Alvarez, K. https://biorender.com/cczqv5r (2026).

We next identified molecular changes associated with tissue-specific ASA periods. ASA intervals were first defined from structural aging trajectories as peak inflection periods unique to each tissue. For each ASA interval (heuristically defined as a 5-year window based on peak width), we compared bulk transcriptomic profiles with those from the immediately preceding 5-year structurally stable window within the same tissue. Differential expression and pathway enrichment analyses were then performed for each ASA-stable transition across tissues (see complete pathway results in Supplementary Table 8; Extended Data Fig. 4).

#### A global inflammatory-metabolic program with organ-specific points of failure

**Fig. 5:**
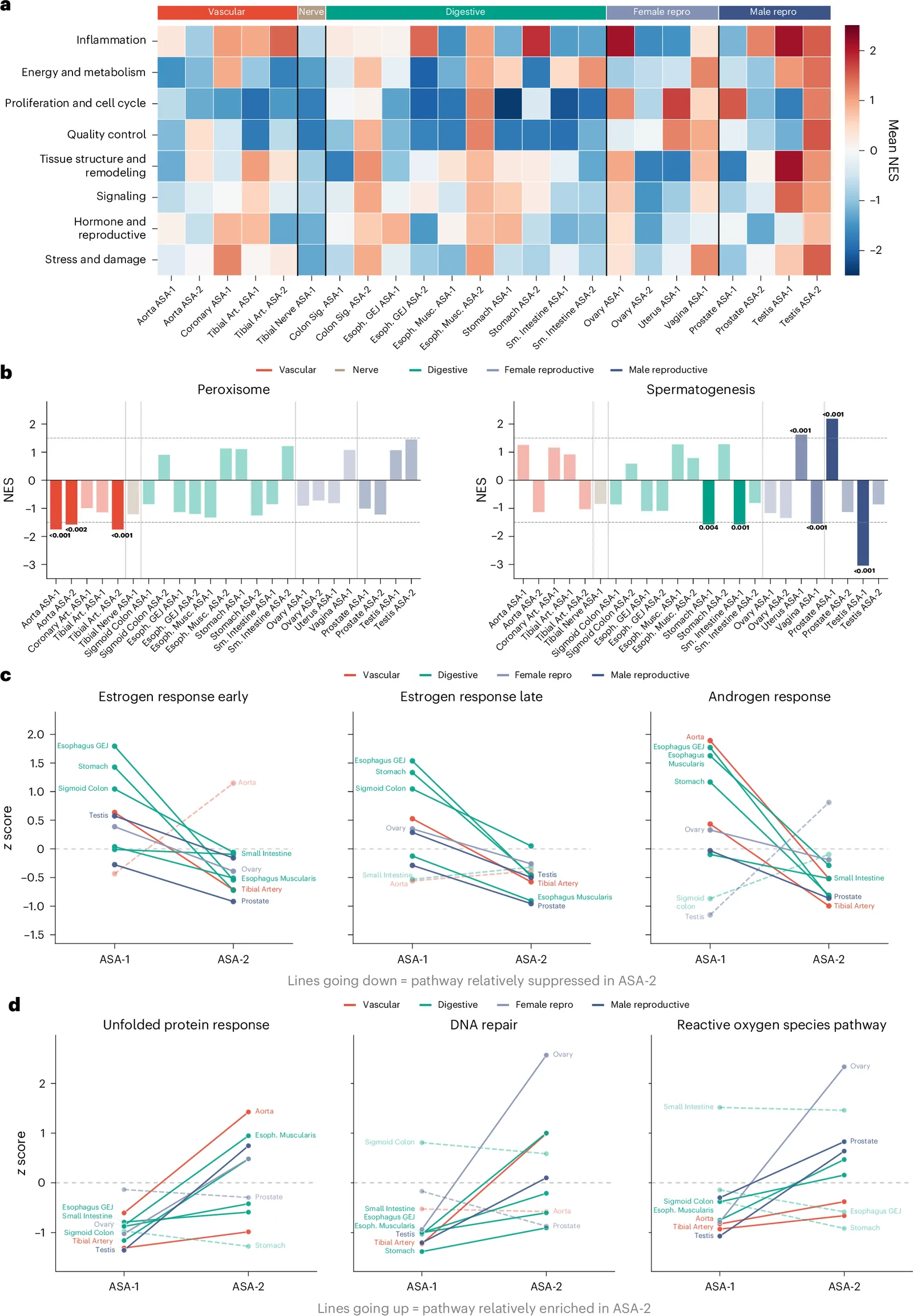
Molecular programs underlying ASA. **a**, Mean normalized enrichment score (NES) of the 50 MSigDB Hallmark pathways, grouped into 8 functional categories, across 24 tissue ASA combinations, organized by organ system. **b**, Tissue-level NES for peroxisome, significant exclusively in arteries, and spermatogenesis, strongest in testis (NES −3.04). *P* values are reported on top of the bar **c**, *Z*-scored NES from ASA-1 to ASA-2 for three hormone pathways, each declining in eight to nine of ten tissues. **d**, Opposing pattern for three damage-response pathways, each increasing in seven to eight of ten tissues. In **c** and **d**, solid lines indicate the dominant direction, and dashed lines indicate exceptions. Enrichment was assessed by two-sided GSEA; positive NES, upregulation; negative NES, downregulation. Exact NES and *P* values are provided in Supplementary Tables 8 and 11.

**Extended Data Fig. 5:**
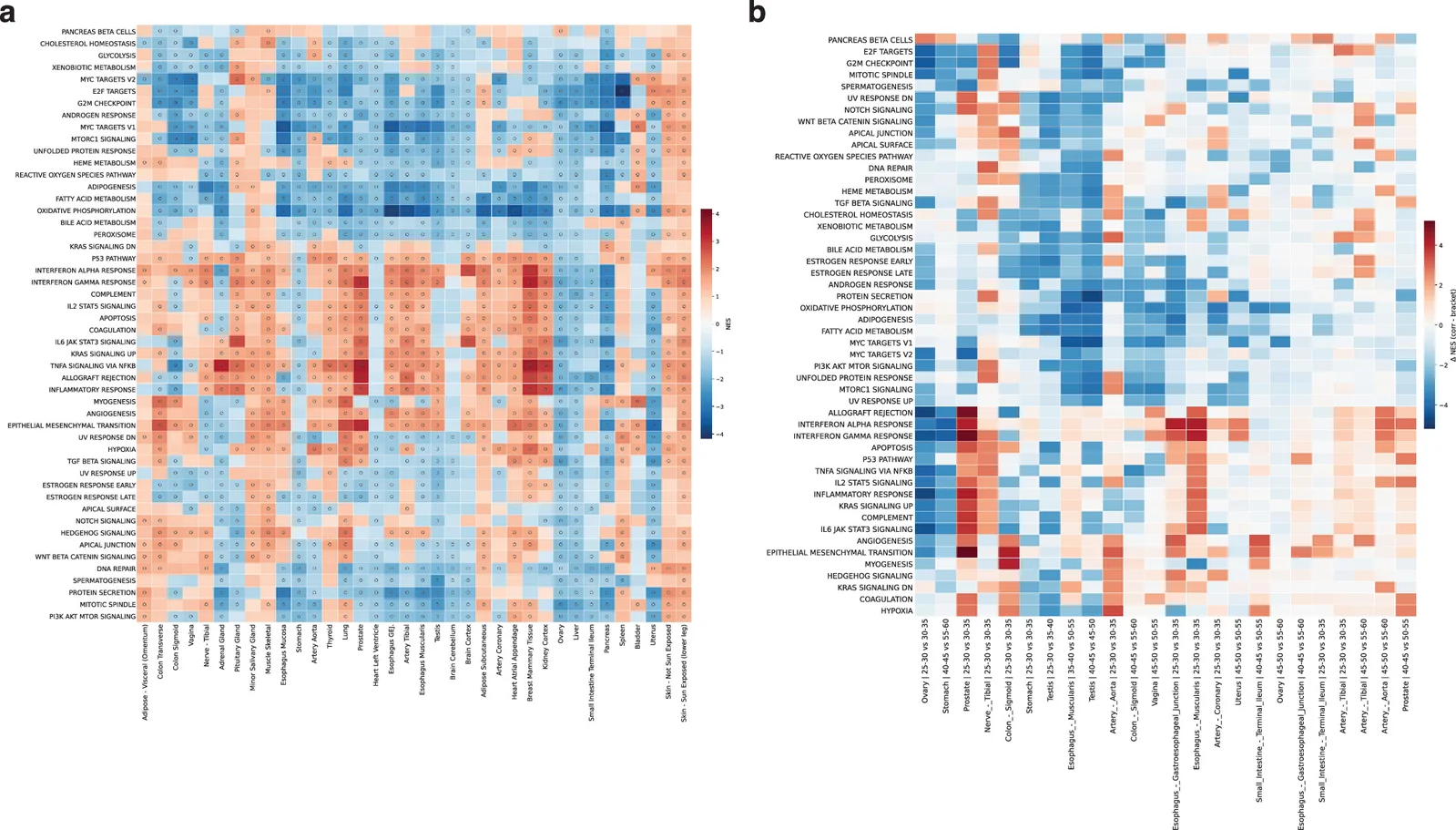
Age-Related Hallmark Pathway Analysis Reveals Tissue-Specific Aging Signatures. **a**, Gene Set Enrichment Analysis (GSEA) using Hallmark pathways performed on genes ranked by correlation with chronological age across tissues. Normalized Enrichment Scores (NES) reveal distinct tissue-specific patterns of pathway activation (red) and suppression (blue) during aging. Notable patterns include widespread upregulation of inflammatory pathways (TNF-α signaling, inflammatory response, IL6-JAK-STAT3 signaling) across multiple tissues, consistent with the inflammaging hypothesis. Metabolic pathways show tissue-specific divergence, with oxidative phosphorylation predominantly downregulated in metabolically active tissues (muscle, heart, liver) while showing mixed patterns in others. **b**, Comparative analysis showing the difference between NES scores from the Accelerated structural aging (ASA) period and the overall age-correlation analysis from panel a. This comparison highlights pathways that are specifically dysregulated during periods of accelerated structural aging versus those that change linearly with age. Figure created in BioRender; Alvarez, K. https://biorender.com/cczqv5r (2026).

Across tissues, ASA periods were defined by a common molecular signature (Fig. 5a): inflammatory pathways (TNF signaling, interferon alpha and gamma response, and complement) were broadly upregulated, while energy production (oxidative phosphorylation), proliferative capacity (E2F targets, G2M checkpoint and MYC targets) and cellular quality control (unfolded protein response, DNA repair and mTORC1 signaling) were simultaneously suppressed (Extended Data Fig. 4). This convergent program—inflammatory activation coinciding with the decline of regenerative, metabolic and quality control—was the dominant molecular feature of structural aging acceleration across the body. Finding pathways that simply linearly increase or decrease with age cannot fully capture this combination of activation and inactivation (Extended Data Fig. 5).

**Extended Data Fig. 6:**
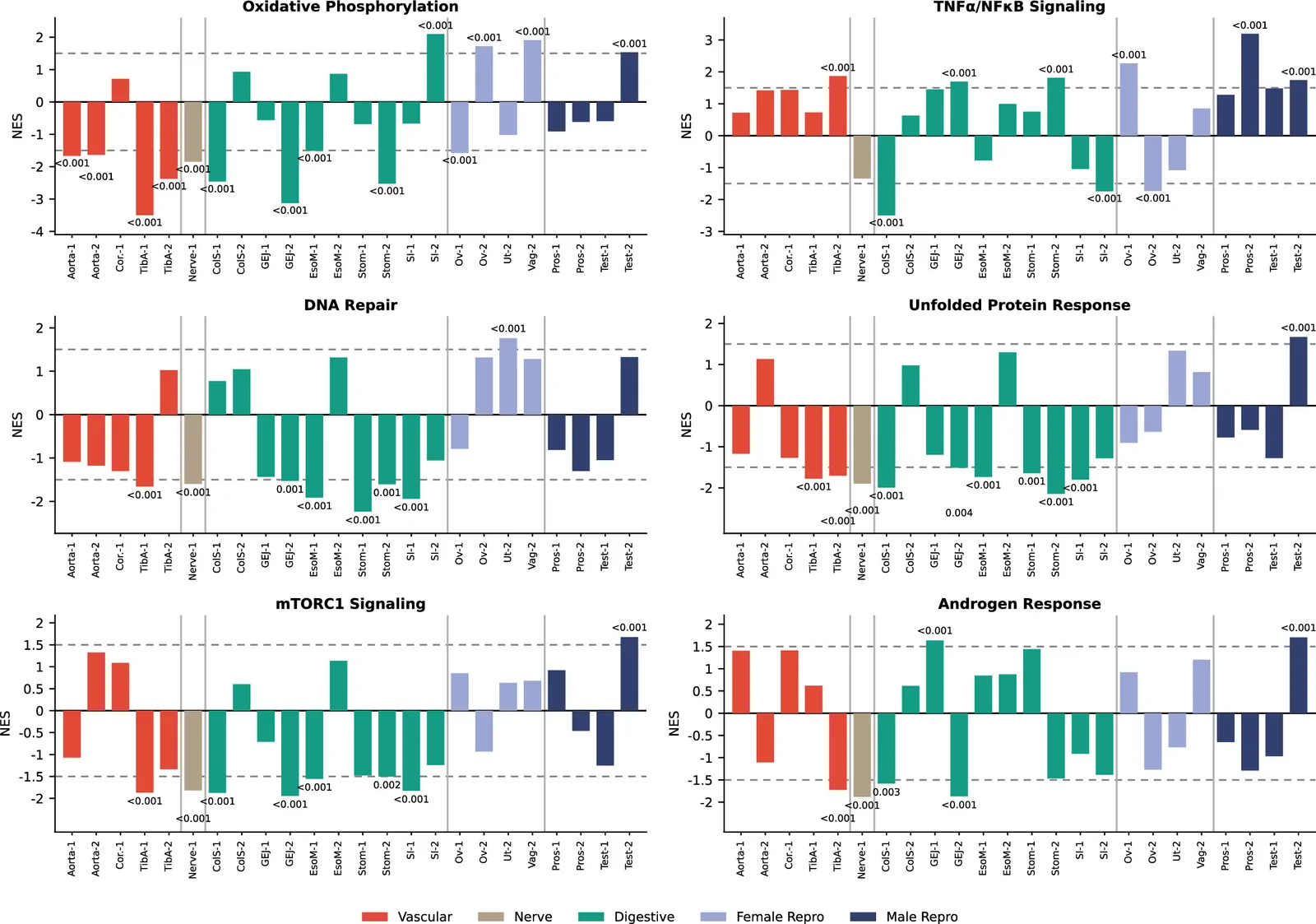
Key Pathway Changes Across Tissues During Accelerated Structural Aging (ASA). Normalized Enrichment Scores (NES) for representative Hallmark pathways are shown across tissues during ASA transitions. Pathways include Oxidative Phosphorylation, TNFα/NFκB Signaling, DNA Repair, Unfolded Protein Response, mTORC1 Signaling, and Androgen Response. Bars are grouped by organ system; asterisks indicate significant enrichment.

**Extended Data Fig. 7:**
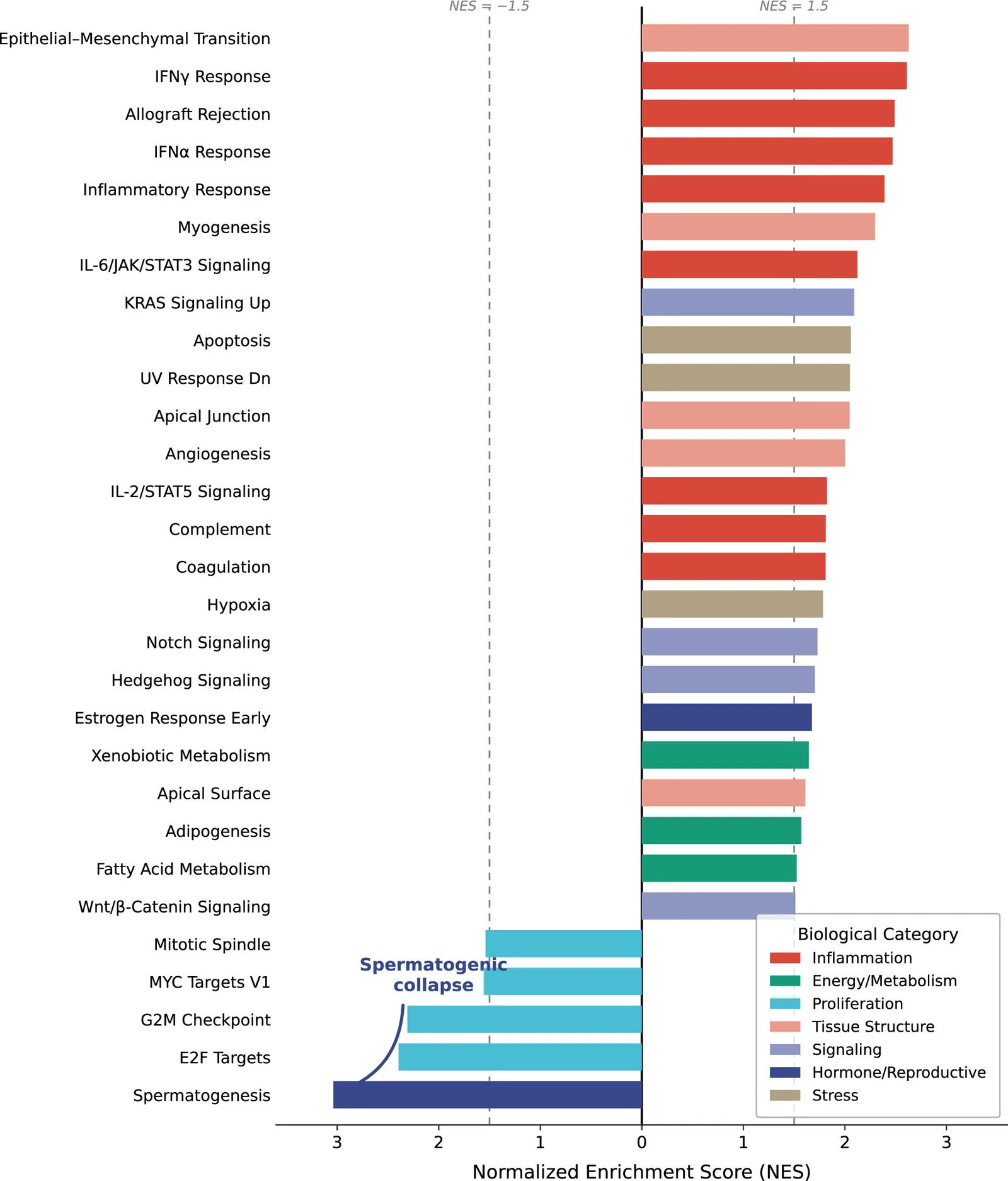
Testis ASA-1 - Significant Pathway Changes. Significantly enriched Hallmark pathways during ASA-1 in Testis are ranked by NES. Positive values indicate activation; negative values indicate suppression. ASA-1 is characterized by strong activation of inflammatory and stress pathways alongside marked suppression of spermatogenesis and cell-cycle programs (MYC, E2F, G2M). This pattern indicates a major early remodeling event in male reproductive tissue.

Within this shared program, individual organ systems harbored distinct molecular vulnerabilities (Fig. 5b and Extended Data Fig. 6). In vascular tissues, the peroxisome pathway activity, responsible for fatty acid oxidation, plasmalogen synthesis and reactive oxygen species detoxification, declined exclusively in the aorta and tibial artery and in no other tissue (Fig. 5b). This artery-specific loss of peroxisomal function may compound the early cardiovascular aging observed in their structural trajectories (Fig. 4a), as peroxisomal failure drives lipid accumulation and oxidative damage in the vessel’s wall. At the other extreme, testis exhibited the most dramatic molecular response of any tissue: upregulated inflammatory and immune pathways, while spermatogenesis was simultaneously suppressed to the strongest degree (Fig. 5b and Extended Data Fig. 7). This pattern is consistent with immune-mediated germ cell destruction following blood–testis barrier breakdown. This suggests an inflammatory catastrophe rather than a hormonal deficiency.

#### A molecular phase transition between ASAs: from hormone signaling to damage response

**Extended Data Fig. 8:**
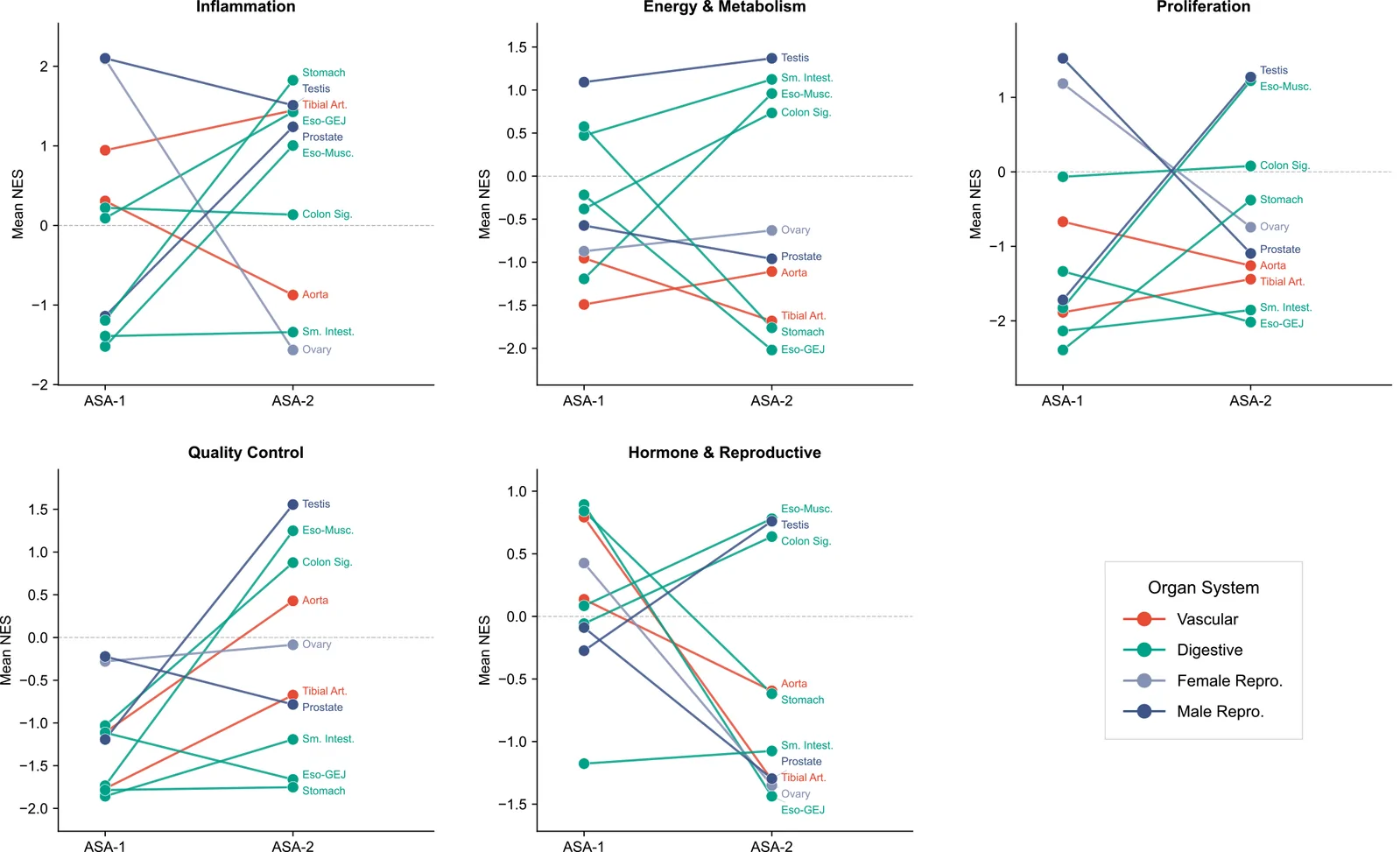
System-Level Pathway Shifts Between ASA-1 and ASA-2. Mean NES values for major biological categories (Inflammation, Energy & Metabolism, Proliferation, Quality Control, Hormone & Reproductive) are shown across two accelerated aging phases (ASA-1 and ASA-2). Lines connect the same tissue across phases; colors denote organ systems.

What distinguishes the first period of acceleration from the second? To answer this, we compared the relative pathway enrichment profiles between ASA-1 and ASA-2. This comparison revealed a consistent temporal transition across two classes of pathways. First, all three hormone-responsive pathways—estrogen response early, estrogen response late and androgen response—declined between ASA-1 and ASA-2 in nearly every tissue examined (9/10, 8/10 and 8/10 tissues, respectively; Fig. 5c), indicating a broad loss of hormonal signaling capacity as tissues enter their later period of structural aging. Notably, this decline was not driven by reproductive organs. The steepest drops occurred in gastrointestinal tissues, particularly the esophagus gastroesophageal junction and stomach, consistent with the known expression of estrogen receptors throughout the digestive epithelium and their role in mucosal barrier maintenance. Second, the damage-response pathways increased between ASA-1 and ASA-2 in the majority of tissues: the unfolded protein response (8/10 tissues), reflecting rising proteotoxic stress from misfolded protein accumulation; DNA repair (7/10), reflecting increasing genotoxic burden; and the reactive oxygen species pathway (7/10), reflecting escalating oxidative damage (Fig. 5d and Extended Data Fig. 8). Together, these indicate that the second period of acceleration is defined by high damage responses across tissues.

Overall, we provide molecular programs, either driving or characterizing, that are critical to the structural aging periods of organs, which will inform geroprotective therapies to protect function in an organ-defined and time-resolved (when and how long to treat) manner.

### Cross-organ coordination of structural aging within individuals

**Fig. 6:**
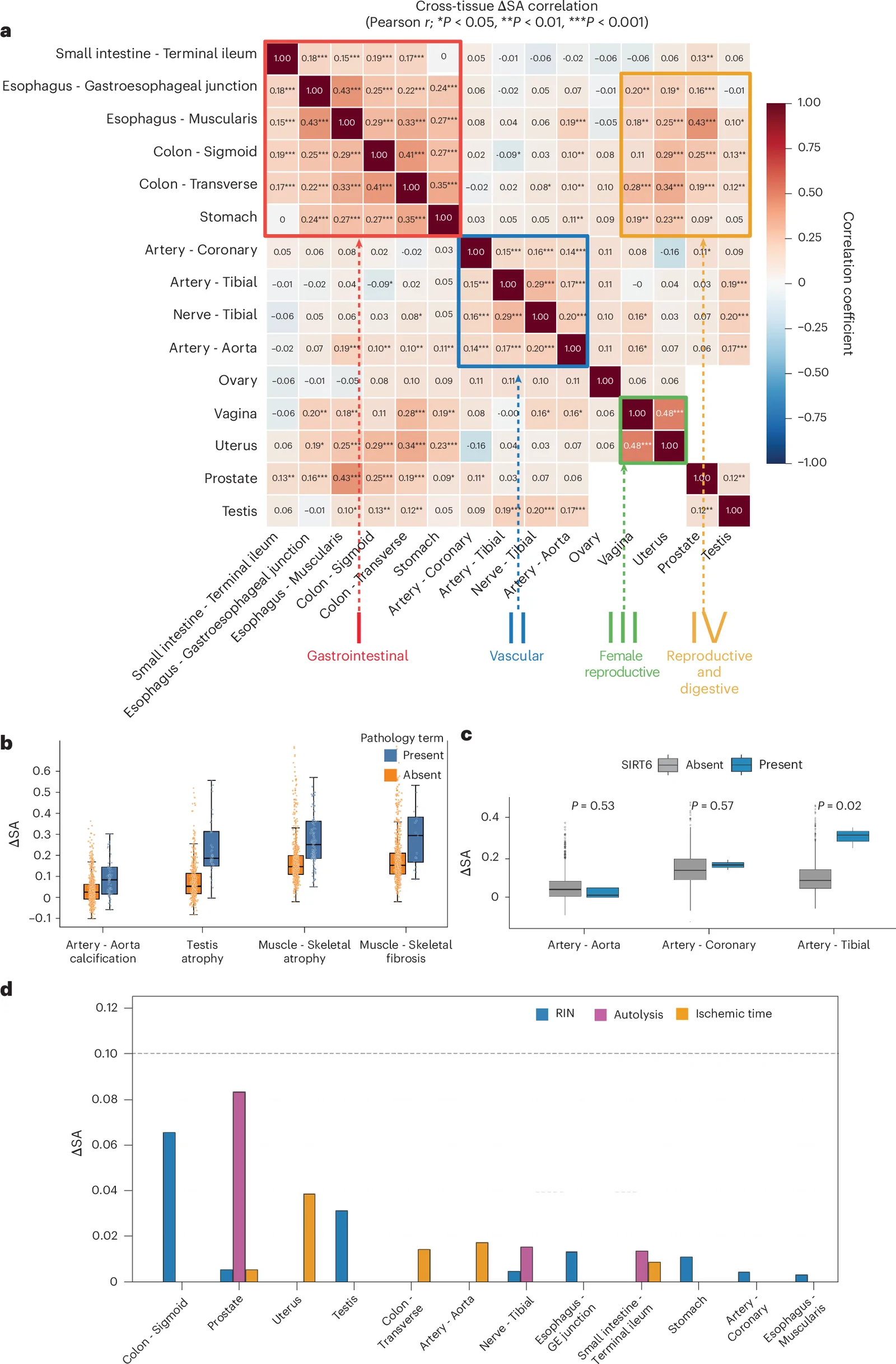
Cross-tissue delta-structural aging score correlations reveal coordinated structural aging within individuals. **a**, Heatmap showing Pearson correlation coefficients between individual delta-structural aging (SA) scores across 15 tissue types from the same individuals (*n* = 970). Delta-SA scores quantify each individual’s deviation from expected structural aging trajectories for each tissue. The color scale represents correlation strength: dark red indicates strong positive correlations (*r* ≥ 0.4), light red/orange indicates moderate positive correlations (*r* = 0.2–0.4), white indicates weak correlations (*r* < 0.2) and blue indicates negative correlations. Strong positive correlations reveal coordinated aging between tissue pairs, where individuals with ASA in one tissue consistently exhibit accelerated aging in the partner tissue, suggesting synchronized deterioration driven by shared mechanisms. The *P* values reported are before FDR correction. ‘I–IV’ represents an organ system grouped together. **b**, Box plots showing delta-SA scores in pathology-present and pathology-absent donor–tissue samples for aortic calcification in artery aorta (*n* = 53 present, *n* = 735 absent; *P* = 6.64 × 10^−5^), atrophy in testis (*n* = 38 present, *n* = 500 absent; *P* = 2.32 × 10^−13^), atrophy in skeletal muscle (*n* = 106 present, *n* = 788 absent; *P* = 2.97 × 10^−18^) and fibrosis in skeletal muscle (*n* = 17 present, *n* = 877 absent; *P* = 0.00161). Each pathology–tissue comparison was tested separately using a two-sided Wilcoxon rank-sum test. Box plots show median, 25th–75th percentiles and minimum–maximum whiskers; dots represent individual donor–tissue samples. **c**, Box plots showing delta-SA scores in SIRT6 variant-present and variant-absent donor–tissue samples across artery tissues: artery aorta (*n* = 3 present, *n* = 785 absent; *P* = 0.548), artery coronary (*n* = 2 present, *n* = 624 absent; *P* = 0.765) and artery tibial (*n* = 3 present, *n* = 874 absent; *P* = 0.0000256). *P* values were calculated using tissue-specific linear regression of the delta-SA score on the SIRT6 gene burden score. Box plots show the median, 25th–75th percentiles and minimum–maximum whiskers; dots represent individual donor–tissue samples. **d**, Within-tissue variance in ΔSA across age groups attributable to artifacts. The variance explained is uniformly low, suggesting that remodeling dynamics are not driven by technical confounders. Figure created in BioRender; Alvarez, K. https://biorender.com/5o5bsgk (2026).

**Extended Data Fig. 9:**
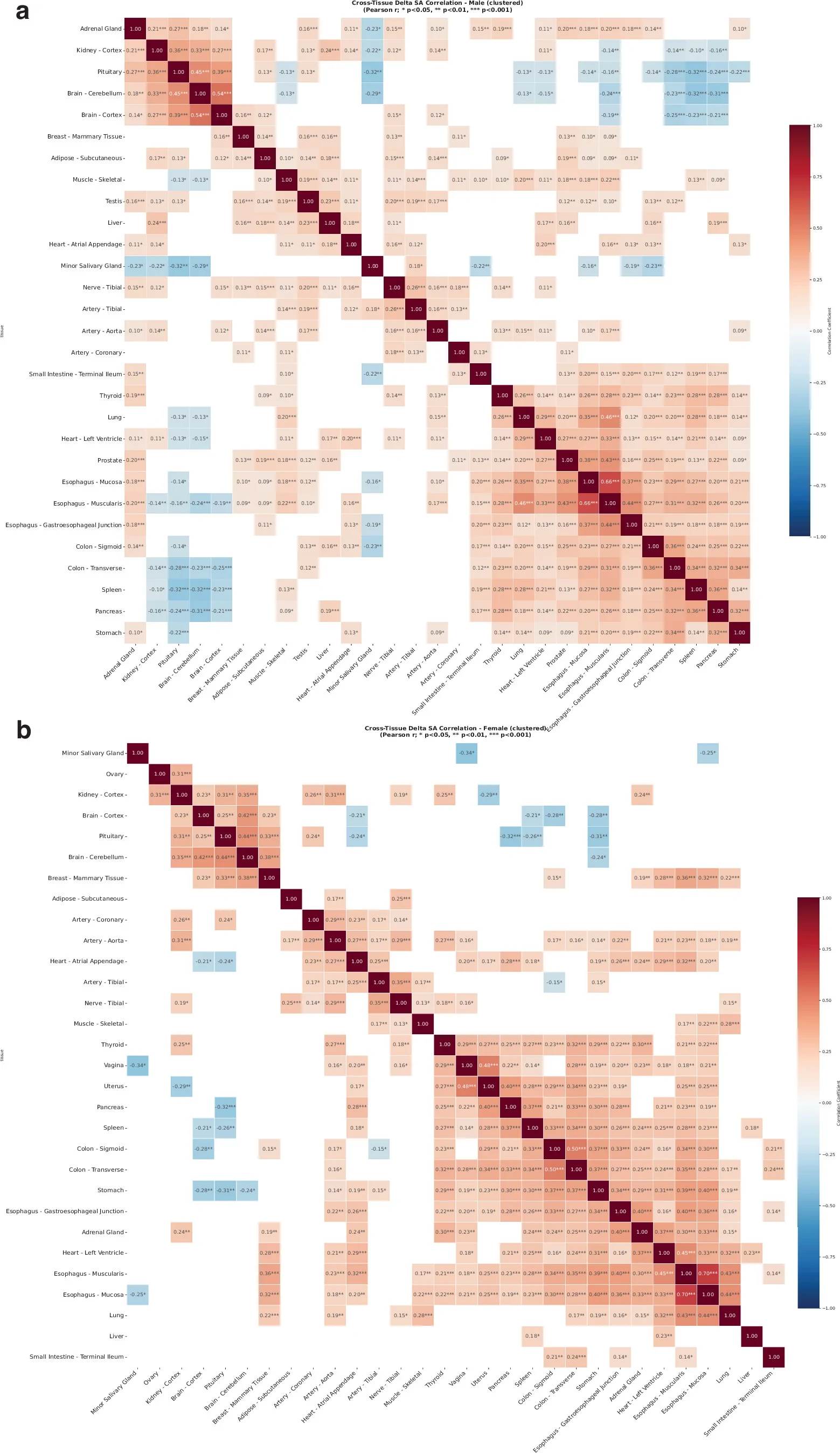
Sex-stratified cross-tissue correlations of ΔSA scores across all 40 tissue types. Heatmaps of Pearson correlation coefficients between individual ΔSA scores across all 40 tissue pairs, shown separately for males (**a**) and females (**b**), computed from the GTEx cohort (n = 970 individuals; 66.4% male). Each cell represents the Pearson r between ΔSA scores of a given tissue pair across individuals; only tissue pairs with at least 10 paired observations are shown (grey cells indicate insufficient observations for reliable inference). Color intensity reflects correlation strength, with dark red indicating strong positive correlations and blue indicating negative correlations. Asterisks denote uncorrected statistical significance thresholds (*P < 0.05, **P < 0.01, ***P < 0.001); note that P values are not FDR-corrected given the exploratory nature of this analysis across all tissue pairs. Positive correlations within canonical organ systems are prominent in both sexes, including within the gastrointestinal tract (colon sigmoid–colon transverse, esophagus gastroesophageal junction–esophagus muscularis, and stomach), the vascular system, and between nerve tibial and artery tibial (r = 0.28). In females, a strong uterus–vagina correlation (r = 0.47) reflects coordinated reproductive structural aging. These patterns extend the within-system coordination observed in the 15-tissue analysis (Fig. 6a) to the full 40-tissue atlas, revealing broad synchronized deterioration across functionally related organ systems. Figure created in BioRender; Alvarez, K. https://biorender.com/cczqv5r (2026).

We next asked whether individuals showing ASA in one tissue also exhibit accelerated aging in related tissues: coordinated deterioration within the same person. PathStAR computes individual level delta-structural aging scores (ΔSA; Supplementary Table 7) denoting an individual’s deviation from its population-level trajectory (Methods). To identify coordinated aging, we computed correlations among ΔSA scores of the 15 tissues with high-confidence trajectories (Fig. 6 and Methods). We first observed that tissues within the same organ system showed significant positive correlations, indicating that individuals who age faster in one tissue tend to age faster in related tissues: gastrointestinal (colon, esophagus and stomach; *r* = 0.20–0.43; Fig. 6a, Box I), female donor reproductive (uterus and vagina; *r* = 0.48, Box III) and vascular (tibial, coronary and aorta; *r* = 0.14–0.16, Box II), with additional brain (cerebellum and cortex: *r* = 0.55 male donors, 0.40 female donors) and cardiac coordination across all tissues (*n* > 200 samples) (Extended Data Fig. 9).

Beyond within-system coordination, we observed an unexpected cross-system coordinated deterioration axis between digestive and reproductive tissues. Individuals who showed ASA in the colon and esophagus also showed accelerated aging in prostate (*r* = 0.14–0.43; Fig. 6a, Box IV). Aligning with this, at the population level, these same tissues share biphasic structural aging trajectories (Fig. 4). Further corroborating this, at the molecular level, gastrointestinal tissues showed the highest enrichment of hormone-responsive pathways (estrogen and androgen response) during their first period of acceleration, even higher than reproductive tissues (Fig. 5a,c). Overall, this suggests that structural aging of digestive and reproductive tissues may be linked through a hormonal signaling axis whose decline drives synchronized structural deterioration across organ system boundaries. Additional cross-system axes (Extended Data Fig. 9) emerged that lacked an obvious shared molecular program but were nonetheless robust: neurovascular coordination (tibial nerve with multiple arteries, *r* = 0.23–0.28) and sex-specific neuroendocrine coordination (pituitary–brain correlations stronger in male donors than female donors). The mechanisms driving these axes remain to be determined.

### Clinical and genetic determinants of organ-specific structural aging

To determine whether ASA has identifiable clinical and genetic drivers, we tested associations between individual level ΔSA scores and clinical health records, lifestyle factors and germline variants. Clinically, accelerated structural aging showed strong positive associations with canonical aging pathologies (Fig. 6b and Supplementary Fig. 12), including atrophy (broad associations across tissues, particularly gastrointestinal and reproductive systems), fibrosis, vascular calcification and hyalinization. Systemic diseases, including autoimmune conditions (lupus) and neurological diseases (dementia), showed positive associations across multiple tissues (Supplementary Fig. 12), confirming that individuals with these conditions exhibit globally ASA. Lifestyle variables had insufficient sample sizes to reach significance (Supplementary Fig. 13).

**Extended Data Fig. 10:**
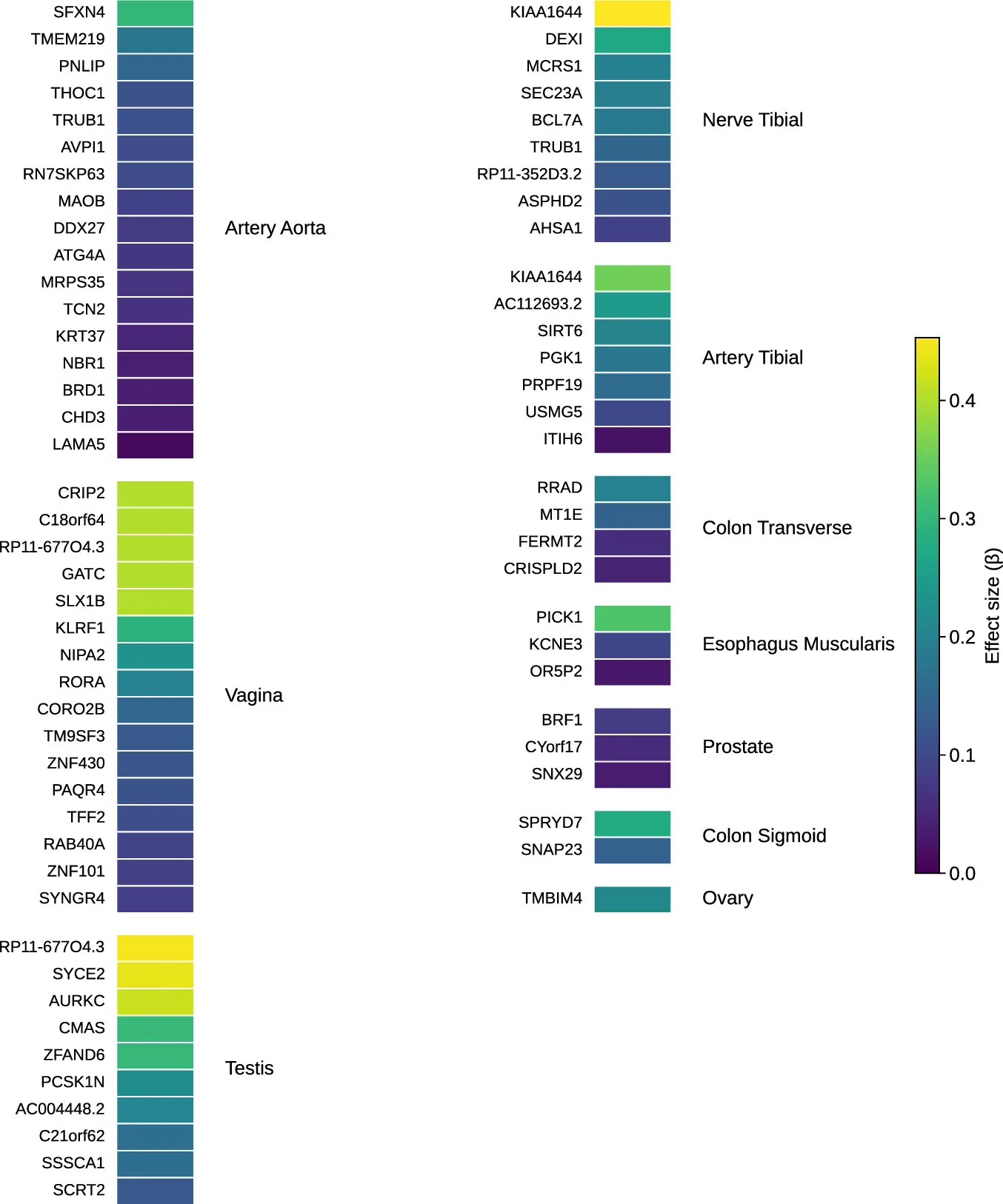
Gene-level associations between functional germline variants and structural aging across tissues. Per-tissue associations between gene-level germline variant burden and ΔSA (structural aging) scores, assessed by two-sided linear regression. Each row is one significant gene–tissue association; the 72 significant associations (69 unique genes, three significant in two tissues) are grouped by tissue and split across two columns. Significance was defined as Benjamini–Hochberg FDR < 0.05; all significant associations were positive, indicating that higher variant burden is associated with accelerated structural aging. Colour encodes the regression coefficient (β). ΔSA, change in structural aging score. Exact β, P and FDR values for all associations are provided in Supplementary Table 9.

We next performed a genome-wide association analysis between germline variants of 970 individuals and ΔSA scores, focusing on 127k functional variants filtered from 998k starting variants (Supplementary Methods). A total of 123 genes showed significant associations (false discovery rate (FDR) *P* < 0.1; Extended Data Fig. 10 and Supplementary Table 9). The pathway enrichment of these hits directly paralleled the molecular programs identified in the section ‘Molecular programs during ASA’: detrimental variants that accelerate structural aging were enriched in oxidative phosphorylation (Supplementary Fig. 14), aligning with the same energy production programs that declined universally during ASA periods (Fig. 5a). Conversely, protective variants were enriched in immune signaling pathways, particularly IL17 and interferon signaling, suggesting that genetic capacity to regulate the inflammatory response, the dominant upregulated program during structural aging acceleration, is protective against it.

The most striking individual association: *SIRT6* variants were specifically associated with accelerated vascular structural aging (Fig. 6c). *SIRT6* encodes a NAD^+^-dependent deacetylase and master regulator of DNA repair, telomere maintenance and metabolic homeostasis, and its knockout in mice produces early atherosclerosis^42^. This vascular specificity mirrors the molecular finding that arteries, and only arteries, showed exclusive decline in peroxisome pathway activity during ASA periods (Fig. 5b), a vulnerability that depends on the same NAD^+^-dependent metabolic processes that SIRT6 regulates. This links genetic variation in a known longevity regulator to organ-specific structural aging, demonstrating that aging pathways have tissue-selective rather than uniform whole-body effects.

## Discussion

Our results establish that the physical structure of human tissues deteriorates through organ-specific trajectory, with many cases of discrete and nonlinear phases, aligned with their known functional decline, rather than gradual decline—a pattern that is invisible to molecular clocks trained on chronological age. Three temporal programs emerged: early vascular aging (30s), late reproductive aging (around menopause) and biphasic aging in digestive and male donor reproductive tissues, indicating that organ systems follow distinct aging schedules. This echoes proteomic evidence of nonlinear molecular transitions^7,8^ but adds a key layer: because structure directly underlies function, these trajectories may track functional decline more directly than molecular markers. The ovary illustrates this: PathStAR captures both fertility decline and menopause from histology, while matched transcriptomic and methylation profiles do not. The framework thus maps what changes when in a tissue resolved manner: pathways active in the first acceleration (hormone signaling) versus the second (damage response), and organ-specific disruptions (peroxisomal metabolism in arteries, spermatogenesis in testis), identifying molecular targets and their timing, a prerequisite for tissue-appropriate geroprotective strategies.

We also find that structural aging is coordinated across organs within individuals, especially within expected organ systems (vascular and digestive). Importantly, this revealed an unexpected axis linking digestive and male donor reproductive tissues. Convergent molecular enrichment of hormone pathways in gastrointestinal tissues suggests shared hormonal regulation of both organ systems, providing a therapeutic opportunity.

We note that our total structural aging rate measure comprises degenerative processes, adaptive remodeling or neutral architectural variation. Thus, interpreting morphology features is critical. We did not do this as current pathology feature extractors cannot reliably interpret (Supplementary Note 4), and a method to resolve this will be a critical step forward. Furthermore, slide-level embeddings rely on mean pooling, a blunt aggregation that attention-based or hierarchical strategies could improve. Temporal resolution is limited by per-age-group sample sizes, requiring 10-year sliding windows that may obscure finer transitions. GTEx’s heterogeneity, which includes diverse clinical backgrounds, ventilator cases and varying post-mortem intervals, introduces confounders beyond chronological aging. Finally, not all age-associated changes imply functional decline; some may reflect adaptive remodeling or neutral variation.

In conclusion, because tissue structure is the basis of function, structural aging offers a more interpretable link to functional decline than molecular approaches alone. We believe this will lay the basis for assessing geroprotective intervention efficacy by quantifying whether treatments preserve architecture, link structural patterns to blood-based biomarkers for non-invasive monitoring, and reveal drug effects and toxicities that manifest as rapid structural change. By establishing structural aging as a quantifiable, predictable process, PathStAR opens a structural dimension for aging research.

## Methods

### Data acquisition

Whole-slide images were obtained from the GTEx public portal via v10 release (https://www.GTExportal.org/home/). All slides were digitized at 20× magnification (~0.5 μm per pixel) using an Aperio scanner (SVS format). Protected data, including participant demographic information (chronological age), lifestyle factors, clinical information and gene expression data, were accessed through dbGAP (request accession ID: 39103).

### Ethics statement

This study used de-identified post-mortem human tissue, genomic, transcriptomic, methylation and histopathology data from the GTEx project. No new human participants were recruited and no new biospecimens were collected for this study. GTEx tissue procurement and data generation were conducted under protocols approved by the relevant institutional review boards at participating collection sites, with appropriate consent or next-of-kin authorization for research use of donor biospecimens and data. Access to controlled GTEx data was obtained through authorized data-use approval. This research complied with all relevant ethical regulations. No participants were recruited or compensated as part of this study; all data derive from the existing GTEx resource, and donor enrollment and any related arrangements were governed by the GTEx project.

### Tissue selection and filtering

Although GTEx includes samples from 54 tissue types collected from nearly 1,000 adult post-mortem donors, we restricted our analysis to tissues selected using a systematic filtering strategy to ensure adequate statistical power, detectable age-related morphological variation, and robust estimation of structural aging trajectories.

First, we retained only tissues with available H&E-stained whole-slide images and a minimum of 200 samples per tissue, providing sufficient representation across age windows. Second, we required evidence of age-associated morphological change, assessed by correlation between UNI-extracted morphological features and chronological age, as well as age-dependent separation in PC analyses of the feature space. We built a simple linear regression model, and tissues with PC 1–2 showing variance *r*^2^ higher than 0.15 explained by age were picked. We also trained a model using linear discriminant analysis to separate young versus old, and the tissues with area under the curve (AUC) higher than 0.8 were picked.

Together, these criteria ensured that all selected tissues exhibited sufficient sample sizes, clear age-related structural remodeling and robust aging signals, enabling reliable construction of tissue-specific structural aging trajectories.

### PathStAR pipeline

Using above filtrated GTEx data, we developed PathStAR pipeline comprising the following three steps:

#### Preprocessing of WSI and feature extraction

We implement the vision encoder UNI for WSI feature extraction. UNI gives patch level embeddings that we then aggregate to create whole-slide representations. UNI is a large ViT, pretrained using the state-of-the-art self-supervised learning algorithm DINOv2^43^—a student–teacher knowledge distillation for ViT architecture. UNI was pretrained on the Mass-100K dataset, a dataset containing more than 100 million tissue patches across 20 major tissue types. To preprocess whole-slide images for UNI, we implemented Sobel edge detection to first identify regions of the slide containing tissue. Each 20× magnification WSI was then segmented into 512 × 512-pixel patches. Patches were excluded if more than 50% of their pixels exhibited low weighted gradient magnitude, as determined by Sobel. RGB color normalization was applied to remaining tiles using Macenko’s. Following image preprocessing, these patches are passed to UNI to extract 1,024-dimension patch-level features. We create a whole-slide representation by aggregating each 1,024-patch level feature together by taking the mean across the patches for each feature, giving us a final output of 1,024 dimensional vectors. Considering that our goal is to capture the overall structure of the tissue, we opted for mean pooling. Furthermore, a recent study^44^ showed that mean pooling performs comparably to more complex aggregation strategies for slide-level representations of whole-slide images.

#### Quantifying the structural aging rate

To identify the age-related structural aging rate in each tissue, we developed a sliding window analysis framework that quantifies effect sizes while stratifying by biological sex. For each tissue type with ≥200 samples, we implemented a comparative analysis between age-adjacent windows. For a given window size *w* (primarily using *w* = 10 years) and target age *t*, we compared the 1,024 slide-level features from UNI, in the lower window [*t* − *w*, *t* − *1*] with those in the upper window [*t*, *t* + *w* − 1]. This analysis was restricted to valid age ranges (*t* ∈ [min_age + *w*, max_age − *w*]) to ensure complete windows.

$${d}_{i}\left(t\right)=\frac{{\mu }_{i}^{\mathrm{upper}}\left(t\right)-{\mu }_{i}^{\mathrm{lower}}\left(t\right)}{{s}_{i,\mathrm{pooled}}\left(t\right)}$$

For each feature dimension *i* (1 ≤ *i* ≤ 1,024), we calculated the total change in the whole-slide morphology vector and the overall effect size as

$$\mathrm{Structural}\,\mathrm{aging}\,\mathrm{rate}=\mathop{\sum }\limits_{i=1}^{1024}{d}_{i}$$

Statistical significance was assessed using Welch’s *t*-test with significance threshold *α* = 0.05. We defined significant age transitions as those where ≥5% of features showed statistically significant changes after testing. For visualization and analysis, we computed the mean absolute effect size across all features for each target age.

For filtering structural aging rate data points with low statistical confidence, we considered only age points with sufficient samples (*n* ≥ 10) in both lower and upper windows, and only these were included in the analysis. We enforced a statistical robustness criterion, retaining only tissues in which at least 80% of estimated structural aging rates were statistically significant, defined as cases where more than 5% of morphological features (UNI) exhibited significant effect sizes during rate estimation (see section ‘Tissue selection and filtering’). Later, only the points where more than 5% UNI features showed a significant structural aging rate (*P* < 0.05) were included in the smoothening process.

All analyses were conducted together and then separately for samples from male and female donors to identify sex-specific structural aging patterns. This approach enabled identification of critical age thresholds where substantial morphological structural aging occurs across different tissue types and biological sexes.

To generate the structural aging trajectory, for spline smoothing, we used scipy’s Univariate Spline with a smoothing factor *s* = (window_size/*n*) × 0.5 × *n*, where window_size = 10 and *n* is the number of data points per tissue. This formulation makes *s* scale linearly with *n*, ensuring comparable smoothing behavior across tissues regardless of sample size. Crucially, rather than fixing the polynomial degree at the default cubic (*k* = 3), we allowed the spline degree to be determined data-adaptively by the smoothing parameters, meaning that the fitted curve is not constrained to a low-degree polynomial but instead balances fidelity to the data against smoothness. To further distinguish biological trends from interpolation artifacts, we complemented spline fits with Gaussian process smoothing using a radial basis function kernel, with length scale set to min(0.5, (window_size/*n*) × 2) and noise level = 0.1 × (window_size/5). Agreement between both methods was used as a criterion for selecting the best-fit trajectory per tissue.

#### Individual-level tissue score

A sliding window approach was implemented to quantify morphological deviations within tissue-specific contexts. For each target sample *i* with age ${\rm{ai}}$ ≥ 30 years, a reference group *R*_*i*_ was defined using the morphological structural aging $\mathrm{R}[{ai}-10,{ai}-1][{ai}-10,{ai}-1]$.

An effect size metric was defined to quantify the deviation in morphological structural aging rate magnitude ${\mathrm{ES}}_{j}$, and the effect size was computed as the absolute mean difference:

$${\mathrm{ES}}_{j}=\left|{x}_{i,j}-{\mu }_{{R}_{i},j}\right|$$

where ${x}_{i,j}$ represents the *j*th feature dimension of the target sample *i*, and ${\mu }_{{R}_{i},j}$ is the mean of the *j*th feature across the reference group *R*_*i*_. This effect size metric captures the absolute magnitude of morphological deviation without normalization by reference group variability, providing a direct measure of the morphological distance between target samples and their younger counterparts. The final effect size for each target sample was computed as the mean across all *p* feature dimensions:

$${\mathrm{ES}}_{i}=\frac{1}{p}\mathop{\sum }\limits_{j=1}^{p}\left|{x}_{i,j}-{\mu }_{{R}_{i},j}\right|$$

The effect size provides an interpretable measure of morphological changes, with larger values indicating greater deviation from the typical morphology of individuals within the same tissue.

#### Trajectory for molecular data

To enable direct comparison with structural aging, we applied the same sliding-window framework to molecular datasets. For tissues with ≥150 samples, we selected the top 20,000 and top 3,000 highly variable genes (or CpGs for methylation) based on variance across samples. Using a 10-year window, we computed mean absolute effect sizes between adjacent age bins, assessed statistical significance with Welch’s *t*-test (FDR-adjusted) and defined significant transitions as those with ≥5% of points significantly (*P* < 0.05) changing.

To reduce dimensional noise and capture dominant aging structure, we additionally computed trajectories using the top 50 PCs derived from the 20,000-feature set. Across tissues (Supplementary Figs. 24 and 25), PC analysis-based trajectories exhibited smoother and more consistent age-dependent patterns, indicating that low-dimensional representations better capture underlying molecular aging dynamics compared with raw feature-level analyses.

To further reduce high-frequency noise and emphasize dominant age-associated transitions, trajectories were smoothed using a uniform spline-based approach. This approach prioritizes detection of broad inflection periods over fine-grained year-to-year fluctuations. While localized peaks may reflect genuine biological variation, the current cohort sizes limit statistical power to robustly resolve such short-interval effects. Larger cohorts will be required to determine whether higher-frequency trajectory features are reproducible.

### Identifying pathways up- and downregulated during ASA periods

We developed a comparison framework to identify the key pathways up- and downregulated during major ASA periods. The key to this was utilizing paired transcriptomics and methylation data from the same biopsies available at GTEx Portal. We identified pathways up- and downregulated during ASA-1 by comparing the expression profile of samples from ASA-1 with the five age periods where the ASA graph shows an inflection point. Gene expression profiles were filtered to include only protein-coding genes based on established gene symbols. Expression values for each gene were extracted for both sample groups, and genes with missing data in either group were excluded from analysis. Statistical significance was assessed using Welch’s two-sample *t*-test, with log_2_ fold changes calculated as log_2_(mean_group2 + 1 × 10^−5^) − log_2_(mean_group1 + 1 × 10^−5^) to avoid division by zero. All genes from the differential expression analysis were ranked by log_2_ fold change in descending order and subjected to gene set enrichment analysis (GSEA) using GSEApy against the Hallmark pathways with 10,000 permutations. Pathways with FDR *q* value <0.1 and absolute normalized enrichment score (|NES|) >1.5 were considered significantly enriched.

The above analysis was repeated for methylation, also downloaded from GTEx Portal, separately for hypermethylated and hypomethylated states. GSEA was performed using the GSEApy package with preranked gene lists derived from differential expression results. Pathway enrichment was assessed against multiple curated gene set collections from the Molecular Signatures Database (MSigDB), including Kyoto Encyclopedia of Genes and Genomes (KEGG) pathways, Gene Ontology (GO) terms and the C3 collection containing transcription factor targets (TFT).

### Cross-tissue aging correlation analysis

To compute cross-tissue coordination in structural aging during aging, we computed correlations between ASA or delta-structural aging scores (denoted as ΔSA) using using Pearson’s correlation coefficients among organs implemented in SciPy. For each tissue pair, individuals with missing data in either tissue were excluded from the correlation calculation to maintain paired observations. Pairwise correlations were computed for ΔSA across all possible tissue combinations, generating both correlation coefficients and associated *P* values. A minimum threshold of ten paired observations was required for correlation calculations to ensure meaningful statistical inference. Statistical significance was assessed at three levels: *P* < 0.05, *P* < 0.01 and *P* < 0.001.

### Germline association with delta-structural aging

We processed the multisample GTEx whole-exome sequencing variant call file, which contains 979 individuals with variant genotypes and functional annotations. From this file, we extracted three layers of information: (1) the core variant descriptors (chromosome, position, reference and alternate alleles, quality and filter status); (2) annotation fields from the INFO column, including allele frequency, allele count, quality metrics and variant effect predictions-derived functional annotations such as predicted consequence, impact, gene symbol and clinical significance; and (3) individual-level genotypes for each sample, standardized into categorical calls (homozygous reference, heterozygous, homozygous alternate or missing). This parsing produced structured variant-level tables in which each row corresponded to a single variant, annotated with both functional attributes and per-sample genotypes.

For each tissue, all variants were grouped by gene symbol, and a gene burden score was calculated for every individual by summing the genotypes across all variants mapping to that gene. We also recorded gene-level metadata including the chromosome assignment and the number of variants contributing to each gene’s burden. Gene burden scores were then associated with patient-specific delta-structural aging scores using linear regression of the form

$${\rm{delta}}\,{\rm{SA}}\,{\rm{score}}={{\beta }}\times {\rm{gene}}\,{\rm{burden}}+{{\varepsilon }}$$

From each model, we extracted the slope (*β*), standard error, *P* value, coefficient of determination (*R*^2^) and correlation coefficient. The resulting tissue-specific association tables contained, for every gene, the number of contributing variants, number of samples analyzed and full regression statistics. Multiple-testing correction was applied using the Benjamini–Hochberg FDR procedure, and both complete and significant results were retained. No covariates were included; ΔSA was regressed on gene burden alone.

### Statistics and reproducibility

No statistical method was used to predetermine sample size. Sample sizes were determined by the number of GTEx donor–tissue samples with available histopathology images, age and metadata, and, where applicable, matched transcriptomic, methylation, genotype or pathology annotation data. Samples or tissues were excluded when required data were unavailable, failed quality-control criteria or did not meet predefined minimum sample requirements for the corresponding analysis. The experiments were not randomized, as this was an observational secondary analysis of existing human donor data. The investigators were not blinded to donor age or tissue identity during computational analysis and outcome assessment. Statistical tests, sample sizes, definitions of biological units and multiple-testing procedures are reported in the relevant Methods sections, figure legends and source data.

### Reporting summary

Further information on research design is available in the Nature Portfolio Reporting Summary linked to this article.

## Supplementary information

Supplementary Information Supplementary Notes S1–S10, Methods and Figs. 1–25.

Reporting Summary

Peer Review File

Supplementary Tables Supplementary Tables 1–10.

## Data Availability

The data used in this study were obtained from the GTEx project (accession: phs000424.v10.p2, project titled #39103). The H&E histological images and transcriptomic data are publicly available and were accessed through the GTEx Portal v10 release (https://GTExportal.org/home/). The germline variant data, phenotype and metadata, such as age and lifestyle factors, were accessed after appropriate institutional approval and data access agreement (general research use: phs000424.v10.p2.c1). The mean-pooled features generated from GTEx histology images using the UNI foundational model are available via Zenodo at https://doi.org/10.5281/zenodo.17042244 (ref. ^45^).

## Code availability

PathStAR is available as an open-source software package under the Apache License 2.0. The complete source code and documentation are available via GitHub at https://github.com/Sinha-CompBio-Lab/PathStAR.git.
