# Supplementary material for: Mapping structural aging across human tissues reveals tissue-specific trajectories and coordinated deterioration
Source: Nat Aging. 2026 Aug 31;6(9):2013–27. doi: 10.1038/s43587-026-01200-4 (PMC13577920; doi:10.1038/s43587-026-01200-4)
Supplement: Supplementary file 1 [file 43587_2026_1200_MOESM1_ESM.pdf]

nature aging

Supplementary information https://doi.org/10.1038/s43587-026-01200-4

Mapping structural aging across human tissues reveals tissue-specific trajectories and coordinated deterioration

In the format provided by the authors and unedited

Supplementary Notes, Methods and Figures

Supplementary Notes

Notes S1. Tissue specific quality control test: We tested whether GTEx histology collection is appropriate for studying tissue aging by testing whether a tissue captured canonical aging-related pathology (Supplementary Fig. 1). Thus, in GTEx biopsies, based on pathology tissues, with age: Artery showed increase in atherosclerosis, arthrosis & calcification. Colon and esophagus showed a decrease in mucosal layer thickness. Ovary showed a decrease in ova and follicles and an increase in corpora luteum till menopause age and throughout decrease of atrophy. Prostate showed increased stroma, where testis showed a sharp decrease in spermatogenesis. Uterus shows a decrease in both endometrium and myometrium, and an increase in atrophy, where vagina shows an increase in atrophy and epithelium layer.

Notes S2. Model Selection and Testing across Multiple Digital Pathology Foundation Models:

A. Why we chose UNI as our feature extractor? We initially selected UNI as the foundational model for PathStAR due to its large, diverse training corpus that also includes normal tissues (>100,000 H&E slides spanning ~20 tissue types from MGH, BWH, and GTEx), which provides broad and unbiased morphological representations. UNI is built on the DINOv2 framework and is optimized for histopathology-scale feature extraction.
B. Model Dependence: To evaluate model dependence, we repeated the full raw structural trajectory analysis (Supplementary Fig. 22–24) using UNI v2, CONCH v1.5, and Virchow 2 embeddings. Across tissues, UNI v2 and CONCH produced highly similar trajectory shapes to UNI v1, preserving major inflection points and accelerated structural aging phases. Virchow 2 also reproduced the primary trajectory patterns in most tissues, despite being trained predominantly on cancer-focused cohorts.

These results demonstrate that the non-linear structural aging trajectory identified by PathStAR are not model-specific artifacts but reflect stable morphological signals recoverable across independently trained pathology foundation models.

Notes S3. Modeling Ischemic time: Considering ischemic time, total time of tissue processing without oxygen, considerably impacts tissue damage, we reason that its correction is critical during PathStAR modeling. We thus tested whether the natural segregation of old vs. young samples in PCA based on morphology is due to ischemic time. To this end, we modelled age as a function of ischemic time, and then add PC1-2 to the model and test whether this addition explains significantly more variance. This was compared using a LRT test. Specifically, in case of PC1-2 addition, 36/40 tissues, addition of PC1-2 addition adds significantly more variance. Specifically, the largest improvements observed in reproductive tissues (uterus $\Delta R^2 = 0.40$, ovary $\Delta R^2 = 0.34$) and neural tissues (tibial nerve $\Delta R^2 = 0.16$). Six tissues (kidney medulla, cervix ectocervix, bladder, brain

cerebellum, thyroid, and breast mammary tissue) showed no significant improvement. For our trajectory analysis, we focused on tissues where PC1-2 adds significantly more variance, aside of three criteria in Notes S7.

Notes S4. Testing PLIP robustness to capture ovary-pathology: We evaluated PLIP's robustness to predict ovary-specific pathology using the following framework. We generated PLIP's embedding on 500 diverse terms which consisted of 3 categories: ovary-specific pathology terms, general pathological vocabulary, and common English words unrelated to ovary (Supplementary Fig 5, Supplementary Method). The ovary-specific pathology terms are: (Ovum, Ova, Fibrosis, Corpora Albicans, Corpora Albicantia, Corpus Luteum, Calcification, Menopause, and Multi Nucleated Giant Cells). While ovary-specific pathological terms exhibited higher mean similarity scores, the substantial overlap to terms unrelated to ovary suggests limited model specificity. Developing a specific model is out of scope of this study and instead we focused on pathology-terms PLIP can successfully capture, validated by pathologist reports. We identified three ovary-related pathologies that PLIP could reliably detect: Fibrosis, Ovum, and Atrophy (Supplementary Fig. 6A). We next observed that atrophy and fibrosis scores demonstrated a positive correlation with age (r = 0.38 & 0.28, respectively, Supplementary Fig. 6 B-C) and ovum scores exhibited a negative correlation with age (r = -0.341, Supplementary Fig 6-D). Comparing these three terms across our ASA period, we find out that fibrosis and atrophy show an increase in both ASA periods, whereas ovum shows an overall decline, especially in ASA 2 (Supplementary Fig. 6 E-G).

Notes S5. Modules of Morphological Changes for ovary: We identified age-informative feature modules in ovary tissue using a two-stage approach: first selecting the top 200 features most associated with chronological age via mutual information analysis, then clustering these features using K-means (K= 4-20) to identify co-regulated modules with similar age-related expression trajectories. This analysis revealed three distinct temporal patterns: features that monotonically increase with age, features that monotonically decrease with age, and features that remain relatively stable across the aging process. Higher K values refined these modules by partitioning them based on magnitude of change while preserving the fundamental tri-modal pattern. (Supplementary Fig 7)

Notes S6. Multiclass Age Bin Classification from Morphological Features: To comprehensively characterize the age-discriminative information encoded in PC1 and PC2 of UNI morphological features, we performed a multiclass classification across all six native GTEx decade bins (20-29 through 70-79), covering the complete sampled age range. Tissues with fewer than 100 total samples or fewer than 10 samples in any individual bin were excluded to ensure reliable estimation.

We trained a multiclass logistic regression classifier (*multi_class='ovr', class_weight='balanced'*) on PC1 and PC2 of UNI morphological features to predict 10-

year age bin membership. Performance was evaluated using stratified 5-fold cross-validation and reported as macro-averaged one-vs-rest AUC, the unweighted mean of per-class AUCs, where each age bin contributes equally regardless of sample size and a random classifier yields 0.5 per class.

Across all 40 tissues, macro-AUC ranged from 0.488 to 0.732 (Supplementary Fig. 9A), with ovary (0.732) and uterus (0.724) showing the strongest age-discriminative signal. The 15 tissues central to the main analysis showed a mean macro-AUC of 0.655. Per-class analysis (Supplementary Fig. 9B) revealed that the 20–29 bin was the most consistently separable across tissues (mean one-vs-rest AUC = 0.748), while the 50–59 bin was the hardest to distinguish (mean OVR AUC = 0.531; lowest-performing bin in 13 of 15 tissues), likely reflecting its transitional position between the two most populous GTEx age groups. Excluding the 50-59 bin raised the mean macro-AUC from 0.655 to 0.703 across the 15 selected tissues, confirming that the difficulty of this transitional decade is the primary driver of the overall performance ceiling when using only the top two morphological principal components.

Notes S7. Filtering criteria tissue: We applied the following filtering criteria to choose tissue for structural aging trajectory: First, we retained only tissue types with sufficient sample size, requiring a minimum of 200 available samples per tissue. Second, we selected tissues that demonstrated age-related morphological patterns by assessing correlation between UNI-extracted morphological features and chronological age, along with clear age-dependent separation in principal component analysis of the morphological feature space. Third, we applied a statistical robustness filter, retaining only tissues where ≥80% of the remodeling rates were statistically significant. Statistical significance was defined as having >5% of morphological features demonstrate significant effect sizes during remodeling rate calculations. This systematic filtering approach ensured adequate statistical power, demonstrable age-related morphological changes, and robust remodeling signals across all selected tissues for downstream analysis.

Note S8. Male Donor Samples vs Female Donor Samples: As shown in Extended Data Fig 1, Table S1, the distribution of male and female donors in our cohort is imbalanced, with male donors comprising approximately 70% of the dataset. This imbalance, combined with the multiple filtering steps in the PathStAR pipeline, poses challenges for modeling male and female donor tissue trajectories separately. As a result, the tissue remodeling trajectories presented in the main analysis were jointly modeled across sexes, a limitation of this study. For reference, however, Supplementary Fig 17 display the raw remodeling trajectories stratified by sex.

We observed marked differences in structural aging trajectories between male and female donors. Among the 15 tissues analyzed, five were sex-specific (prostate, testis, uterus, vagina, and ovary), leaving 10 shared tissues for direct comparison. Within the gastrointestinal tract, male donor samples exhibited a pronounced biphasic aging pattern with two distinct peaks in the colon (transverse and sigmoid), esophagus (muscularis and gastroesophageal junction), and stomach. By contrast, female donor samples displayed more gradual and moderate changes, without evidence of discrete biphasic peaks.

Distinct vascular patterns were also evident. In the aorta, male donor samples showed multiple peaks of structural change, whereas female donor samples displayed a sustained elevated rate between ages 30–50 followed by a gradual decline. In the coronary artery, both sexes exhibited an overall decline with age. However, in the tibial artery, male donor samples showed a stronger biphasic pattern, with structural aging decreasing until the early fifties and increasing thereafter, while female donor samples followed a comparatively attenuated trajectory.

In the tibial nerve, we observed a striking sex divergence: structural aging declined progressively in male donor samples, whereas female donor samples exhibited the opposite trajectory, with structural change accelerating beyond age 50. Importantly, these findings indicate that male and female donor tissues not only follow distinct patterns but also operate on different scales of structural aging, underscoring fundamental sex-specific differences in the temporal dynamics of tissue remodeling.

Note S9. Smoothing strategy for age trajectories: To model age-related structural trajectories, we evaluated four smoothing approaches: spline(k=3), Gaussian process regression, LOWESS, and Savitzky–Golay filtering. All methods captured the overall non-linear trends (Supplementary Fig 15); however, spline regression consistently provided the most stable fit across tissues, balancing smoothness with preservation of local transitions and avoiding boundary instability or overfitting. Based on these comparisons, spline smoothing was selected for all downstream analyses.

Note S10. Correlation between gene expression and structural aging rate: To test whether molecular trajectories directly mirror structural aging, we correlated age-resolved mean gene expression with the Structural Aging Rate across the lifespan for each tissue. Genes were ranked by Pearson correlation coefficient and subjected to pathway enrichment analysis (Hallmark and KEGG; Supplementary Fig 16). This lifespan-wide correlation approach did not yield biologically coherent or tissue-relevant pathway patterns, likely because opposing molecular programs across distinct structural phases are averaged into a single summary statistic. These results motivated the phase-specific ASA-guided molecular analyses presented in the main text.

Supplementary Methods

Cluster the ovary feature changes with age to find modules

To identify age-informative feature modules in ovary tissue, we employed a two-stage approach combining mutual information (MI) feature selection with K-means clustering. Mutual information between each feature and continuous chronological age was calculated using k-nearest neighbor density estimation, which directly handles continuous age values without discretization. Features with higher MI scores indicate stronger

association with age-related changes. The top 200 features with highest MI scores were selected and subsequently clustered using K-means clustering after z-score normalization to ensure equal weighting across features with different expression ranges. Clustering was performed across multiple K values (4, 5, 6, 9, 12, 15, and 20) with K-means initialization and fixed random seed (47) for reproducibility. Each resulting cluster represents co-regulated features exhibiting similar trajectories.

Mechanistic Interpretation of Morphological Features using vision language models

To interpret morphological features extracted by UNI, we employed two state-of-the-art vision-language models: PLIP. PLIP leverages contrastive learning on paired pathology images and diagnostic text to learn clinically relevant representations, while Conch, developed by the Mahmood laboratory, specializes in histopathological image understanding through self-supervised learning on large-scale tissue collections.

We established interpretability baselines by computing patch-level semantic similarity scores via dot product operations between patch embeddings and a curated vocabulary of 500 terms, comprising 10 ovary-specific descriptors, 240 general pathological terms, and 250 common English words. Slide-level similarity scores were derived by aggregating patch-level scores using the 95th percentile to capture the most relevant morphological patterns.

Three complementary analyses were performed: (1) validation against ground truth annotations from GTEx pathology reports to assess model accuracy, (2) correlation analysis between similarity scores and chronological age for ovary-specific features including follicular structures, stromal fibrosis, and tissue atrophy, and (3) comparative analysis of pathological features during the menopausal transition period. This multimodal approach enabled quantitative interpretation of morphological changes while maintaining clinical relevance and biological plausibility.

Disease and Lifestyle Association with Accelerated Structural Aging

We tested three categories of clinical features for their association with delta Structural Aging scores (ΔSA): (1) pathology terms extracted from histopathological reports, (2) Hardy Scale death classifications, and (3) binary disease history indicators from phenotypic metadata. For pathology terms, we applied text mining approaches to identify the presence or absence of specific pathological conditions within clinical notes and structured pathology categories for each sample.

Statistical associations were evaluated using multiple linear regression to control for ischemic time as a potential confounding variable. The regression model was specified as: ΔSA ~ clinical feature + ischemic time + intercept, where clinical feature represents the binary indicator (presence/absence) of each pathological condition, Hardy Scale comparison, or disease history. Missing ischemic time values were imputed as zero to maximize sample retention while maintaining analytical rigor. For each clinical feature, we extracted regression coefficients, p-values, and R^2 values to quantify the magnitude and significance of associations after controlling for ischemic time effects.

Hardy Scale comparisons were performed systematically across six predefined death circumstance pairs (e.g., violent vs. slow death, intermediate vs. slow death), while disease features compared individuals with and without specific medical histories from the top 20 most prevalent conditions. All pathology terms identified through text mining were tested comprehensively rather than limiting to high-frequency terms. To address multiple testing, we applied Bonferroni correction both within tissues and globally across all comparisons. Only associations with adequate sample sizes (≥10 samples per group) were included in the analysis to ensure statistical power for regression modeling.

Molecular Clock

Transcriptomic molecular clock

We trained tissue-specific transcriptomic clocks to predict chronological age from bulk RNA-seq data in GTEx. For each tissue, expression matrices (log-transformed TPMs) were processed after collapsing technical replicates to a single donor-level profile, retaining samples with ≥100 individuals. Within each training fold, genes were pre-filtered by correlation with age, and the top 3,000 most age-associated features (by absolute Pearson correlation) were retained. Features were standardized (z-scored) within the training set, and age prediction models were trained using an elastic net regression framework. Regularization hyperparameters (α across 18 log-spaced values and l1_ratio ∈ {0.1, 0.5, 0.9}) were tuned internally by five-fold cross-validation. To avoid information leakage, feature selection and scaling were repeated independently within each fold, and folds were defined by GroupKFold stratification over donor IDs to ensure that multiple samples from the same individual did not cross training–validation boundaries.

Performance was assessed in each fold using mean absolute error (MAE, in years) and coefficient of determination (R^2).

Methylation molecular clock

We trained tissue-specific clocks to predict chronological age from DNA methylation profiles. For each tissue with ≥100 samples, methylation matrices and aligned metadata (age, donor ID) were loaded, and feature selection was performed within each cross-validation fold to avoid leakage. In each fold, CpGs were ranked by their absolute Pearson correlation with age, and the top 2,000 sites were retained. Selected features were standardized using training statistics, and age was predicted using elastic net regression with hyperparameters tuned by internal cross-validation (α across 12 log-spaced values; l1_ratio ∈ {0.1, 0.9}). Donor IDs were used as grouping factors in five-fold cross-validation, ensuring samples from the same individual did not cross training–validation boundaries.

Performance was assessed in each fold using mean absolute error (MAE, in years) and coefficient of determination (R^2).

Supplementary Figures

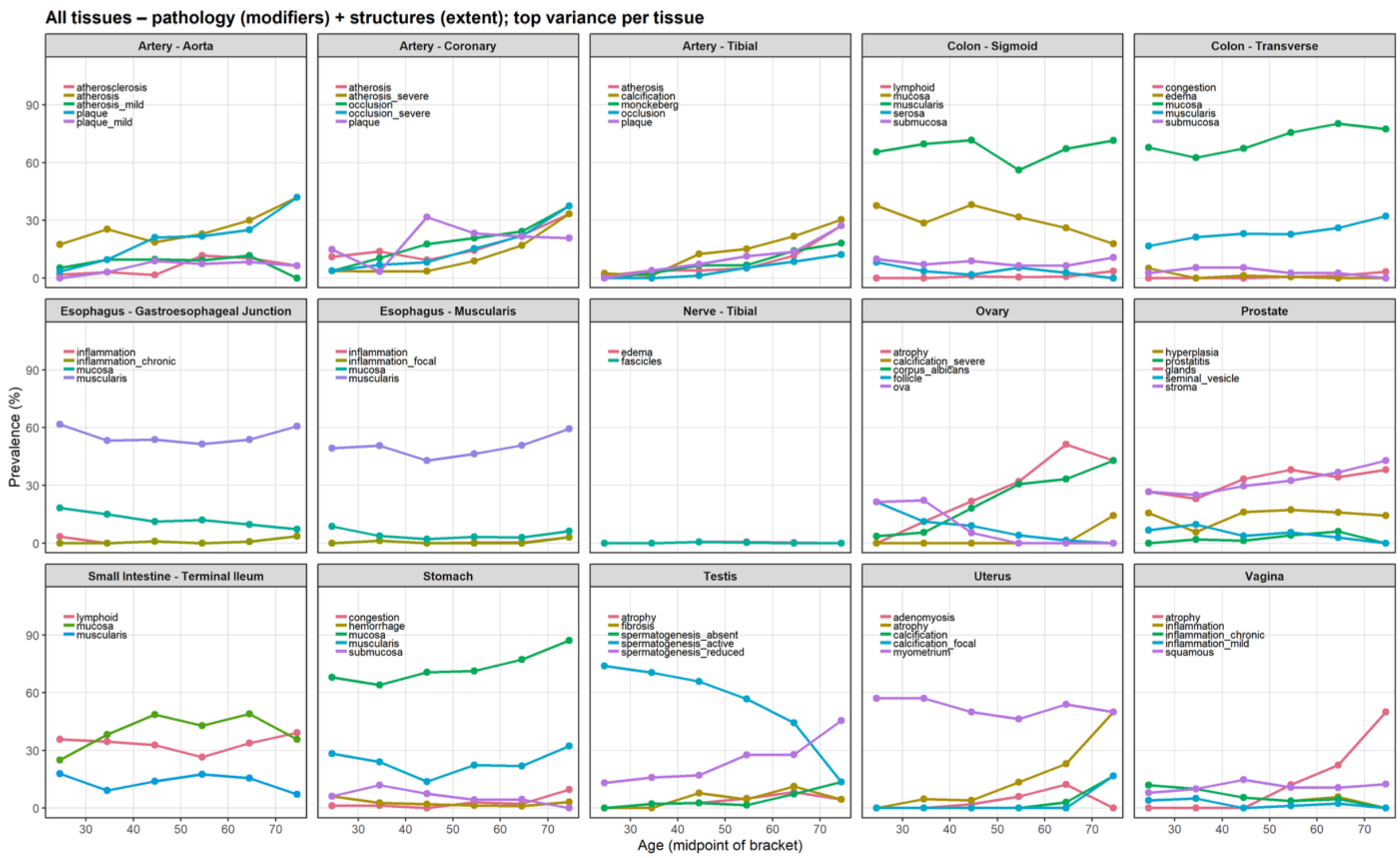

Supplementary Fig. 1: Age-related changes in the frequency of pathologies across 15 different tissue types. Each panel shows the prevalence of cases with specific pathological conditions plotted against age groups from 21 years to 70+ years.

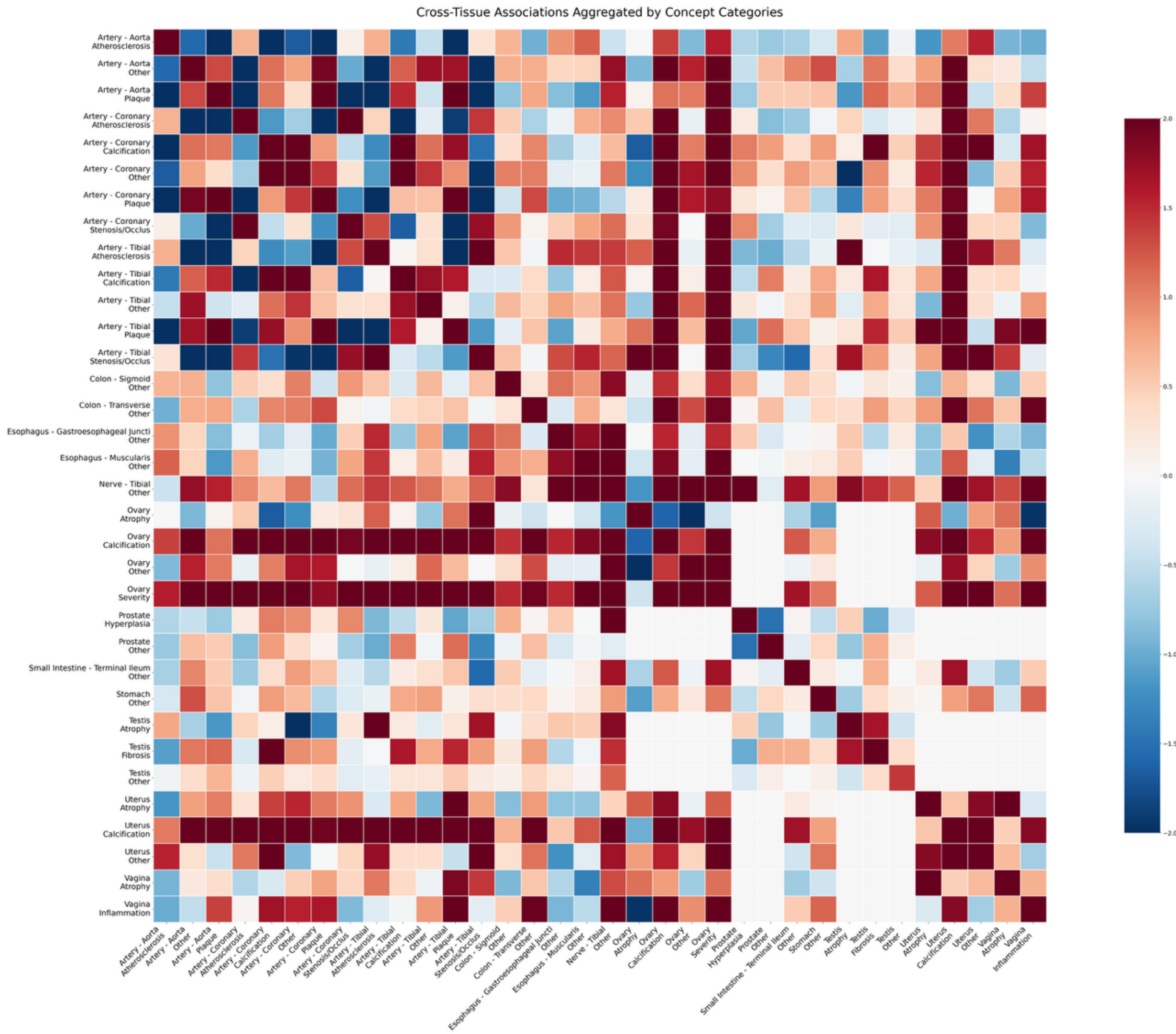

Supplementary Fig. 2: **Cross Tissue Pathology Term Co-occurrence Heatmap.** This heatmap illustrates the strength of association between pairs of pathological findings from the Genotype-Tissue Expression (GTEx) project. To simplify the analysis, numerous specific pathology terms were grouped into the concept categories shown on the x and y-axes. Each cell's color represents the mean Log2 odds ratio, with dark red indicating a strong positive association (i.e., the two conditions tend to occur together) and dark blue indicating a strong negative association.

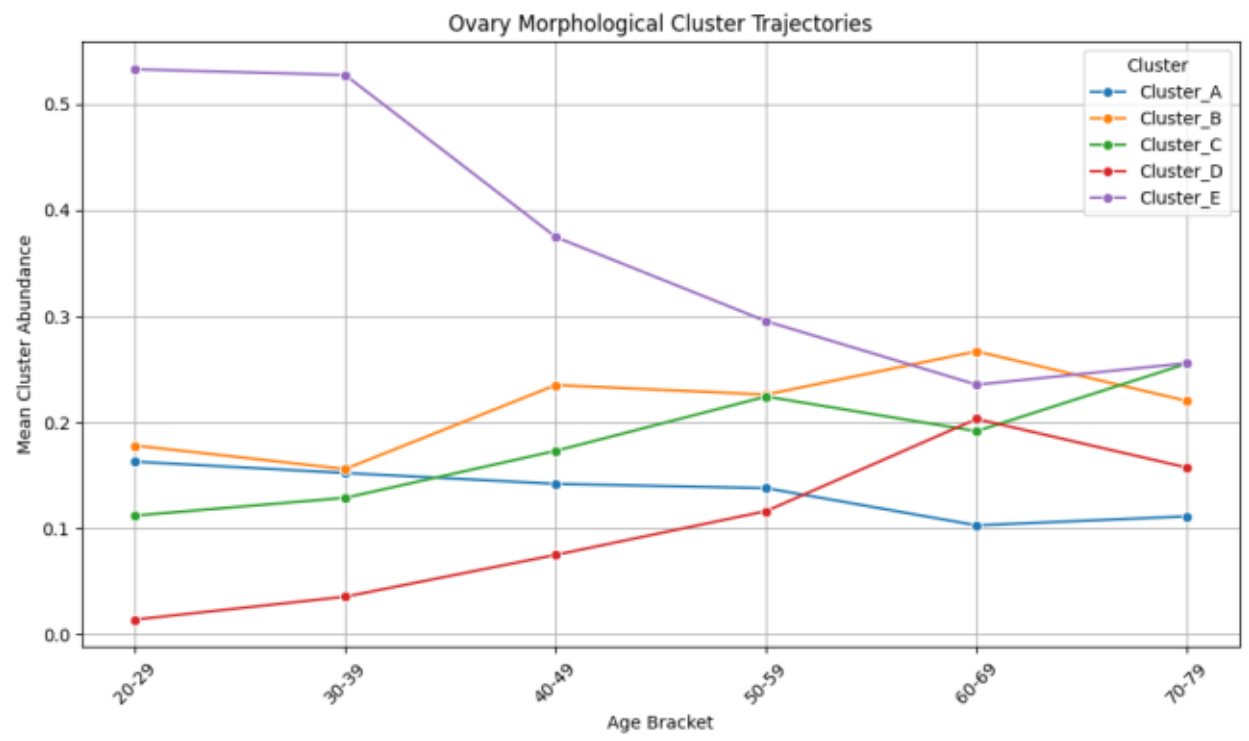

Supplementary Fig. 3: Ovary morphological organization trend across age. Patch embeddings were globally clustered with k-means (k=5) to assign fixed morphologic labels A–E. For each slide we computed cluster abundance (fraction of patches per cluster) and averaged abundances within 10-year age brackets. We find that cluster **E** dominates in the 20s (~0.53) but steadily declines to ~0.24-0.26 by late life. Cluster **D** rises from near zero in the 20s to ~0.20 in the 60s, with a slight taper in the 70s. Clusters **B** and **C** increase through mid-life (B peaks ~0.27 at 60–69, then dips; C continues to ~0.26 by 70–79), while **A** gradually decreases (~0.16 → ~0.10–0.11). These abundance shifts are consistent with co-occurrence results showing loss of E self-coherence and growth of D-centered mixing.

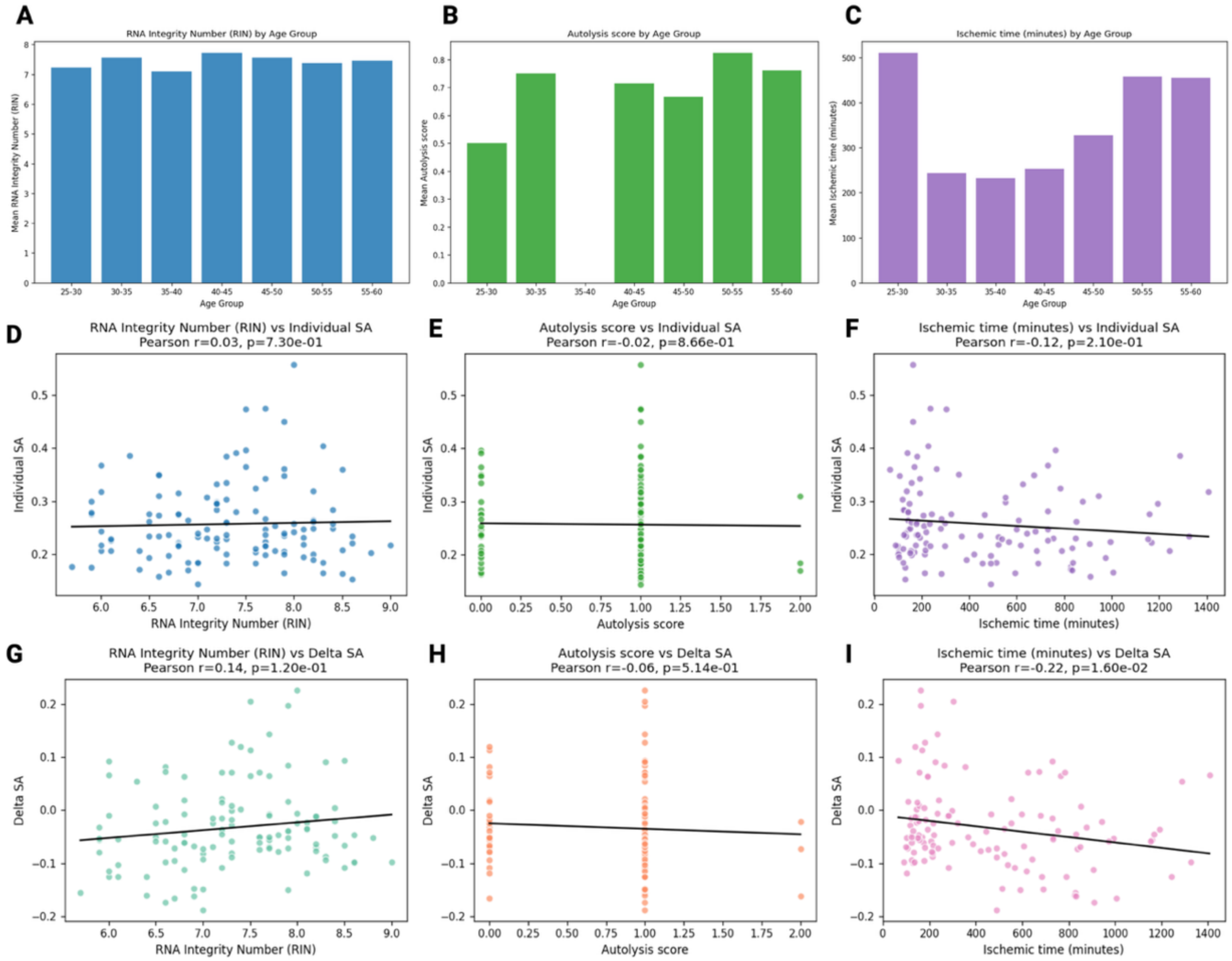

Supplementary Fig. 4: Quality metrics and covariates across GTEx samples in Ovary. (A–C) RNA integrity (RIN), autolysis score, and ischemic time stratified by age group. **(D–F)** Associations of these variables with individual structural aging (SA) scores. **(G–I)** Associations with delta SA scores. Only ischemic time showed a modest negative correlation with delta SA

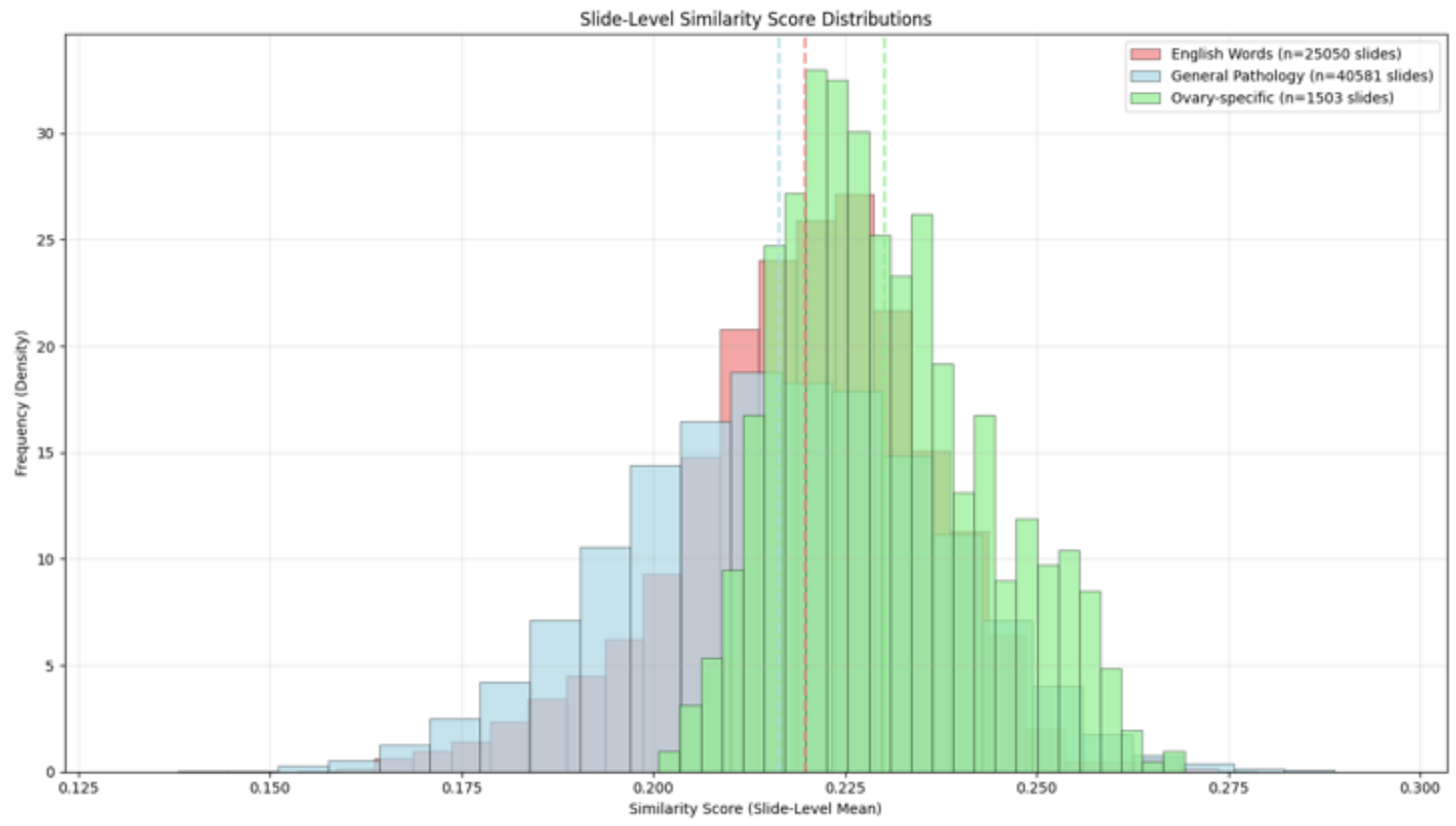

Supplementary Fig. 5: Slide-level similarity scores compared to different word categories. Distribution of slide-level (mean across patch) similarity scores between histopathology images and three categories of text terms: ovary-specific terms (green), general pathology terms (blue), and English words (red)

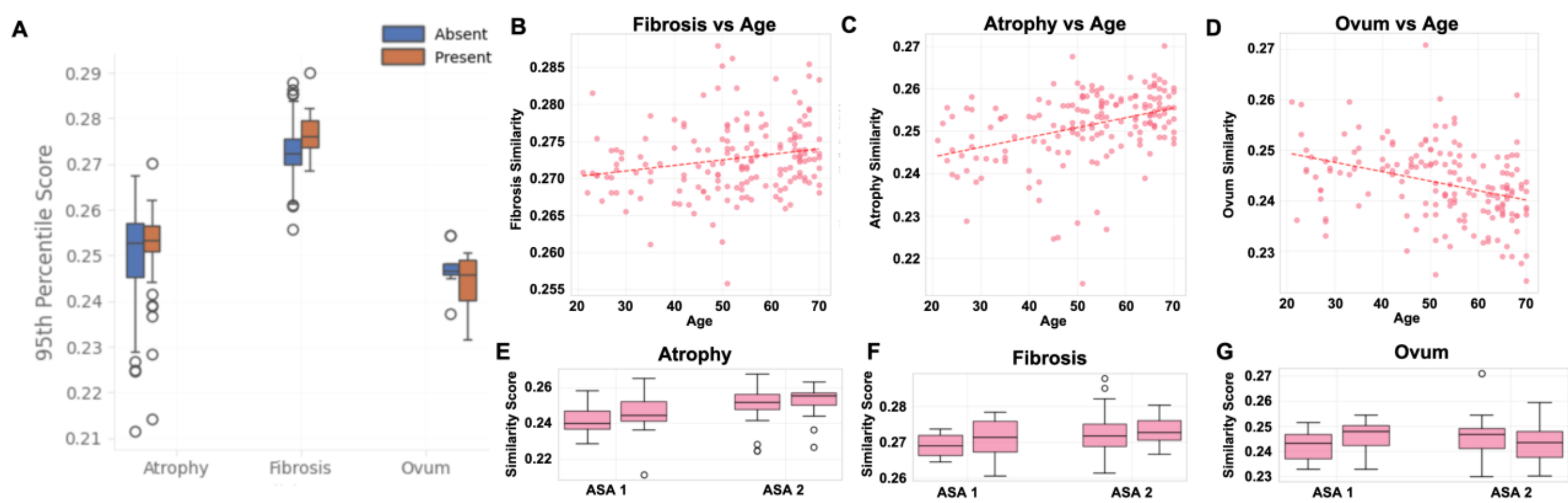

Supplementary Fig. 6: Interpretation of two ovarian remodeling periods. (A) Box plots comparing 95th percentile similarity scores between pathology-present and pathology-absent cases for fibrosis (n=160 absent, n=7 present), atrophy (n=105 absent, n=62 present), and ovum detection (n=9 each group). **(B-D)** Scatter plots with regression lines showing age-dependent trends in similarity scores for each pathological feature (atrophy: positive correlation; fibrosis: positive correlation; ovum: negative correlation). **(E-G)** Box plots stratifying similarity scores by reproductive age groups 25-30 years vs ASA 1 (35-40 years), 45-50 years vs ASA 2 (55-60 years) for each pathological feature, demonstrating distinct age-related patterns.

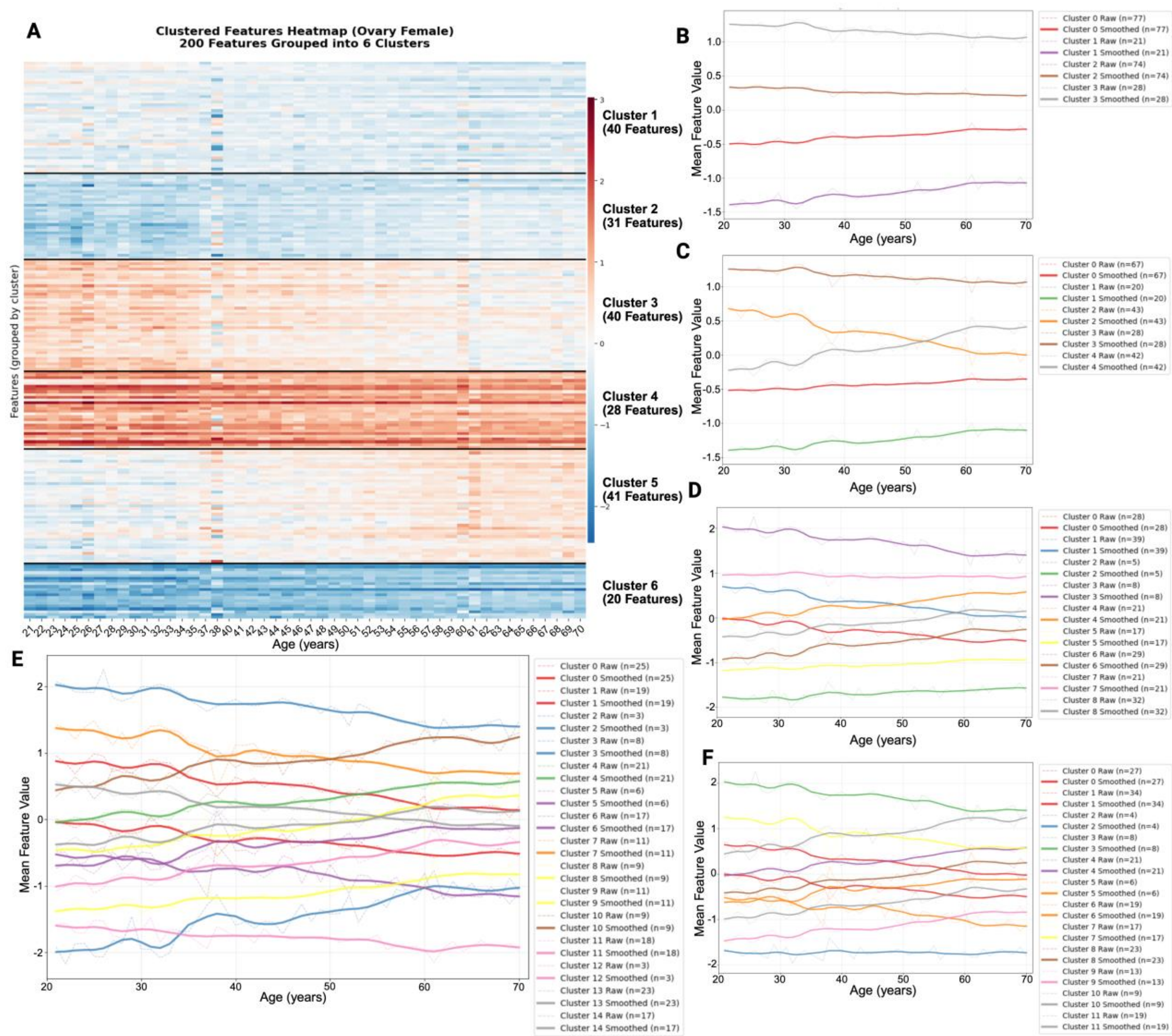

Supplementary Fig. 7: Identification of Distinct Ovarian Structural Remodeling Modules During Aging. PathStAR features were ranked by Normalized Mutual Information (NMI) with age, and the top 200 age-associated features were clustered to identify coordinated remodeling patterns. Clustering was performed with k = 4, 5, 6, 9, 12, 15, and 20 to determine optimal feature groupings. **(A)** Heatmap of the top 200 age-associated structural features in ovarian tissue, organized into 6 distinct clusters based on their temporal expression patterns. Features are arranged by cluster membership (indicated by color bars) and show clear age-dependent trajectories from ages 20-70 years. Red indicates higher feature values; blue indicates lower values. **(B-F)** Cluster trajectory plots showing feature group dynamics across different clustering solutions (k = 4, 5, 9, 12, and 15). Each line represents the mean trajectory of features within a cluster, revealing distinct remodeling modules with varying temporal patterns: early declining features, late-declining features, progressively increasing features and stepwise changes. Higher k values provide increased granularity in identifying coordinated structural changes that correspond to specific phases of ovarian aging, demonstrating that tissue remodeling occurs through orchestrated modules.

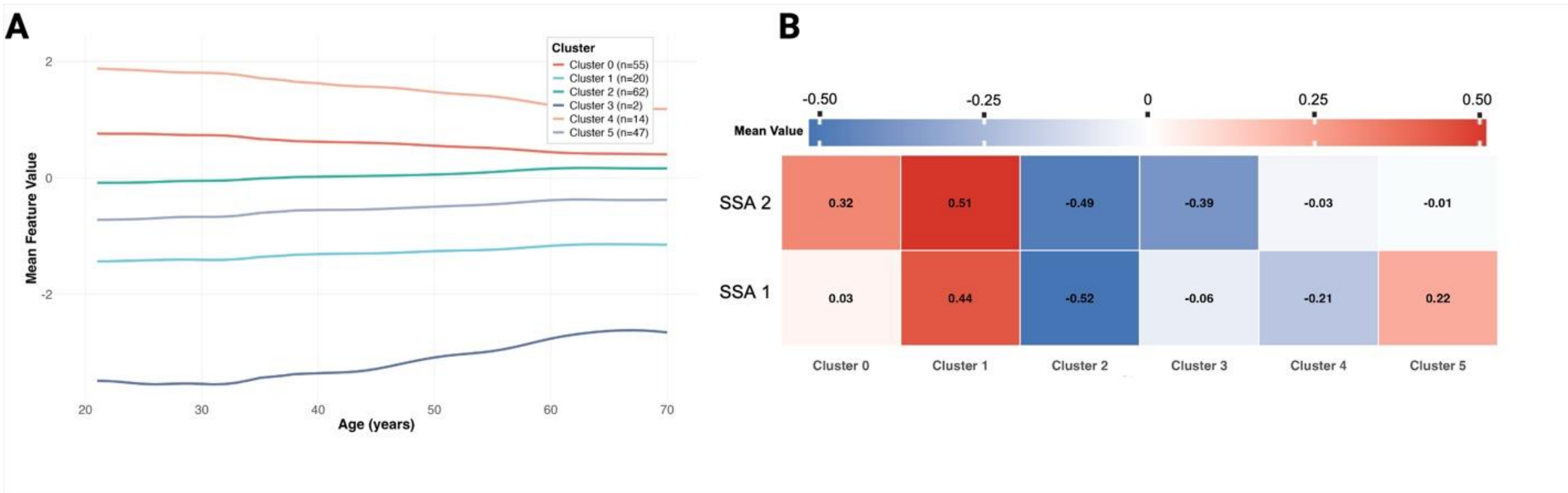

Supplementary Fig. 8: Feature value clustering vs Age and within different ASA period: (**A**) Six different morphological-features modules identified by clustering the top 200 UNI extracted features into 6 clusters, where cluster 0 and 1 showed highest decline with age opposed to cluster 3 which shows increase with age. **(B)** Heatmap showing cluster enrichment in ASA 1 and ASA 2.

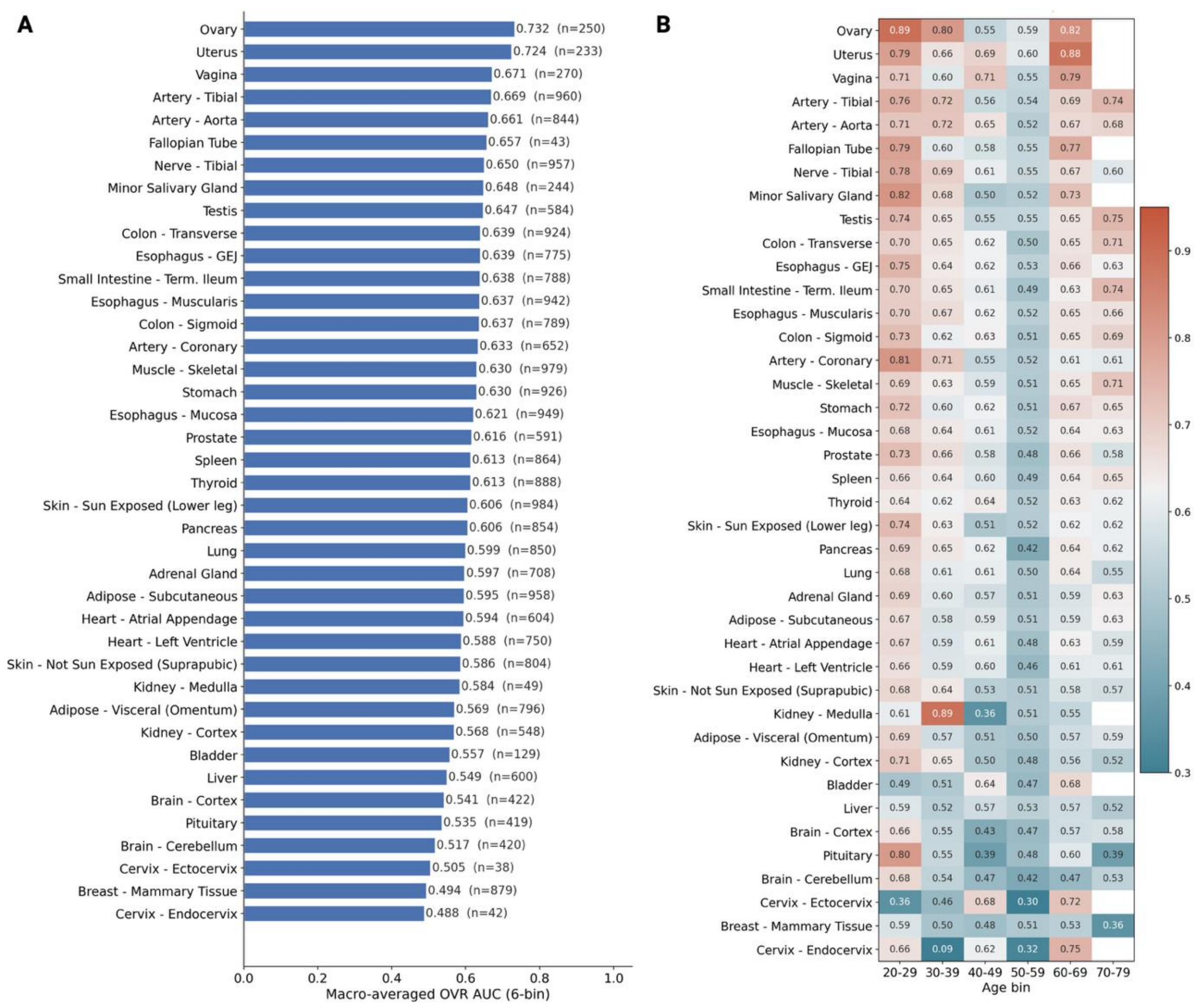

Supplementary Fig. 9: Macro-averaged AUC across different tissues and age bins: (A) Macro-averaged one-vs-rest AUC for multiclass logistic regression classification

across GTEx age-decade bins using only PC1 and PC2 of UNI-derived morphology features. **(B)** Per-age-bin one-vs-rest AUCs for each tissue. Rows are ordered by macro-AUC. Stronger separation is observed for the youngest and oldest age bins, whereas the 50–59 decade shows weaker discrimination across most tissues.

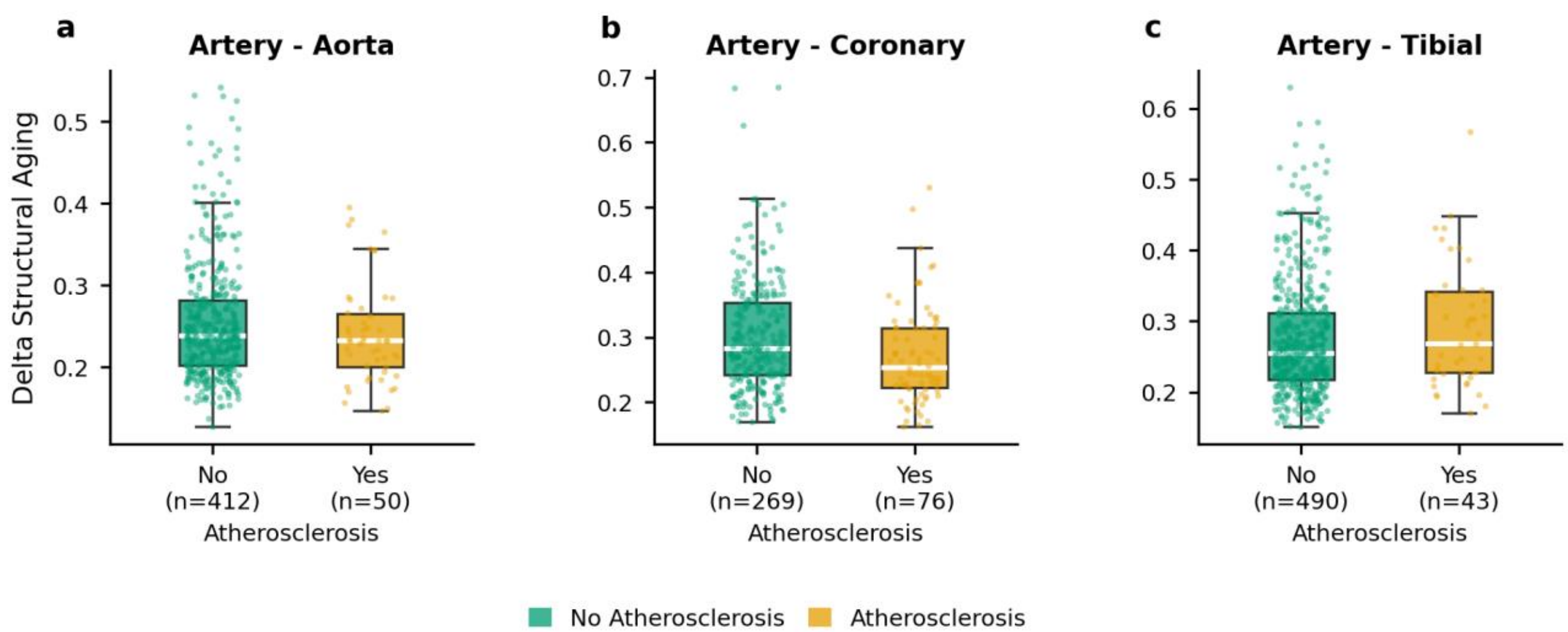

Supplementary Fig. 10: PathStAR Delta Scores Distinguish Atherosclerosis Presence. a-c, Box plots comparing delta SA scores between individuals with atherosclerosis (orange) and without atherosclerosis (green) across three arterial tissues in the 30-59 age group. Boxes show interquartile range (IQR) with median (white line); whiskers extend to 1.5× IQR. Individual data points are shown as overlaid dots. Group comparisons performed using two-sided Mann–Whitney U test. **(a)** Artery - Aorta: no atherosclerosis n=412, atherosclerosis n=50, Mann–Whitney U p = 0.299 **(b)** Artery - Coronary: no atherosclerosis n=269, atherosclerosis n=76, Mann–Whitney U p = 0.003 **(c)** Artery - Tibial: no atherosclerosis n=490, atherosclerosis n=43, Mann
Whitney U p = 0.185.

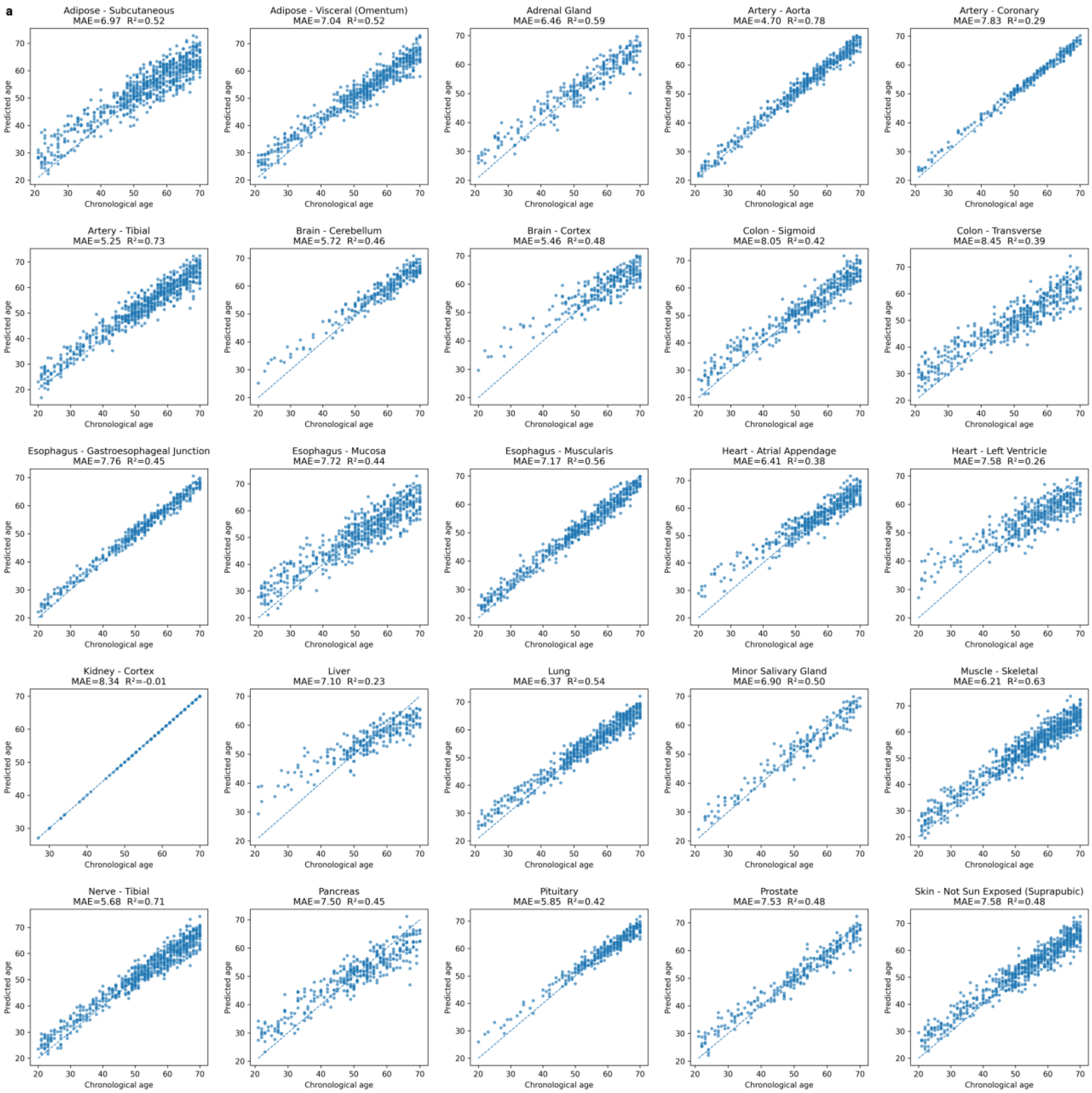
a
Adipose - Subcutaneous
MAE=6.97 R²=0.52
Adipose - Visceral (Omentum)
MAE=7.04 R²=0.52
Adrenal Gland
MAE=6.46 R²=0.59
Artery - Aorta
MAE=4.70 R²=0.78
Artery - Coronary
MAE=7.83 R²=0.29
Artery - Tibial
MAE=5.25 R²=0.73
Brain - Cerebellum
MAE=5.72 R²=0.46
Brain - Cortex
MAE=5.46 R²=0.48
Colon - Sigmoid
MAE=8.05 R²=0.42
Colon - Transverse
MAE=8.45 R²=0.39
Esophagus - Gastroesophageal Junction
MAE=7.76 R²=0.45
Esophagus - Mucosa
MAE=7.72 R²=0.44
Esophagus - Muscularis
MAE=7.17 R²=0.56
Heart - Atrial Appendage
MAE=6.41 R²=0.38
Heart - Left Ventricle
MAE=7.58 R²=0.26
Kidney - Cortex
MAE=8.34 R²=-0.01
Liver
MAE=7.10 R²=0.23
Lung
MAE=6.37 R²=0.54
Minor Salivary Gland
MAE=6.90 R²=0.50
Muscle - Skeletal
MAE=6.21 R²=0.63
Nerve - Tibial
MAE=5.68 R²=0.71
Pancreas
MAE=7.50 R²=0.45
Pituitary
MAE=5.85 R²=0.42
Prostate
MAE=7.53 R²=0.48
Skin - Not Sun Exposed (Suprapubic)
MAE=7.58 R²=0.48
Predicted age
Chronological age

b

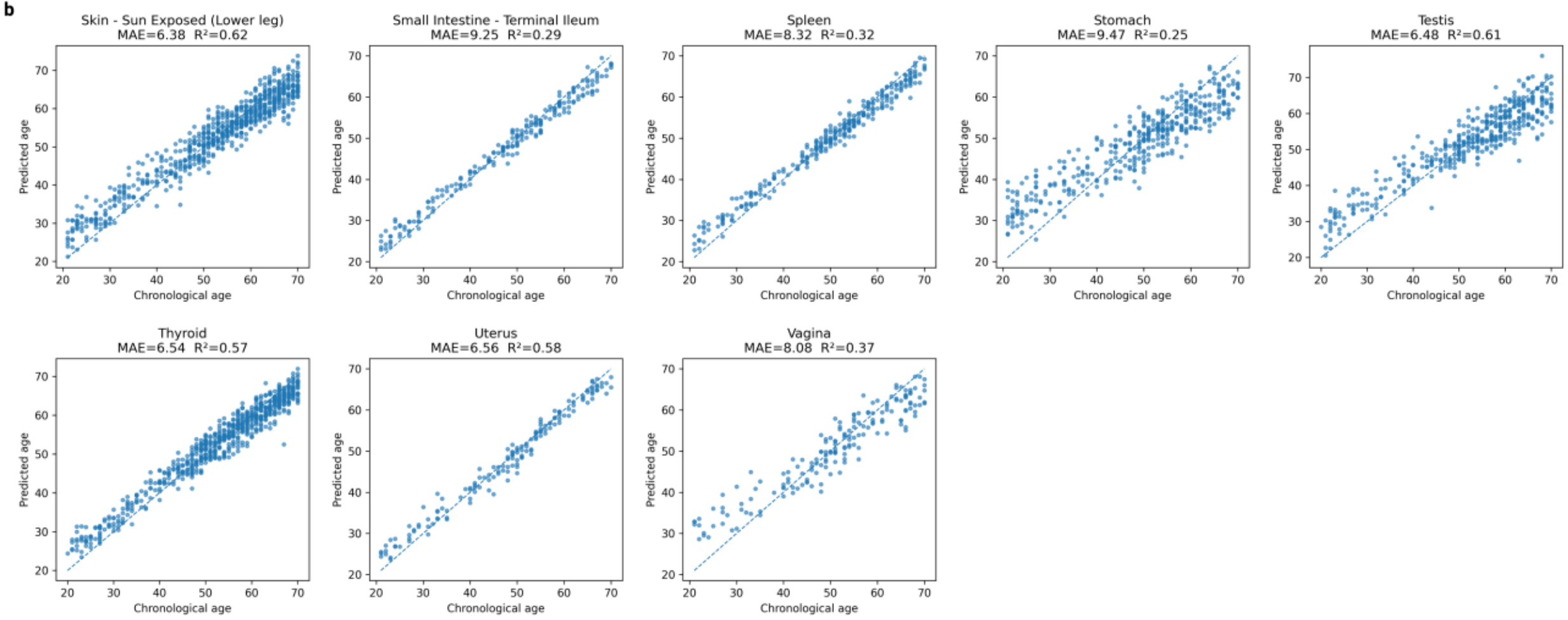

c

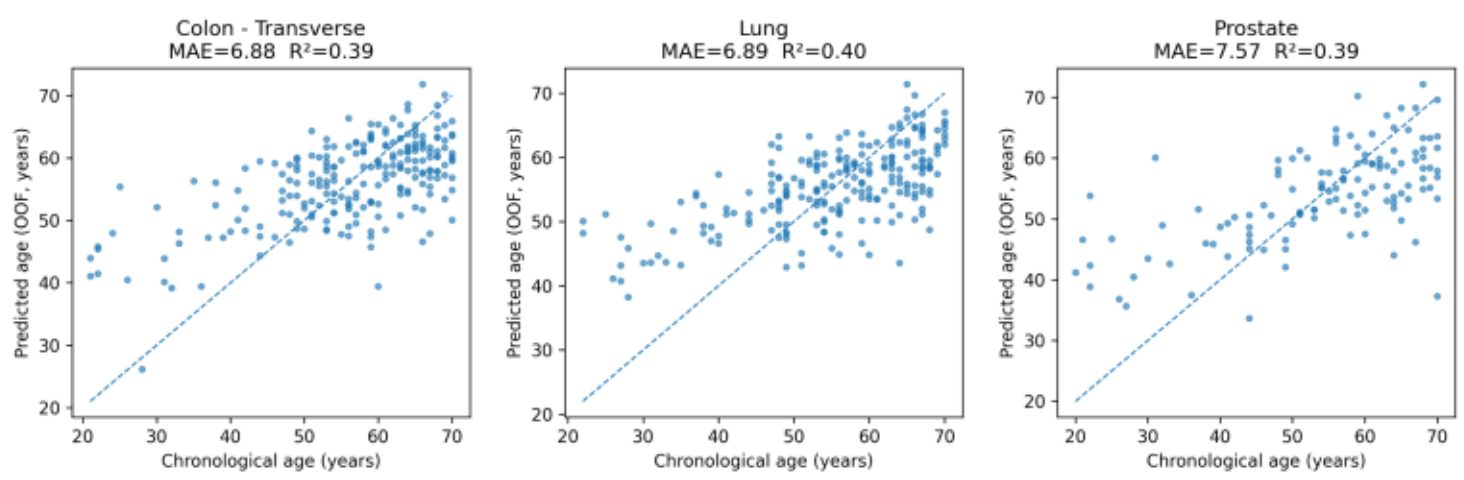

Supplementary Fig. 11: Transcriptomic Molecular Clocks. (a-b) Transcription-based molecular clocks trained separately for each tissue type using elastic net regression with 5-fold cross-validation. The clocks demonstrate strong linear correlations with chronological age across tissues (MAE range: 0.57-8.34 years), confirming their ability to capture age-associated molecular changes. However, these linear models fail to detect the non-linear aging trajectories and tissue-specific inflection points revealed by PathStAR's structural approach. **(c)Methylation Molecular Clocks:** Methylation-based molecular clocks trained for three tissue types with sufficient sample availability, using identical elastic net methodology as transcriptomic clocks. The clocks demonstrate strong linear correlations with chronological age across tissues (MAE range:1.57-8.88), confirming their ability to capture age-associated molecular changes.

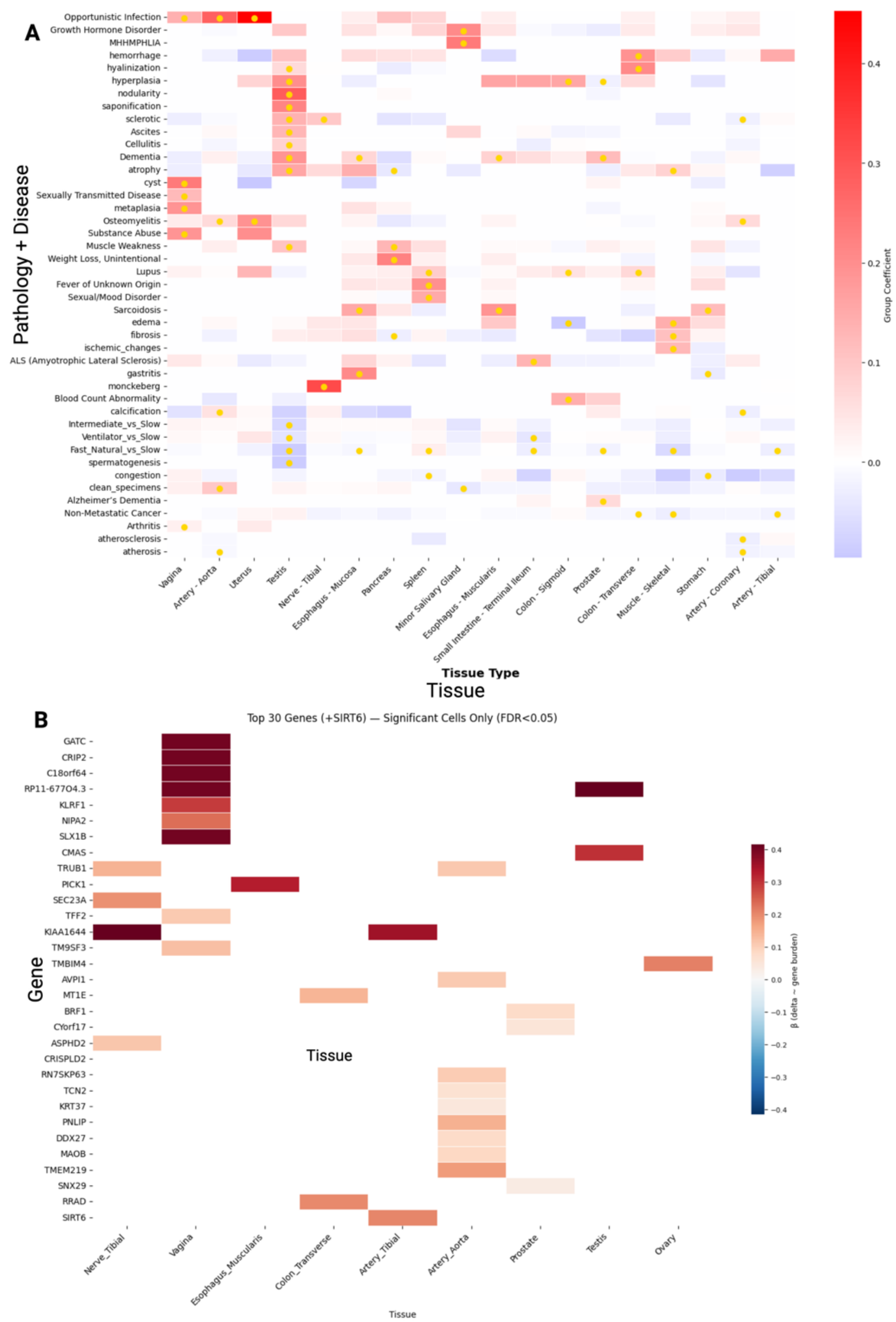

Supplementary Fig. 12: Heatmaps showing associations between delta-SA scores (ΔSA) and health/genetic factors across tissue types. (A) Heatmap of multivariate linear regression coefficients quantifying associations between individual ΔSA scores and pathological conditions (including atrophy, fibrosis, calcification, hyalinization, and systemic diseases) and post-mortem technical factors (including ischemic time, autolysis score, and RIN) across tissue types. Regression was performed separately per tissue with ΔSA score as the outcome; coefficients are shown without a significance threshold to display the full association landscape. Positive coefficients (red) indicate associations with accelerated structural remodeling; negative coefficients (blue) indicate associations

with decelerated remodeling. Color intensity reflects coefficient magnitude.The circles represent significant association where p-value <0.05. **(B)** Heatmap of gene-level associations between functional germline variants and tissue-specific ΔSA scores in n = 970 individuals. Functional variants were defined as non-synonymous, splice-site, and stop-gain variants and filtered from 998,000 starting variants to a final set of 127,000 tested variants (Methods 5.9). For each gene, variants were aggregated and tested against per-tissue ΔSA scores using linear regression. FDR-adjusted P values were computed using the Benjamini–Hochberg procedure. Genes with at least one tissue association meeting FDR < 0.1 are shown; 17 representative genes from the 123 meeting this threshold are displayed. Color intensity reflects effect size magnitude, with red indicating associations with accelerated and blue with decelerated structural aging.

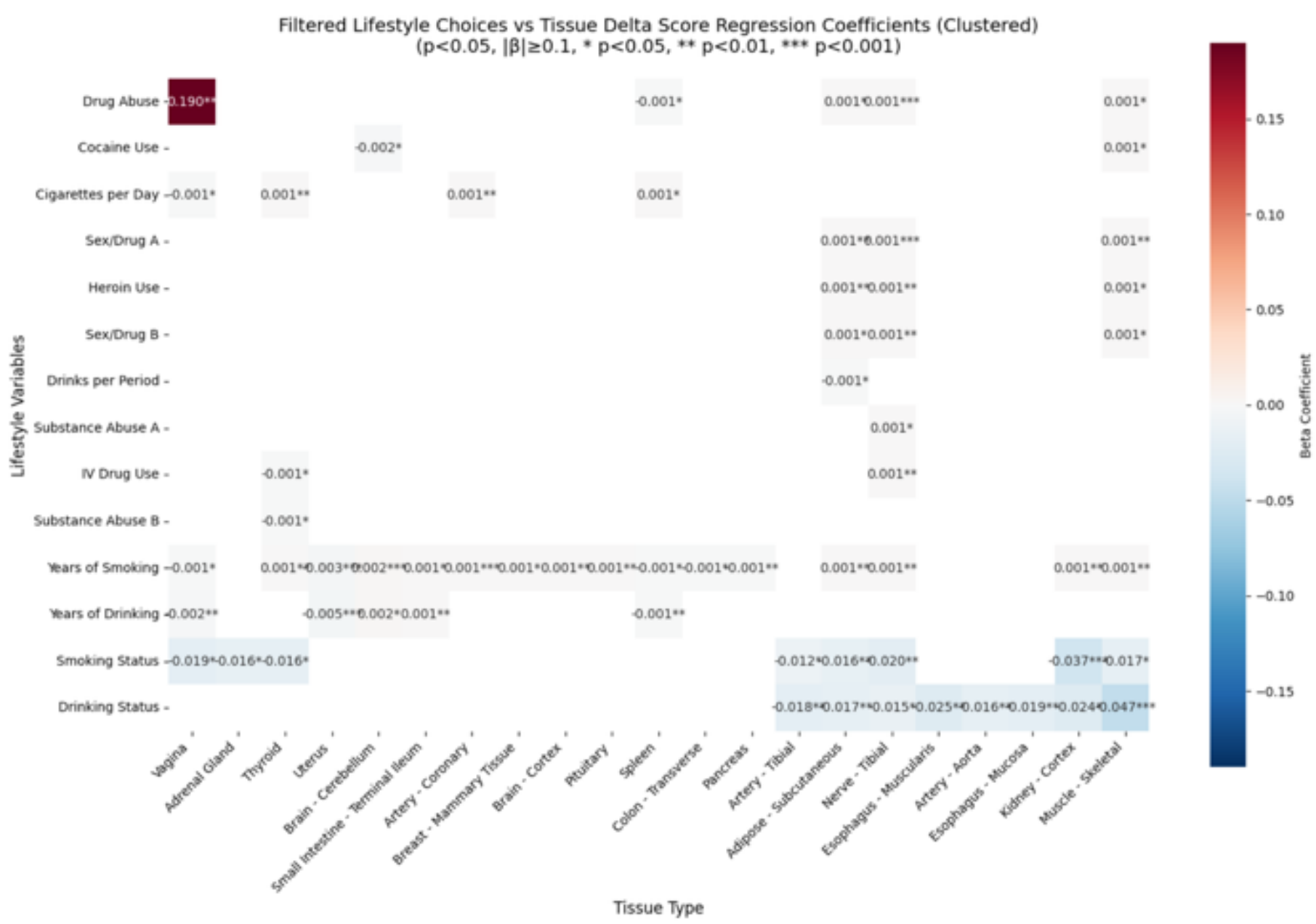

Supplementary Fig. 13: Association between lifestyle factors and tissue-specific accelerated structural aging (ΔSA) scores across GTEx samples. Heatmap displaying Mann–Whitney U test statistics comparing ΔSA scores between individuals with and without each lifestyle characteristic across tissue types (*n* = 970 individuals). Lifestyle factors were extracted from GTEx phenotypic metadata and include smoking history (current, former, or never smoker), alcohol consumption, recreational drug use, and self-reported physical activity. For each factor, individuals were stratified into binary groups (presence versus absence of the characteristic) and ΔSA distributions were compared between groups using a two-sided Mann–Whitney U test, a non-parametric test appropriate for non-normally distributed scores. No associations reached statistical significance after FDR correction (Benjamini–Hochberg), consistent with limited

statistical power due to small and unbalanced group sizes for several lifestyle variables in the GTEx cohort. Color intensity reflects the magnitude of the U statistic; red indicates higher ΔSA scores in the group with the lifestyle characteristic present, and blue indicates lower scores. The asterisk denotes p value - * : <0.05, **: <0.01, ***: <0.0001.

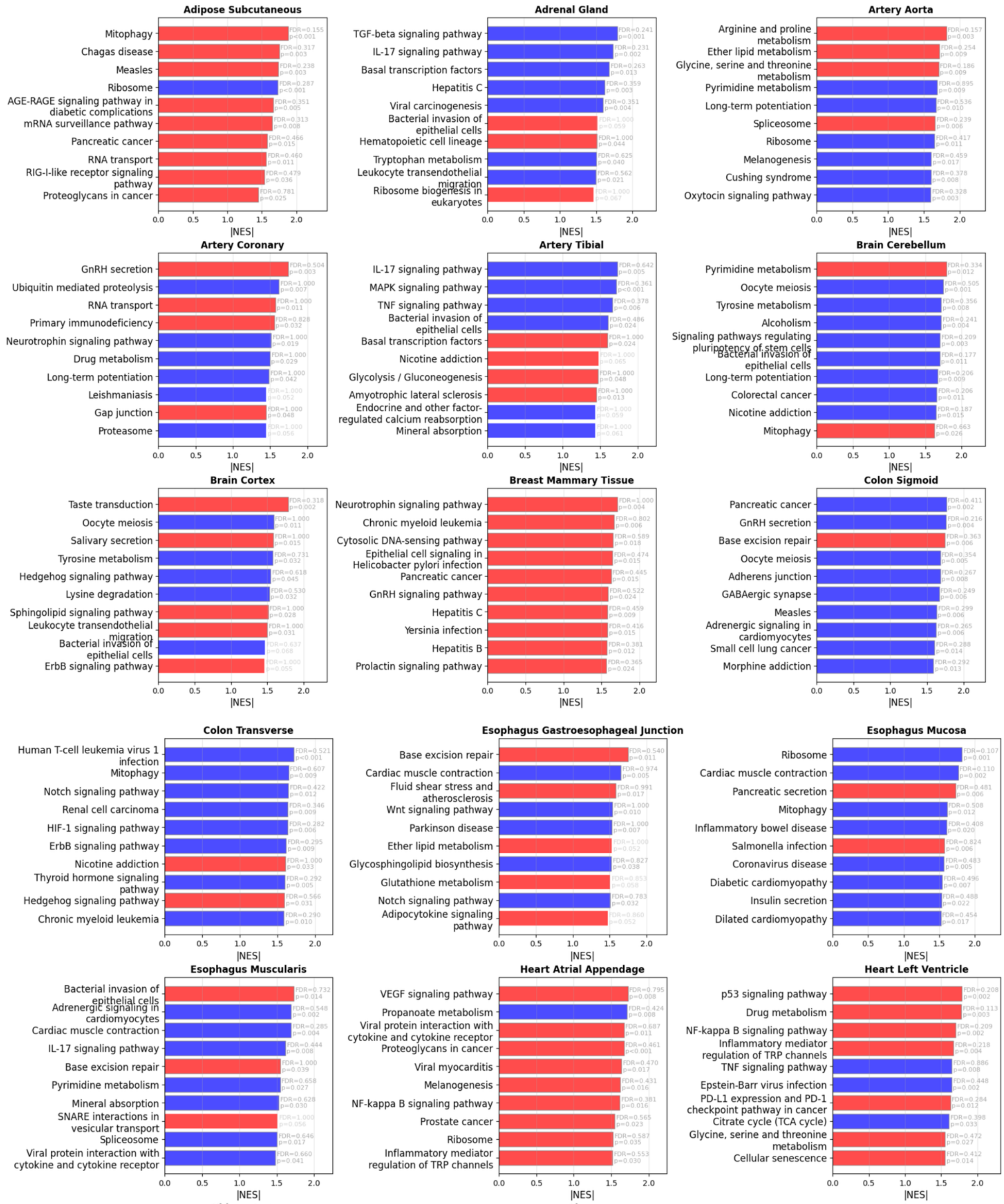

Adipose Subcutaneous
Mitophagy
Chagas disease
Measles
Ribosome
AGE-RAGE signaling pathway in diabetic complications
mRNA surveillance pathway
Pancreatic cancer
RNA transport
RIG-I-like receptor signaling pathway
Proteoglycans in cancer
|NES|
Adrenal Gland
TGF-beta signaling pathway
IL-17 signaling pathway
Basal transcription factors
Hepatitis C
Viral carcinogenesis
Bacterial invasion of epithelial cells
Hematopoietic cell lineage
Tryptophan metabolism
Leukocyte transendothelial migration
Ribosome biogenesis in eukaryotes
|NES|
Artery Aorta
Arginine and proline metabolism
Ether lipid metabolism
Glycine, serine and threonine metabolism
Pyrimidine metabolism
Long-term potentiation
Spliceosome
Ribosome
Melanogenesis
Cushing syndrome
Oxytocin signaling pathway
|NES|
Artery Coronary
GnRH secretion
Ubiquitin mediated proteolysis
RNA transport
Primary immunodeficiency
Neurotrophin signaling pathway
Drug metabolism
Long-term potentiation
Leishmaniasis
Gap junction
Proteasome
|NES|
Artery Tibial
IL-17 signaling pathway
MAPK signaling pathway
TNF signaling pathway
Bacterial invasion of epithelial cells
Basal transcription factors
Nicotine addiction
Glycolysis / Gluconeogenesis
Amyotrophic lateral sclerosis
Endocrine and other factor-regulated calcium reabsorption
Mineral absorption
|NES|
Brain Cerebellum
Pyrimidine metabolism
Oocyte meiosis
Tyrosine metabolism
Alcoholism
Signaling pathways regulating pluripotency of stem cells
Bacterial invasion of epithelial cells
Long-term potentiation
Colorectal cancer
Nicotine addiction
Mitophagy
|NES|
Brain Cortex
Taste transduction
Oocyte meiosis
Salivary secretion
Tyrosine metabolism
Hedgehog signaling pathway
Lysine degradation
Sphingolipid signaling pathway
Leukocyte transendothelial migration
Bacterial invasion of epithelial cells
ErbB signaling pathway
|NES|
Breast Mammary Tissue
Neurotrophin signaling pathway
Chronic myeloid leukemia
Cytosolic DNA-sensing pathway
Epithelial cell signaling in Helicobacter pylori infection
Pancreatic cancer
GnRH signaling pathway
Hepatitis C
Yersinia infection
Hepatitis B
Prolactin signaling pathway
|NES|
Colon Sigmoid
Pancreatic cancer
GnRH secretion
Base excision repair
Oocyte meiosis
Adherens junction
GABAergic synapse
Measles
Adrenergic signaling in cardiomyocytes
Small cell lung cancer
Morphine addiction
|NES|
Colon Transverse
Human T-cell leukemia virus 1 infection
Mitophagy
Notch signaling pathway
Renal cell carcinoma
HIF-1 signaling pathway
ErbB signaling pathway
Nicotine addiction
Thyroid hormone signaling pathway
Hedgehog signaling pathway
Chronic myeloid leukemia
|NES|
Esophagus Gastroesophageal Junction
Base excision repair
Cardiac muscle contraction
Fluid shear stress and atherosclerosis
Wnt signaling pathway
Parkinson disease
Ether lipid metabolism
Glycosphingolipid biosynthesis
Glutathione metabolism
Notch signaling pathway
Adipocytokine signaling pathway
|NES|
Esophagus Mucosa
Ribosome
Cardiac muscle contraction
Pancreatic secretion
Mitophagy
Inflammatory bowel disease
Salmonella infection
Coronavirus disease
Diabetic cardiomyopathy
Insulin secretion
Dilated cardiomyopathy
|NES|
Esophagus Muscularis
Bacterial invasion of epithelial cells
Adrenergic signaling in cardiomyocytes
Cardiac muscle contraction
IL-17 signaling pathway
Base excision repair
Pyrimidine metabolism
Mineral absorption
SNARE interactions in vesicular transport
Spliceosome
Viral protein interaction with cytokine and cytokine receptor
|NES|
Heart Atrial Appendage
VEGF signaling pathway
Propanoate metabolism
Viral protein interaction with cytokine and cytokine receptor
Proteoglycans in cancer
Viral myocarditis
Melanogenesis
NF-kappa B signaling pathway
Prostate cancer
Ribosome
Inflammatory mediator regulation of TRP channels
|NES|
Heart Left Ventricle
p53 signaling pathway
Drug metabolism
NF-kappa B signaling pathway
Inflammatory mediator regulation of TRP channels
TNF signaling pathway
Epstein-Barr virus infection
PD-L1 expression and PD-1 checkpoint pathway in cancer
Citrate cycle (TCA cycle)
Glycine, serine and threonine metabolism
Cellular senescence
|NES|

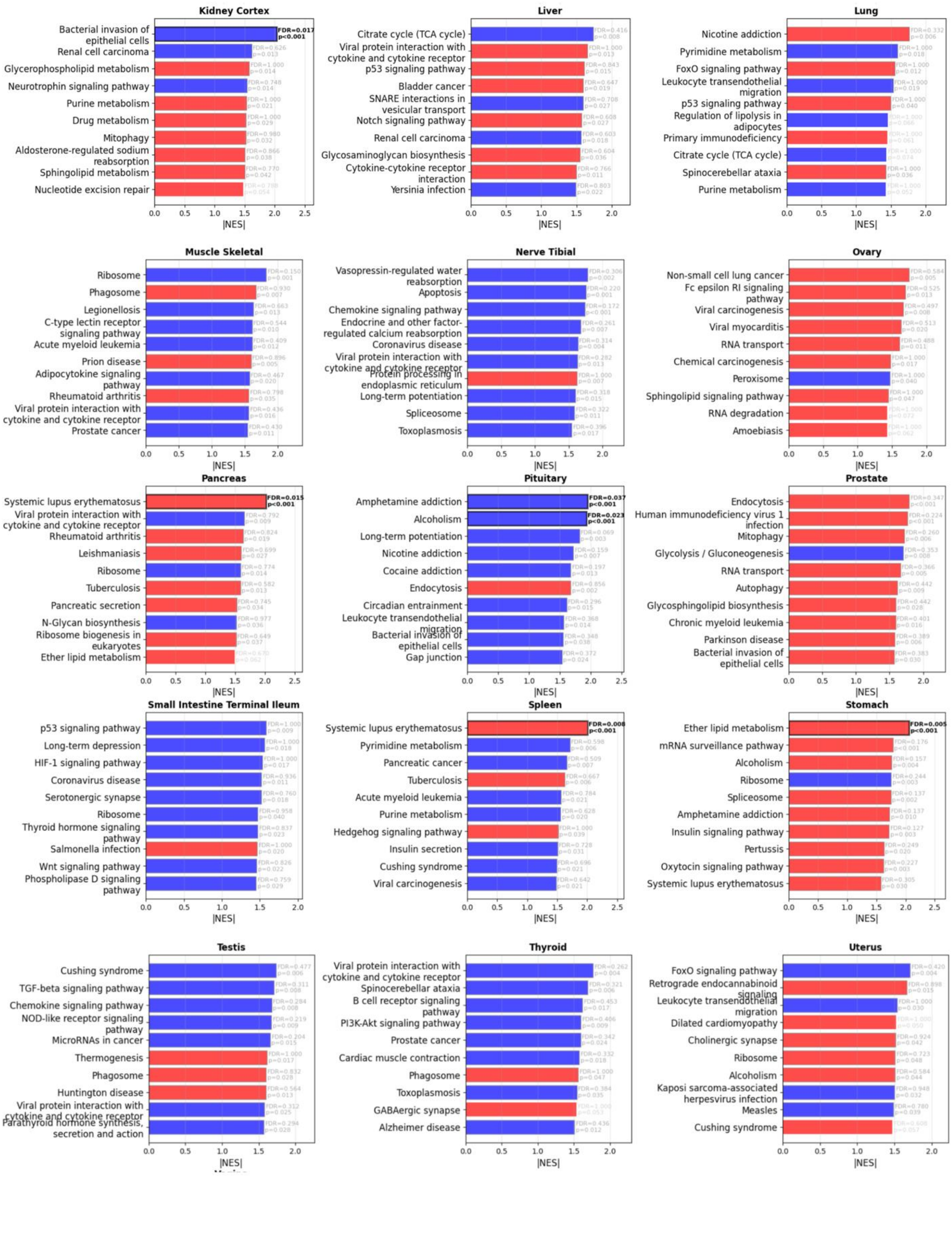
Kidney Cortex
Bacterial invasion of epithelial cells
FDR=0.017
p<0.001
Renal cell carcinoma
Glycerophospholipid metabolism
Neurotrophin signaling pathway
Purine metabolism
Drug metabolism
Mitophagy
Aldosterone-regulated sodium reabsorption
Sphingolipid metabolism
Nucleotide excision repair
|NES|
Liver
Citrate cycle (TCA cycle)
Viral protein interaction with cytokine and cytokine receptor
p53 signaling pathway
Bladder cancer
SNARE interactions in vesicular transport
Notch signaling pathway
Renal cell carcinoma
Glycosaminoglycan biosynthesis
Cytokine-cytokine receptor interaction
Yersinia infection
|NES|
Lung
Nicotine addiction
Pyrimidine metabolism
FoxO signaling pathway
Leukocyte transendothelial migration
p53 signaling pathway
Regulation of lipolysis in adipocytes
Primary immunodeficiency
Citrate cycle (TCA cycle)
Spinocerebellar ataxia
Purine metabolism
|NES|
Muscle Skeletal
Ribosome
Phagosome
Legionellosis
C-type lectin receptor signaling pathway
Acute myeloid leukemia
Prion disease
Adipocytokine signaling pathway
Rheumatoid arthritis
Viral protein interaction with cytokine and cytokine receptor
Prostate cancer
|NES|
Nerve Tibial
Vasopressin-regulated water reabsorption
Apoptosis
Chemokine signaling pathway
Endocrine and other factor-regulated calcium reabsorption
Coronavirus disease
Viral protein interaction with cytokine and cytokine receptor
Protein processing in endoplasmic reticulum
Long-term potentiation
Spliceosome
Toxoplasmosis
|NES|
Ovary
Non-small cell lung cancer
Fc epsilon RI signaling pathway
Viral carcinogenesis
Viral myocarditis
RNA transport
Chemical carcinogenesis
Peroxisome
Sphingolipid signaling pathway
RNA degradation
Amoebiasis
|NES|
Pancreas
Systemic lupus erythematosus
FDR=0.015
p<0.001
Viral protein interaction with cytokine and cytokine receptor
Rheumatoid arthritis
Leishmaniasis
Ribosome
Tuberculosis
Pancreatic secretion
N-Glycan biosynthesis
Ribosome biogenesis in eukaryotes
Ether lipid metabolism
|NES|
Pituitary
Amphetamine addiction
FDR=0.037
p<0.001
Alcoholism
FDR=0.023
p<0.001
Long-term potentiation
Nicotine addiction
Cocaine addiction
Endocytosis
Circadian entrainment
Leukocyte transendothelial migration
Bacterial invasion of epithelial cells
Gap junction
|NES|
Prostate
Endocytosis
Human immunodeficiency virus 1 infection
Mitophagy
Glycolysis / Gluconeogenesis
RNA transport
Autophagy
Glycosphingolipid biosynthesis
Chronic myeloid leukemia
Parkinson disease
Bacterial invasion of epithelial cells
|NES|
Small Intestine Terminal Ileum
p53 signaling pathway
Long-term depression
HIF-1 signaling pathway
Coronavirus disease
Serotonergic synapse
Ribosome
Thyroid hormone signaling pathway
Salmonella infection
Wnt signaling pathway
Phospholipase D signaling pathway
|NES|
Spleen
Systemic lupus erythematosus
FDR=0.008
p<0.001
Pyrimidine metabolism
Pancreatic cancer
Tuberculosis
Acute myeloid leukemia
Purine metabolism
Hedgehog signaling pathway
Insulin secretion
Cushing syndrome
Viral carcinogenesis
|NES|
Stomach
Ether lipid metabolism
FDR=0.005
p<0.001
mRNA surveillance pathway
Alcoholism
Ribosome
Spliceosome
Amphetamine addiction
Insulin signaling pathway
Pertussis
Oxytocin signaling pathway
Systemic lupus erythematosus
|NES|
Testis
Cushing syndrome
TGF-beta signaling pathway
Chemokine signaling pathway
NOD-like receptor signaling pathway
MicroRNAs in cancer
Thermogenesis
Phagosome
Huntington disease
Viral protein interaction with cytokine and cytokine receptor
Parathyroid hormone synthesis, secretion and action
|NES|
Thyroid
Viral protein interaction with cytokine and cytokine receptor
Spinocerebellar ataxia
B cell receptor signaling pathway
PI3K-Akt signaling pathway
Prostate cancer
Cardiac muscle contraction
Phagosome
Toxoplasmosis
GABAergic synapse
Alzheimer disease
|NES|
Uterus
FoxO signaling pathway
Retrograde endocannabinoid signaling
Leukocyte transendothelial migration
Dilated cardiomyopathy
Cholinergic synapse
Ribosome
Alcoholism
Kaposi sarcoma-associated herpesvirus infection
Measles
Cushing syndrome
|NES|

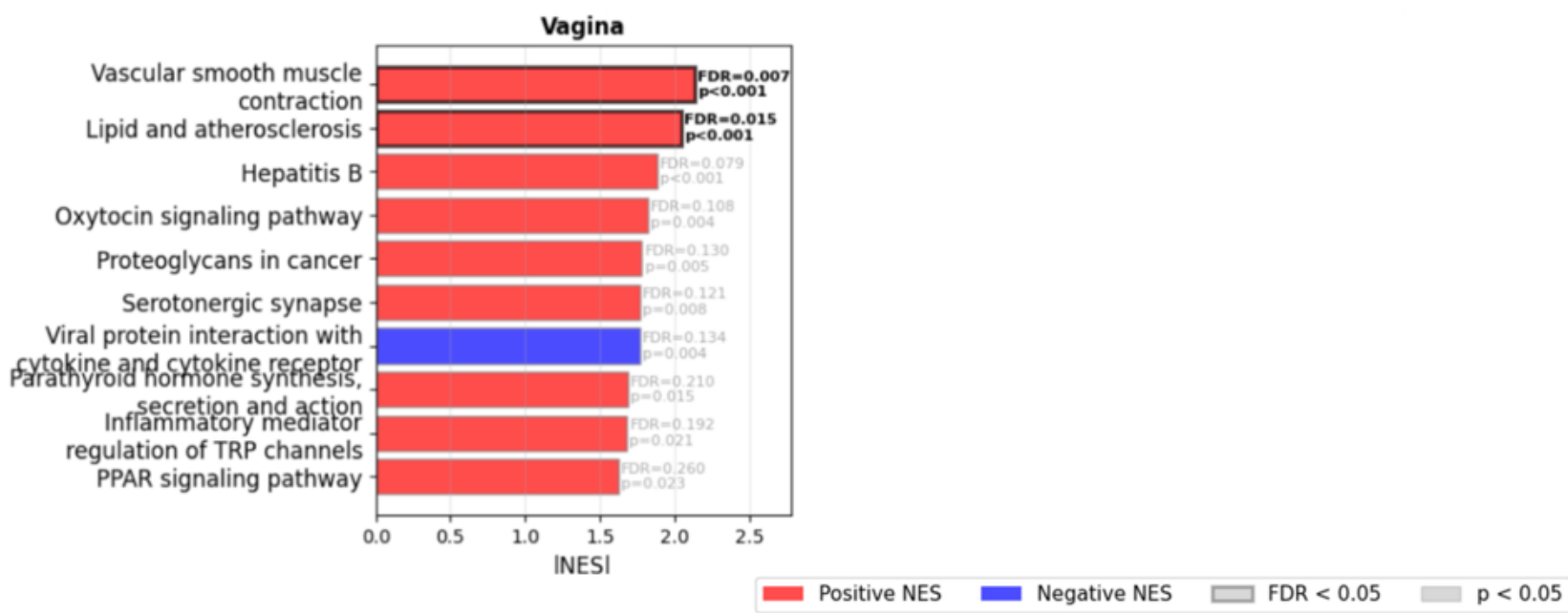

Supplementary Fig. 14: **Tissue-specific Hallmark Pathway Gene Enrichment.** Tissue-specific Hallmark Pathways enriched in genes whose germline variants were associated with delta-SA score. The Blue represents Negative NES value and red represent Positive NES value. FDR and P value for significant pathways

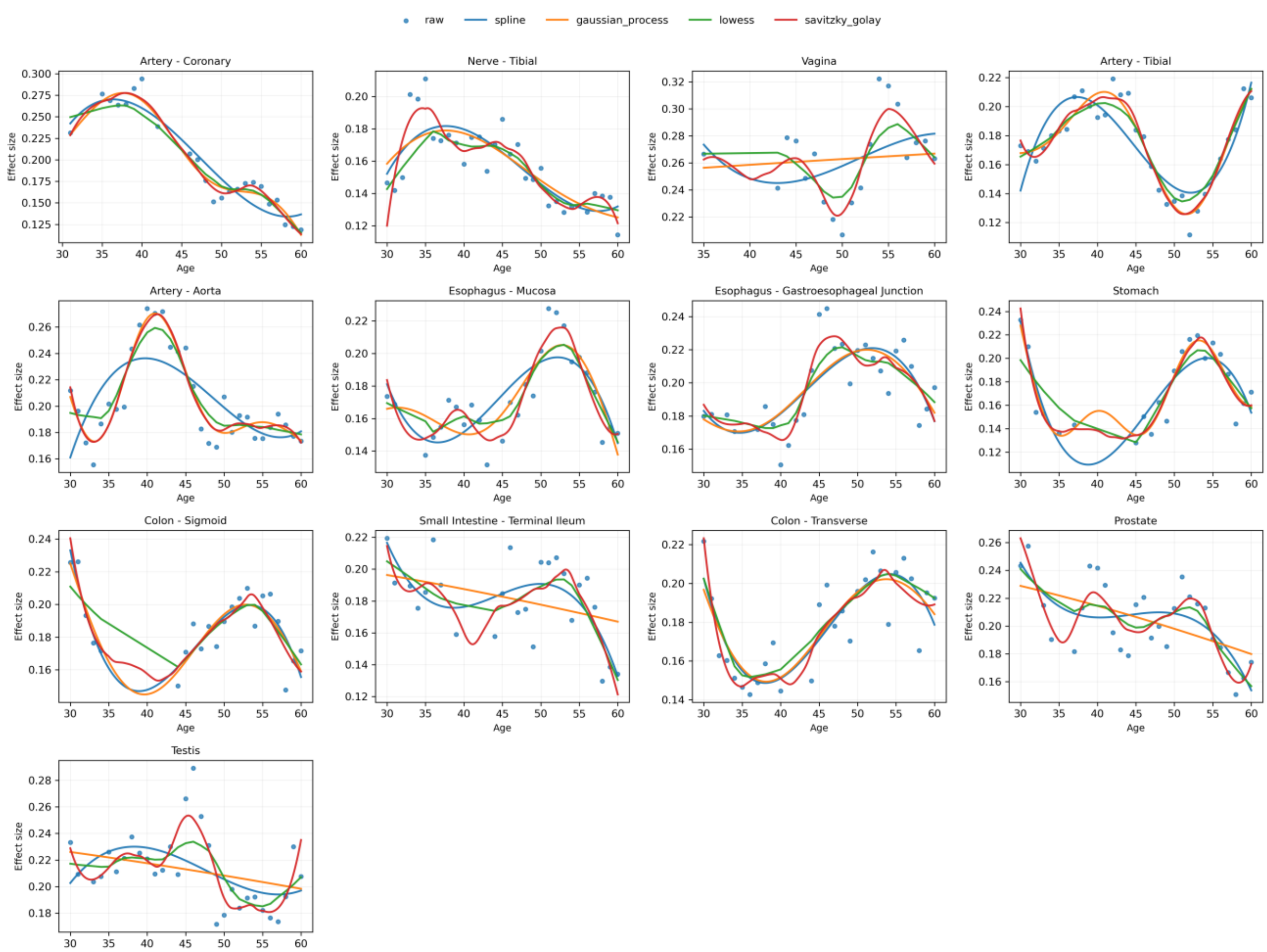

Supplementary Fig. 15: Effect of Smoothing Methods on Raw Structural Aging Trajectories. Raw Structural Aging Trajectories derived from UNI v1 embeddings are shown for 15 tissues: Artery (Aorta, Coronary, Tibial), Colon (Sigmoid, Transverse), Esophagus (Gastroesophageal Junction, Muscularis), Nerve (Tibial), Ovary, Prostate, Small Intestine (Terminal Ileum), Stomach, Testis, Uterus, and Vagina. Blue dots represent the raw effect size at each adjacent age transition, reflecting the magnitude of morphological change between consecutive age windows. To evaluate the influence of smoothing strategy on trajectory shape, four commonly used approaches were applied: cubic spline interpolation (blue), Gaussian process regression (orange), LOWESS (green), and Savitzky–Golay filtering (red). While all methods recover similar global trends, methods such as LOWESS and Savitzky–Golay introduce local fluctuations in some tissues, and Gaussian process smoothing can produce broader curvature depending on kernel assumptions. Cubic spline smoothing captures the main inflection points and periods of accelerated remodeling while avoiding unnecessary fluctuations. It provides a smooth and stable fit without overfitting the data. Based on this balance, spline fitting was used for primary PathStAR analyses.

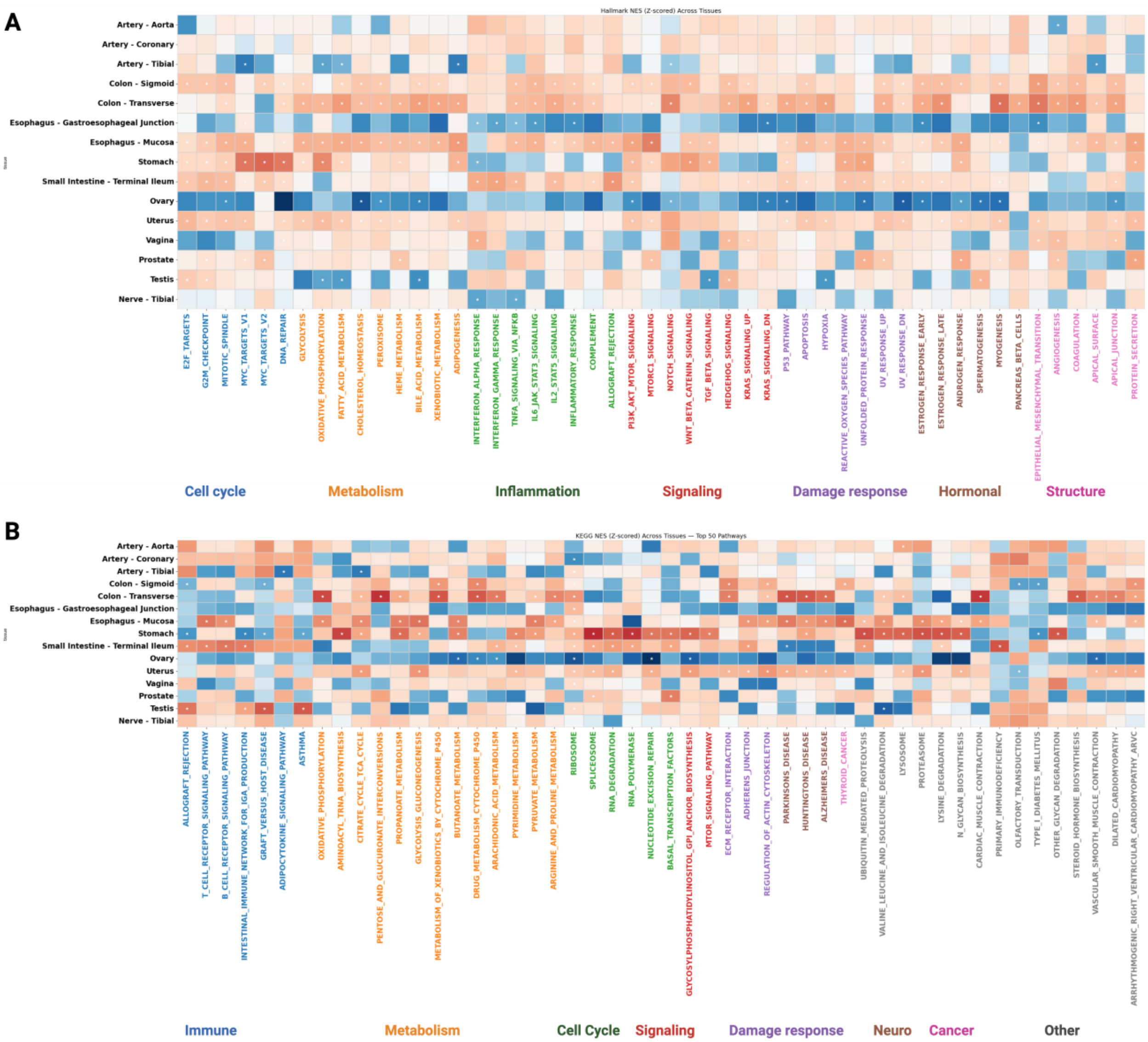

Supplementary Fig. 16: Pathways enrichment of genes that follow structural aging trajectory. (A) based on hallmarks pathways and **(B)** based on KEGG pathways. Red pathways are positively correlated with structural aging rate and vice versa for blue, significant pathways are represented with white dots where p-value <0.05.

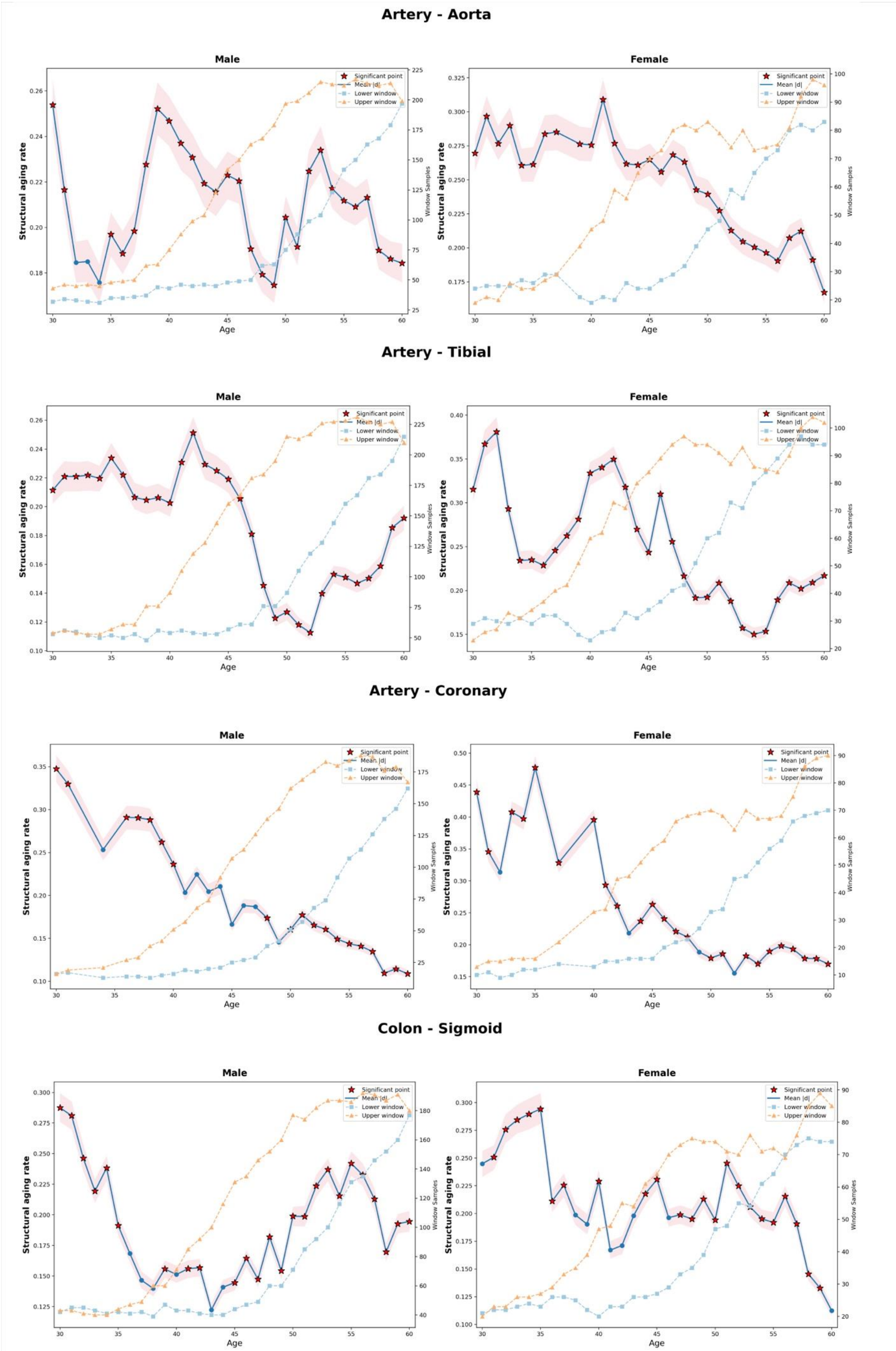
Artery - Aorta
Male
Female
Artery - Tibial
Male
Female
Artery - Coronary
Male
Female
Colon - Sigmoid
Male
Female
Significant point
Mean |d|
Lower window
Upper window
Structural aging rate
Window Samples
Age

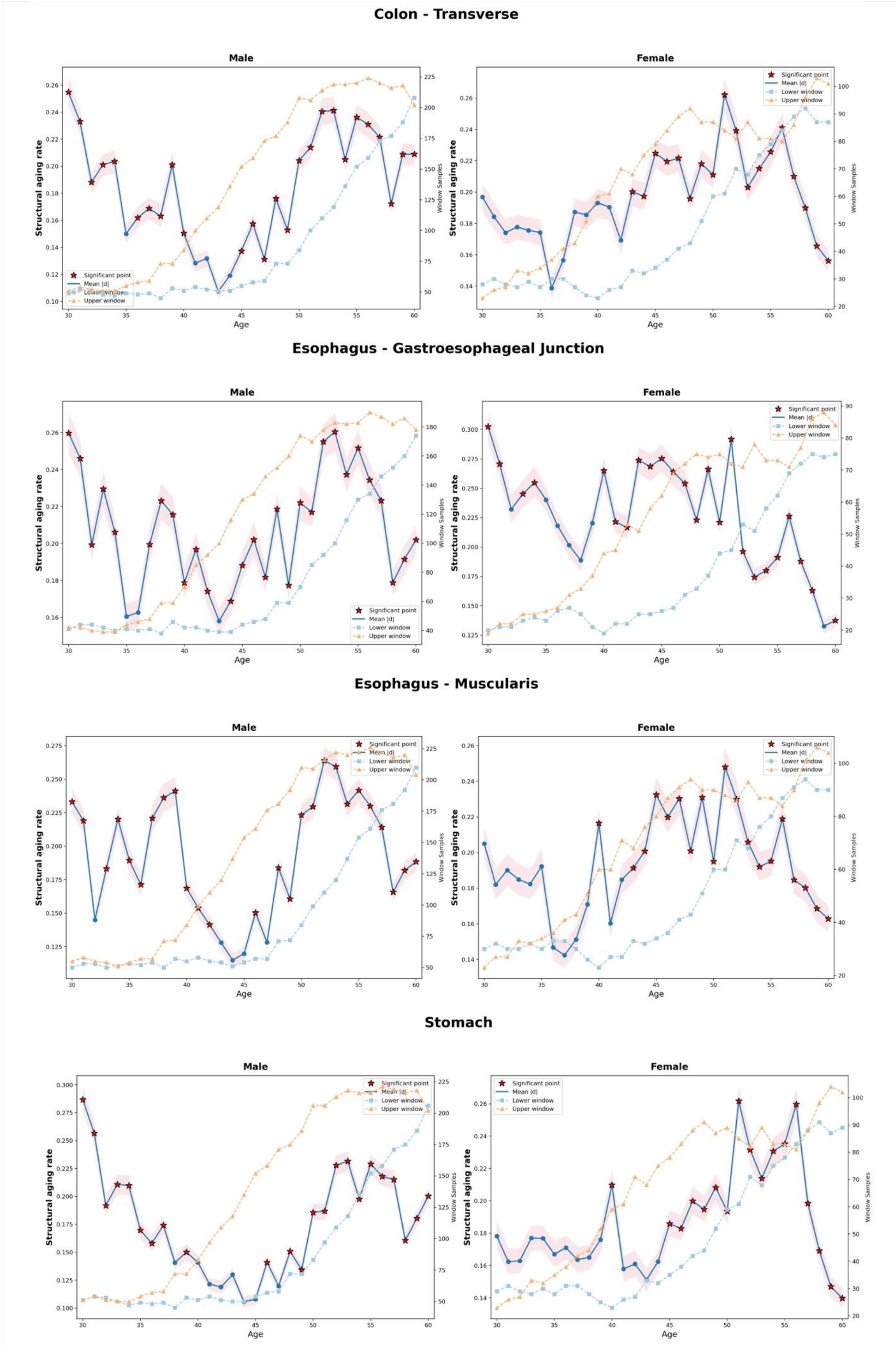
Colon - Transverse
Male
Female
Esophagus - Gastroesophageal Junction
Male
Female
Esophagus - Muscularis
Male
Female
Stomach
Male
Female
Structural aging rate
Window Samples
Age
Significant point
Mean |d|
Lower window
Upper window

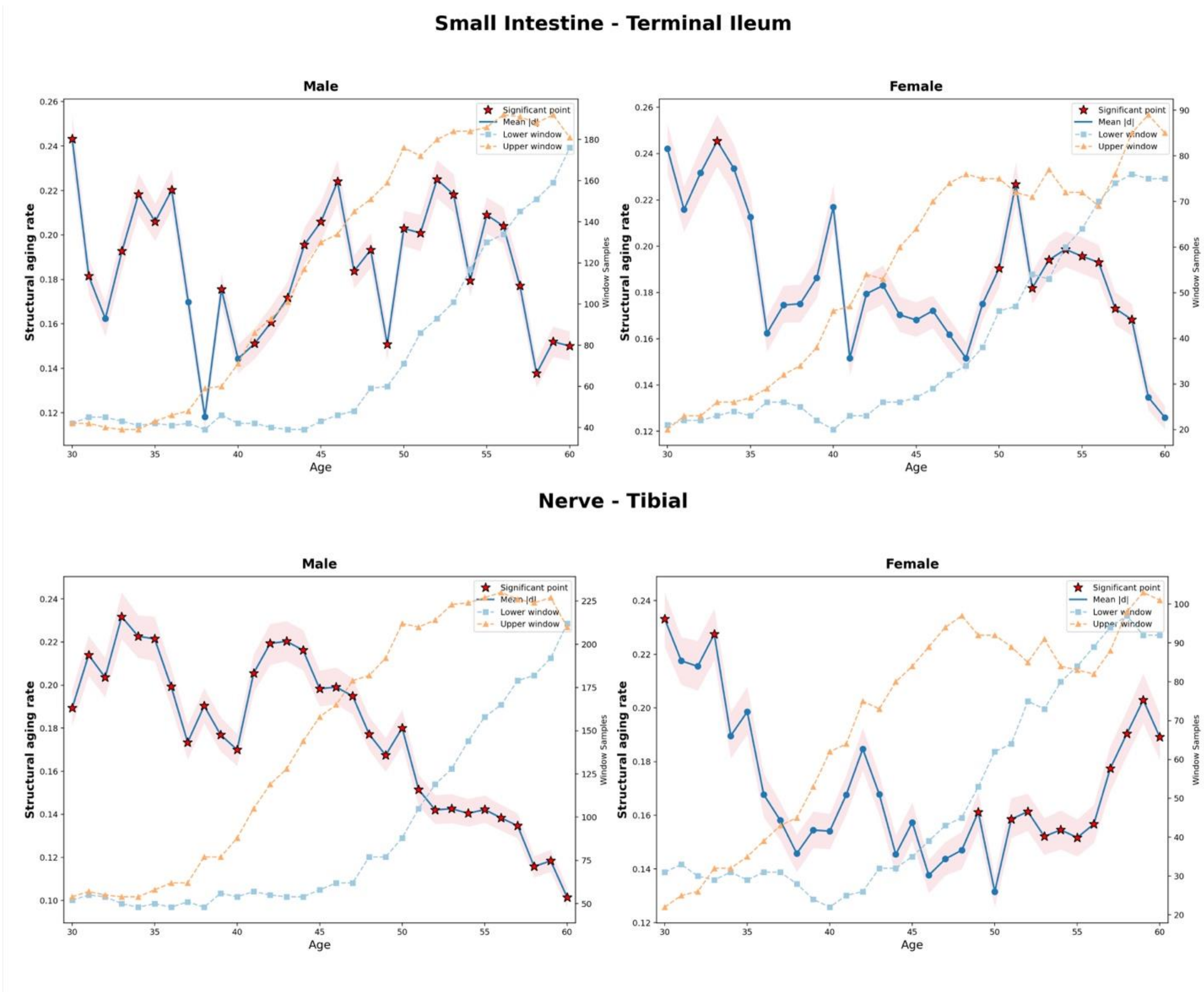

Supplementary Fig 17: Sex-stratified trajectories of structural aging across human tissues: Line plots depict age-related structural aging rates, estimated as the mean absolute effect size between adjacent 10-year age windows, separately for male (left) and female (right) donors. Blue lines indicate effect sizes, with shaded regions showing the 95% confidence intervals obtained by bootstrap resampling. Red stars mark significant age transitions (≥5% of features with Welch's *t*-test $p < 0.05$). Orange and light-blue dashed lines indicate the sample counts contributing to the upper and lower age windows, respectively. Panels summarize 10 of the 15 tissues studied here; the remaining five tissues (Prostate, Testis, Ovary, Uterus, and Vagina) are sex-specific and are shown in Extended Data Fig. 3.

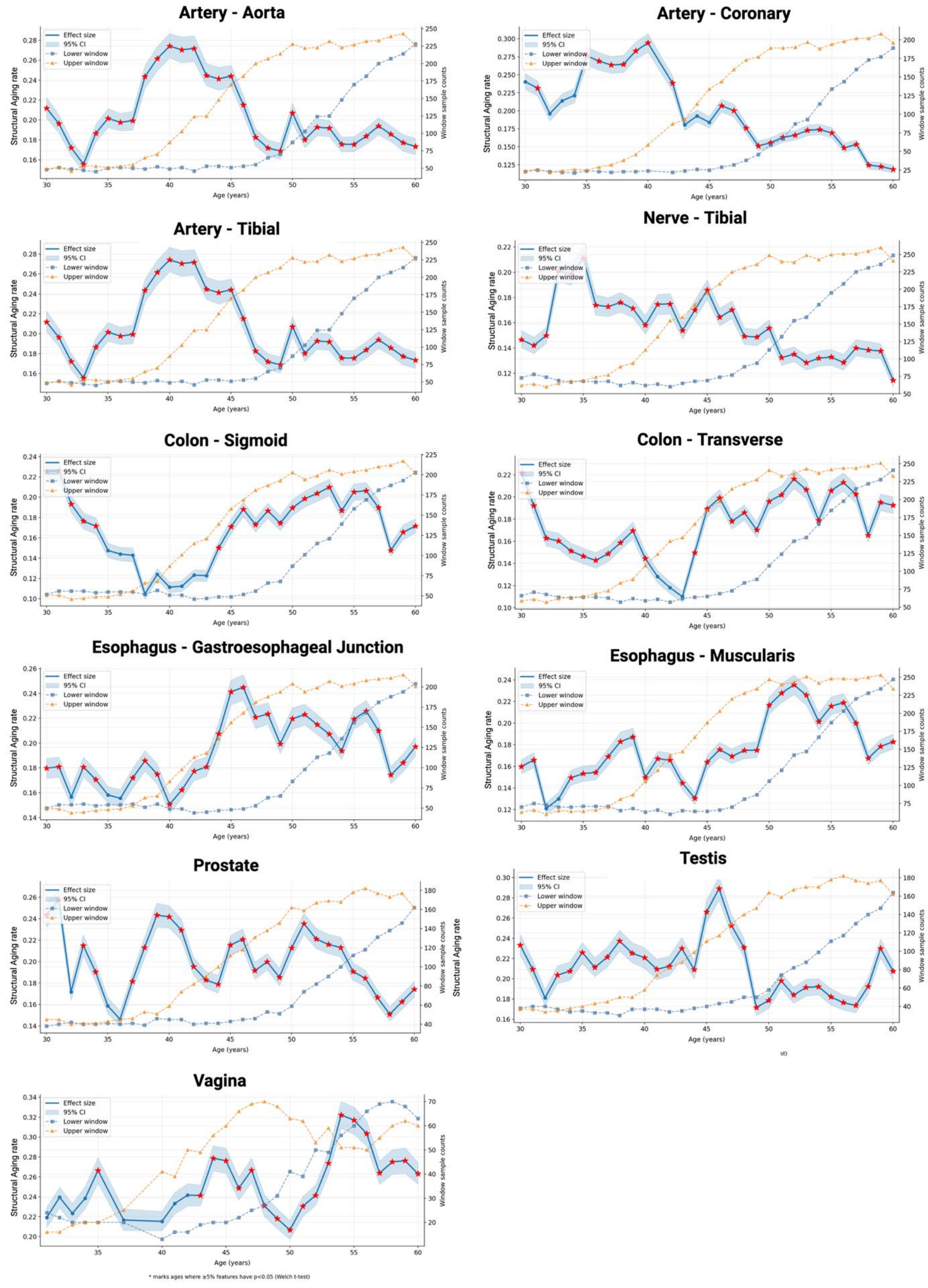

* marks ages where ≥5% features have p<0.05 (Welch t-test)

Supplementary Fig 18: **Raw trajectories after removing the diseased samples**: The plots show age-related structural aging rates quantified as the mean absolute effect size between adjacent 10-year age windows (blue line, left *y*-axis). Shaded regions represent the 95% confidence interval of the effect size, estimated by bootstrap resampling. Red stars indicate significant age transitions (≥5% of features with Welch's *t*-test $p < 0.05$). Sample counts contributing to each transition are shown for the lower (blue dashed line, right *y*-axis) and upper (orange dashed line, right *y*-axis) age windows. Each panel depicts trajectories for a distinct tissue, restricted to donors without major medical conditions or cancers, thereby capturing baseline, non-diseased structural aging dynamics.

Adipose - Subcutaneous

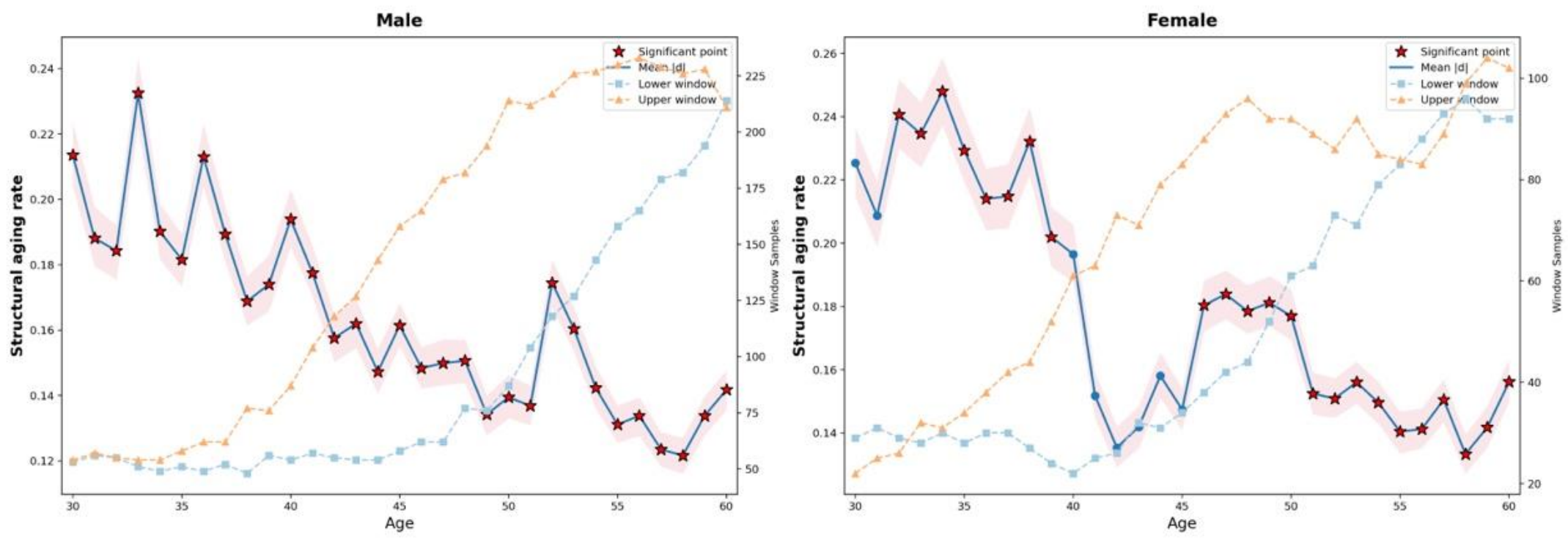

Adrenal Gland

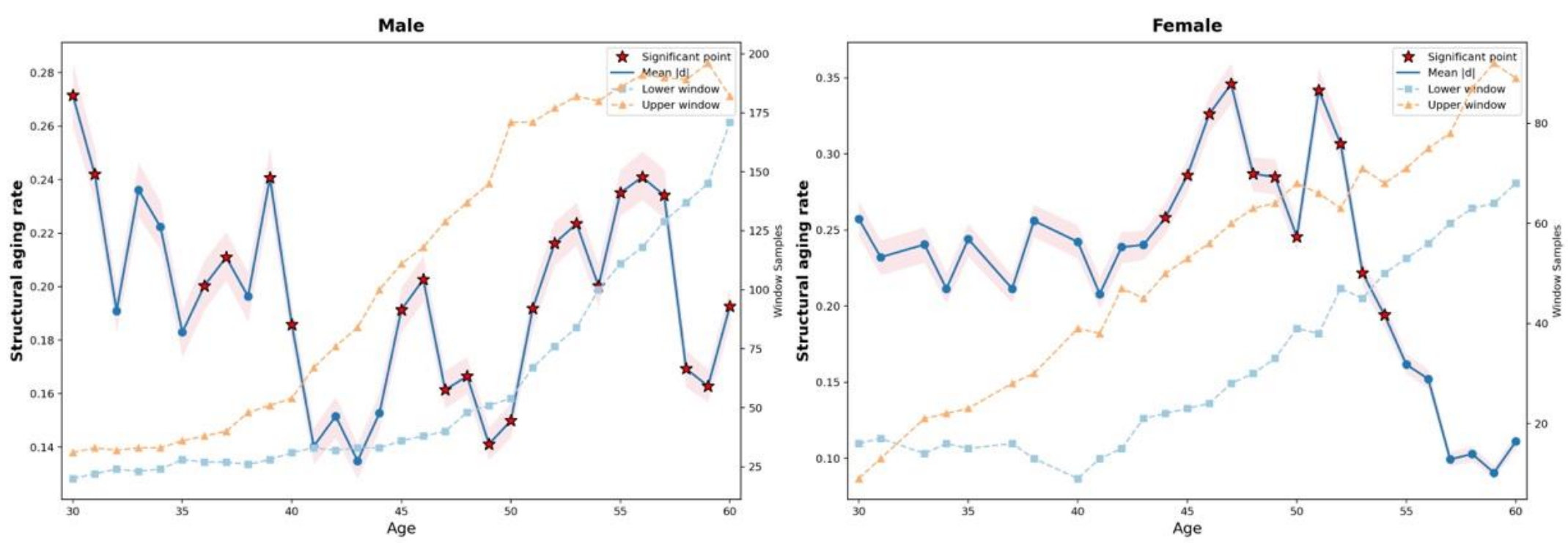

Brain - Cortex

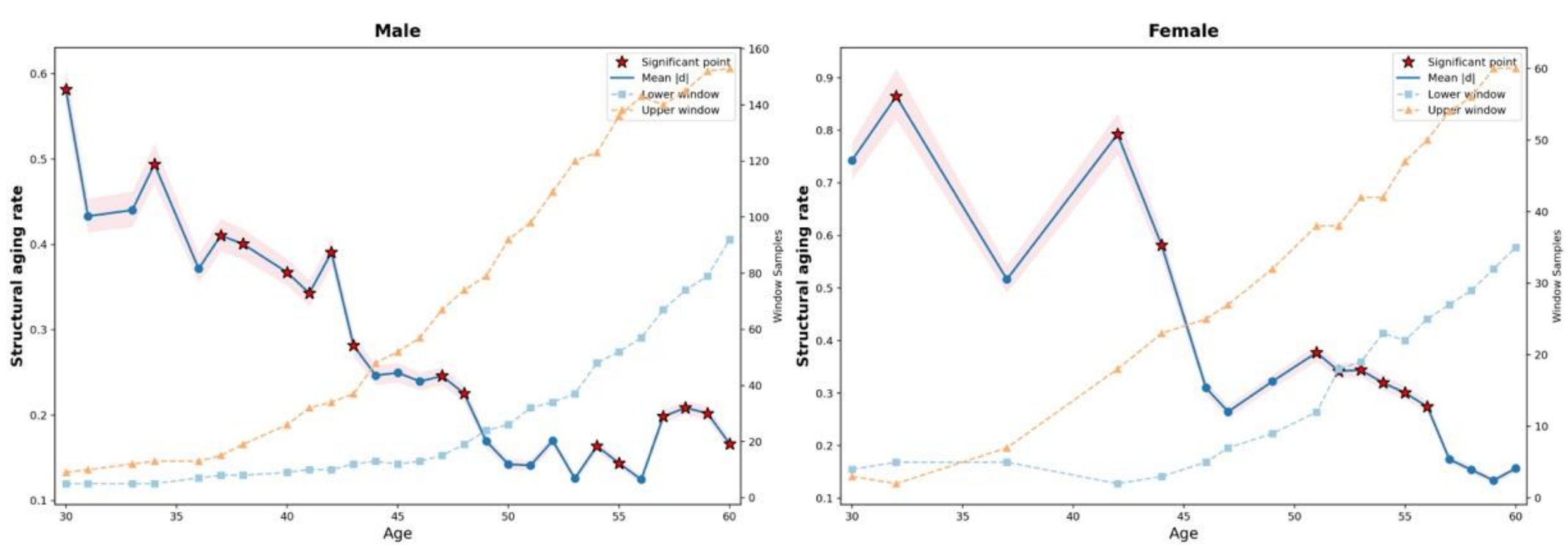

Brain - Cerebellum

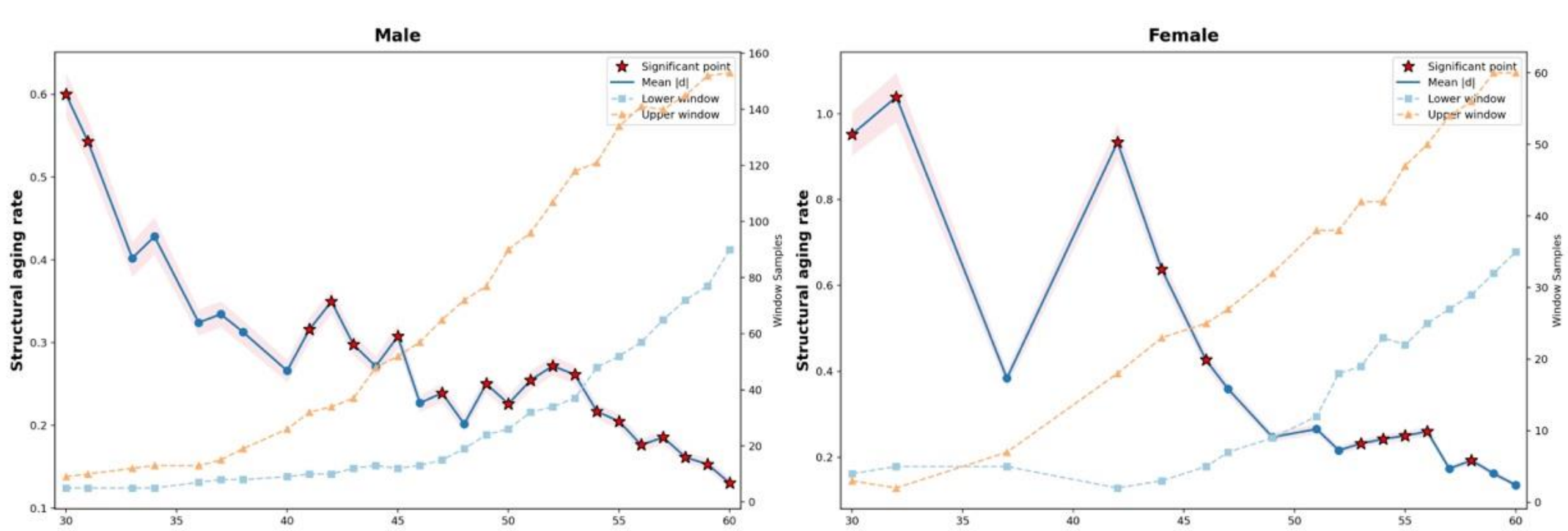

Breast - Mammary Tissue

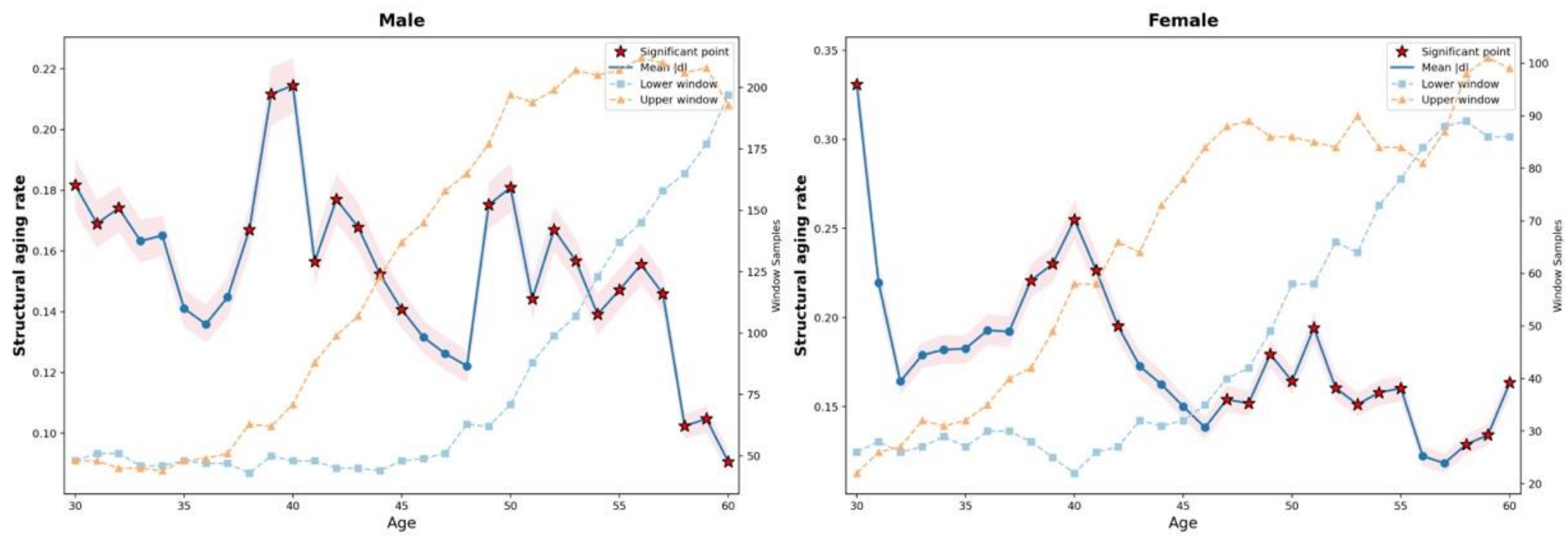

Esophagus - Mucosa

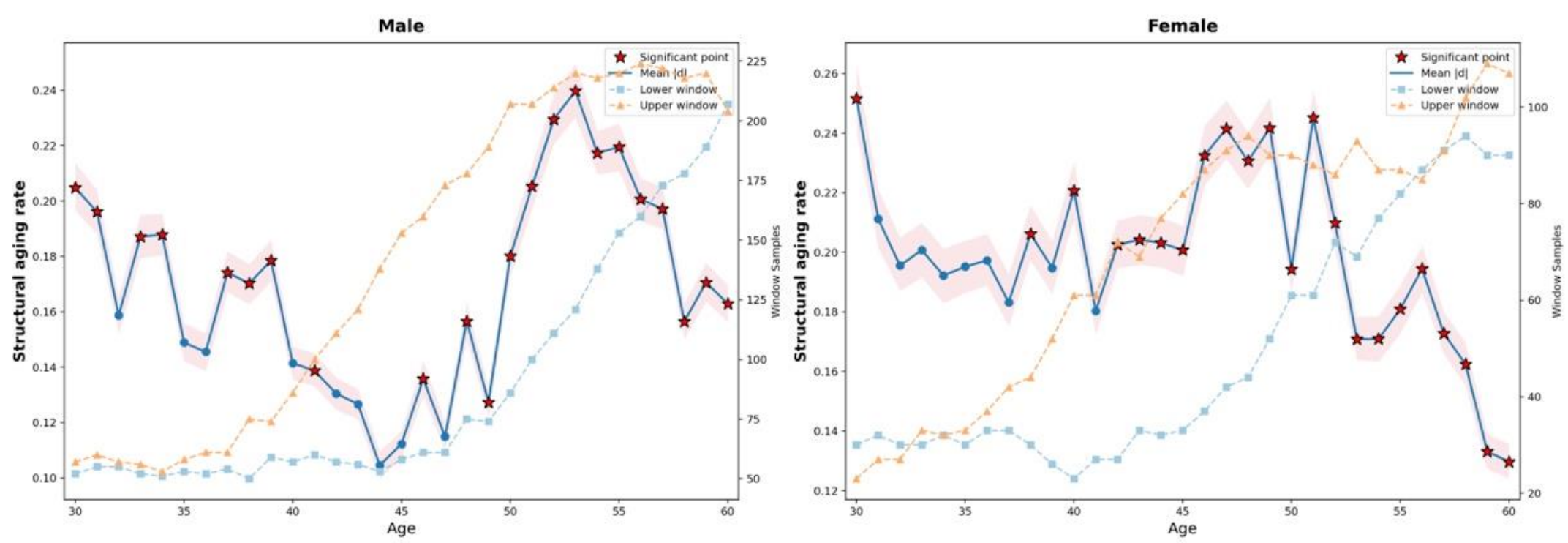

Heart - Atrial Appendage

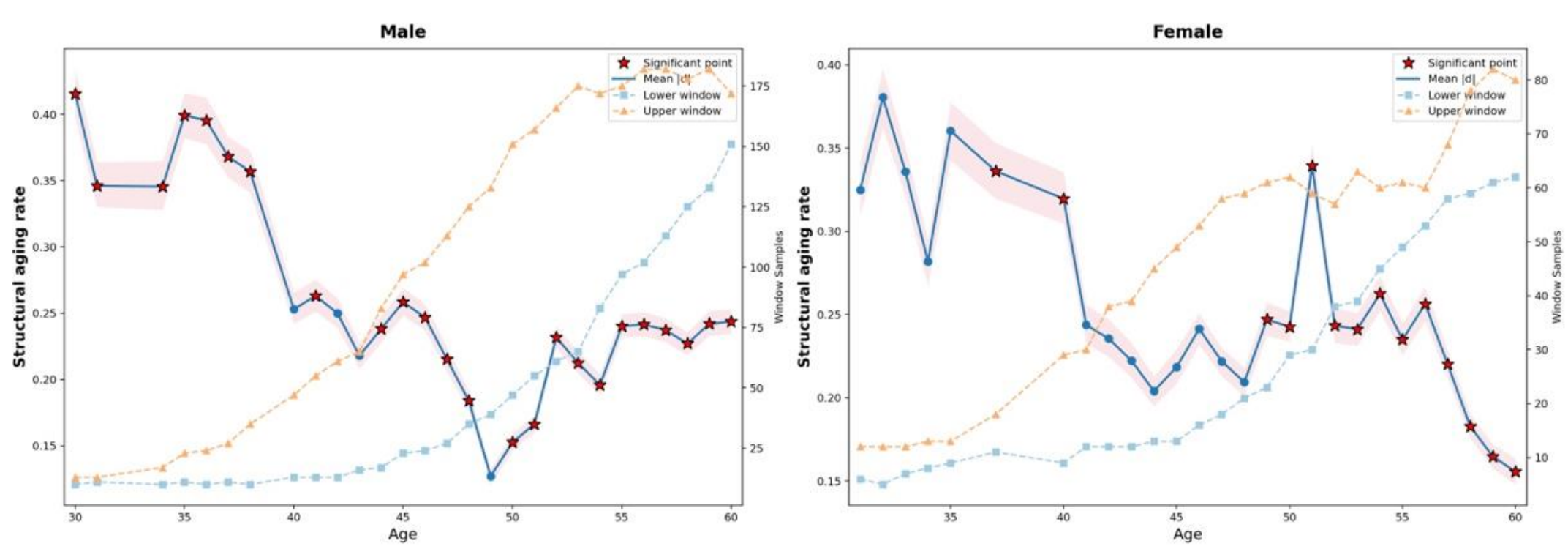

Heart - Left Ventricle

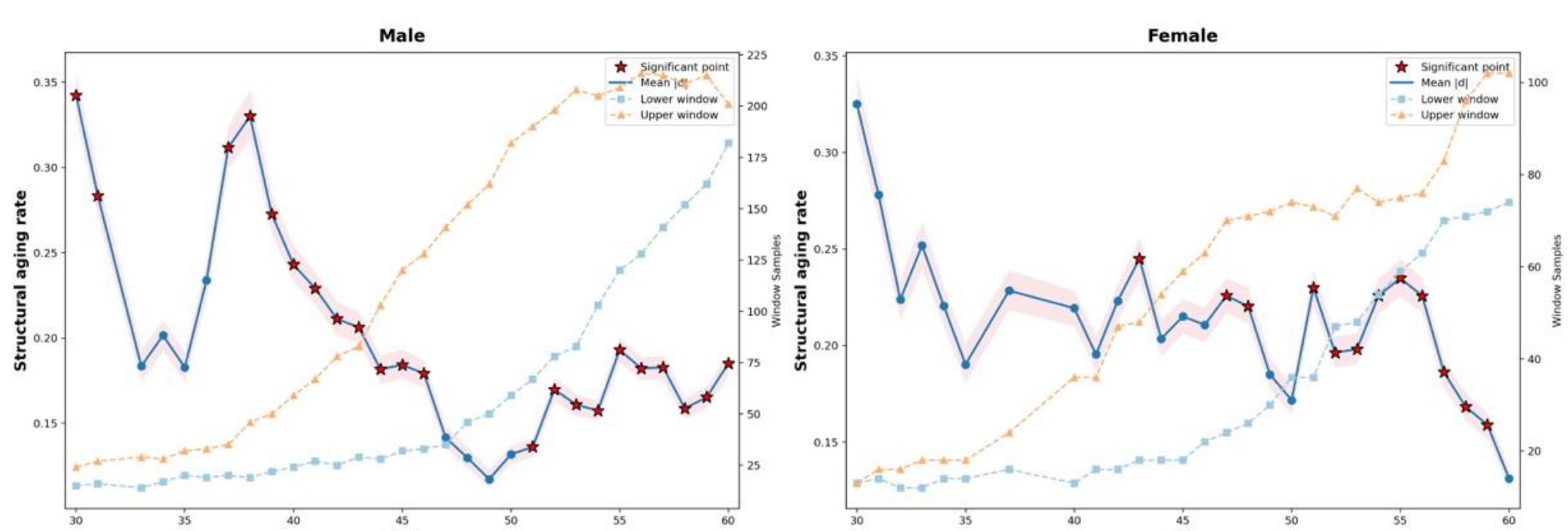

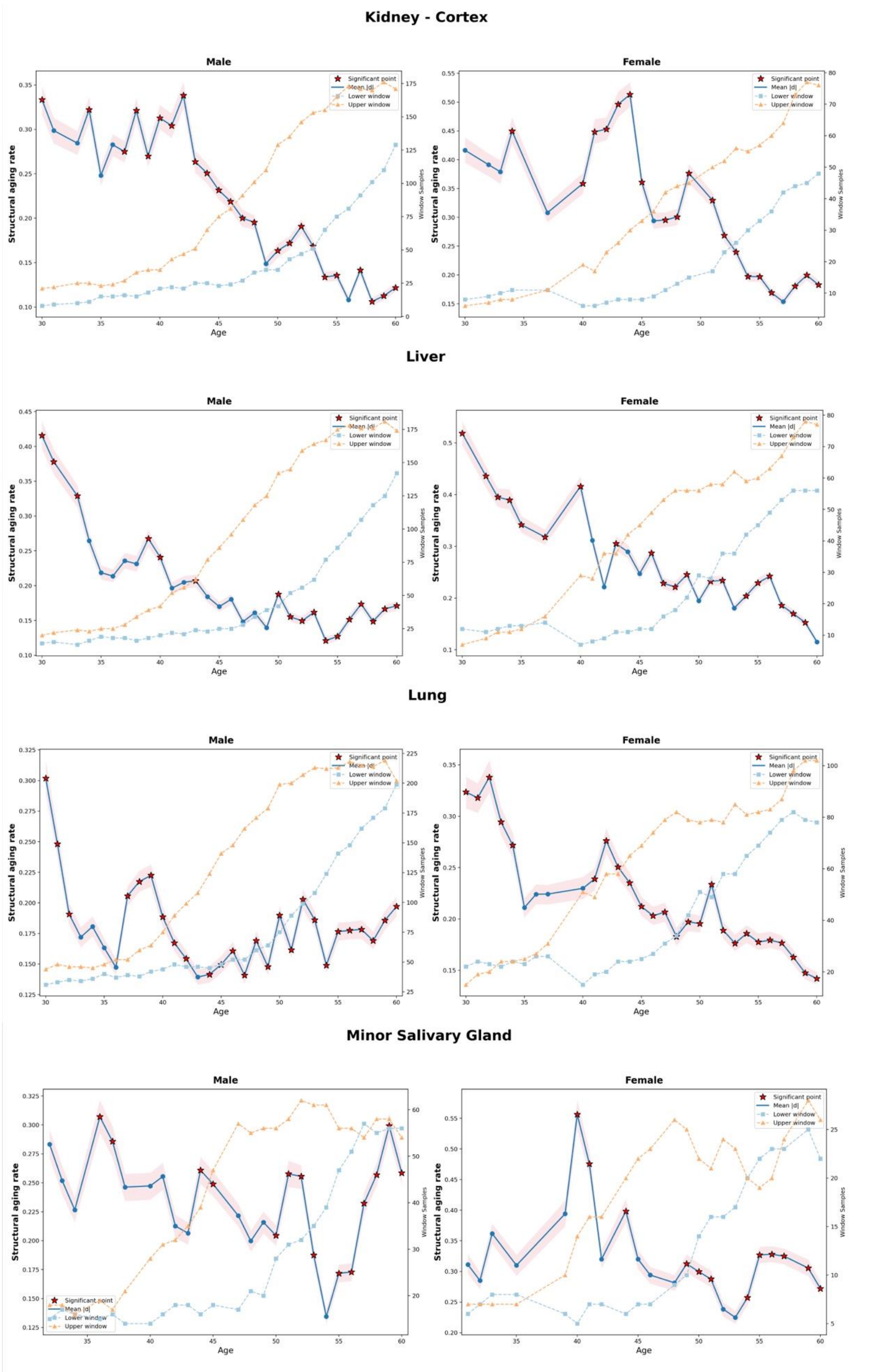
Kidney - Cortex
Male
Female
Liver
Male
Female
Lung
Male
Female
Minor Salivary Gland
Male
Female
Significant point
Mean |d|
Lower window
Upper window
Structural aging rate
Window Samples
Age

Pancreas

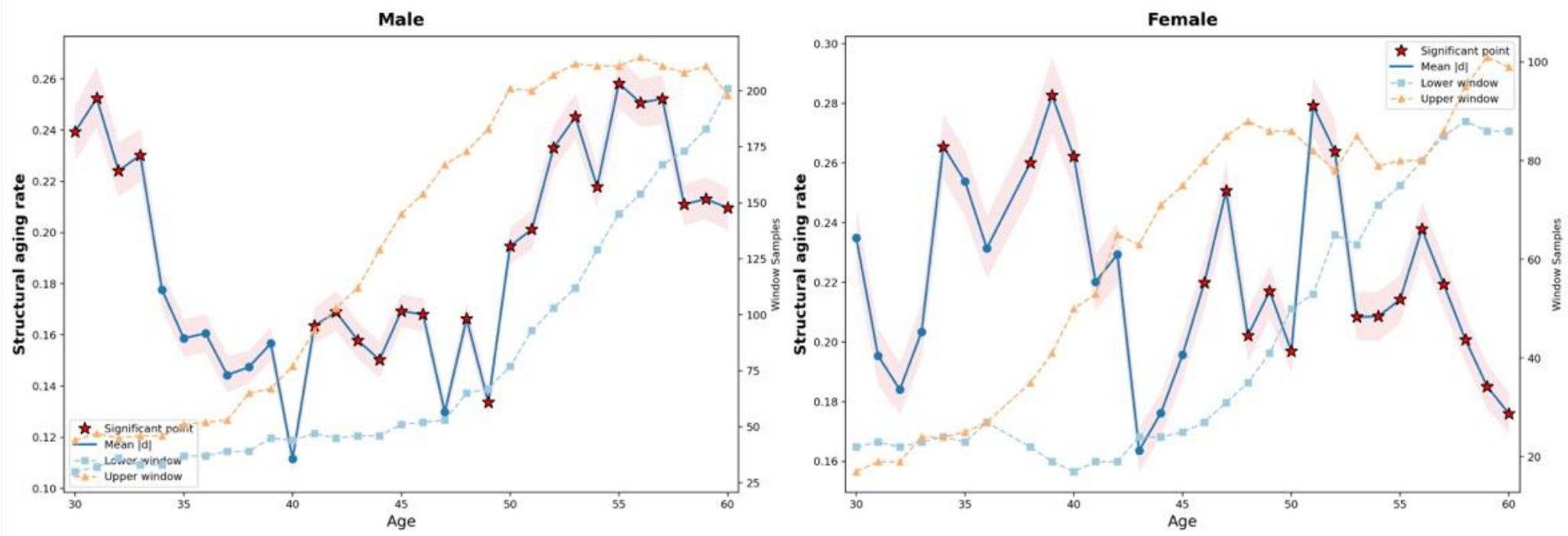

Pituitary

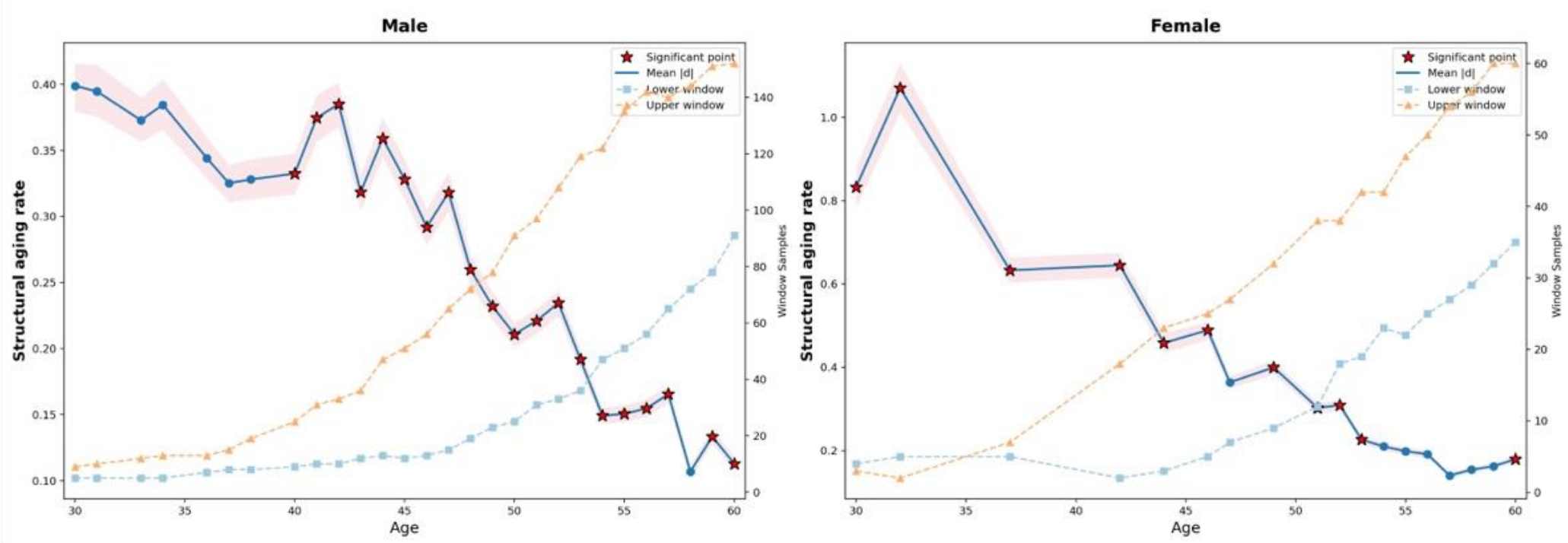

Skin - Sun Exposed (Lower leg)

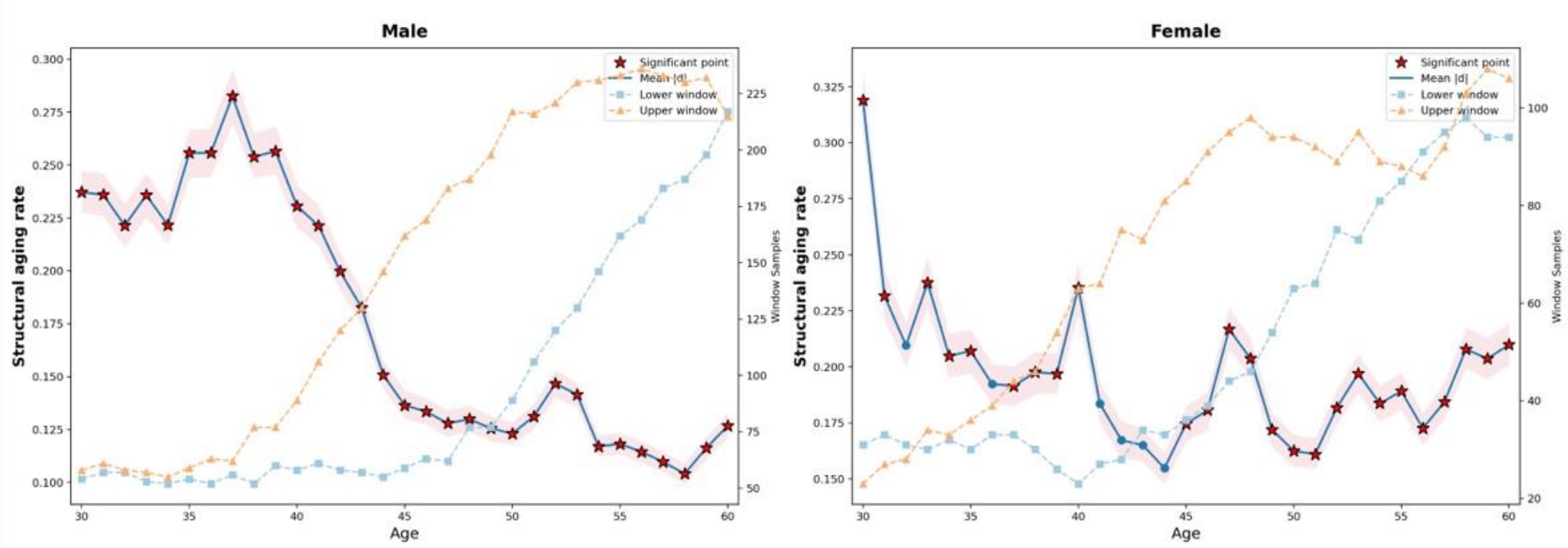

Skin - Not Sun Exposed (Suprapubic)

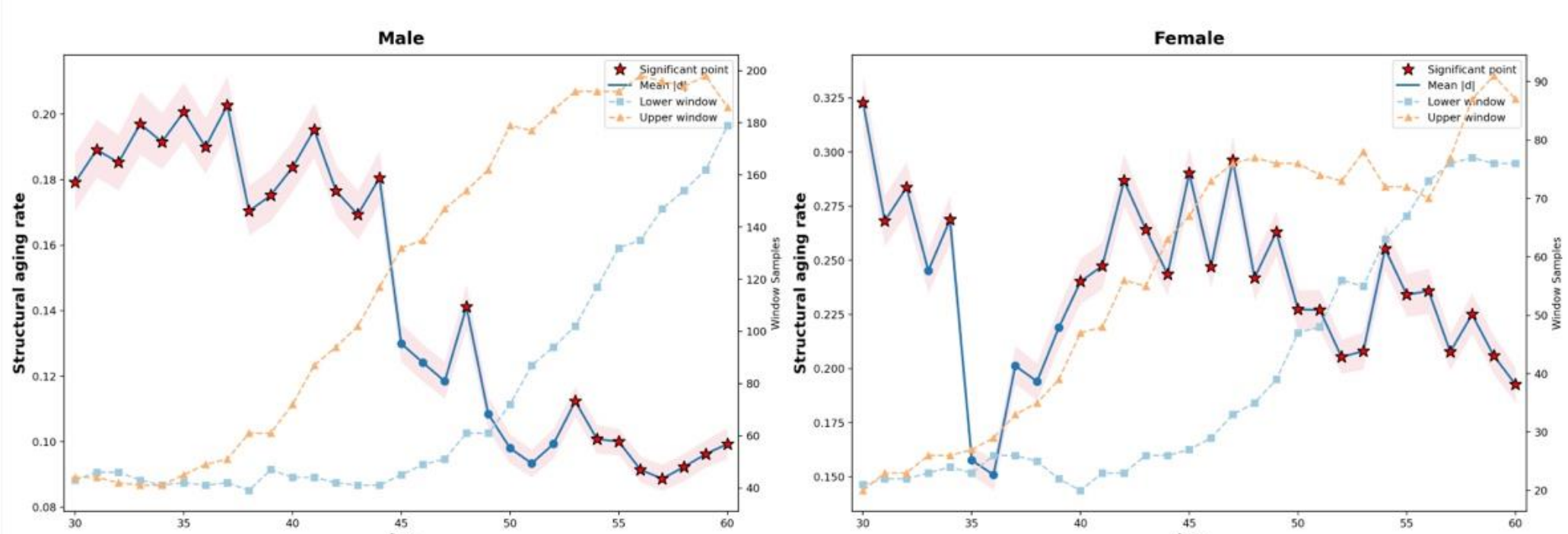

Supplementary Fig 19: Raw Structural Aging Analysis Across Additional Human Tissues with Sufficient Sample Size (n≥200) Stratified by Biological Sex: This figure presents the raw, unsmoothed Structural Aging patterns across 20 additional human tissue types that met the minimum sample size requirement (n≥200) for PathStAR analysis, analyzed separately for male and female samples using the methodology described in Method 5.4. The tissues examined represent diverse organ systems including adipose tissue (Adipose - Subcutaneous, Adipose - Visceral [Omentum]), endocrine organs (Adrenal Gland, Pancreas, Pituitary, Thyroid), vascular structures (Artery - Aorta), central nervous system (Brain - Cerebellum, Brain - Cortex), mammary tissue (Breast - Mammary Tissue), gastrointestinal components (Esophagus - Mucosa, Liver), cardiovascular system (Heart - Atrial Appendage, Heart - Left Ventricle), renal tissue (Kidney - Cortex), respiratory system (Lung), musculoskeletal system (Muscle - Skeletal), integumentary system (Skin - Not Sun Exposed [Suprapubic], Skin - Sun Exposed [Lower leg]), and lymphoid tissue (Spleen).(See Method 5.3)

Adipose - Subcutaneous

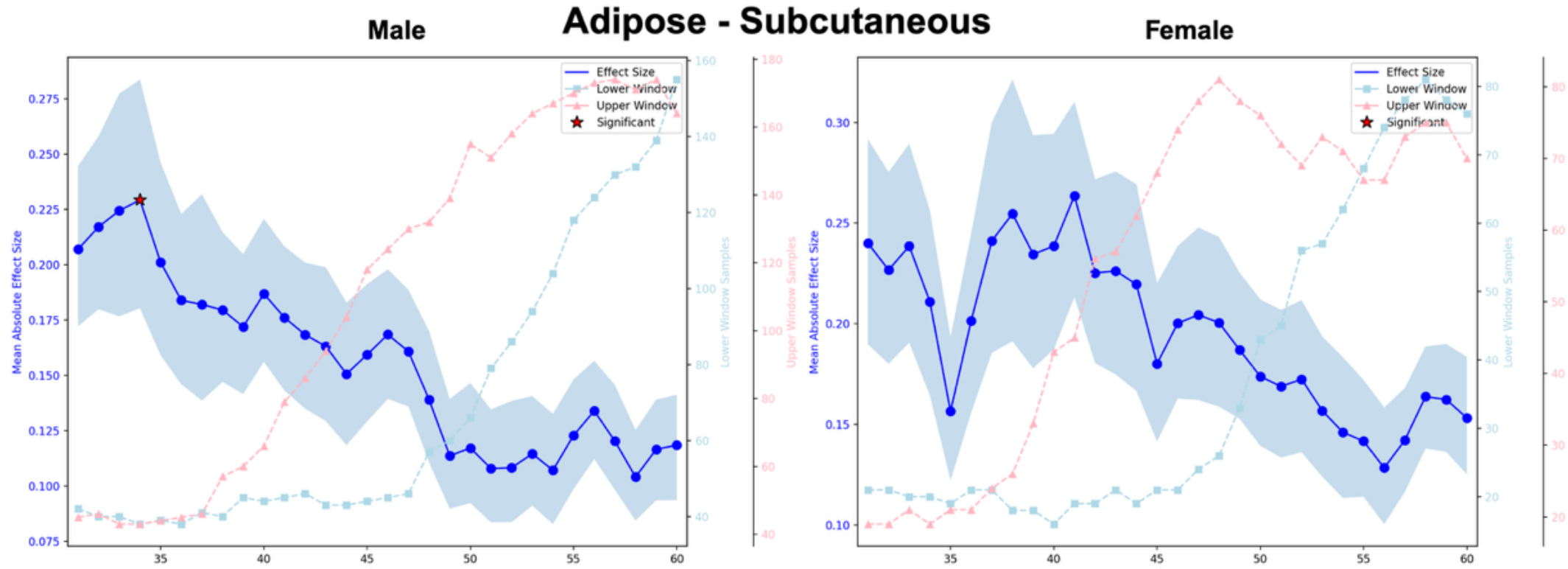

* indicates statistically significant changes (p < 0.05)

Adipose - Visceral (Omentum)

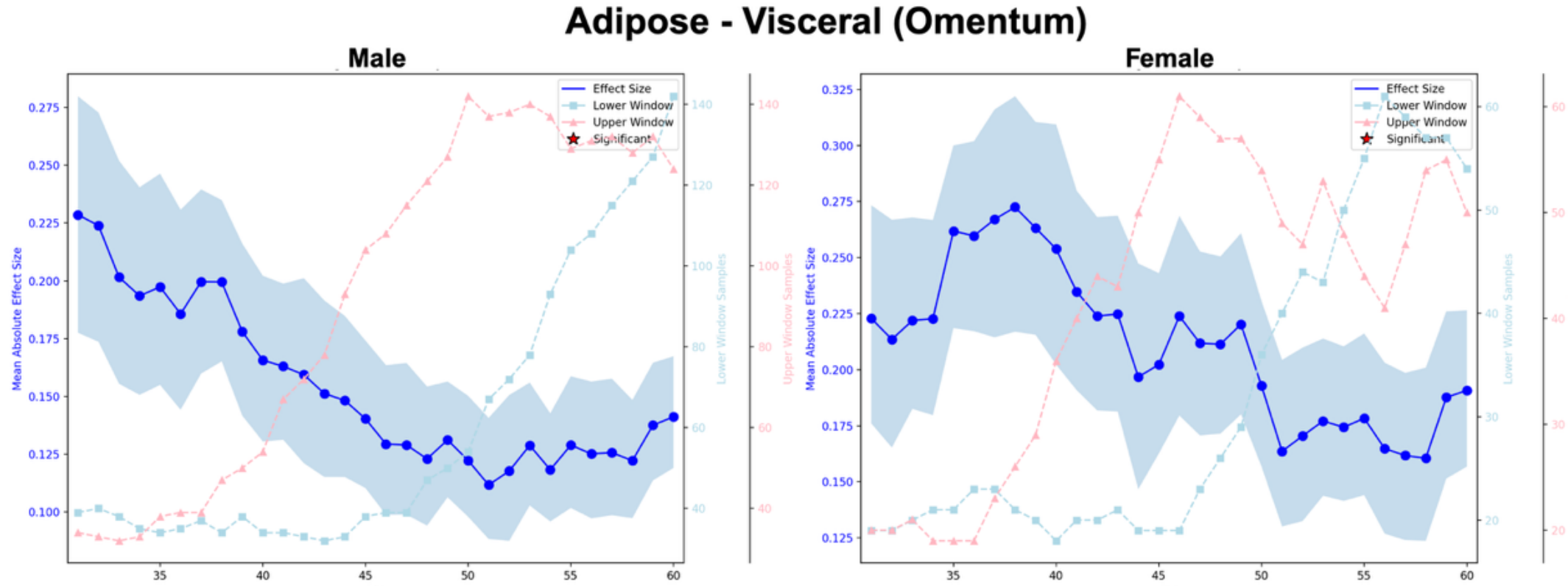

* indicates statistically significant changes (p < 0.05)

Adrenal Gland

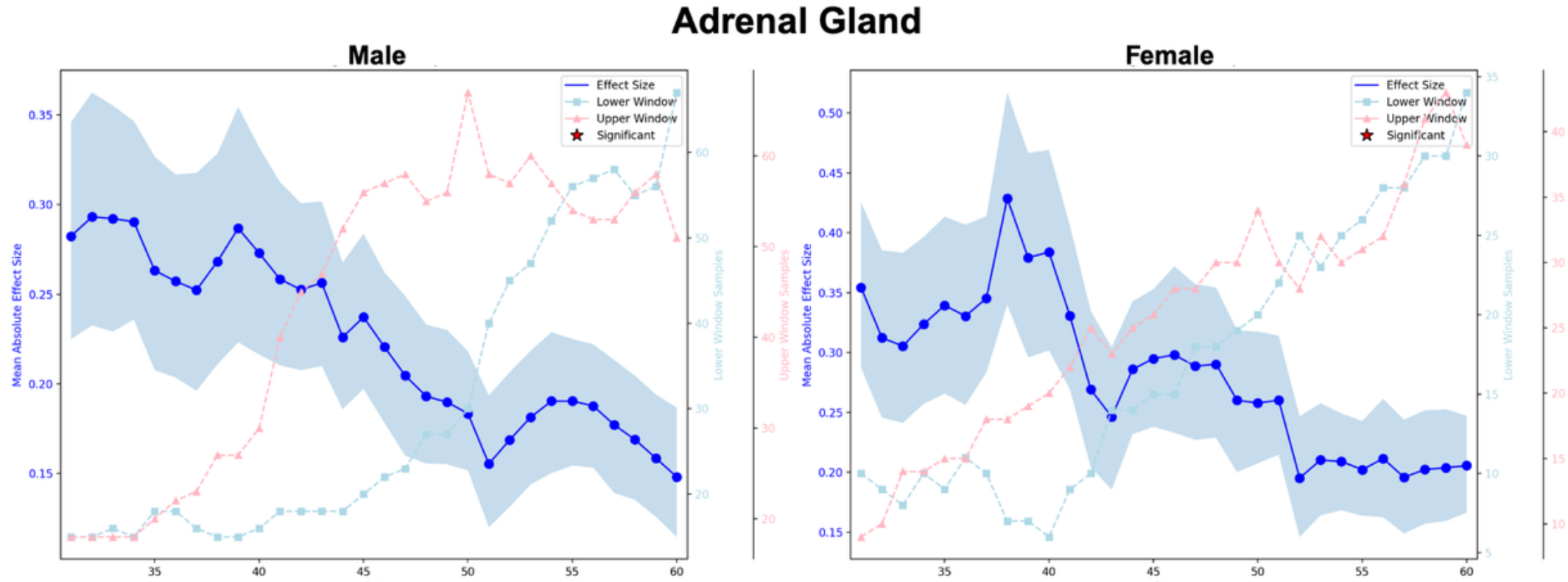

* indicates statistically significant changes (p < 0.05)

Artery - Aorta

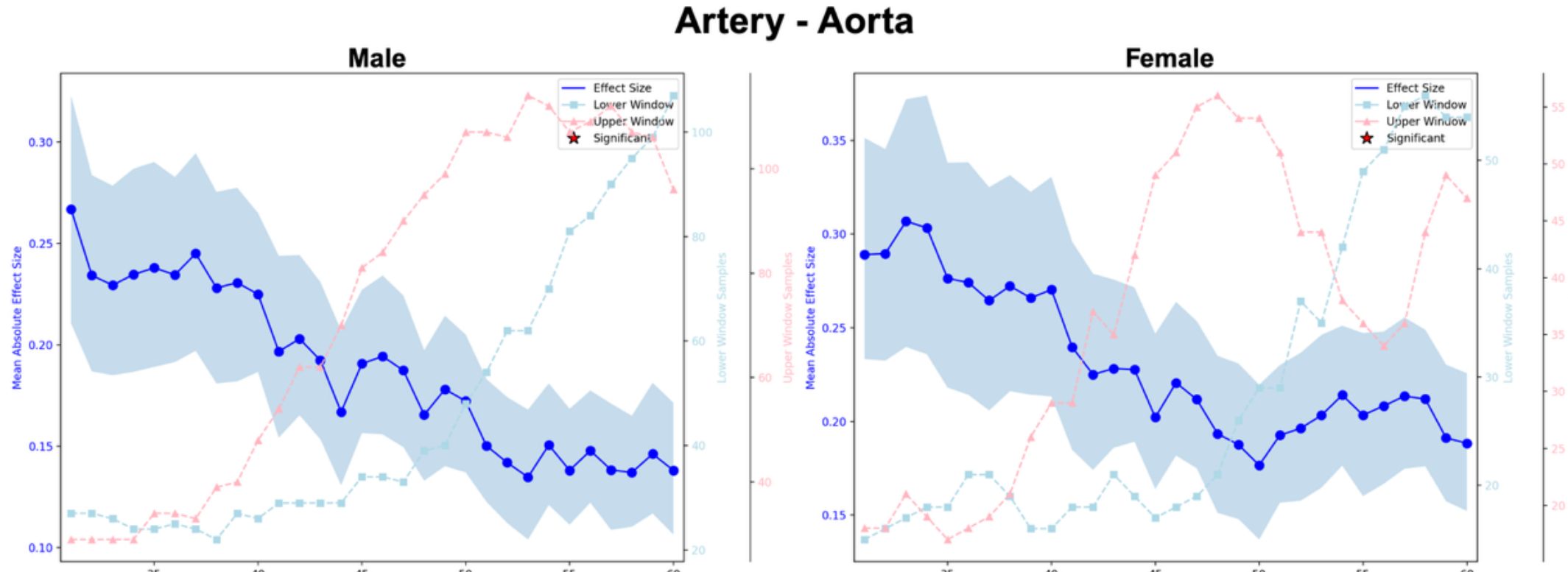

* indicates statistically significant changes (p < 0.05)

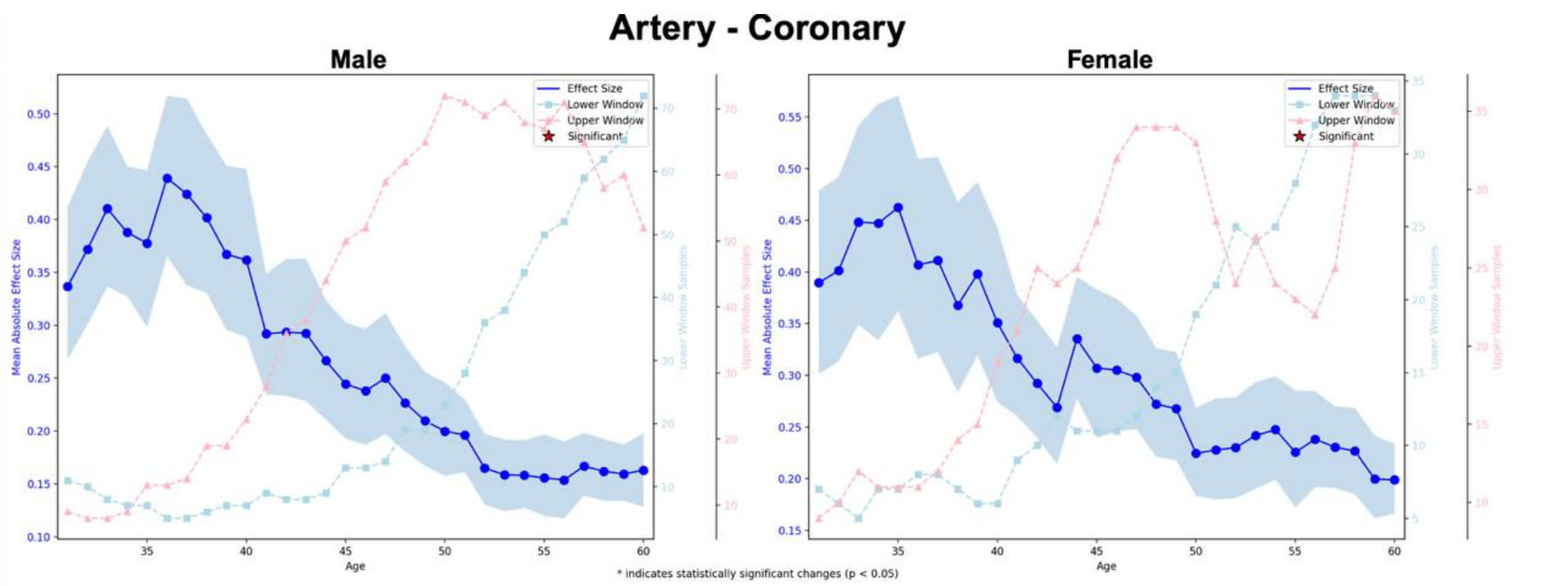
Artery - Coronary
Male
Female
Effect Size
Lower Window
Upper Window
Significant
Mean Absolute Effect Size
Lower Window Samples
Upper Window Samples
Age
* indicates statistically significant changes (p < 0.05)

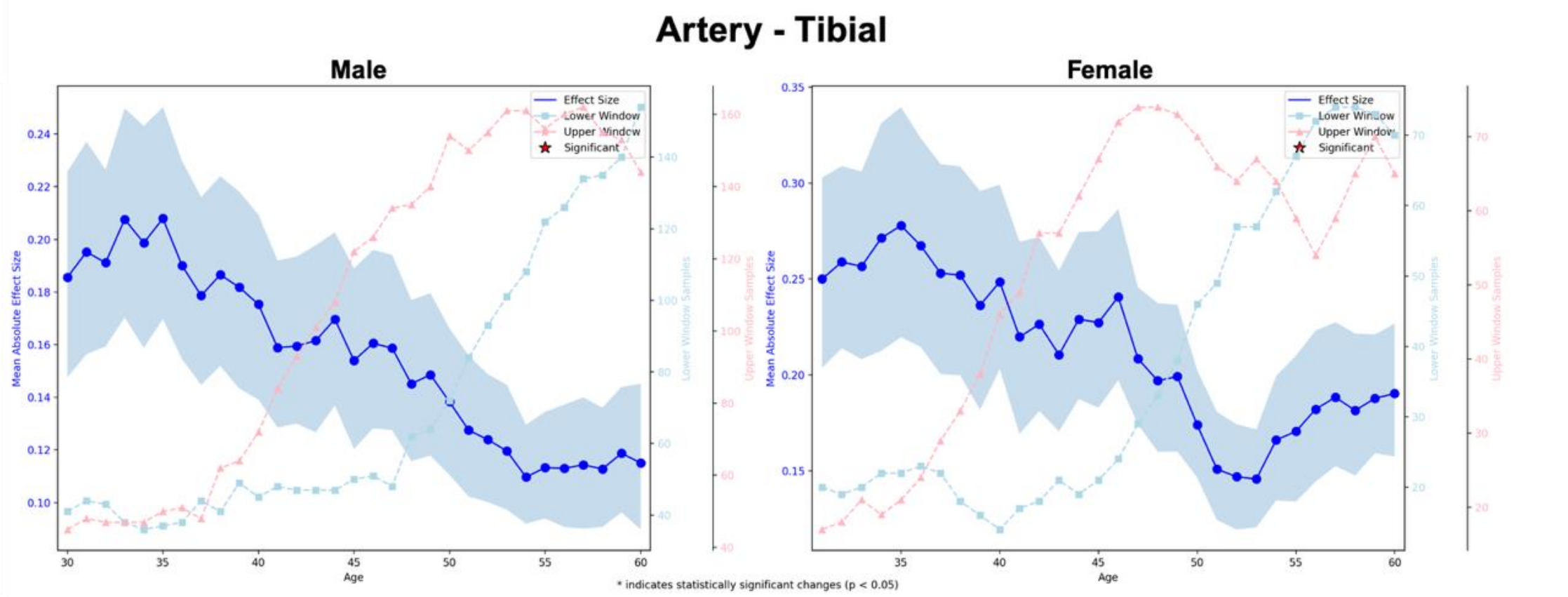
Artery - Tibial
Male
Female
Effect Size
Lower Window
Upper Window
Significant
Mean Absolute Effect Size
Lower Window Samples
Upper Window Samples
Age
* indicates statistically significant changes (p < 0.05)

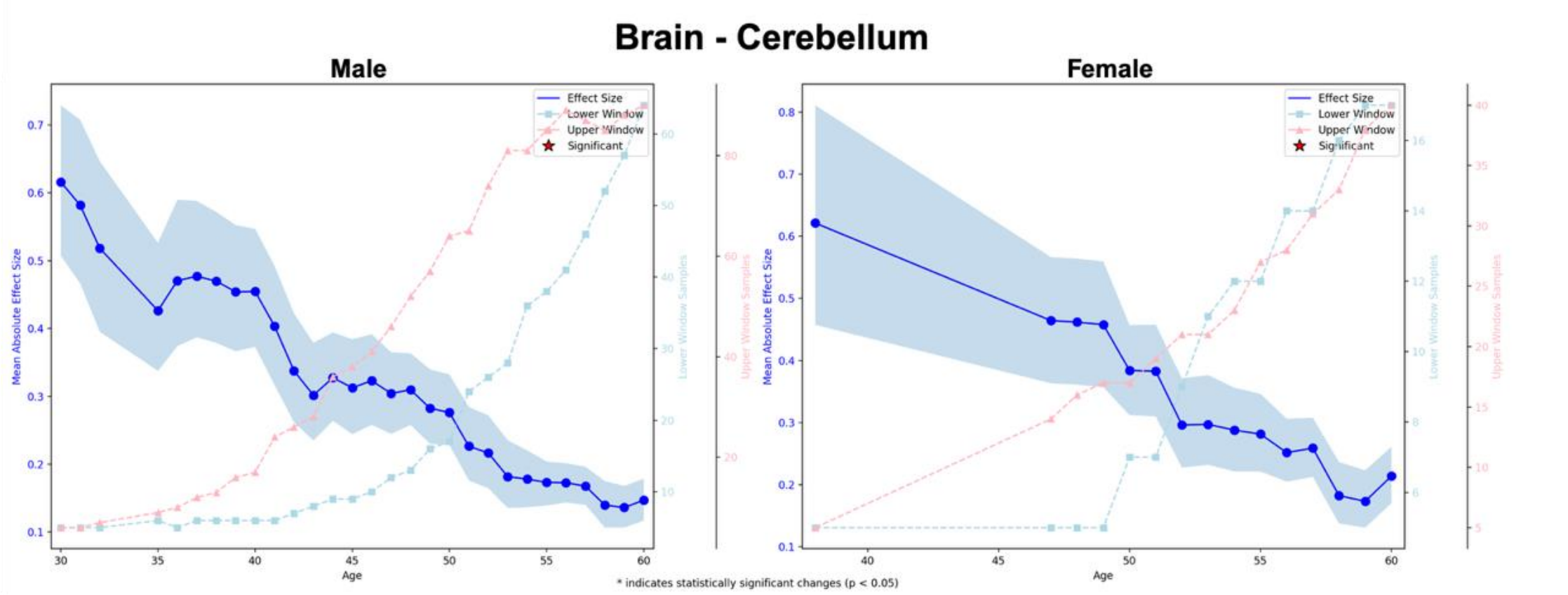
Brain - Cerebellum
Male
Female
Effect Size
Lower Window
Upper Window
Significant
Mean Absolute Effect Size
Lower Window Samples
Upper Window Samples
Age
* indicates statistically significant changes (p < 0.05)

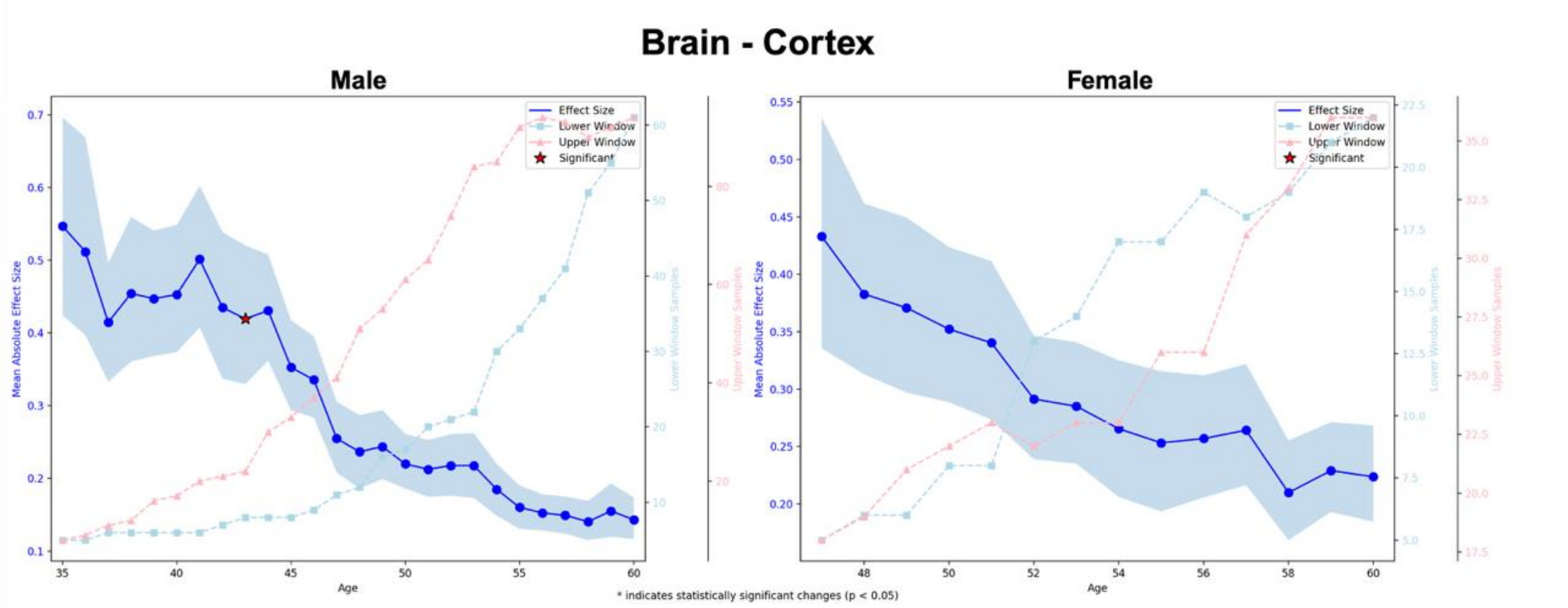
Brain - Cortex
Male
Female
Effect Size
Lower Window
Upper Window
Significant
Mean Absolute Effect Size
Lower Window Samples
Upper Window Samples
Age
* indicates statistically significant changes (p < 0.05)

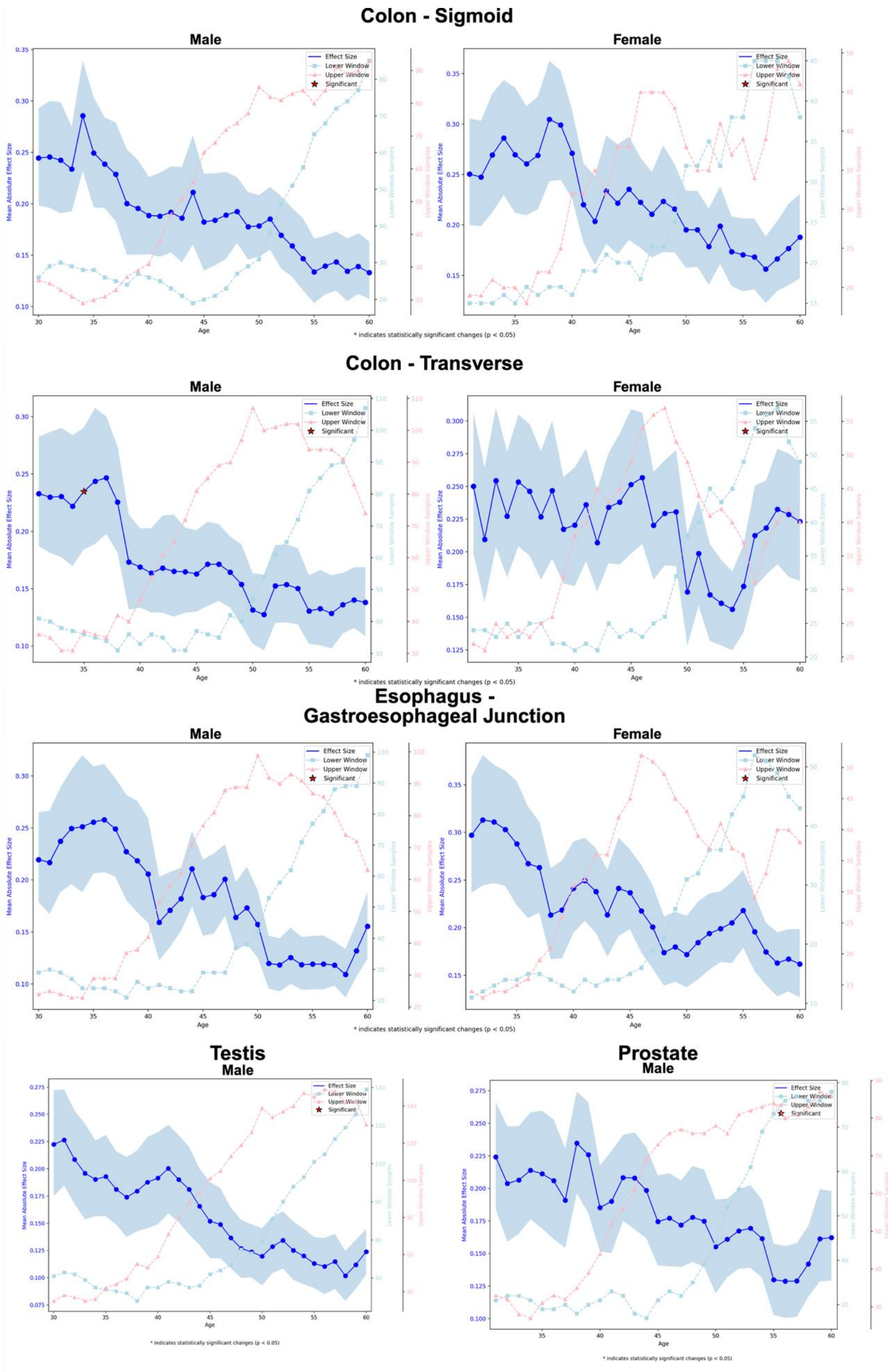
Colon - Sigmoid
Male
Female
Effect Size
Lower Window
Upper Window
Significant
Mean Absolute Effect Size
Lower Window Samples
Upper Window Samples
Age
* indicates statistically significant changes (p < 0.05)
Colon - Transverse
Male
Female
Esophagus - Gastroesophageal Junction
Male
Female
Testis
Male
Prostate
Male

Esophagus - Mucosa

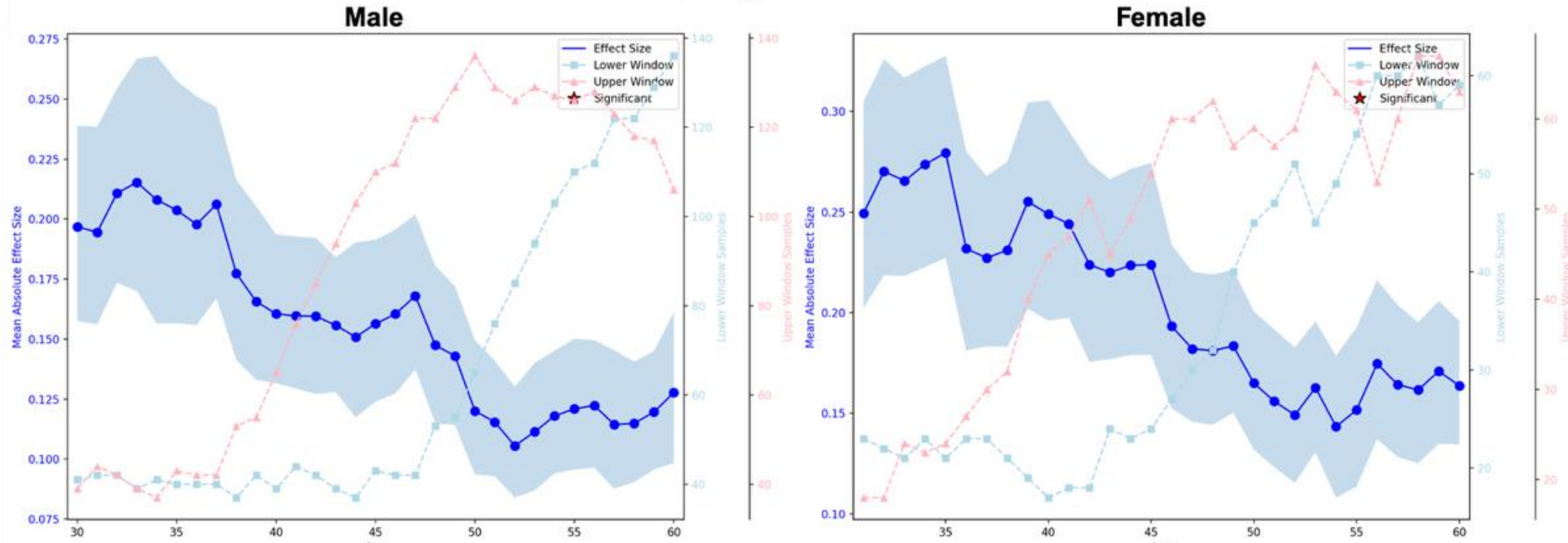

* indicates statistically significant changes (p < 0.05)

Esophagus - Muscularis

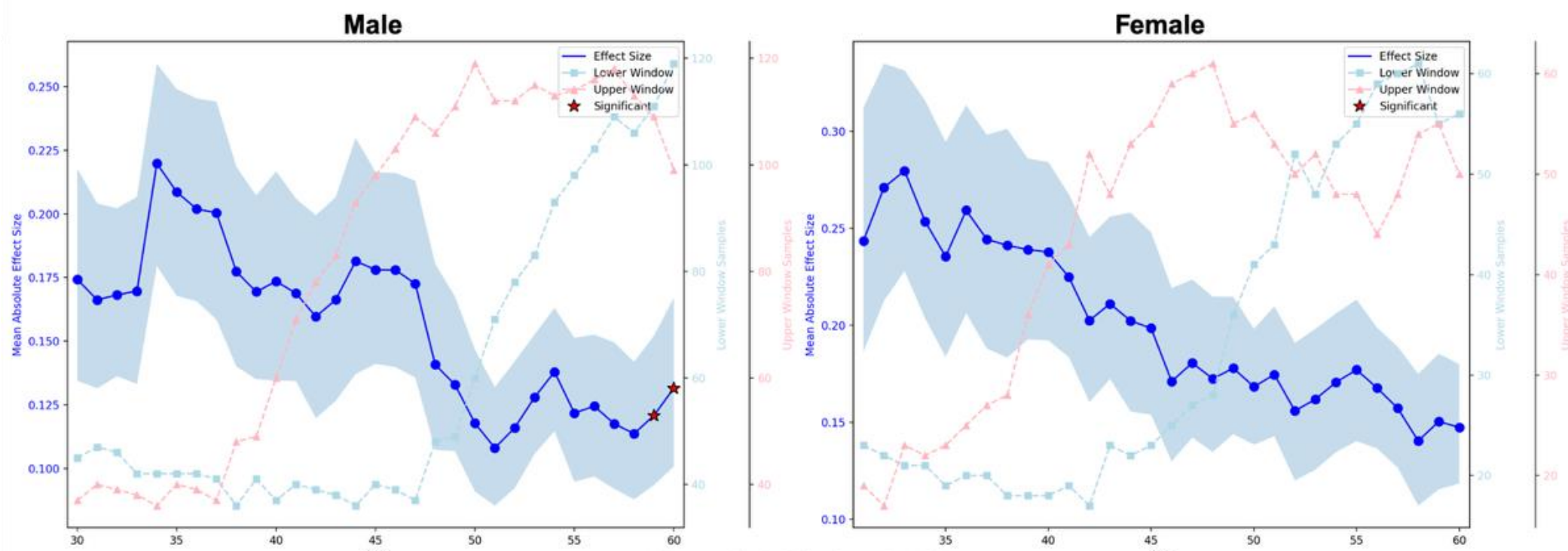

* indicates statistically significant changes (p < 0.05)

Heart - Atrial Appendage

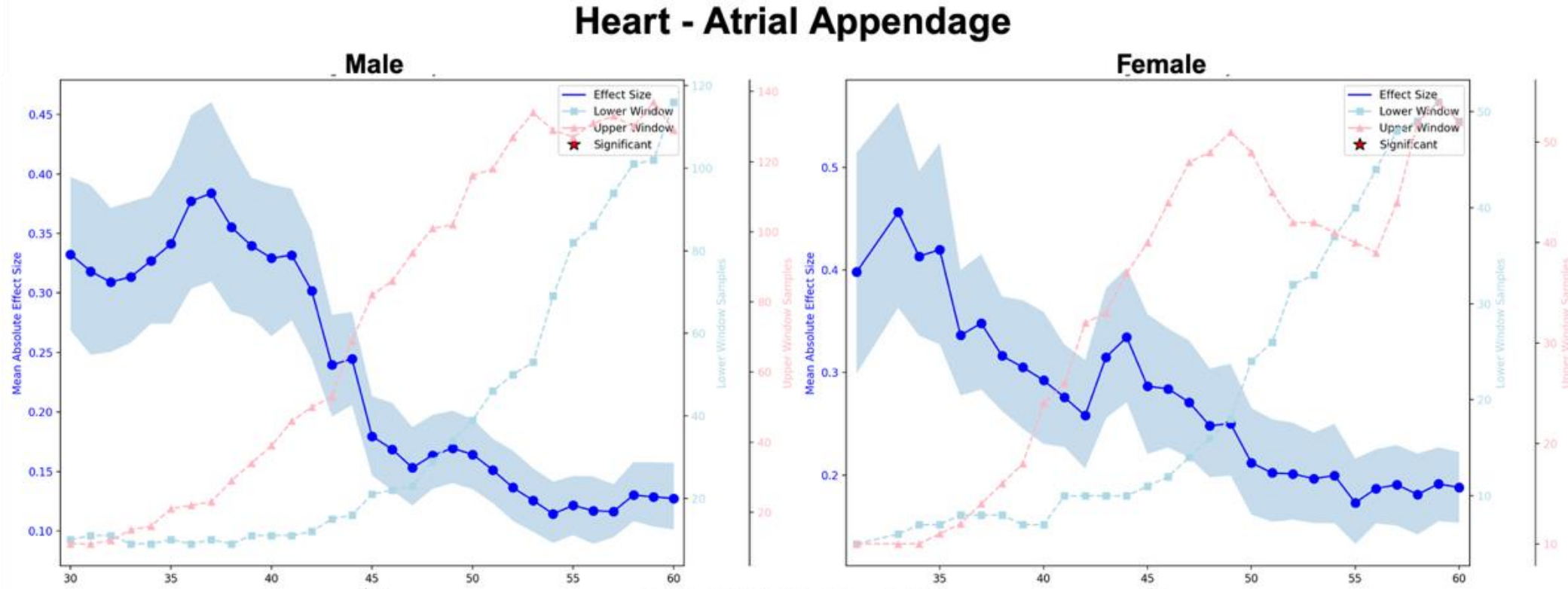

* indicates statistically significant changes (p < 0.05)

Heart - Left Ventricle

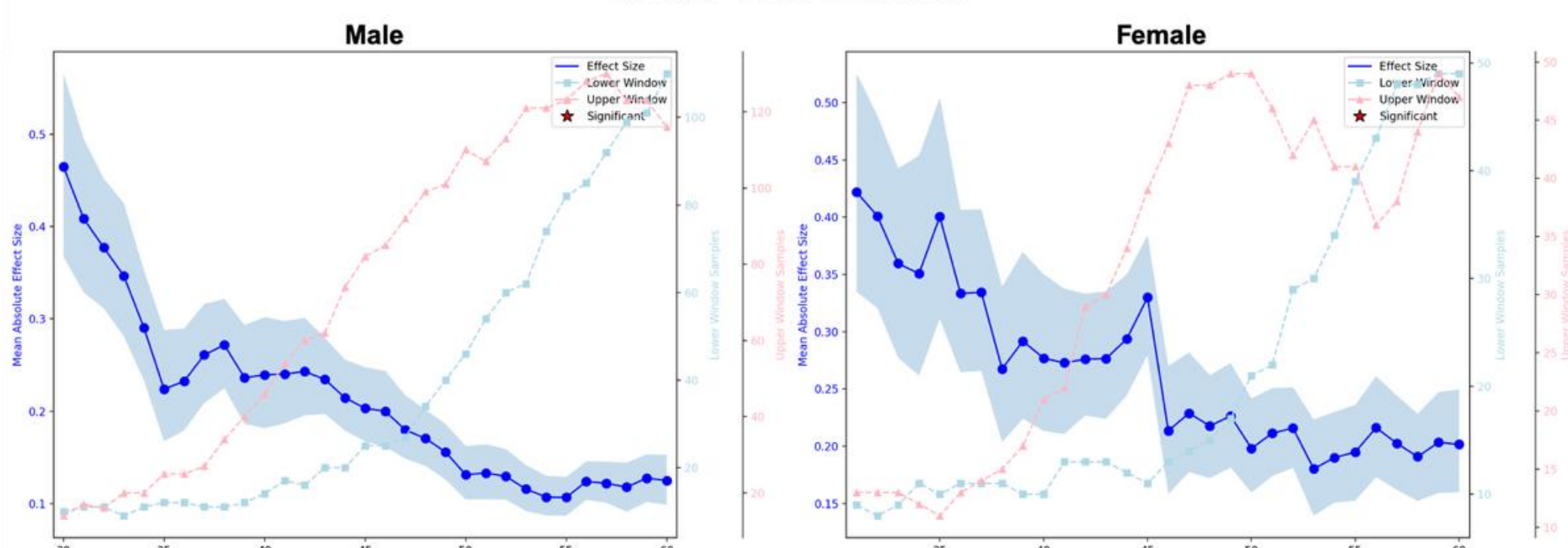

* indicates statistically significant changes (p < 0.05)

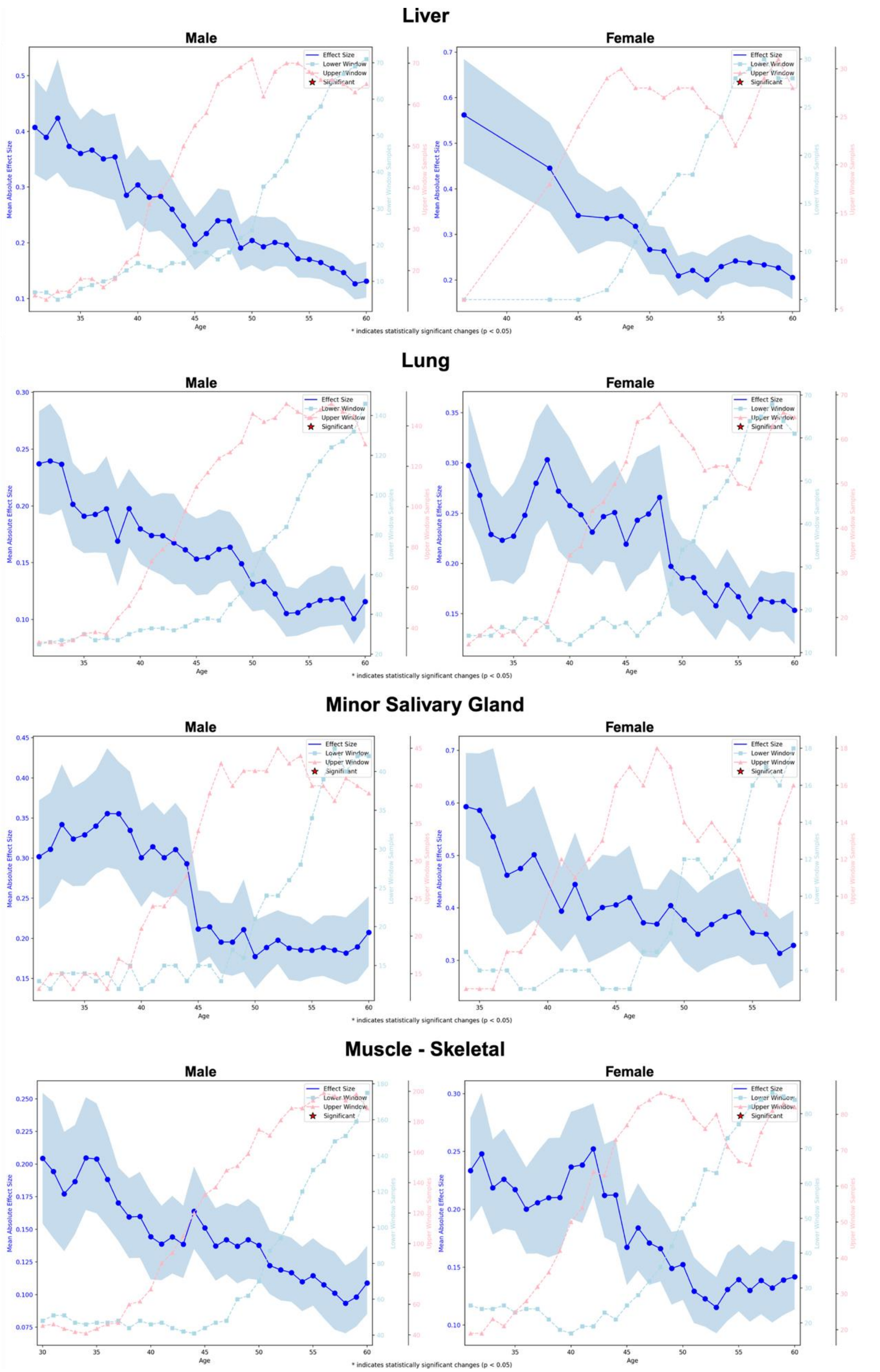
Liver
Male
Female
Lung
Male
Female
Minor Salivary Gland
Male
Female
Muscle - Skeletal
Male
Female
Effect Size
Lower Window
Upper Window
Significant
Mean Absolute Effect Size
Lower Window Samples
Upper Window Samples
Age
* indicates statistically significant changes (p < 0.05)

Nerve - Tibial

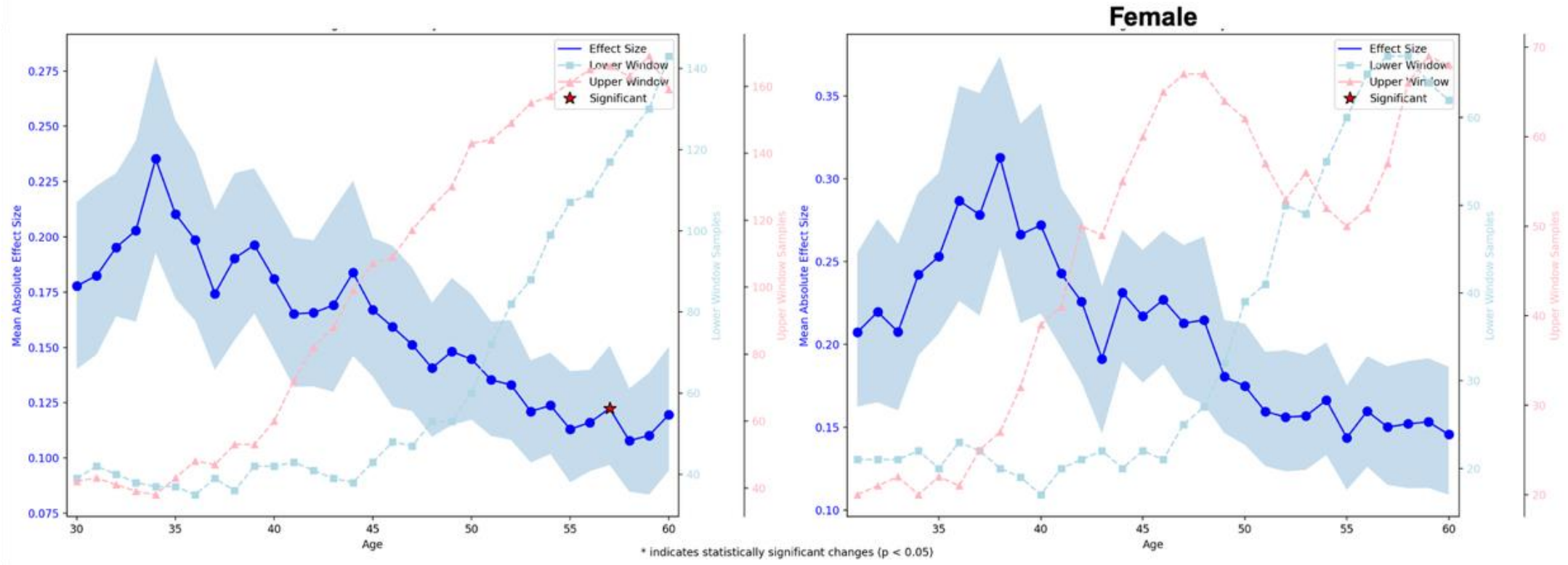

Pancreas

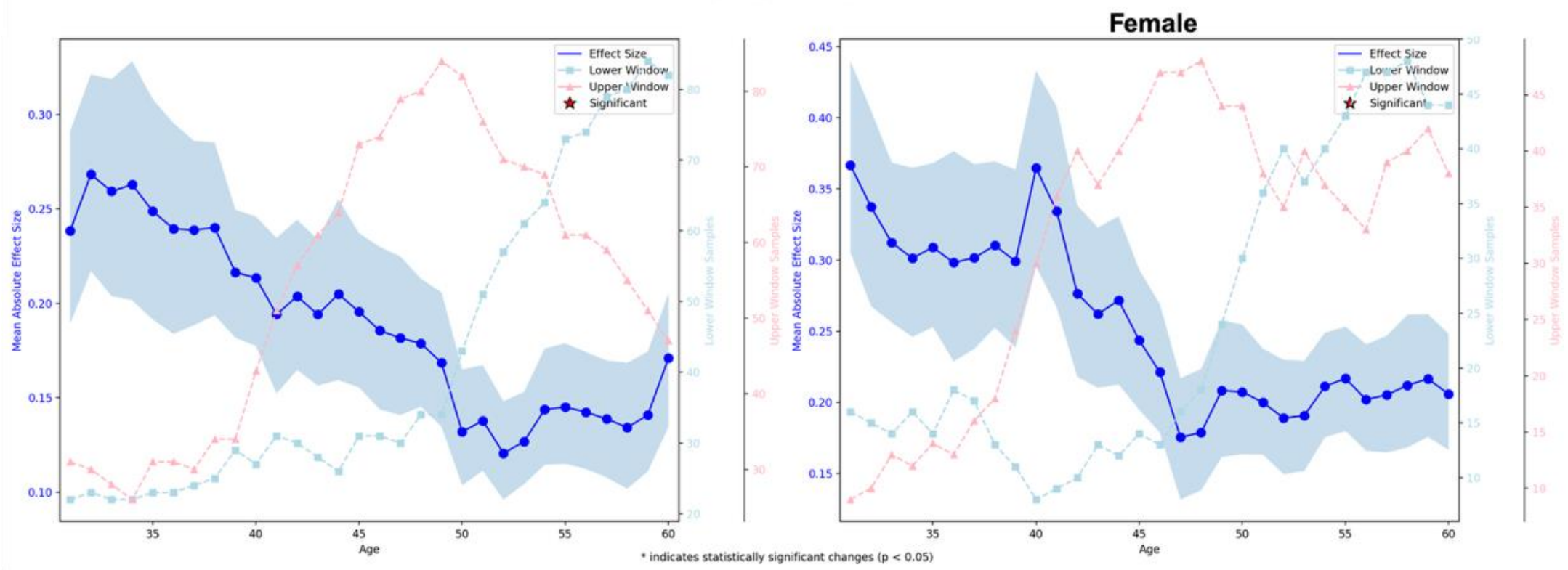

Pituitary

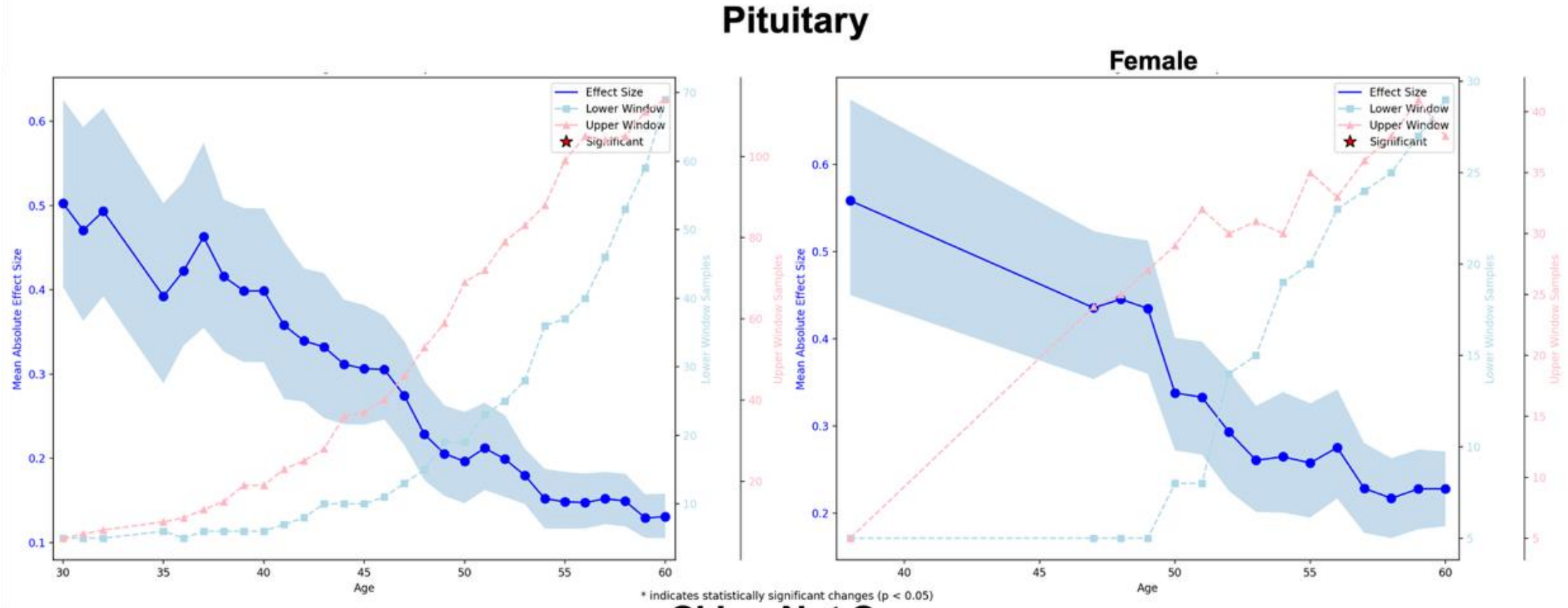

Skin - Not Sun Exposed (Suprapubic)

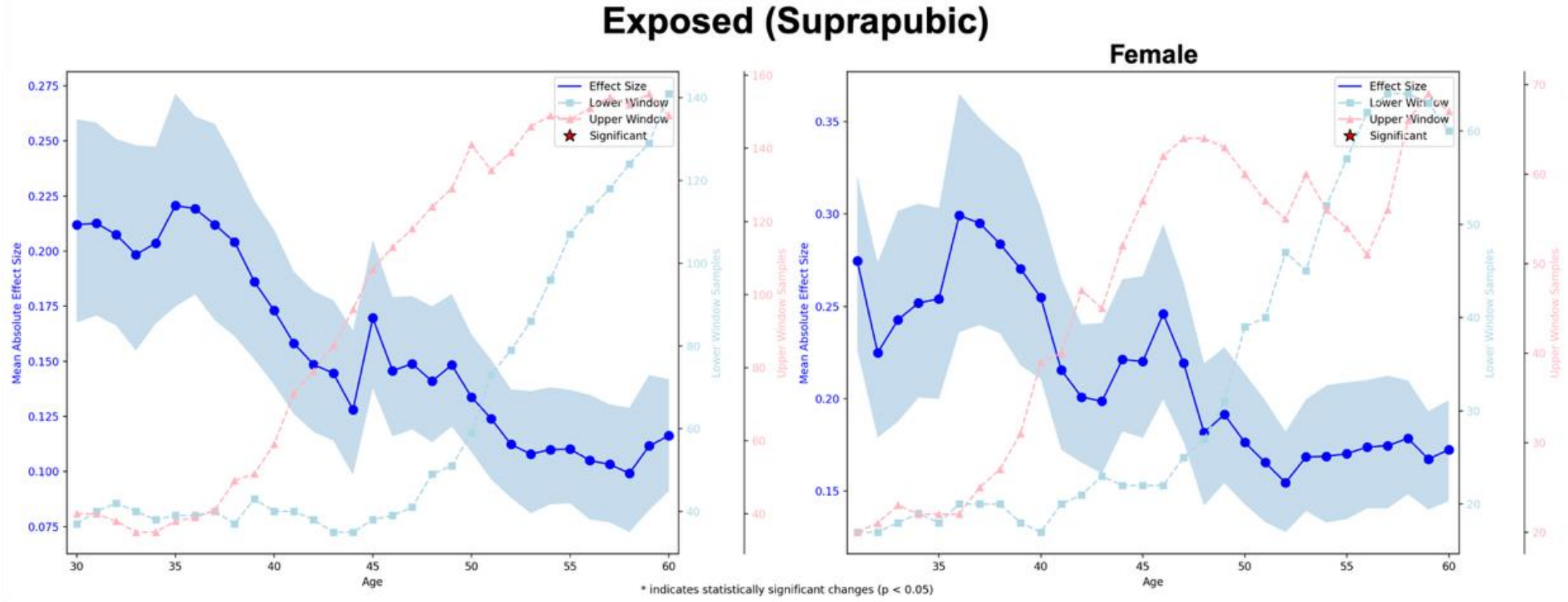

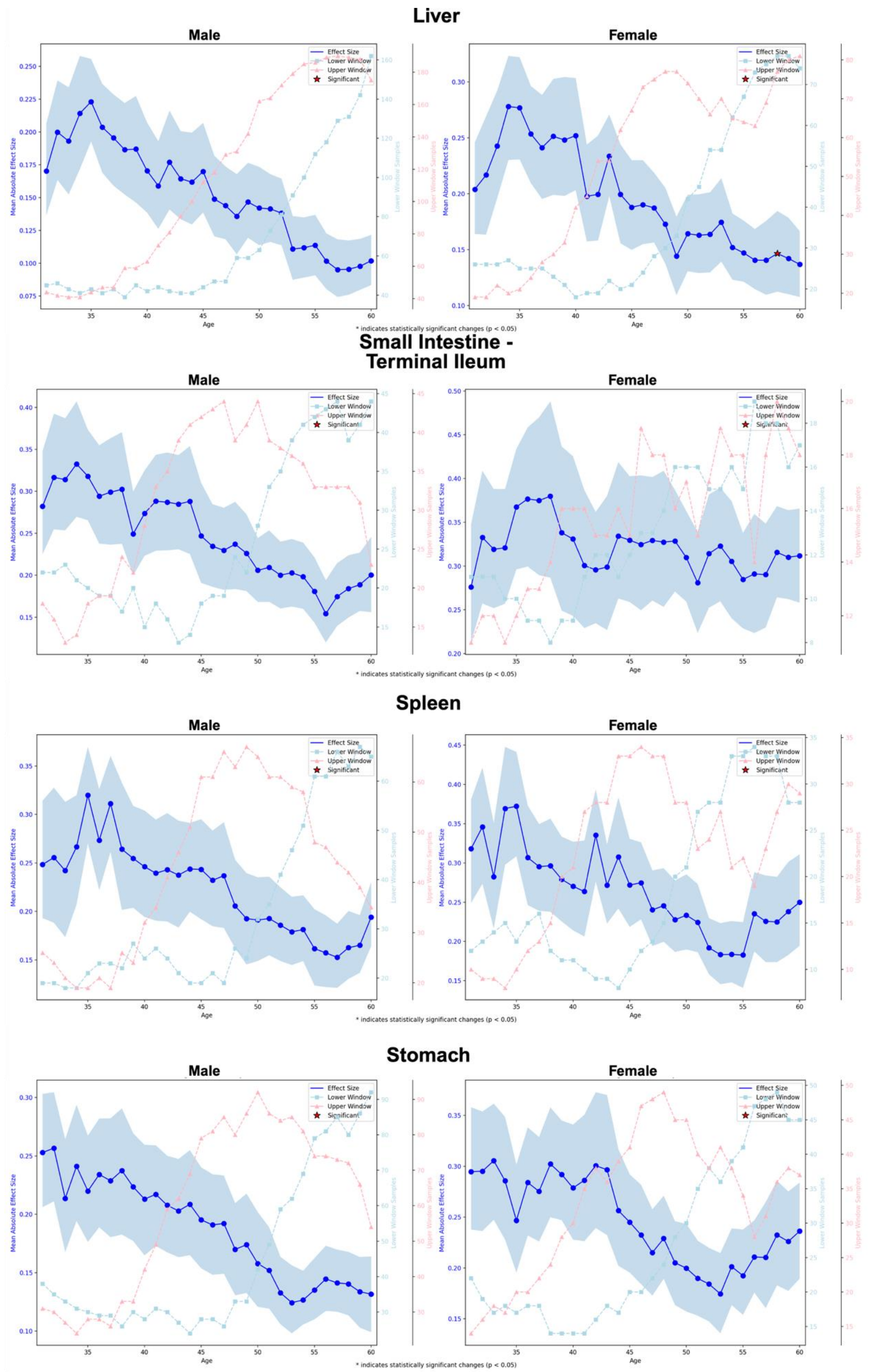
Liver
Male
Female
Effect Size
Lower Window
Upper Window
Significant
Mean Absolute Effect Size
Lower Window Samples
Upper Window Samples
Age
* indicates statistically significant changes (p < 0.05)
Small Intestine - Terminal Ileum
Male
Female
Effect Size
Lower Window
Upper Window
Significant
Mean Absolute Effect Size
Lower Window Samples
Upper Window Samples
Age
* indicates statistically significant changes (p < 0.05)
Spleen
Male
Female
Effect Size
Lower Window
Upper Window
Significant
Mean Absolute Effect Size
Lower Window Samples
Upper Window Samples
Age
* indicates statistically significant changes (p < 0.05)
Stomach
Male
Female
Effect Size
Lower Window
Upper Window
Significant
Mean Absolute Effect Size
Lower Window Samples
Upper Window Samples
Age
* indicates statistically significant changes (p < 0.05)

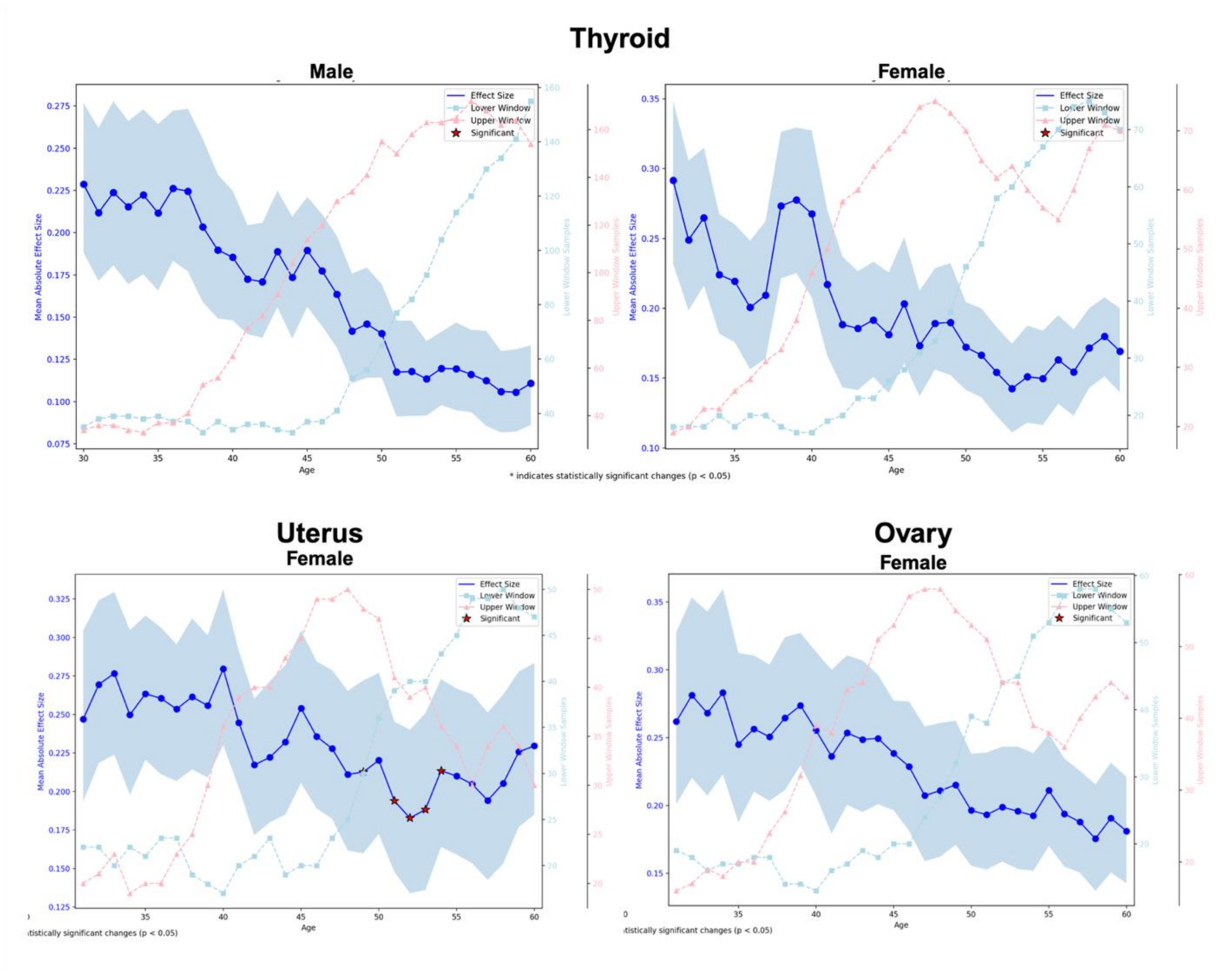

Supplementary Fig 20: Transcriptomic Aging Rate Trajectories Across Human Tissues Stratified by Biological Sex. Sliding window analysis applied to transcriptomic principal component features across 58 tissue-sex combinations (29 tissues with sufficient samples for both sexes, plus 4 sex-specific tissues) to quantify the rate of molecular change during aging. Gene expression data (TPMs) were log1p-transformed and subjected to tissue-specific PCA using the top 5,000 most variable genes reduced to 50 principal components. Using 10-year sliding windows across ages 30-79, the magnitude of change in transcriptomic PC space between consecutive age periods was computed to derive tissue-specific and sex-specific aging rate trajectories.

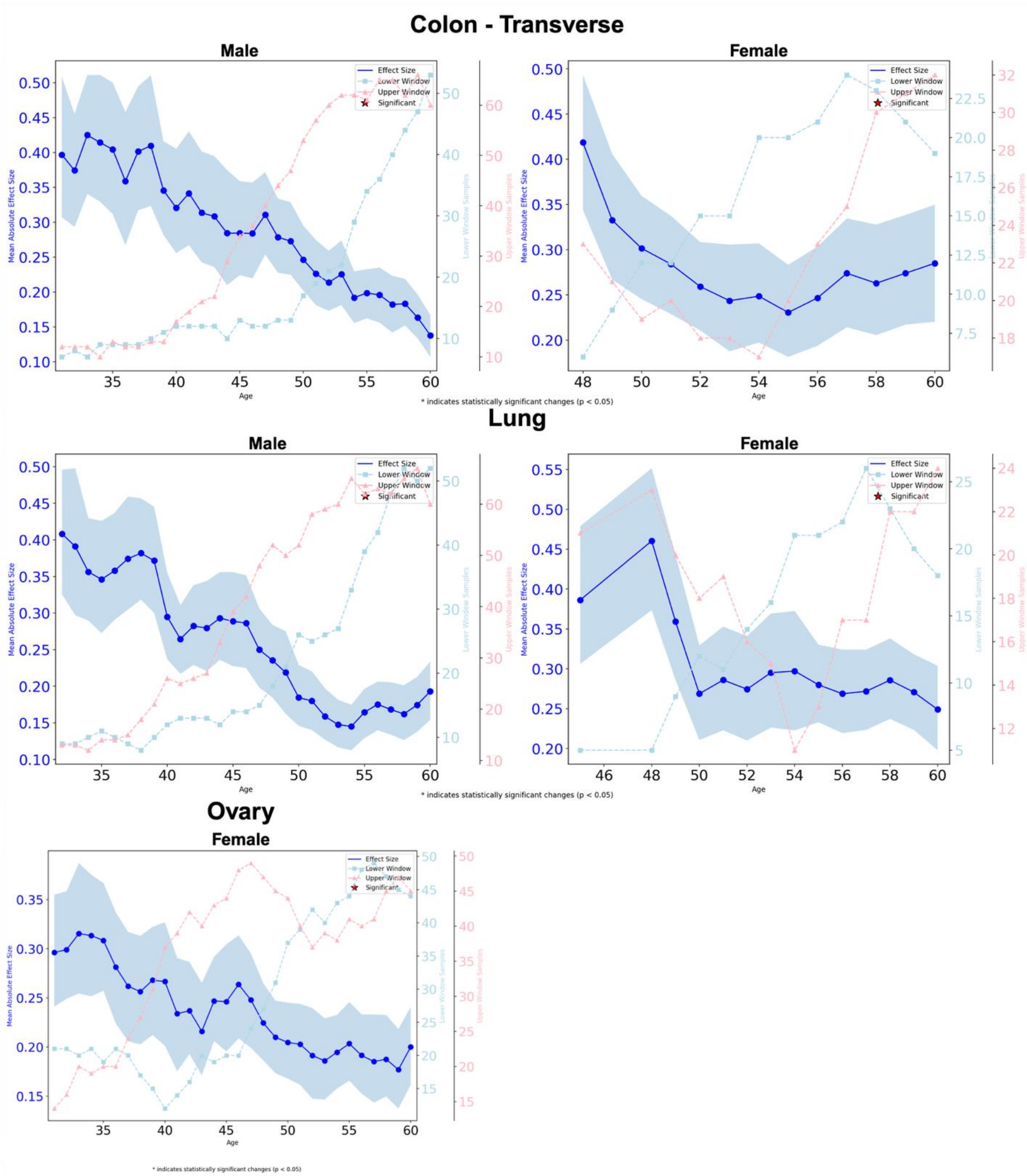

Supplementary Fig 21: DNA Methylation Aging Rate Trajectories Across Human Tissues Stratified by Biological Sex. Sliding window analysis applied to DNA methylation principal component features across 5 tissue-sex combinations (Colon-Transverse and Lung for both sexes, Ovary for females only) to quantify the rate of epigenetic change during aging. Methylation beta values were normalized, converted to M-values, filtered for <5% missing data per CpG, imputed with per-CpG medians, and

reduced to 50 principal components using the top 10,000 most variable CpG sites per tissue. Using 10-year sliding windows across ages 30-79, the magnitude of change in methylation PC space between consecutive age periods was computed to derive tissue-specific and sex-specific aging rate trajectories.

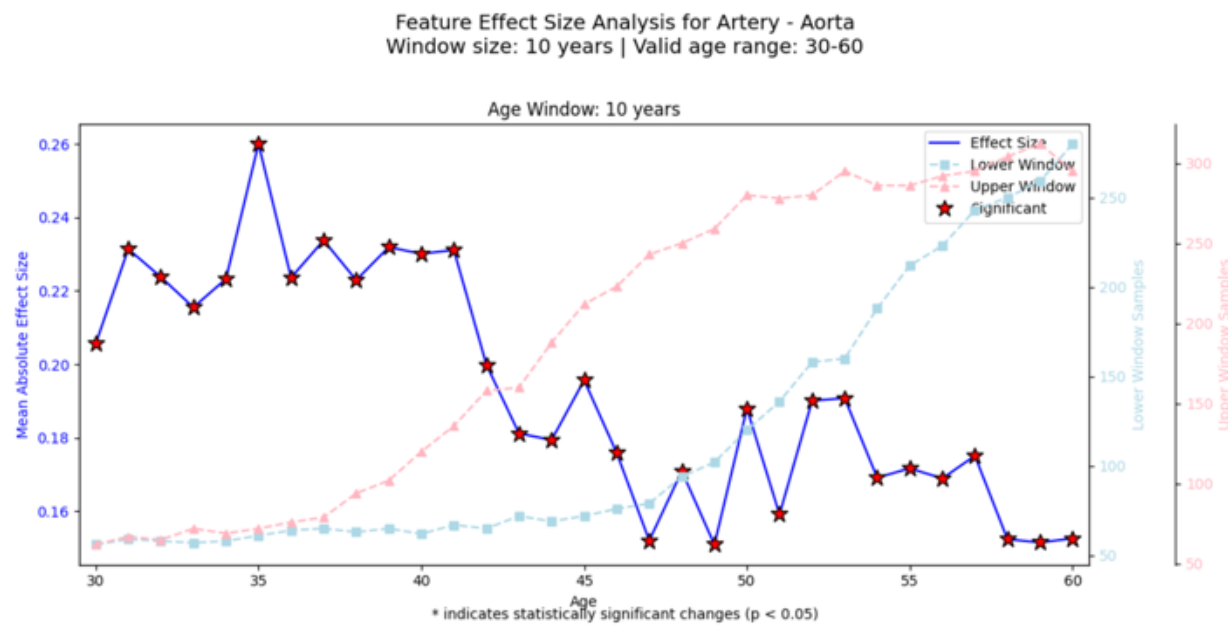
Feature Effect Size Analysis for Artery - Aorta
Window size: 10 years | Valid age range: 30-60
Age Window: 10 years
Mean Absolute Effect Size
Lower Window Samples
Upper Window Samples
Effect Size
Lower Window
Upper Window
Significant
Age
* indicates statistically significant changes (p < 0.05)

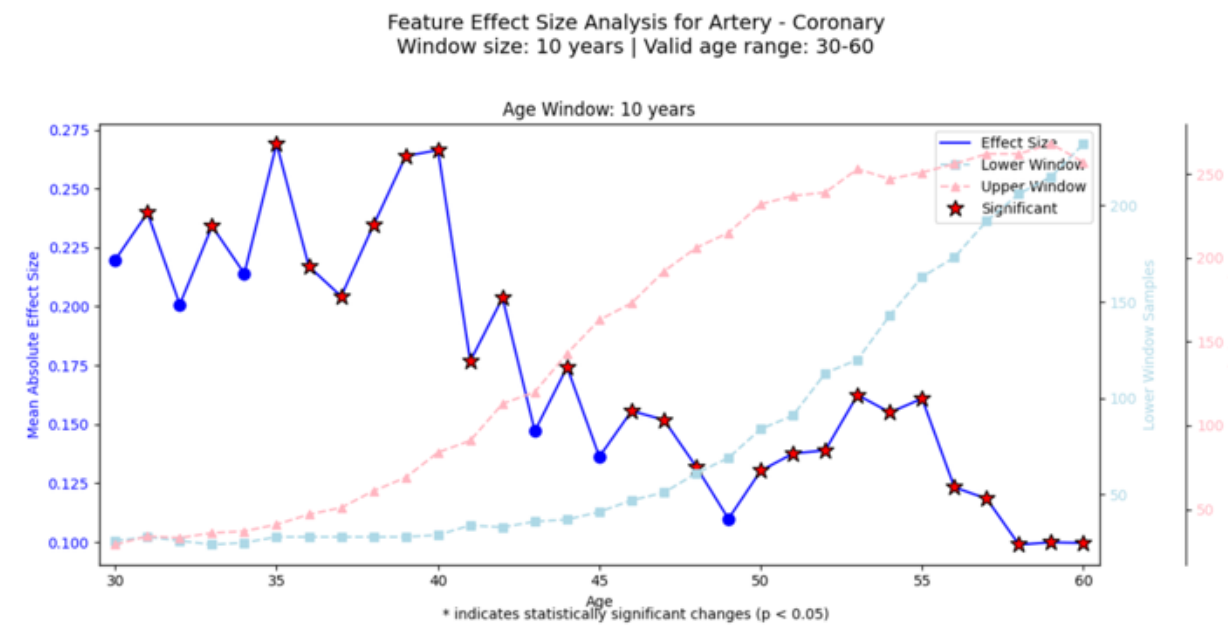
Feature Effect Size Analysis for Artery - Coronary
Window size: 10 years | Valid age range: 30-60
Age Window: 10 years
Mean Absolute Effect Size
Lower Window Samples
Upper Window Samples
Effect Size
Lower Window
Upper Window
Significant
Age
* indicates statistically significant changes (p < 0.05)

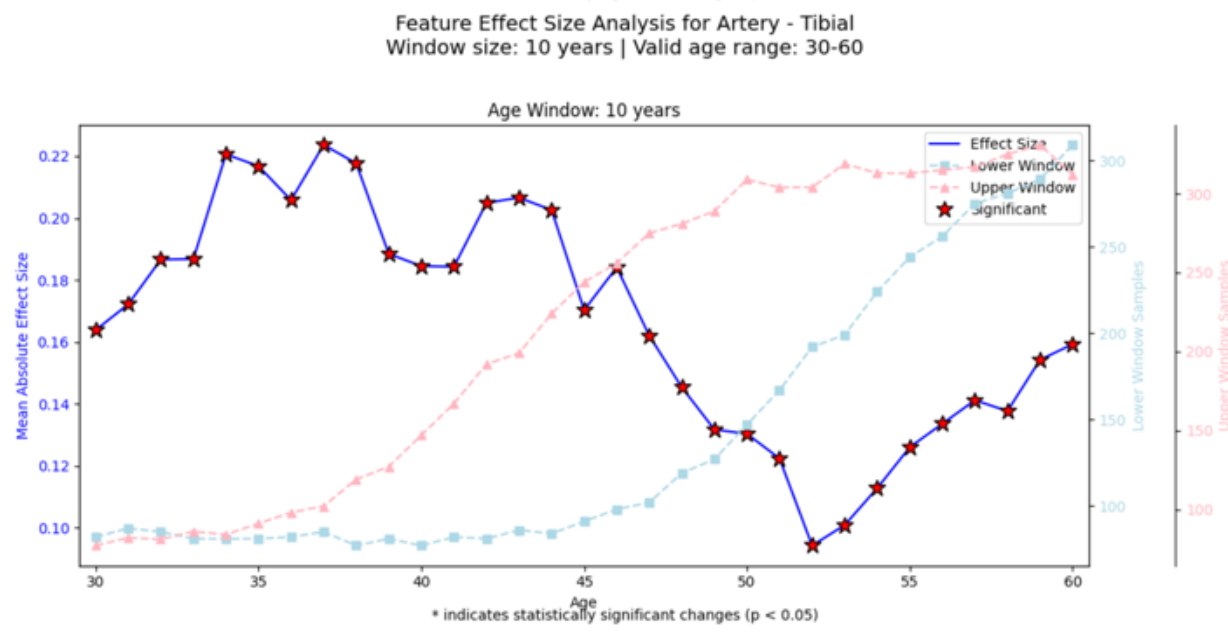
Feature Effect Size Analysis for Artery - Tibial
Window size: 10 years | Valid age range: 30-60
Age Window: 10 years
Mean Absolute Effect Size
Lower Window Samples
Upper Window Samples
Effect Size
Lower Window
Upper Window
Significant
Age
* indicates statistically significant changes (p < 0.05)

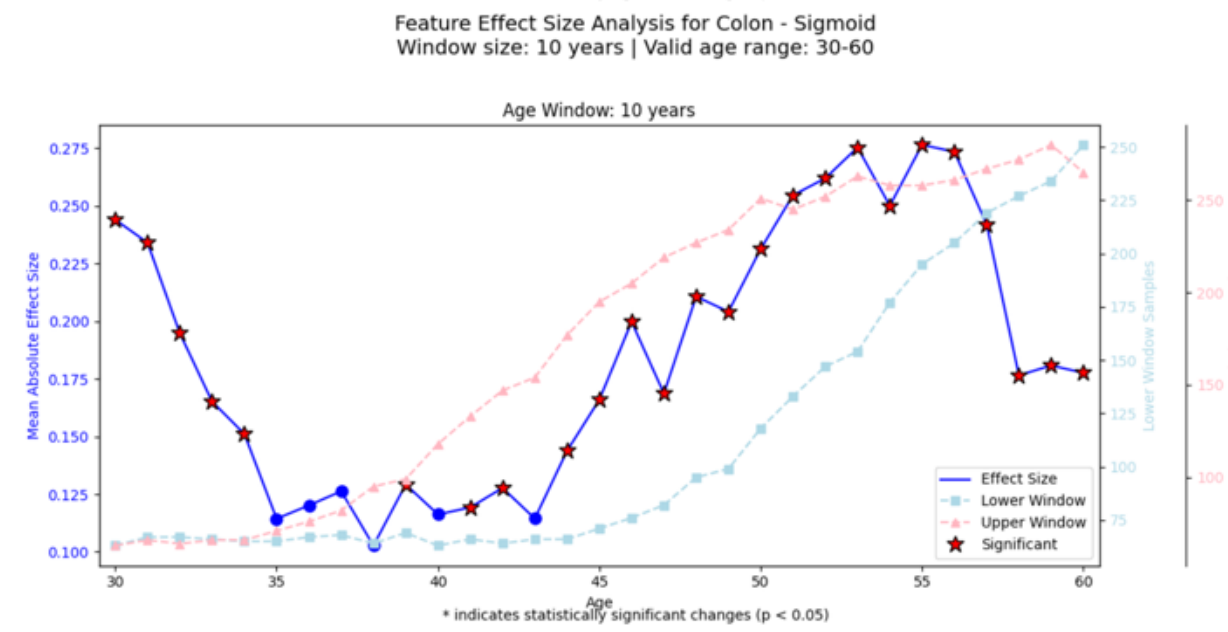
Feature Effect Size Analysis for Colon - Sigmoid
Window size: 10 years | Valid age range: 30-60
Age Window: 10 years
Mean Absolute Effect Size
Lower Window Samples
Upper Window Samples
Effect Size
Lower Window
Upper Window
Significant
Age
* indicates statistically significant changes (p < 0.05)

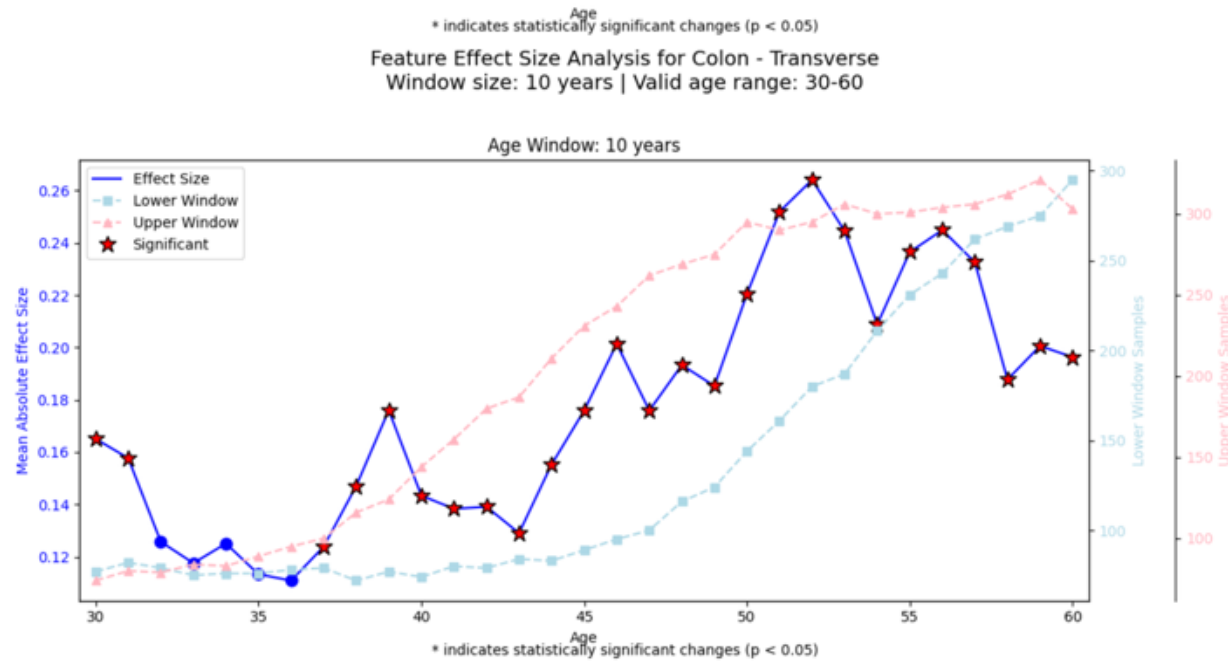
Feature Effect Size Analysis for Colon - Transverse
Window size: 10 years | Valid age range: 30-60
Age Window: 10 years
Mean Absolute Effect Size
Lower Window Samples
Upper Window Samples
Effect Size
Lower Window
Upper Window
Significant
Age
* indicates statistically significant changes (p < 0.05)

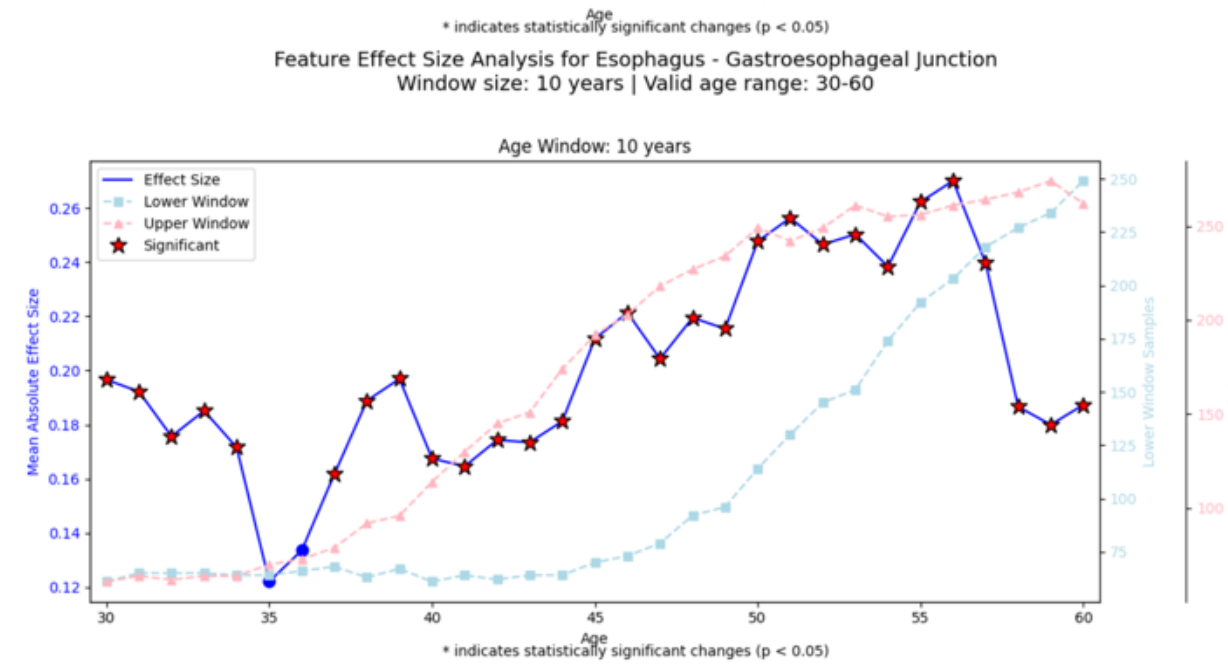
Feature Effect Size Analysis for Esophagus - Gastroesophageal Junction
Window size: 10 years | Valid age range: 30-60
Age Window: 10 years
Mean Absolute Effect Size
Lower Window Samples
Upper Window Samples
Effect Size
Lower Window
Upper Window
Significant
Age
* indicates statistically significant changes (p < 0.05)

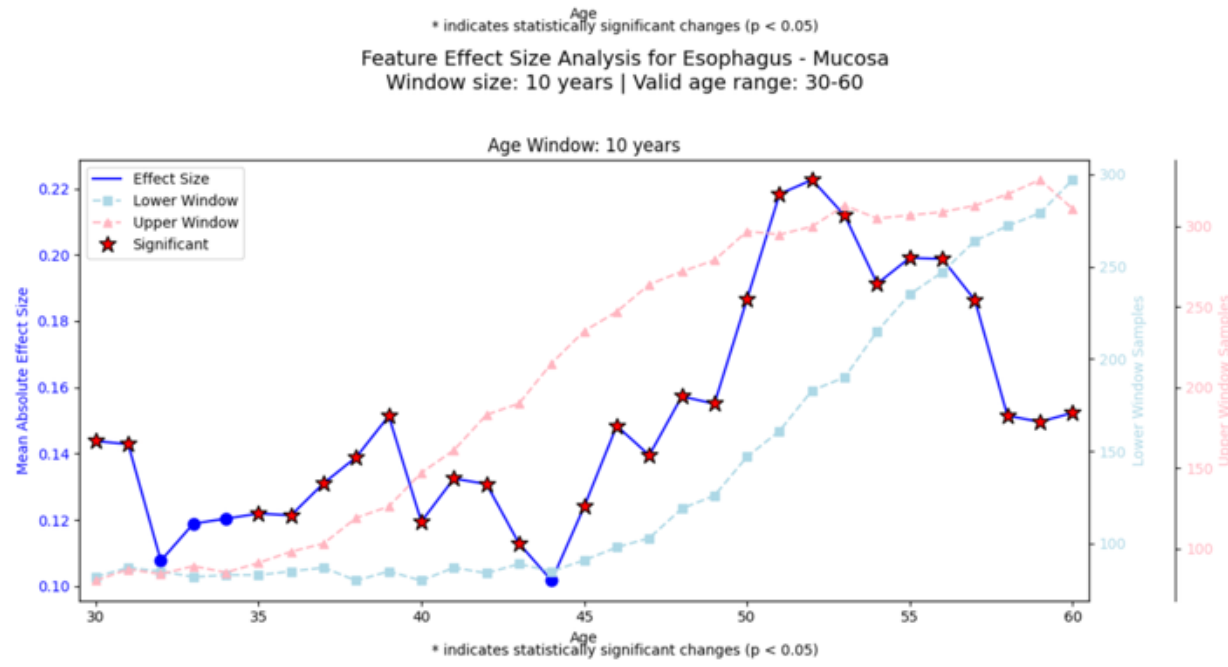
Feature Effect Size Analysis for Esophagus - Mucosa
Window size: 10 years | Valid age range: 30-60
Age Window: 10 years
Mean Absolute Effect Size
Lower Window Samples
Upper Window Samples
Effect Size
Lower Window
Upper Window
Significant
Age
* indicates statistically significant changes (p < 0.05)

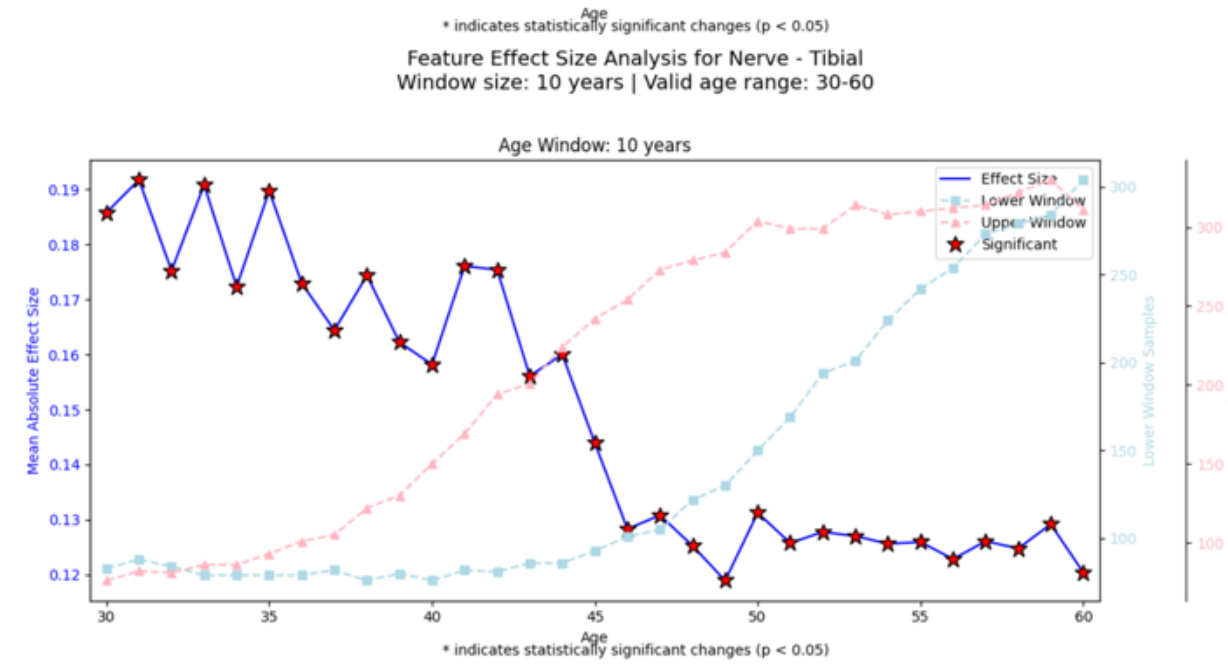
Feature Effect Size Analysis for Nerve - Tibial
Window size: 10 years | Valid age range: 30-60
Age Window: 10 years
Mean Absolute Effect Size
Lower Window Samples
Upper Window Samples
Effect Size
Lower Window
Upper Window
Significant
Age
* indicates statistically significant changes (p < 0.05)

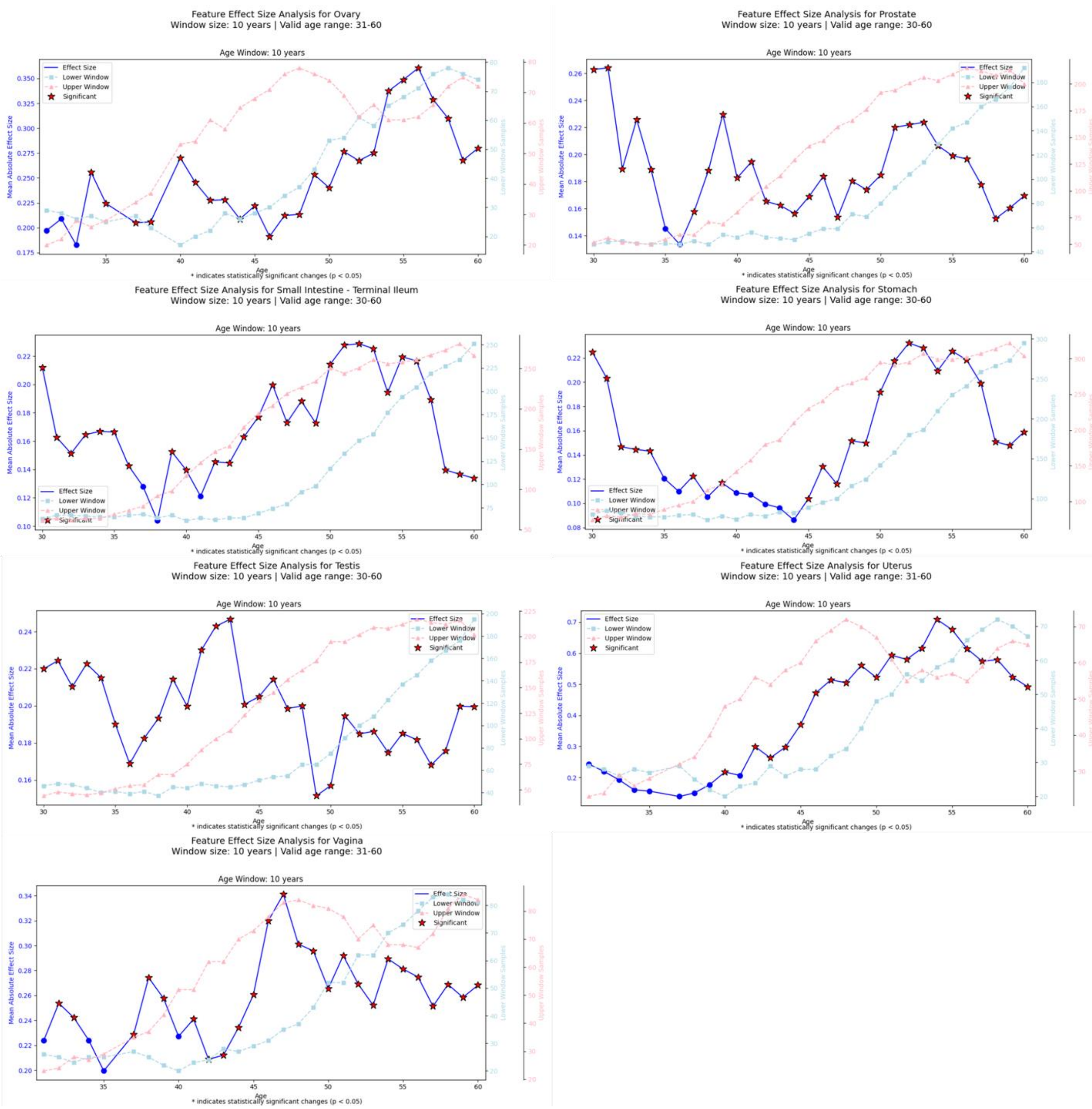

Supplementary Fig 22: Raw Structural Aging Trajectories using UNI v2

Raw Structural Aging Trajectories (blue lines) are shown for the same 15 tissues as in the main analysis. Morphological change between adjacent age windows was computed using slide-level embeddings extracted from **UNI v2**, an updated Vision Transformer–based pathology foundation model trained via large-scale self-supervised learning on diverse whole-slide images. UNI v2 produces high-capacity 1536-dimensional patch embeddings, which were aggregated to construct structural representations for each sample before applying the PathStAR pipeline.

Red stars denote age transitions where ≥5% of features exhibit nominal significance ($p < 0.05$). Dashed curves indicate the number of samples contributing to the lower (squares) and upper (triangles) age windows. The persistence of discrete accelerated structural aging phases across tissues using UNI v2 confirms that PathStAR captures biologically meaningful tissue remodeling patterns independent of embedding dimensionality or specific pretraining strategy.

Feature Effect Size Analysis for Artery - Aorta
Window size: 10 years | Valid age range: 30-60

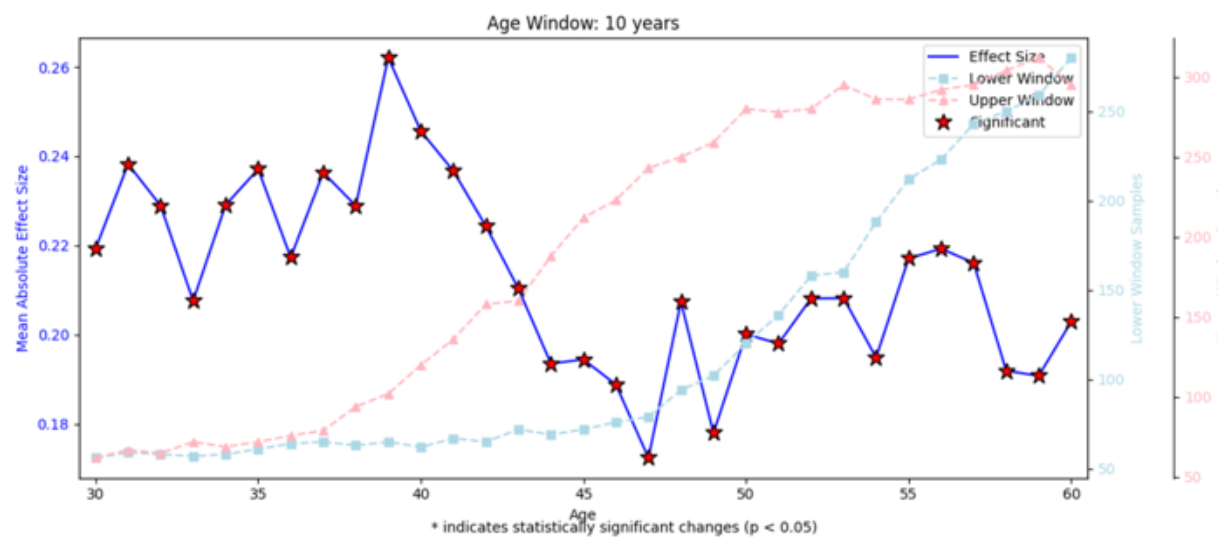

Feature Effect Size Analysis for Artery - Coronary
Window size: 10 years | Valid age range: 30-60

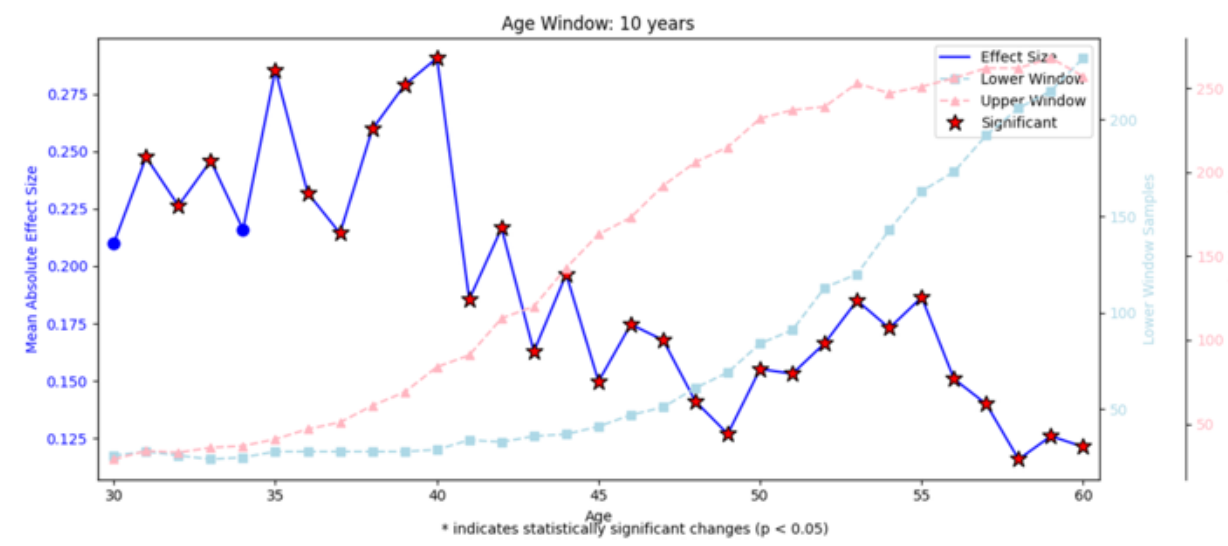

Feature Effect Size Analysis for Artery - Tibial
Window size: 10 years | Valid age range: 30-60

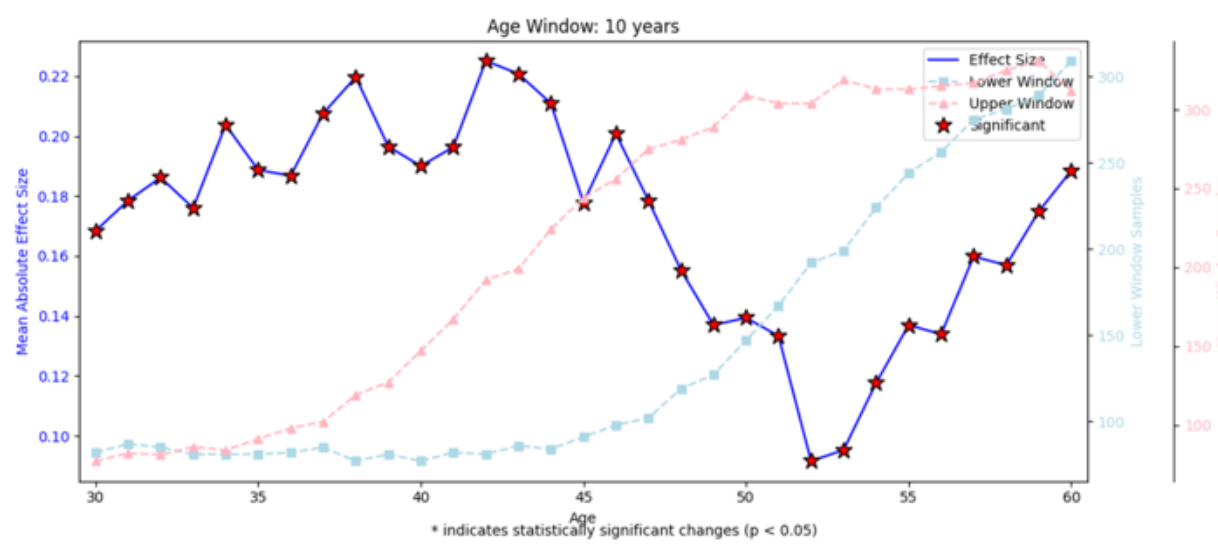

Feature Effect Size Analysis for Colon - Sigmoid
Window size: 10 years | Valid age range: 30-60

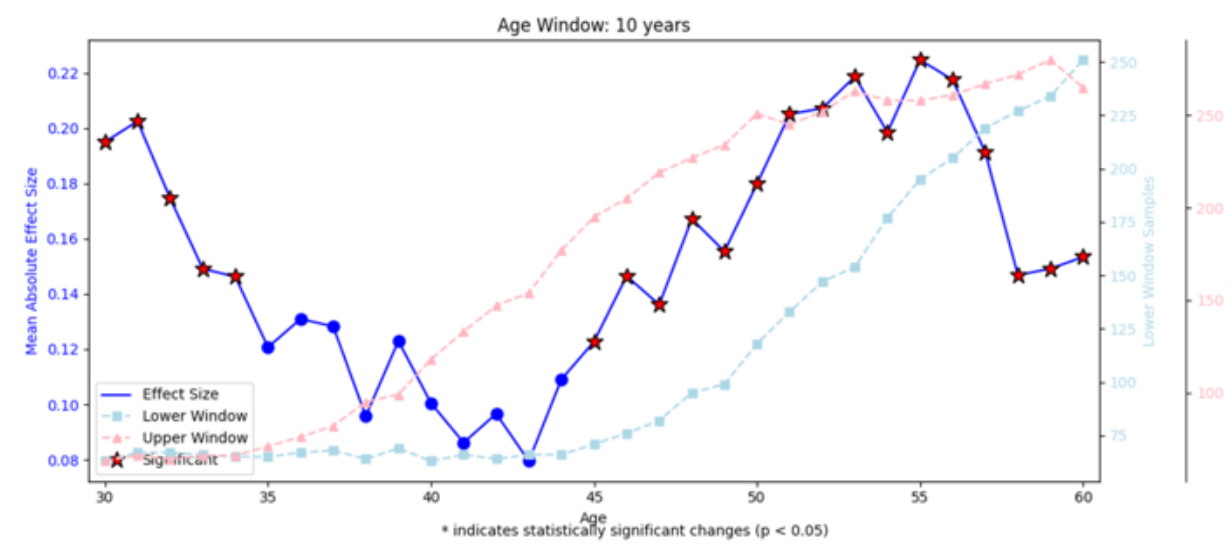

Feature Effect Size Analysis for Colon - Transverse
Window size: 10 years | Valid age range: 30-60

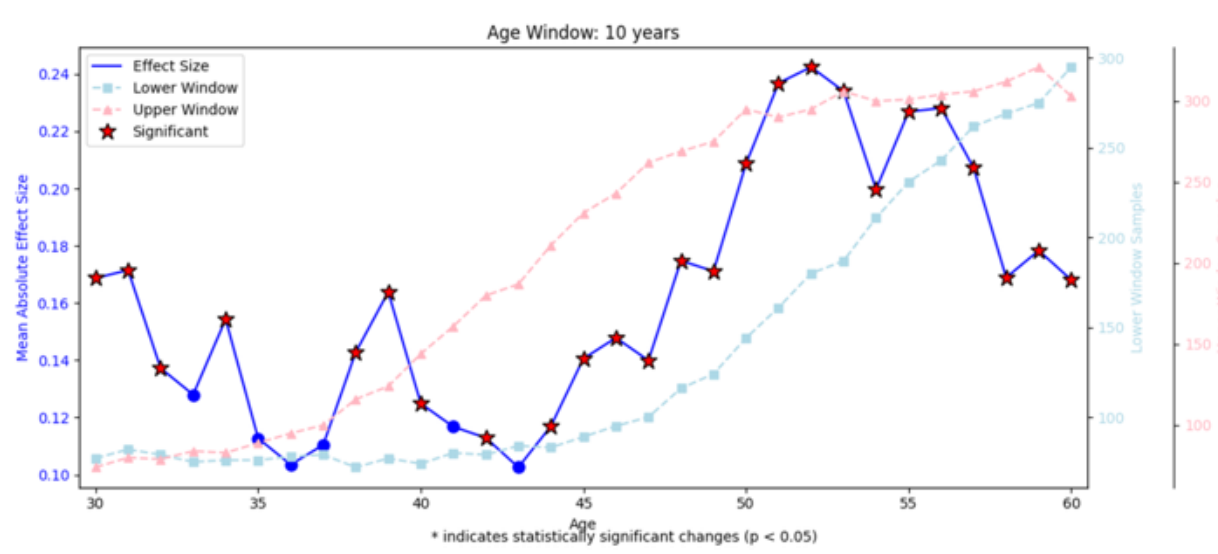

Feature Effect Size Analysis for Esophagus - Gastroesophageal Junction
Window size: 10 years | Valid age range: 30-60

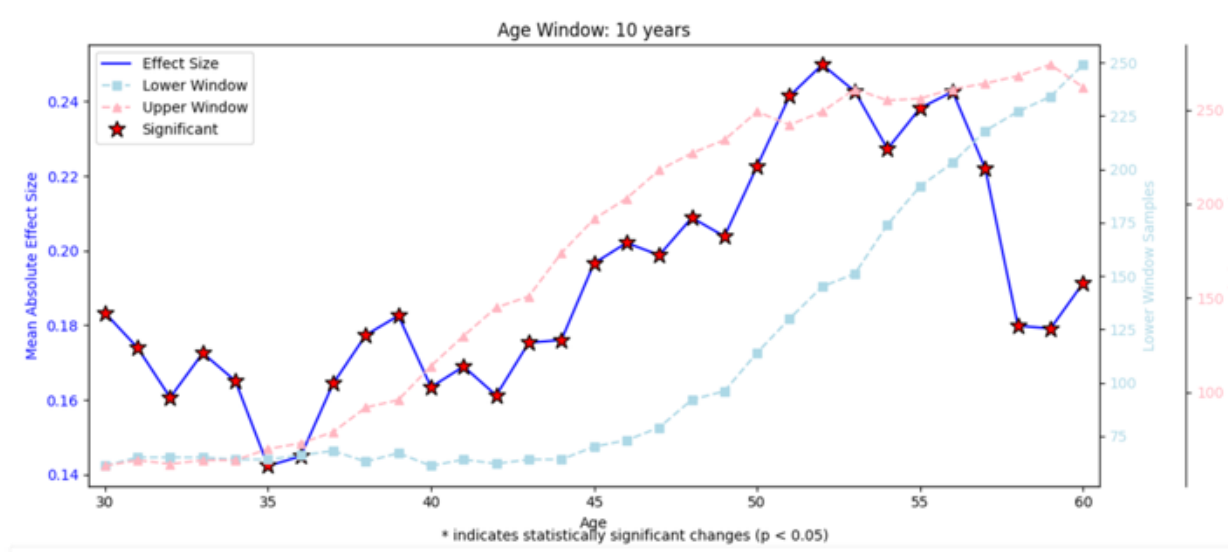

Feature Effect Size Analysis for Esophagus - Mucosa
Window size: 10 years | Valid age range: 30-60

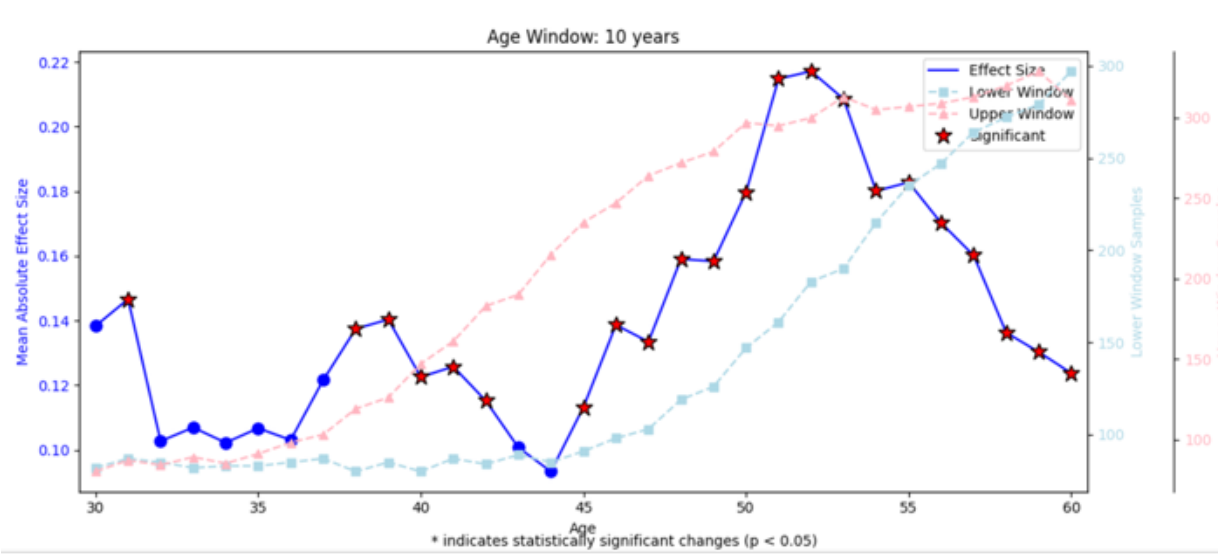

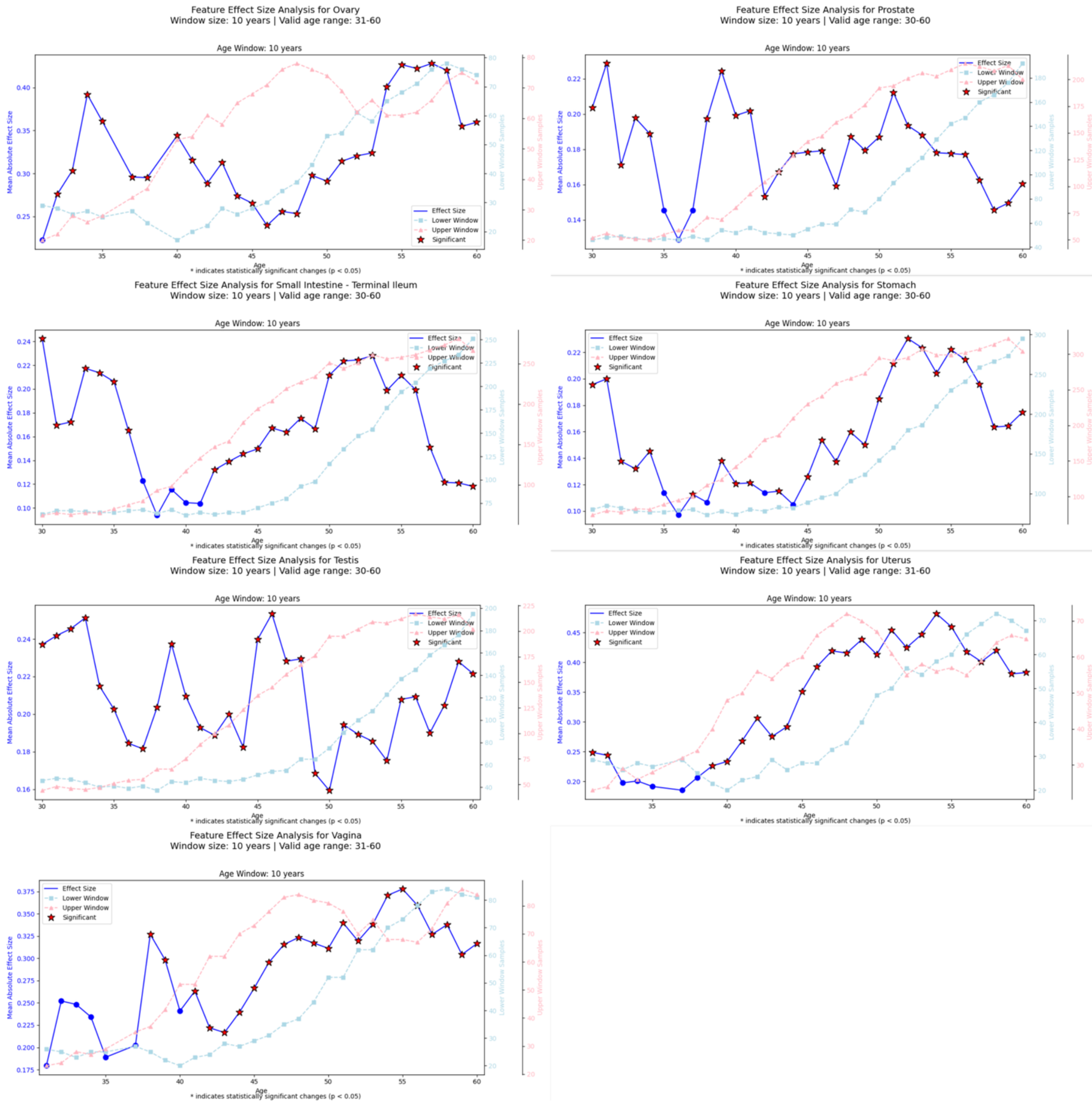

Supplementary Fig 23 : Raw Structural Aging Trajectories using CONCH v1.5. Raw Structural Aging Trajectories (blue lines) are shown for the same 15 tissues: Artery (Aorta, Coronary, Tibial), Colon (Sigmoid, Transverse), Esophagus (Gastroesophageal Junction, Muscularis), Nerve (Tibial), Ovary, Prostate, Small Intestine (Terminal Ileum), Stomach, Testis, Uterus, and Vagina. Each trajectory represents the magnitude of morphological change between adjacent age windows, computed using slide-level representations derived from **CONCH v1.5**, a vision-language pathology foundation model trained on large-scale histopathology datasets with contrastive supervision. Patch embeddings (768-dimensional) were extracted and aggregated to generate tissue-level structural representations prior to PathStAR analysis.

Red stars denote age transitions where ≥5% of features exhibit nominal significance ($p < 0.05$). Dashed curves on the secondary axis show the number of samples contributing to the lower (squares) and upper (triangles) age windows at each transition. The recapitulation of tissue-specific accelerated periods using CONCH demonstrates that structural aging trajectories are robust to the choice of feature extractor and not dependent on a single pretrained model.

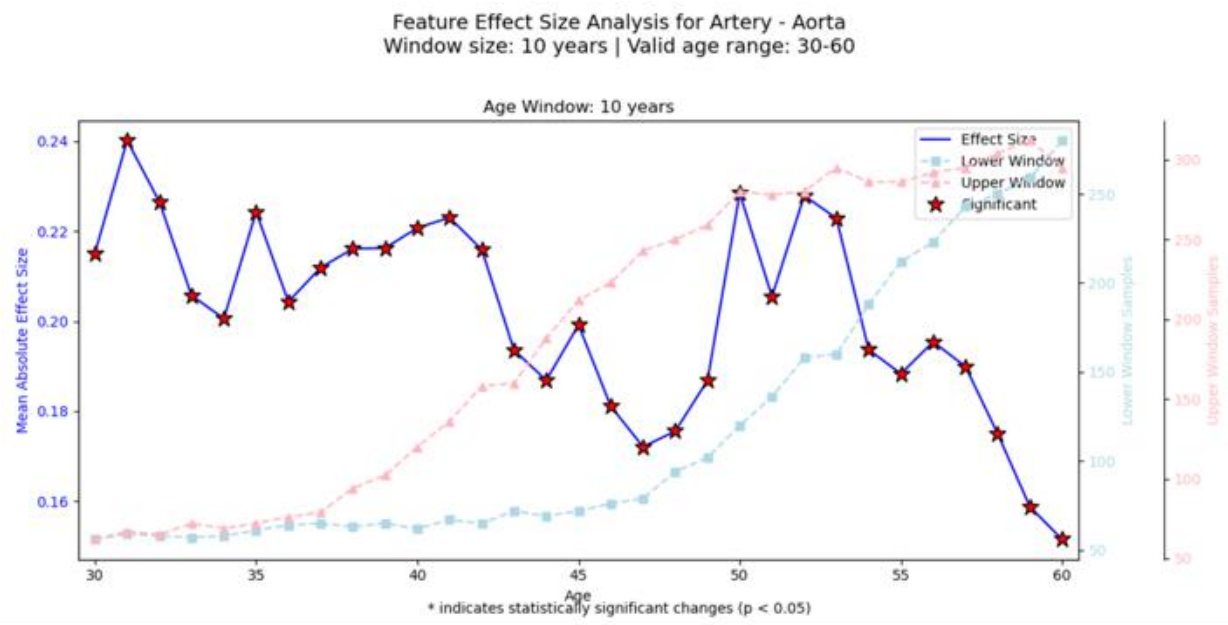
Feature Effect Size Analysis for Artery - Aorta
Window size: 10 years | Valid age range: 30-60
Age Window: 10 years
Effect Size
Lower Window
Upper Window
Significant
Mean Absolute Effect Size
Lower Window Samples
Upper Window Samples
Age
* indicates statistically significant changes (p < 0.05)

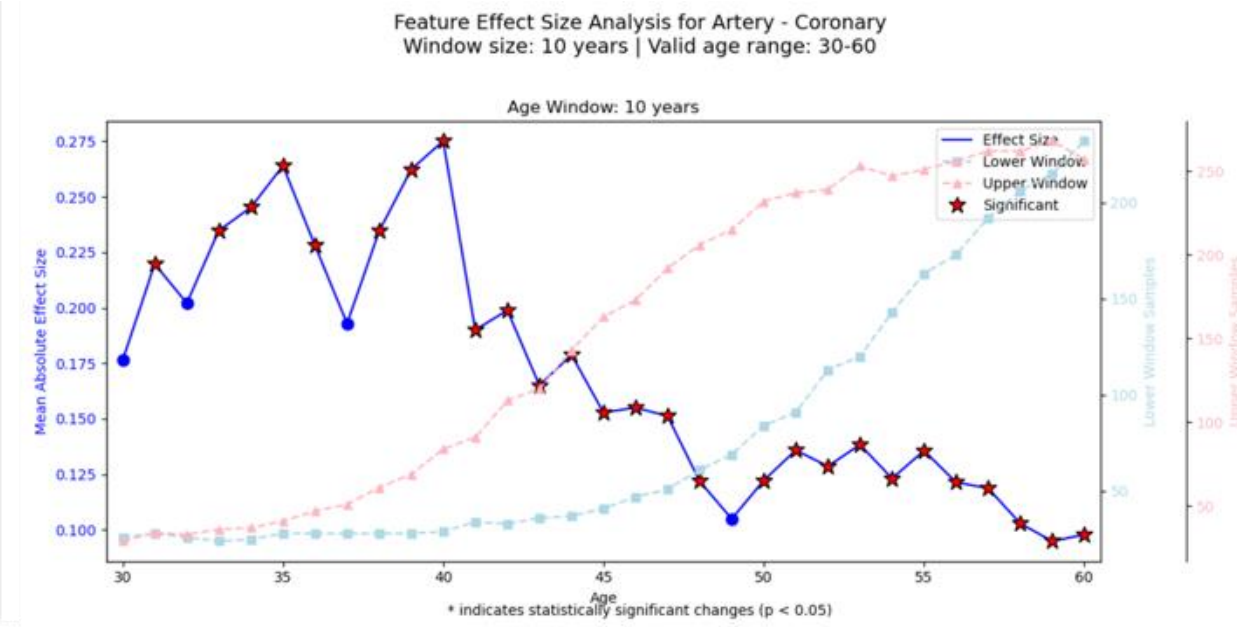
Feature Effect Size Analysis for Artery - Coronary
Window size: 10 years | Valid age range: 30-60
Age Window: 10 years
Effect Size
Lower Window
Upper Window
Significant
Mean Absolute Effect Size
Lower Window Samples
Upper Window Samples
Age
* indicates statistically significant changes (p < 0.05)

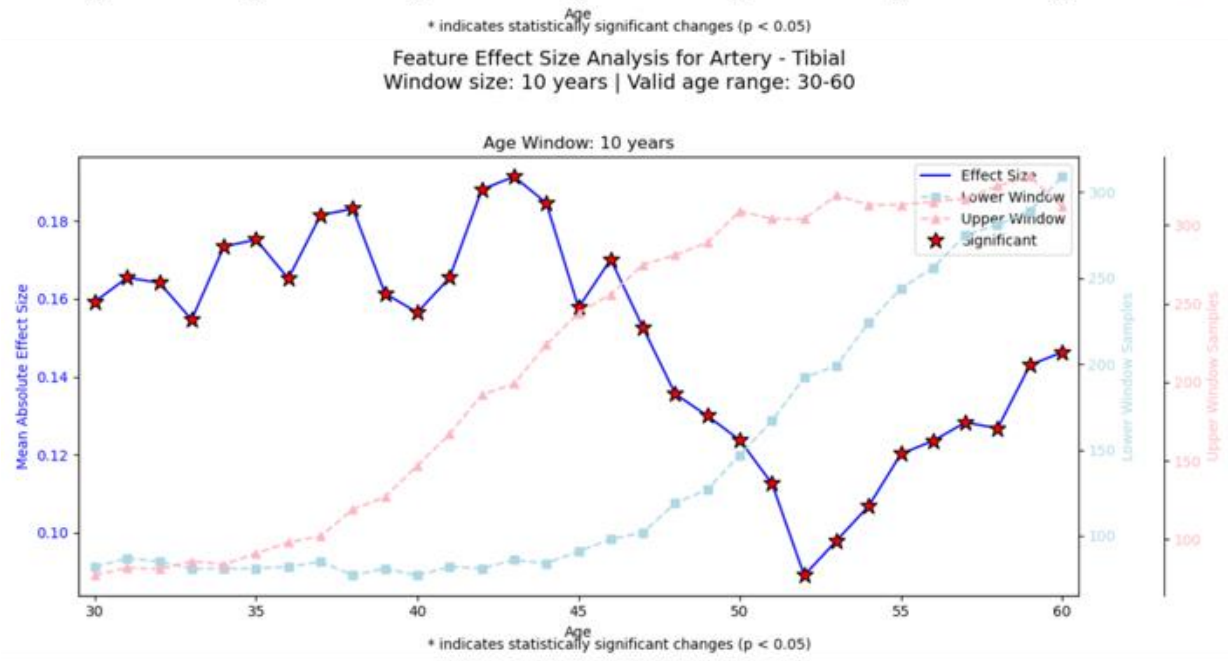
Feature Effect Size Analysis for Artery - Tibial
Window size: 10 years | Valid age range: 30-60
Age Window: 10 years
Effect Size
Lower Window
Upper Window
Significant
Mean Absolute Effect Size
Lower Window Samples
Upper Window Samples
Age
* indicates statistically significant changes (p < 0.05)

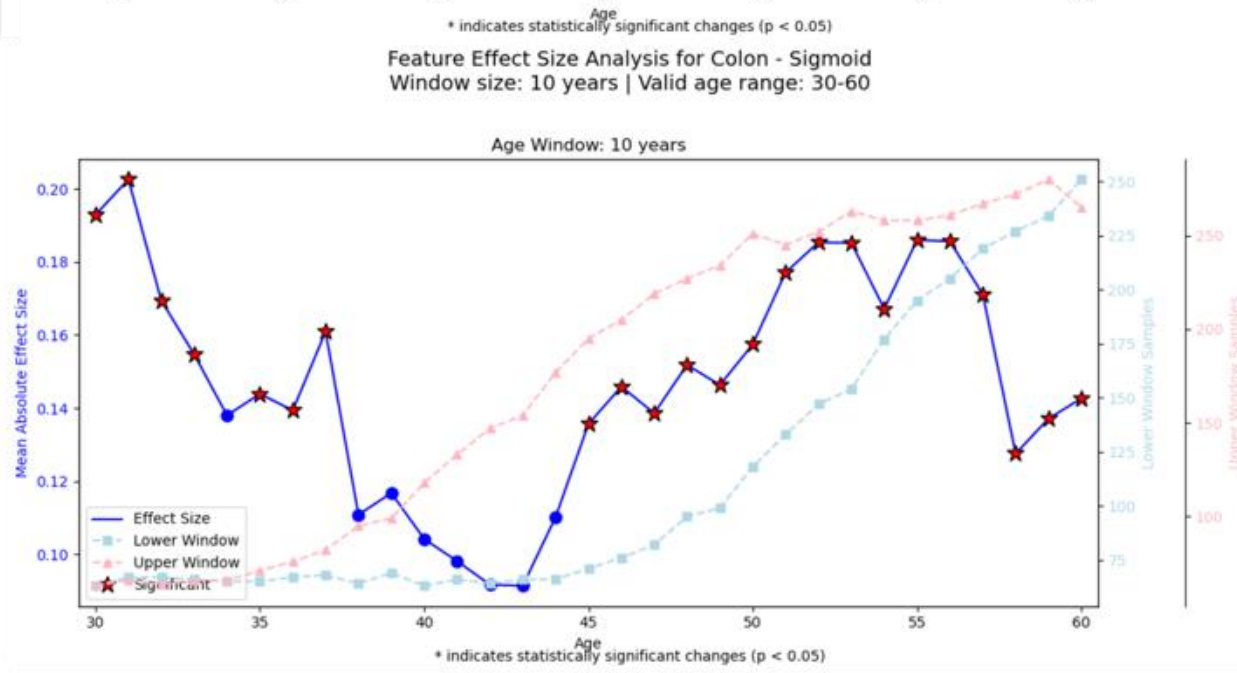
Feature Effect Size Analysis for Colon - Sigmoid
Window size: 10 years | Valid age range: 30-60
Age Window: 10 years
Effect Size
Lower Window
Upper Window
Significant
Mean Absolute Effect Size
Lower Window Samples
Upper Window Samples
Age
* indicates statistically significant changes (p < 0.05)

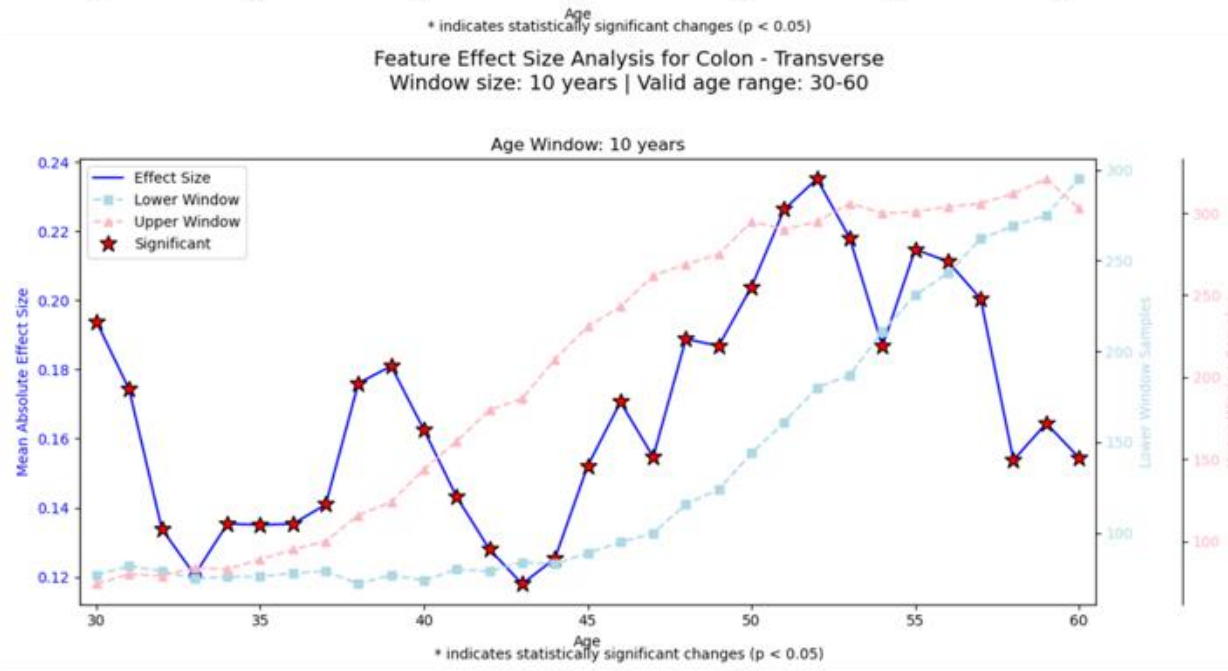
Feature Effect Size Analysis for Colon - Transverse
Window size: 10 years | Valid age range: 30-60
Age Window: 10 years
Effect Size
Lower Window
Upper Window
Significant
Mean Absolute Effect Size
Lower Window Samples
Upper Window Samples
Age
* indicates statistically significant changes (p < 0.05)

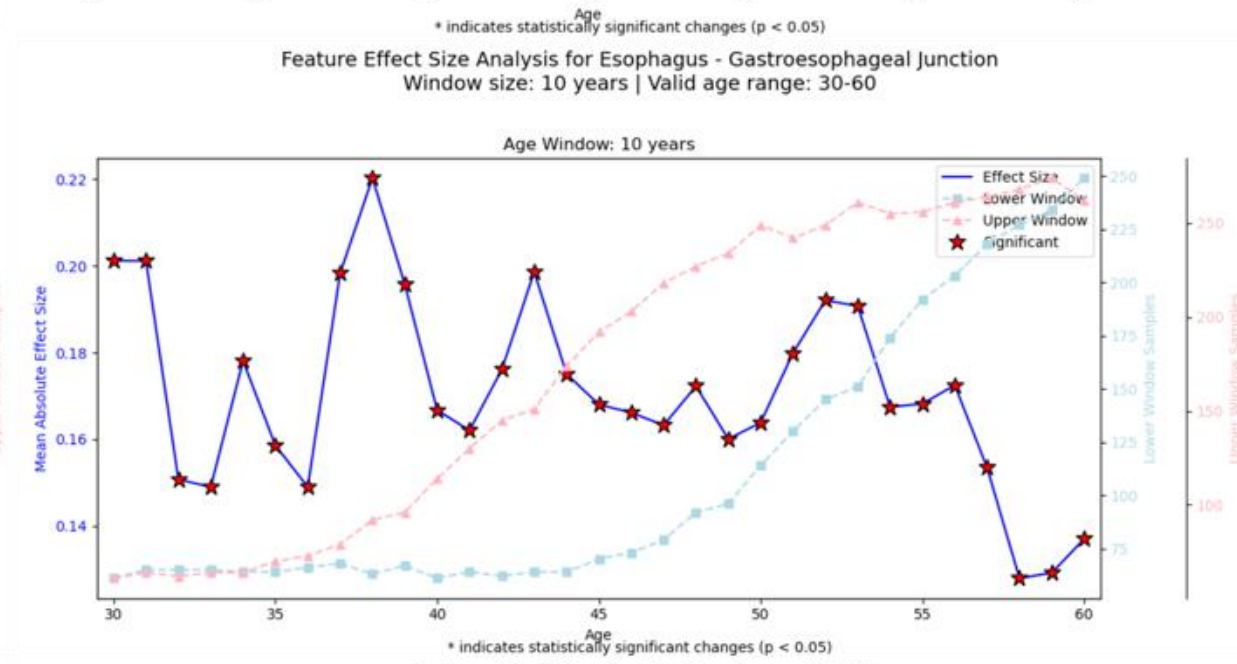
Feature Effect Size Analysis for Esophagus - Gastroesophageal Junction
Window size: 10 years | Valid age range: 30-60
Age Window: 10 years
Effect Size
Lower Window
Upper Window
Significant
Mean Absolute Effect Size
Lower Window Samples
Upper Window Samples
Age
* indicates statistically significant changes (p < 0.05)

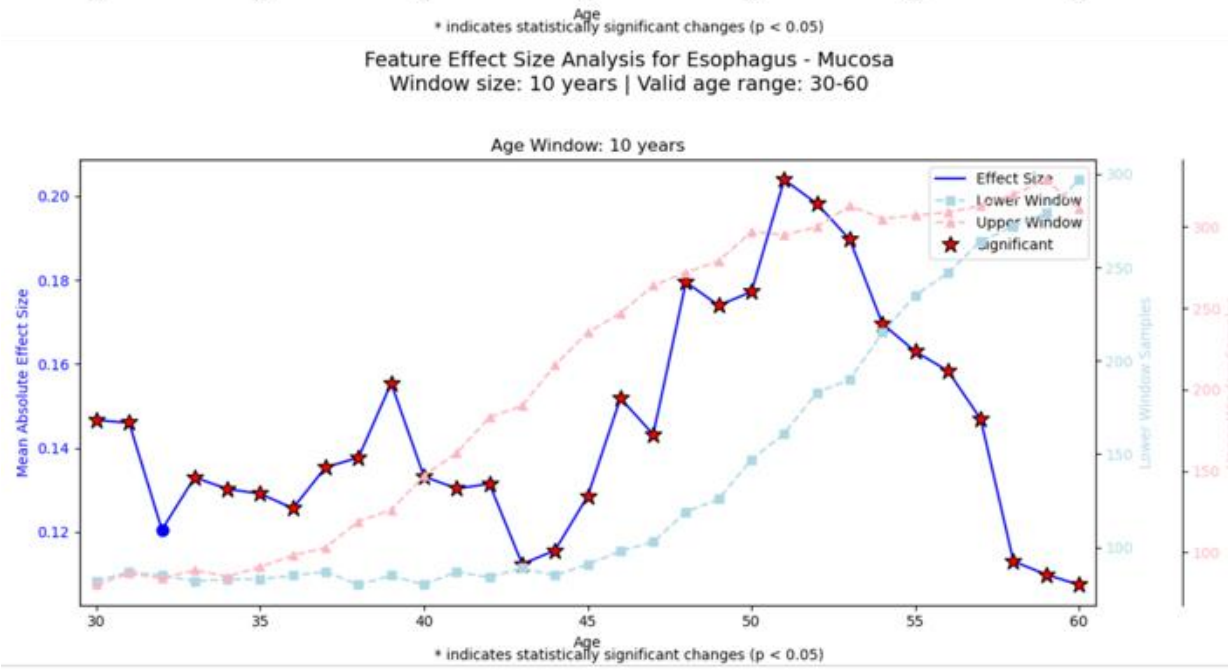
Feature Effect Size Analysis for Esophagus - Mucosa
Window size: 10 years | Valid age range: 30-60
Age Window: 10 years
Effect Size
Lower Window
Upper Window
Significant
Mean Absolute Effect Size
Lower Window Samples
Upper Window Samples
Age
* indicates statistically significant changes (p < 0.05)

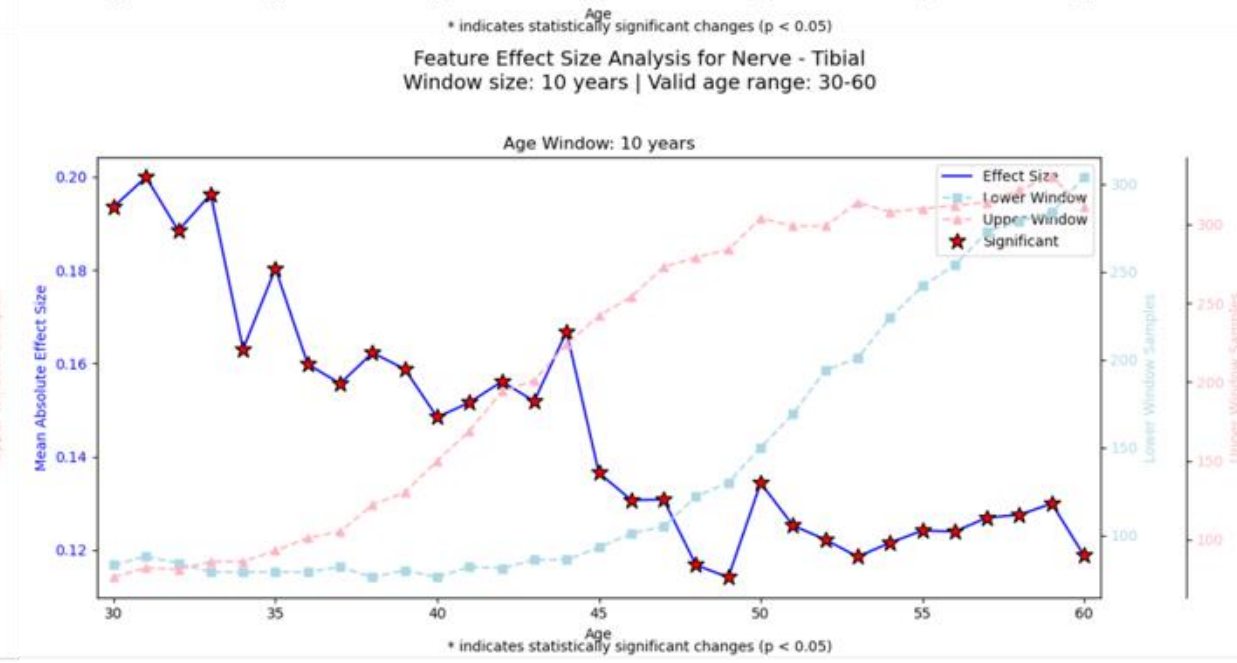
Feature Effect Size Analysis for Nerve - Tibial
Window size: 10 years | Valid age range: 30-60
Age Window: 10 years
Effect Size
Lower Window
Upper Window
Significant
Mean Absolute Effect Size
Lower Window Samples
Upper Window Samples
Age
* indicates statistically significant changes (p < 0.05)

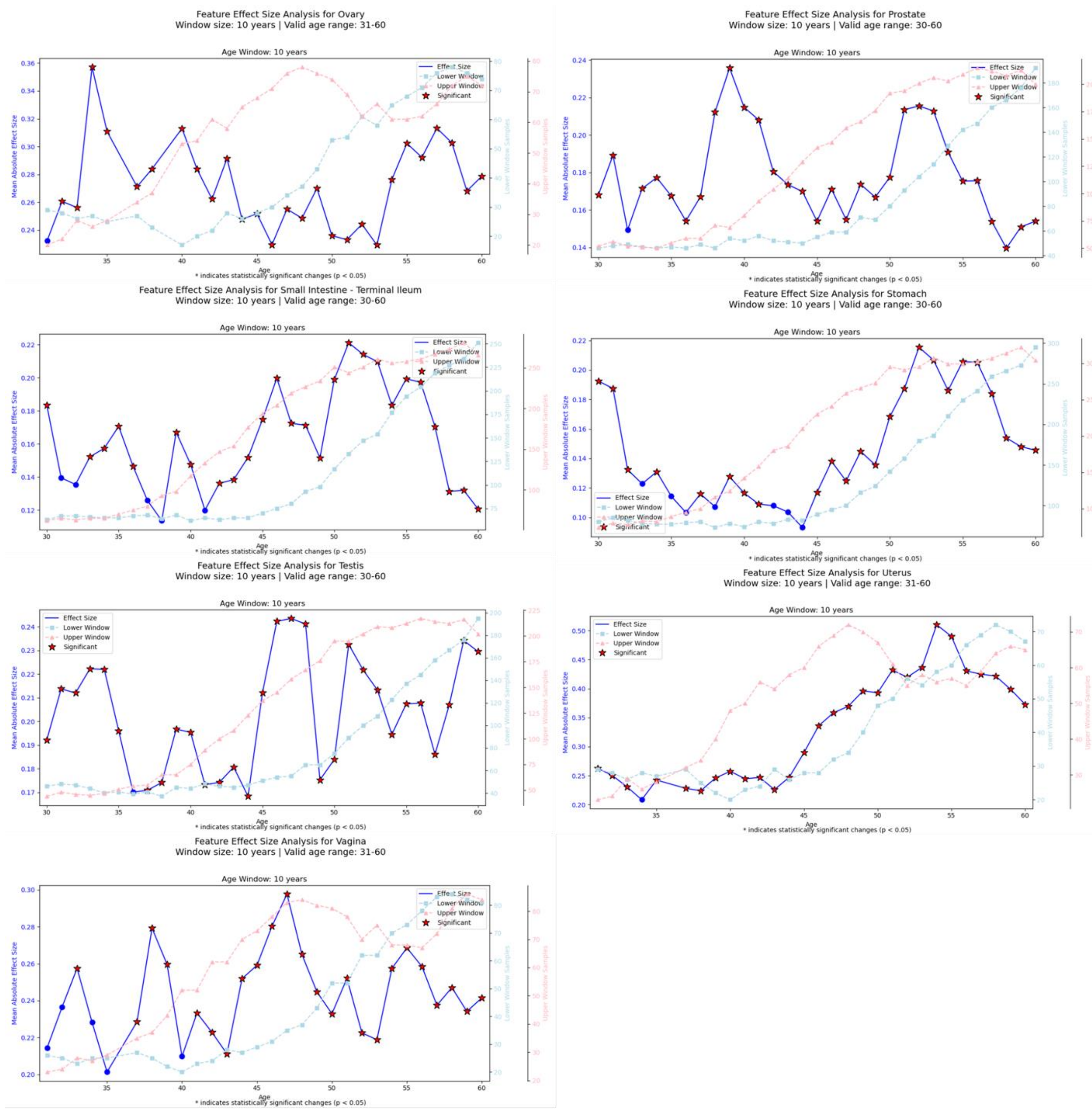

Supplementary Fig 24: Raw Structural Aging Trajectories using Virchow 2. Raw Structural Aging Trajectories (blue lines) are shown for the same 15 tissues. Structural transitions were computed using embeddings extracted from **Virchow 2**, a large-scale histopathology foundation model trained using self-supervised transformer architectures optimized for morphological representation learning. Virchow 2 generates 1280-dimensional patch embeddings, which were aggregated into slide-level representations prior to PathStAR analysis. Red stars denote age transitions where ≥5% of features show nominal significance ($p < 0.05$). Dashed curves display the number of samples contributing to each adjacent age comparison. Replication of tissue-specific structural aging patterns in most tissues using Virchow 2 further demonstrates that the observed non-linear and tissue-specific remodeling trajectories are not artifacts of a particular

model architecture but reflect consistent age-dependent structural reorganization detectable across independent foundation models.

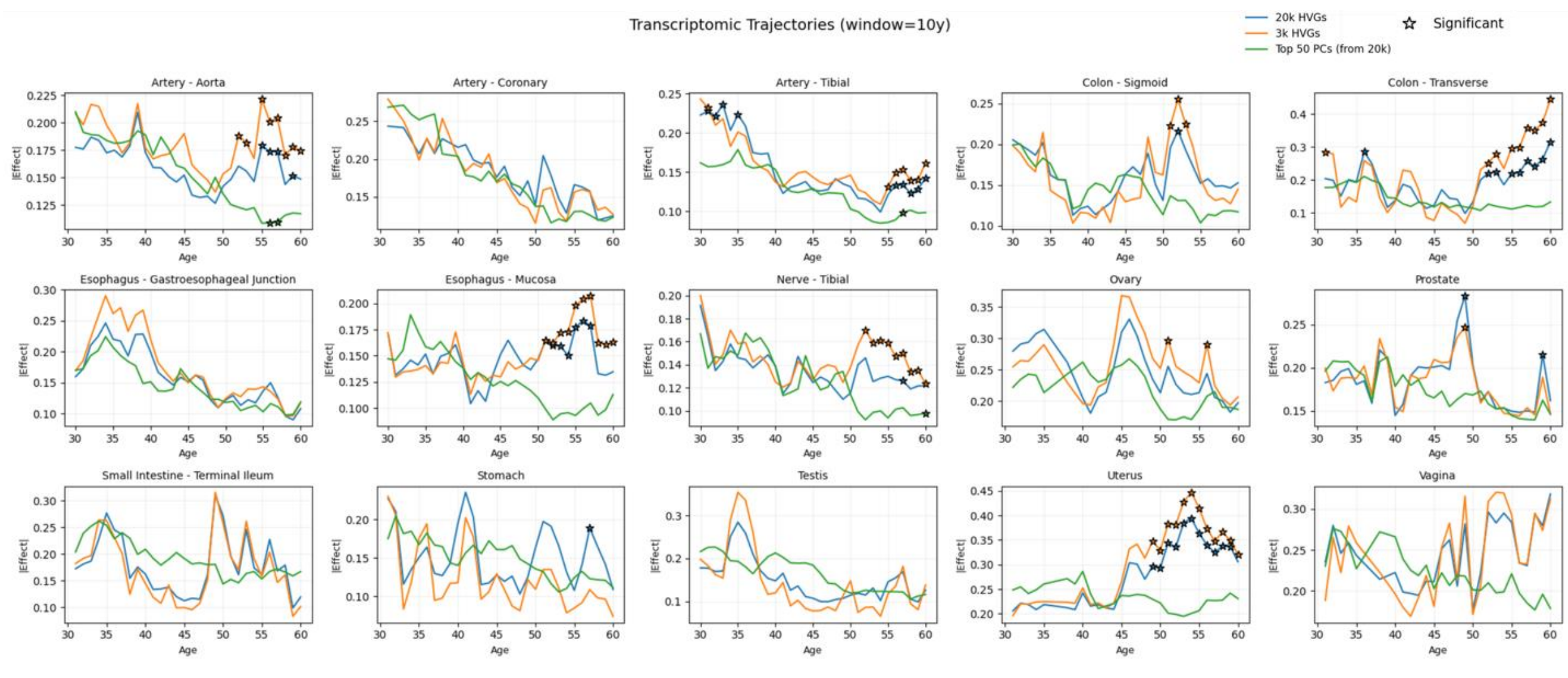

Supplementary Fig 25 : Transcriptomic aging trajectories across tissues (window = 10 years). We computed sliding-window effect sizes across age using three feature spaces per tissue: top 20k highly variable genes (HVGs), top 3k HVGs, and the top 50 principal components (PCs) derived from the 20k HVGs. Across tissues, trajectories derived from PCA (top 50 PCs) show smoother, more consistent age-dependent patterns and clearer significant transitions, indicating that PCA better captures the dominant aging trajectory compared to raw gene-level features.

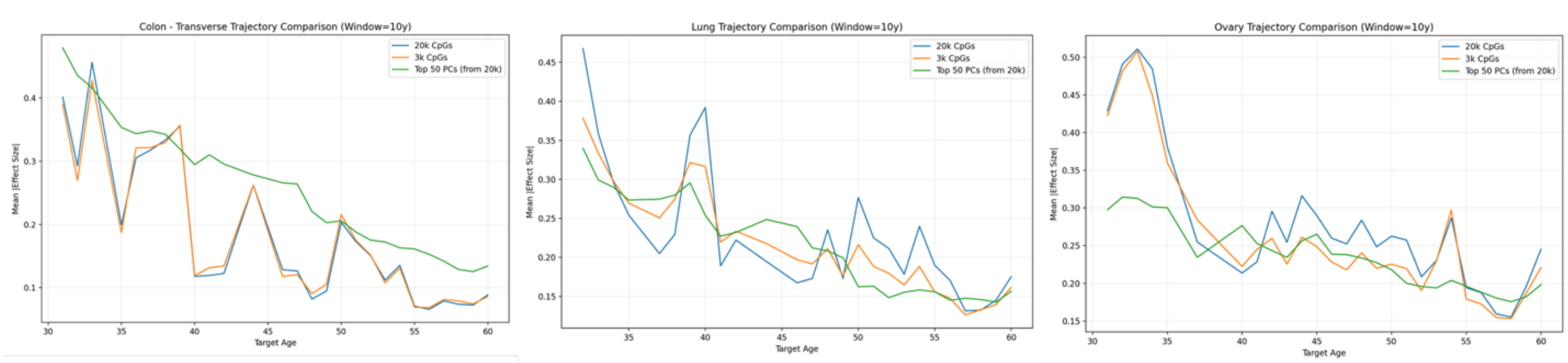

Supplementary Fig 26: Methylation aging trajectory comparison (window = 10 years). Using the same sliding-window framework, we compared trajectories built from 20k CpGs, 3k CpGs, and the top 50 PCs derived from the 20k CpGs. PCA-based trajectories exhibit more stable age trends and reduced noise, suggesting that low-

dimensional principal components more effectively capture the underlying methylation aging structure than individual CpG features.
