# Supplementary material for: Mapping structural aging across human tissues reveals tissue-specific trajectories and coordinated deterioration
Source: Nat Aging. 2026 Aug 31;6(9):2013–27. doi: 10.1038/s43587-026-01200-4 (PMC13577920; doi:10.1038/s43587-026-01200-4)
Supplement: Supplementary file 2 [file 43587_2026_1200_MOESM2_ESM.pdf]

nature portfolio

Corresponding author(s): Sanju Sinha

Last updated by author(s): Jun 29, 2026

Reporting Summary

Nature Portfolio wishes to improve the reproducibility of the work that we publish. This form provides structure for consistency and transparency in reporting. For further information on Nature Portfolio policies, see our Editorial Policies and the Editorial Policy Checklist.

Statistics

For all statistical analyses, confirm that the following items are present in the figure legend, table legend, main text, or Methods section.

n/a	Confirmed	
☐	☒	The exact sample size (*n*) for each experimental group/condition, given as a discrete number and unit of measurement
☐	☒	A statement on whether measurements were taken from distinct samples or whether the same sample was measured repeatedly
☐	☒	The statistical test(s) used AND whether they are one- or two-sided<br>*Only common tests should be described solely by name; describe more complex techniques in the Methods section.*
☐	☒	A description of all covariates tested
☐	☒	A description of any assumptions or corrections, such as tests of normality and adjustment for multiple comparisons
☐	☒	A full description of the statistical parameters including central tendency (e.g. means) or other basic estimates (e.g. regression coefficient) AND variation (e.g. standard deviation) or associated estimates of uncertainty (e.g. confidence intervals)
☐	☒	For null hypothesis testing, the test statistic (e.g. *F*, *t*, *r*) with confidence intervals, effect sizes, degrees of freedom and *P* value noted<br>*Give P values as exact values whenever suitable.*
☒	☐	For Bayesian analysis, information on the choice of priors and Markov chain Monte Carlo settings
☐	☒	For hierarchical and complex designs, identification of the appropriate level for tests and full reporting of outcomes
☐	☒	Estimates of effect sizes (e.g. Cohen's *d*, Pearson's *r*), indicating how they were calculated

Our web collection on statistics for biologists contains articles on many of the points above.

Software and code

Policy information about availability of computer code

Data collection	No software was used for data collection. All data were obtained from the Genotype-Tissue Expression (GTEx) Project (whole-slide histopathology images, transcriptomic, methylation and whole-exome genotype data); no new data were generated. Controlled-access data were obtained via dbGaP (accession phs000424.v10.p2; project #39103).
Data analysis	Analyses were performed in Python 3.10. The custom PathStAR pipeline is available at https://github.com/Sinha-CompBio-Lab/PathStAR (Apache License 2.0). Numerical and data handling used NumPy ≥1.23, pandas ≥1.5 and pyarrow ≥11. Statistical analyses used SciPy ≥1.9 (Welch's t-test, Pearson correlation, UnivariateSpline), scikit-learn ≥1.2 (linear discriminant analysis, Gaussian Process regression, PCA) and statsmodels (linear regression of ΔSA on gene burden). Pathway enrichment used GSEApy against MSigDB Hallmark, KEGG, GO and C3-TFT gene sets. Histology feature extraction used the UNI v1 vision foundation model (with UNI v2, CONCH v1.5 and Virchow2 for robustness analyses). Figures were generated with matplotlib ≥3.6 and seaborn ≥0.12.

For manuscripts utilizing custom algorithms or software that are central to the research but not yet described in published literature, software must be made available to editors and reviewers. We strongly encourage code deposition in a community repository (e.g. GitHub). See the Nature Portfolio guidelines for submitting code & software for further information.

Data

Policy information about availability of data

All manuscripts must include a data availability statement. This statement should provide the following information, where applicable:

- Accession codes, unique identifiers, or web links for publicly available datasets
- A description of any restrictions on data availability
- For clinical datasets or third party data, please ensure that the statement adheres to our policy

All data derive from the GTEx Project (dbGaP accession phs000424.v10.p2). H&E histology images and transcriptomic data are publicly available via the GTEx Portal v10 (https://gtexportal.org/home/). Germline variant data, donor phenotypes and metadata (including chronological age, lifestyle and clinical variables) are controlled-access and were obtained under approved data use (General Research Use; phs000424.v10.p2.c1). Mean-pooled histology features generated in this study are deposited on Zenodo (DOI: 10.5281/zenodo.17042244). Source data for figures are provided in the Supplementary Information / Supplementary Tables.

Research involving human participants, their data, or biological material

Policy information about studies with human participants or human data. See also policy information about sex, gender (identity/presentation), and sexual orientation and race, ethnicity and racism.

Reporting on sex and gender	Analyses were based on genetic sex as recorded in GTEx donor metadata; gender identity was not available and was not used. All structural-aging analyses were performed both pooled and stratified by sex, and sex-specific patterns are reported throughout (e.g., female-donor reproductive vs male-donor reproductive and digestive tissues). Sex-disaggregated results are provided in the figures and Supplementary Information.
Reporting on race, ethnicity, or other socially relevant groupings	Race/ethnicity were not used as variables in this study. These attributes are available only as controlled-access GTEx metadata and were not included in any analysis; no findings are stratified by race or ethnicity.
Population characteristics	The cohort comprises 970 postmortem GTEx donors aged 21–70 years (25,306 H&E whole-slide images across 40 tissues); the germline analysis used 970 donors and the whole-exome VCF contained all 970 individuals. Donors represent the GTEx postmortem population; covariate detail (exact age, clinical and lifestyle variables) is controlled-access via dbGaP (phs000424.v10.p2) and was used under approved authorization.
Recruitment	No participants were recruited for this study. All data derive from the existing GTEx resource, in which postmortem donors were enrolled by GTEx under its own protocols with consent or next-of-kin authorization. As a secondary analysis of pre-collected data, no recruitment or self-selection bias was introduced by this study; any sampling characteristics are inherited from the GTEx cohort design.
Ethics oversight	This study analyzed de-identified existing GTEx data; no new participants or biospecimens were involved. GTEx tissue procurement and data generation were approved by the institutional review boards of the participating sites, with appropriate consent/authorization. Controlled-access GTEx data were obtained under authorized data-use approval (dbGaP accession phs000424.v10.p2; project #39103). This research complied with all relevant ethical regulations.

Note that full information on the approval of the study protocol must also be provided in the manuscript.

Field-specific reporting

Please select the one below that is the best fit for your research. If you are not sure, read the appropriate sections before making your selection.

☒ Life sciences ☐ Behavioural & social sciences ☐ Ecological, evolutionary & environmental sciences

For a reference copy of the document with all sections, see nature.com/documents/nr-reporting-summary-flat.pdf

Life sciences study design

All studies must disclose on these points even when the disclosure is negative.

Sample size	No statistical method was used to predetermine sample size; all eligible GTEx donors meeting the inclusion criteria were analyzed. The structural-aging analysis used 25,306 H&E whole-slide images from 970 postmortem donors across 40 tissues (ages 21–70 years). Tissues were retained only if they had ≥200 samples; molecular-trajectory analyses required ≥150 samples per tissue, and sliding-window and cross-tissue correlation analyses required ≥10 paired observations. The germline analysis used the same 970 donors (whole-exome sequencing). Sample sizes were determined by data availability in the GTEx resource rather than by a power calculation, and are large relative to comparable human tissue-aging studies.
Data exclusions	Exclusion criteria were pre-established. Tissues were excluded if they lacked H&E images, had <200 samples, or failed to show age-associated morphological change (PC1–2 variance explained by age $r^2 < 0.15$, or young-vs-old LDA AUC < 0.8). Image patches were excluded if >50% of pixels showed low Sobel gradient magnitude (non-tissue). Structural-aging rate points were excluded if fewer than 10 samples were available in either age window, and tissues were retained only if ≥80% of their estimated rates were statistically significant. Genes/CpGs with missing data in either comparison group were excluded from differential analyses. Twenty additional tissues (≥200 cases) that did not meet all inclusion criteria were excluded from the main analysis and reported separately in the Supplementary Information.
Replication	This is a secondary computational analysis; no new experiments were performed, so wet-lab replication does not apply. Robustness was instead assessed computationally: structural trajectories were reproduced using three independent pathology foundation models (UNI v2, CONCH v1.5, Virchow2), and trajectory fits were cross-validated by agreement between two independent smoothing methods (spline and Gaussian Process regression). All computational analyses are fully reproducible from the deposited code and data. All replication attempts (alternative encoders) reproduced the main structural patterns.
Randomization	Randomization is not applicable. This is an observational secondary analysis of existing GTEx donor data with no experimental intervention or group allocation. Where relevant, analyses were stratified by tissue and by donor sex; biological covariates inherent to the GTEx cohort (e.g., age, sex) were handled by stratification rather than randomization.
Blinding	Blinding is not applicable. As an observational computational study with no experimental groups or interventions, there was no allocation to conceal. All analyses were automated and applied uniformly across samples; investigators did not manually assign or score outcomes, minimizing subjective bias, though they were not formally blinded to donor age or tissue identity.

Reporting for specific materials, systems and methods

We require information from authors about some types of materials, experimental systems and methods used in many studies. Here, indicate whether each material, system or method listed is relevant to your study. If you are not sure if a list item applies to your research, read the appropriate section before selecting a response.

Materials & experimental systems

n/a	Involved in the study	
☒	☐	Antibodies
☒	☐	Eukaryotic cell lines
☒	☐	Palaeontology and archaeology
☒	☐	Animals and other organisms
☐	☒	Clinical data
☒	☐	Dual use research of concern
☒	☐	Plants

Methods

n/a	Involved in the study	
☒	☐	ChIP-seq
☒	☐	Flow cytometry
☒	☐	MRI-based neuroimaging

Clinical data

Policy information about clinical studies

All manuscripts should comply with the ICMJE guidelines for publication of clinical research and a completed CONSORT checklist must be included with all submissions.

Clinical trial registration	This study is not a clinical trial. It is a secondary, observational analysis of de-identified postmortem human tissue and associated clinical/pathology annotations from the existing Genotype-Tissue Expression (GTEx) project, with no prospective intervention or

enrollment. Trial registration is therefore not applicable; no registration number exists.

Study protocol

No prospective study protocol applies, as no new participants, interventions, or data collection were involved. Donor enrollment and tissue/data collection were performed by the GTEx consortium under its own approved protocols. The analysis plan for this study is described in full in the Methods.

Data collection

No data were collected by the authors. All clinical, pathology, demographic, transcriptomic, methylation, genotype and histology data derive from the GTEx project (dbGaP accession phs000424.v10.p2), accessed under authorized data use (project #39103). Controlled-access clinical and demographic variables were obtained via dbGaP; open-access histology and transcriptomic data via the GTEx Portal v10. Data span 970 postmortem donors aged 21–70 collected by GTEx; collection timing and settings are as described in the GTEx project documentation.

Outcomes

Outcomes were defined post hoc for this analysis and were not pre-registered clinical endpoints.

Plants

Seed stocks

n/a

Novel plant genotypes

n/a

Authentication

n/a
