# Supplementary material for: Mapping structural aging across human tissues reveals tissue-specific trajectories and coordinated deterioration
Source: Nat Aging. 2026 Aug 31;6(9):2013–27. doi: 10.1038/s43587-026-01200-4 (PMC13577920; doi:10.1038/s43587-026-01200-4)
Supplement: Supplementary file 3 [file 43587_2026_1200_MOESM3_ESM.pdf]

nature portfolio

Peer Review File

Mapping structural aging across human tissues reveals tissue-specific trajectories and coordinated deterioration

Corresponding Author: Dr Sanju Sinha

Version 0:

Reviewer comments:

Reviewer #1

(Remarks to the Author)
The manuscript has signifcanly improved and my comments have been largely addressed.

(Remarks on code availability)

Reviewer #2

(Remarks to the Author)
All my previous comments have been addressed.

(Remarks on code availability)

Reviewer #3

(Remarks to the Author)
I appreciate the authors' efforts in addressing the concerns raised during the previous review cycle. The refined definition of "structural aging" is now more precise, and the additional data provided strengthens the core claims. Please find my point-by-point evaluation below:

Points 1–2: I commend the shift toward more neutral terminology regarding "structural aging." Defining it as a spectrum of degenerative, adaptive, and neutral variations prevents over-simplification. The authors also demonstrate the model's potential to distinguish specific age groups.

However, concerns arise with the new data. Importantly, the definition of young [21-30 years] vs. old [51-60 years] is problematic. This is a selective subgroup and may not represent the population across the full age spectrum. Why not use binary grouping <50 or >50? I would recommend reporting multi-class performance with a 10-year span, for tissue types with a sufficient sample size.

There's inconsistency with two different versions for old age: 51-60 and 60-69 years. The AUC is not uniformly high across tissues, e.g., for breast, only 0.59. Some tissue types have very few (<10) samples within specific age groups. AUC has known limitations of being inflated in highly unbalanced datasets. Other more appropriate metrics should also be reported.

Point 3: The updated visualizations provide much-needed transparency. However, because low-degree (n=3) polynomials naturally tend to produce these specific patterns, I suggest the authors explicitly document the parameters used for their spline interpolation. This would help readers distinguish between biological trends and potential interpolation artifacts.

Points 4–6: The clarified data selection criteria and the detailed breakdown of patient demographics across groups address my previous concerns regarding cohort transparency.

Points 7–8: The inclusion of technical comparisons across different foundation models improves the rigor of the findings. Additionally, the justification for utilizing mean-pooling is technically sound and well-reasoned.

(Remarks on code availability)

Reviewer #4

(Remarks to the Author)

(Remarks on code availability)

Version 1:

Reviewer comments:

Reviewer #3

(Remarks to the Author)
All my comments have been addressed.

(Remarks on code availability)

Open Access This Peer Review File is licensed under a Creative Commons Attribution 4.0 International License, which permits use, sharing, adaptation, distribution and reproduction in any medium or format, as long as you give appropriate credit to the original author(s) and the source, provide a link to the Creative Commons license, and indicate if changes were made.
In cases where reviewers are anonymous, credit should be given to 'Anonymous Referee' and the source.
The images or other third party material in this Peer Review File are included in the article's Creative Commons license, unless indicated otherwise in a credit line to the material. If material is not included in the article's Creative Commons license and your intended use is not permitted by statutory regulation or exceeds the permitted use, you will need to obtain permission directly from the copyright holder.
To view a copy of this license, visit https://creativecommons.org/licenses/by/4.0/

Reviewers' Comments:

Reviewer #1 (Remarks to the Author):
The manuscript has significantly improved and my comments have been largely addressed.
Thank you and we appreciate your efforts.

Reviewer #2 (Remarks to the Author):
All my previous comments have been addressed.
Thank you and we appreciate your efforts.

Reviewer #3 (Remarks to the Author):

I appreciate the authors' efforts in addressing the concerns raised during the previous review cycle. The refined definition of "structural aging" is now more precise, and the additional data provided strengthens the core claims. Please find my point-by-point evaluation below:
Thank you!

Points 1–2: I commend the shift toward more neutral terminology regarding "structural aging." Defining it as a spectrum of degenerative, adaptive, and neutral variations prevents over-simplification. The authors also demonstrate the model's potential to distinguish specific age groups.
Thank you!

However, concerns arise with the new data. Importantly, the definition of young [21-30 years] vs. old [51-60 years] is problematic. This is a selective subgroup and may not represent the population across the full age spectrum. Why not use binary grouping <50 or >50? I would recommend reporting multi-class performance with a 10-year span, for tissue types with a sufficient sample size.

There's inconsistency with two different versions for old age: 51-60 and 60-69 years. The AUC is not uniformly high across tissues, e.g., for breast, only 0.59. Some tissue types have very few (<10) samples within specific age groups. AUC has known limitations of being inflated in highly unbalanced datasets. Other more appropriate metrics should also be reported.

The reviewer is right to point out that the original binary comparison (21–30 vs. 51–60) was a selective subgroup analysis and did not represent the full age spectrum. Thus, as suggested, we replaced this binary comparison with a multiclass classification across all six GTEx decade bins (20–29 through 70–79 with 10 year gap), covering the full sampled age range. Further, to address

some issues or age-range with low sample number, tissues with fewer than 100 total samples or fewer than 10 samples in any bin were excluded. The full-result is presented in **Figure L1.**

*Briefly, we trained a multiclass logistic regression classifier on PC1 and PC2, evaluated by stratified 5-fold cross-validation. We report macro-averaged one-vs-rest AUC to ensure our performance is not driven by one class with many samples (**Figure L1B**). This Macro-AUC over classes represents the average ability of a model to segregate a class from the rest of the classes.*

Aligning with previous results, the ovary and uterus showed the highest segregation power (macroAUC of 0.73 and 0.72, respectively). Our 15 selected tissues focused in the manuscript have an average macro-AUC of 0.66. We observed that the young tissues (Age 20-29) are easiest to segregate (one-vs.rest AUC=0.75), and the 50-59 age group was hardest to segregate (mean OVR AUC = 0.531). When we retrained the multiclass classifier after excluding the difficult 50–59 decade, the mean macro-AUC increased from 0.655 to 0.703.

*Overall, this adds nuance to our conclusion that (quoted from manuscript, Results Page 8-9) “Major PCs of morphology (PC1-2) readily separated young from old samples in many tissues without any training on age (**Figure 3**), indicating that aging, not individual variation or batch effects, explains majority of the structural change and might be the driver.”* We reiterate that this should not be compared to any other clock's ability to predict age, as we only used PC1-2 of morphology above. A supervised clock based on complete-morphology predicts chronological age with Pearson’s R=0.79 in 15 selected tissues and with R=0.7 across all tissues.

We accordingly added a sentence to add this after above in main text: “To quantify this separation, we measured both the age-related variance captured by PC1–2 (cross-validated OLS, Table S1) and the ability to distinguish age groups with 10-year bins (cross-validated logistic regression, Notes S6, Table S2). We find the young tissues (Age 20-29) are easiest to segregate (one-vs.rest AUC=0.75, Figure S11), and the 50-59 age group was hardest to segregate (mean OVR AUC = 0.531).”

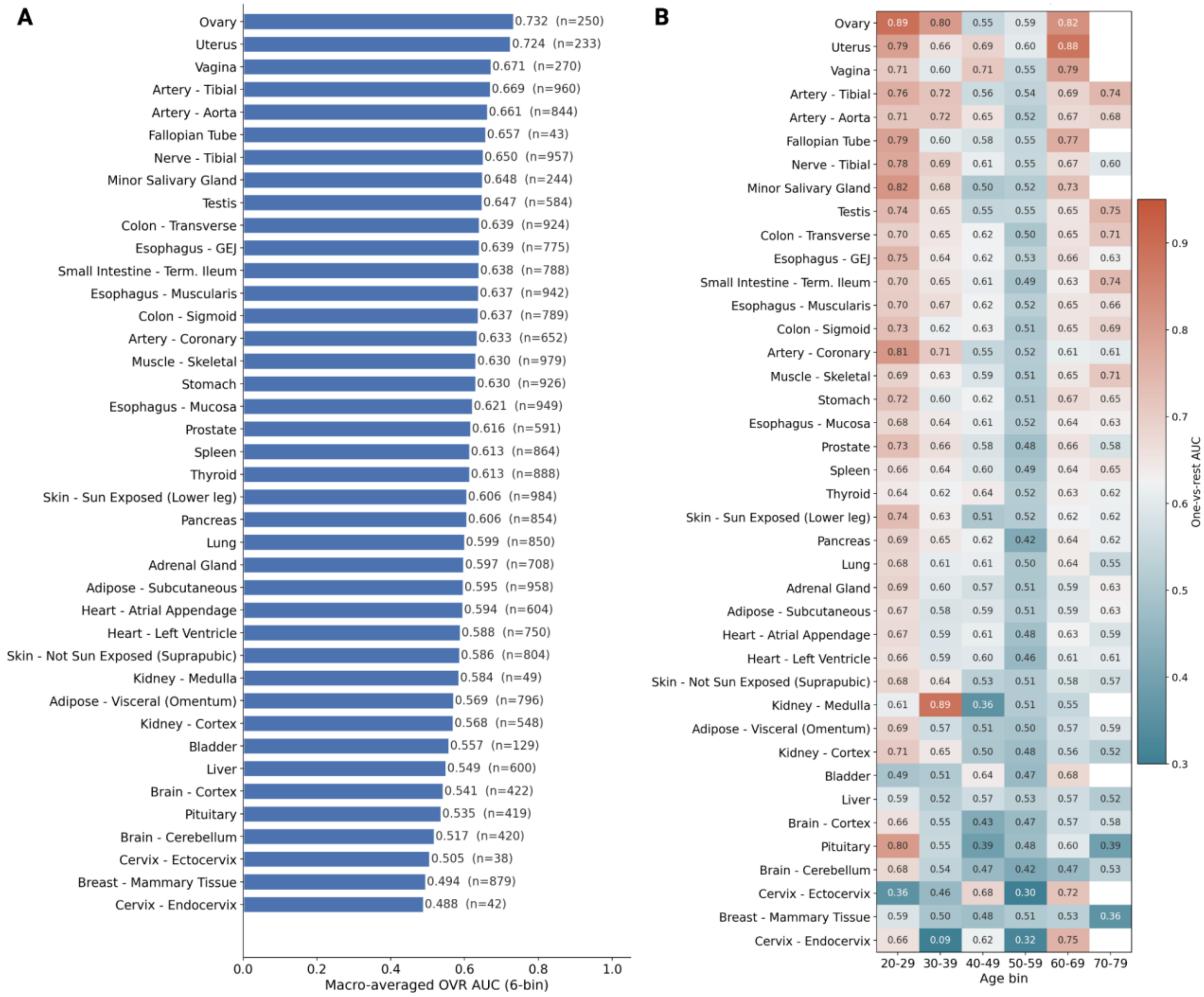

Figure L1 (also Figure S11) : (A) *Macro-averaged one-vs-rest AUC for multiclass logistic regression classification across GTEx age-decade bins using only PC1 and PC2 of UNI-derived morphology features.* ***(B)*** *Per-age-bin one-vs-rest AUCs for each tissue. Rows are ordered by macro-AUC. Stronger separation is observed for the youngest and oldest age bins, whereas the 50–59 decade shows weaker discrimination across most tissues.*

Point 3: The updated visualizations provide much-needed transparency. However, because low-degree (n=3) polynomials naturally tend to produce these specific patterns, I suggest the authors explicitly document the parameters used for their spline interpolation. This would help readers distinguish between biological trends and potential interpolation artifacts.

We agree that this will help. We have added a paragraph in method section with suggested details as following: **Methods, Page 22:** <u>*Generating Structural Aging Trajectory:*</u> *For spline smoothing, we employed scipy's Univariate Spline with a smoothing factor s =*

(window_size / n) × 0.5 × n, where window_size = 10 and n is the number of data points per tissue. This formulation makes s scale linearly with n, ensuring comparable smoothing behavior across tissues regardless of sample size. Crucially, rather than fixing the polynomial degree at the default cubic (k=3), we allowed the spline degree to be determined data-adaptively by the smoothing parameters, meaning the fitted curve is not constrained to a low-degree polynomial but instead balances fidelity to the data against smoothness. To further distinguish biological trends from interpolation artifacts, we complemented spline fits with Gaussian Process smoothing using an RBF kernel, with length scale set to min(0.5, (window_size / n) × 2) and noise level = 0.1 × (window_size / 5). Agreement between both methods was used as a criterion for selecting the best-fit trajectory per tissue.

Points 4–6: The clarified data selection criteria and the detailed breakdown of patient demographics across groups address my previous concerns regarding cohort transparency.
Thanks!

Points 7–8: The inclusion of technical comparisons across different foundation models improves the rigor of the findings. Additionally, the justification for utilizing mean-pooling is technically sound and well-reasoned.
Thanks!
